# Relay Approach: A Convergent Synthesis of Key Fragments en route to (+)‐Neosorangicin A

**DOI:** 10.1002/chem.71360

**Published:** 2026-07-02

**Authors:** Marvin Wenninger, Maxim Munt, Le Chang, Rositsa Stoykova, Roberto Ojcius, Oliver Spieß, Dieter Schinzer

**Affiliations:** ^1^ Institut für Chemie Otto‐von‐Guericke‐Universität Magdeburg Magdeburg Germany

**Keywords:** (+)‐neosorangicin A, convergent synthesis, dioxabicyclo[3.2.1]octane, Grubbs metathesis, macrolide antibiotic

## Abstract

Herein, we report the synthesis of the three key fragments of the macrolide antibiotic (+)‐neosorangicin A, a compound exhibiting potent activity against multiple pathogens. The tetrahydropyran unit (THP) was prepared via stereocontrolled Brown crotylation, followed by hydroboration and epoxide‐opening/cyclization steps. Subsequent coupling with the known dioxabicyclo[3.2.1]octane fragment (BCO) via Grubbs metathesis furnished the THP–BCO key intermediate on a gram scale. This intermediate was further transformed into an *E*‐vinyl iodide for a late‐stage Stille coupling with the dihydropyran fragment (DHP), incorporating a shortened side chain relative to (+)‐sorangicin A and bearing a secondary alcohol. This fragment was synthesized via 1,2‐addition to a dihydropyran‐methyl ketone followed by dehydration. Subsequent functional group manipulations enabled access to a phosphonate intermediate, which was converted into a stannylated *Z*,*Z*‐diene via Ando olefination. The Stille coupling between the THP–BCO and the DHP fragments provided the carbon skeleton of the natural product and was confirmed by a relay approach using isolated (+)‐neosorangicin A. This work establishes a selective route to the key building blocks, providing a foundation for the convergent total synthesis of (+)‐neosorangicin A and a proof of concept via a relay approach.

## Graphical abstract

A selective synthesis of three key fragments of the antibiotically active (+)‐neosorangicin A is reported, providing a foundation for its convergent total synthesis and proof of strategy via a relay approach.

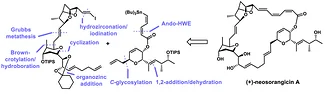

## 1 Introduction

**FIGURE 1:**
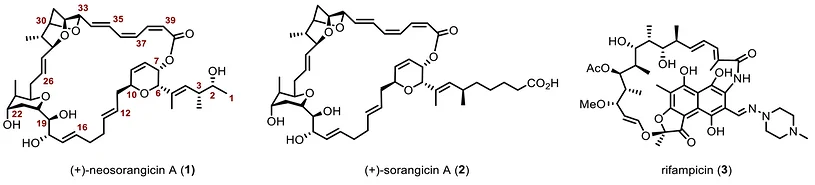
Selected members of the sorangicin family (**1**, **2**) and rifampicin (**3**).

Macrolide polyether natural products constitute an important class of antibiotics, distinguished by their complex molecular architectures and potent biological activities. Among these, the sorangicin family was first described in 1985, when Höfle and coworkers isolated and structurally characterized (+)‐sorangicin A (**2**, Figure 1) from the myxobacterium *Sorangium cellulosum* strain So ce12 at the former German Research Centre for Biotechnology [1]. Structural elucidation revealed a highly elaborated 31‐membered macrolactone featuring a tetrasubstituted tetrahydropyran ring, a trisubstituted dihydropyran ring, a rare dioxabicyclo[3.2.1]octane motif, and a sensitive (*E*,*Z*,*Z*)‐trienyl ester functionality. Recently, Müller, Herrmann, and coworkers reported the isolation of (+)‐neosorangicin A (**1**) from a different strain of the same organism (So ce439) [2]. This congener is closely related to (+)‐sorangicin A (**2**), differing in the side chain attached to the dihydropyran ring, which bears a chiral secondary alcohol at C‐2 and an inverted methyl group at C‐3 (Figure 1). The neosorangicin biosynthetic gene cluster (nsr BGC) from *Sorangium cellulosum* Soce417 is highly similar to the sorangicin pathway but exhibits distinct domain differences, including additional ACP, KR, DH, and *O*‐methyltransferase domains. These variations likely account for the structural differences between (+)‐neosorangicin A (**1**) and (+)‐sorangicin A (**2**), although key steps such as pathway initiation, specific oxidation events, and macrocyclization remain unresolved [2]. Additionally, extensive NMR studies of Müller, Herrmann, and coworkers could not finally reveal the exact configuration at C‐3, leading to a proposal based on the biosynthesis for both sorangicins. The present study was planned based on the assigned stereochemistry from an earlier patent [3], with the aim to confirm the absolute configuration by total synthesis.

Biologically, both macrolides (**1** and **2**) show broad antibacterial activity against clinically relevant Gram‐positive and Gram‐negative pathogens, including methicillin‐resistant *Staphylococcus aureus* and *Mycobacterium tuberculosis*. Notably, (+)‐neosorangicin A (**1**) shows significantly higher efficacy against several pathogens, with MIC values ranging from 0.25 – 8 µg/mL against several ESKAPE pathogens, than the already potent (+)‐sorangicin A (**2**) [2]. Mechanistic studies, including cryo‐electron microscopy investigations by Darst and coworkers, demonstrated that (+)‐sorangicin A (**2**) inhibits bacterial RNA polymerase through a mechanism overlapping with that of the first‐line antibiotic rifampicin (**3**), likely resulting from binding into the same β‐subunit pocket of the RNA polymerase [4, 5]. These findings highlight the therapeutic relevance of the sorangicin scaffold.

Although both (+)‐sorangicin A (**2**) and (+)‐neosorangicin A (**1**) can be obtained through fermentation, biotechnological production remains limited in terms of scalability and does not readily allow structural modifications. Therefore, the development of an efficient and stereoselective synthetic route to (+)‐neosorangicin A (**1**) represents an important objective at the interface of natural product synthesis and antibiotic drug discovery.

To date, only one total synthesis has been reported for (+)‐sorangicin A (**2**) by the group of Smith III [6, 7, 8, 9, 10]) and a formal total synthesis by Crimmins and co‐workers [11, 12], as well as the construction of the dioxabicyclo[3.2.1]octane (BCO)‐tetrahydropyran (THP) fragment [13]. Furthermore, the work of several research groups, including ours [14, 15, 16], should be highlighted, particularly those focusing on the synthesis of the dihydropyran (DHP) [17, 18, 19], BCO [20, 21], or the THP [22, 23, 24] fragment of (+)‐sorangicin A (**2**). While the stereochemistry at the C‐7 position was initially unclear, the configuration was later corrected by Höfle and coworkers [7, 25]. Since the modified side‐chain significantly improves the biological activity of (+)‐neosorangicin A (**1**), we aimed to develop a total synthesis of the potent macrolide (+)‐neosorangicin A (**1**).

**SCHEME 1:**
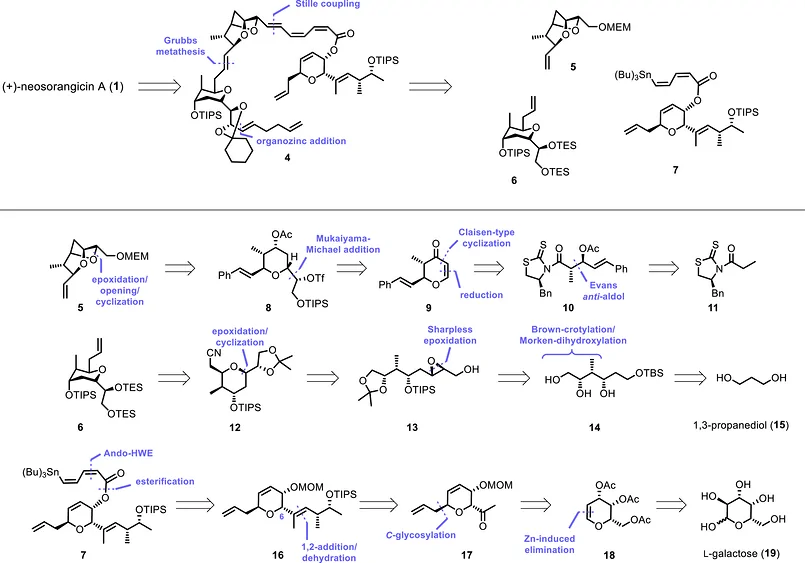
Retrosynthetic analysis for the synthesis of (+)‐neosorangicin A (**1**) (top) and the key fragments **5**, **6**, and **7** (bottom).

The total synthesis of (+)‐neosorangicin A (**1**) is designed to proceed via late‐stage ring‐closing metathesis to form the 31‐membered macrolactone, followed by a final global deprotection (Scheme 1). The critical ring‐closing metathesis is expected to deliver the macrocycle while minimizing undesired *E*/*Z* isomerization. The precursor **4** is assembled through a modified Stille coupling between an *E*‐vinyl iodide, arising from a Grubbs metathesis product of the BCO **5** and THP **6** fragments, and a stannylated *Z*,*Z*‐dienoate **7**. The BCO fragment **5** was synthesized from acylated Evans‐auxiliary **11** following our previous route [16] employing an Evans *anti*‐aldol reaction, a Mukaiyama‐Michael addition of a ketal, and final epoxide opening. The THP fragment **6** should be accessible from 1,3‐propanediol (**15**) via Brown crotylation, followed by Morken‐dihydroxylation, cyclization by opening of epoxide **13** after acetonide deprotection. The stannylated *Z*,*Z*‐dienoate **7**, which forms the dihydropyran fragment (DHP), is prepared via esterification followed by an Ando olefination. Installation of the side chain at C‐6 of compound **16** will be achieved by a stereoselective organometallic addition to methyl ketone **17**, derived from L‐galactose (**19**) via Zn‐induced elimination and *C*‐glycosylation.

## 2 Results and Discussion

### 2.1 THP Fragment

The synthesis of the tetrahydropyran fragment commenced with mono‐TBS protection and subsequent

**SCHEME 2:**
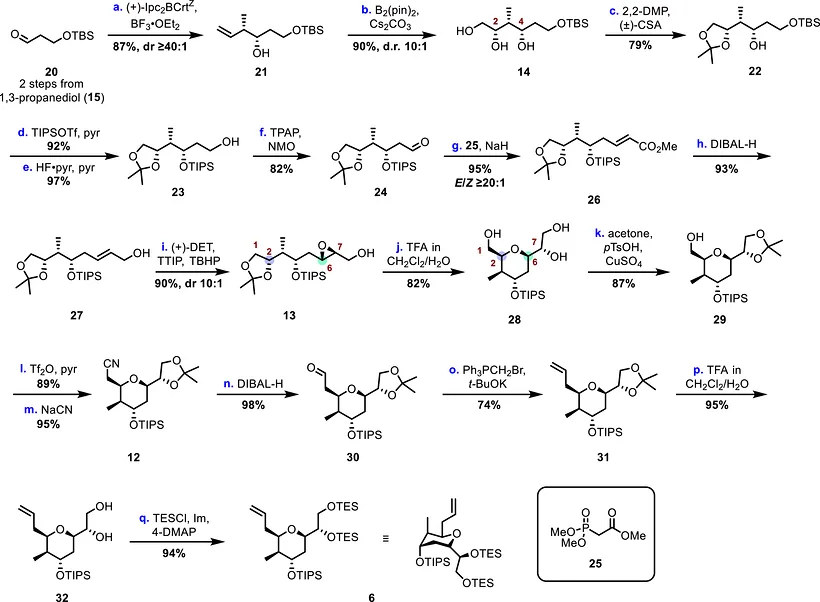
Synthesis of THP‐fragment **6**. Conditions: Aldehyde **20** accessible via 2 steps from 1,3‐propanediol (**15**) (82% yield, see Supporting Information); (a) (+)‐Ipc_2_BCrt^Z^, BF_3_•OEt_2_ *then* NaOH, H_2_O_2_, Et_2_O, –78°C, 87%, 3 h, dr ≥ 40:1; (b) B_2_(pin)_2_, Cs_2_CO_3_, MeOH *then* NaOH, H_2_O_2_, THF, r.t. to 70°C, 90%, 3 h, dr 10:1; (c) 2,2‐DMP, (±)‐CSA, 3Å MS, CH_2_Cl_2_, 0°C, 1 h, 79%; (d) TIPSOTf, pyr, CH_2_Cl_2_, –78°C to r.t., 1 h, 92%; (e) HF•pyr, THF/pyr, 0°C to r.t., 4 h, 97%; (f) TPAP, NMO, 3Å MS, CH_2_Cl_2_, r.t., 1 h, 82%; (g) **25**, NaH, THF, 0°C to r.t., 80 min, 95%, *E*/*Z* ≥20:1; (h) DIBAL‐H, CH_2_Cl_2_, –78°C, 1 h, 93%; (i) (+)‐DET, TTIP, TBHP, 3Å MS, CH_2_Cl_2_, –78 to –25°C, 20.5 h, 90%, dr 10:1; (j) TFA/CH_2_Cl_2_/H_2_O, 0°C, 1 h, 82%; (k) acetone, *p*TsOH•H_2_O, CuSO_4_, 0°C, 1 h, 87%; (l) Tf_2_O, CH_2_Cl_2_, –78°C, 30 min, 89%; (m) NaCN, DMSO, r.t., 1 h, 95%; (n) DIBAL‐H, CH_2_Cl_2_, –78°C, 2 h, 98%; (o) Ph_3_PMeBr, *t*‐BuOK, THF, r.t., 2.5 h, 74%; (p) TFA/CH_2_Cl_2_/H_2_O, 0°C, 1 h, 95%; (q) TESCl, Im, 4‐DMAP, CH_2_Cl_2_, r.t., 16 h, 94%; (+)‐Ipc_2_BCrt^Z^ = (+)‐diisopinocampheylcrotylborane (*Z*‐configuration), TPAP = tetra‐*n*‐propylammonium perruthenate, DET = (*R*,*R*)‐diethyl tartrate, TTIP = titanium(IV) isopropoxide, Im = 1*H‐*imidazole.

Parikh–Doering oxidation of 1,3‐propanediol (**15**), affording aldehyde **20** in 82% yield (see Supporting Information). Aldehyde **20** was transformed via Brown crotylation into alcohol **21** in 87% yield and high diastereoselectivity (dr ≥ 40:1) using 5.2 equivalents of *cis*‐2‐butene and constructing both stereocenters at C‐3 and C‐4 (Scheme 2) [26, 27, 28]. Subsequent dihydroxylation by a modified Brown hydroboration reported by the Morken group [29] afforded triol **14** in good yield on a multi‐gram scale (*syn*/*anti*, 10:1). To assign whether the 2‐OH/4‐OH groups are *syn* or *anti* oriented, the primary alcohol group was protected with TBS and the diol of the resulting TBS ether was protected with 2,2‐DMP (see Supporting Information). The coupling constant between 2‐H and 3‐H was found to be *J* = 2.2 Hz, confirming that the *syn*‐diol was successfully installed.

Next, the 1,2‐diol moiety was protected as the acetonide **22** with 2,2‐DMP and (±)‐CSA. Protection of the remaining hydroxy group with TIPSOTf afforded the fully masked tetraol, which was further subjected to deprotection of the TBS moiety with HF•pyridine, while using a crucial excess of pyridine to achieve alcohol **23** with the excellent yield of 97%. Subsequent Ley–Griffith oxidation afforded aldehyde **24** in good yield setting the stage for the chain elongation using a Horner–Wadsworth–Emmons reaction. This step was concluded by the reaction with trimethyl phosphonoacetate (**25**) and afforded *α*,*β*‐unsaturated ester **26** in excellent yield and *E*/*Z* ratio (95%, *E*/*Z* ≥20:1). Thereafter, a DIBAL‐H reduction of the ester furnished allylic alcohol **27** in excellent yield, paving the way for the Sharpless epoxidation. The required epoxide **13** was successfully isolated in 90% yield and a dr ≥10:1 after 20.5 h at −25°C using standard conditions. The critical deprotection and necessary epoxide opening at C‐7 could be achieved when using a mixture of dichloromethane, trifluoroacetic acid, and water, affording triol **28** in 82% yield in 1 h at 0°C. However, conditions like (±)‐CSA failed in our hands [30]. Again, acetonide protection of the diol and triflation of the primary alcohol with Tf_2_O resulted in the formation of triflate **28a** in excellent yield (see Supporting Information). Notably, this triflate was found to be unstable and was used directly for the substitution with sodium cyanide in DMSO, forming nitrile **12** in high yield. For the implementation of the terminal alkene, DIBAL‐H was used once more to reduce the nitrile to the corresponding aldehyde **30** in nearly quantitative yield. After Wittig olefination with Ph_3_PMeBr, the acetonide protecting group of the obtained olefin **31** was cleaved under the same conditions as used during the conversion of compound **13** to **28**. Both hydroxy groups were lastly protected as their TES ether **6** to provide the feasible THP‐fragment for a later introduction of the diene linker [11]. In summary, the THP‐fragment **6** could be synthesized over 19 steps in 11.5% overall yield from 1,3‐propanediol (**15**).

### 2.2 DHP Fragment

**SCHEME 3:**
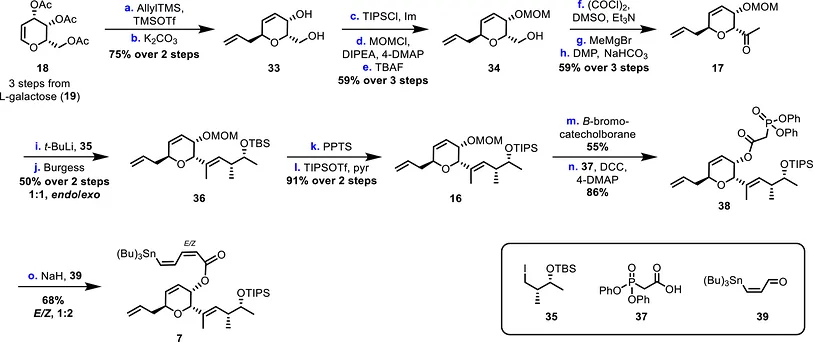
Synthesis of the DHP‐fragment **7**. Conditions: D‐Galactal 18 accessible via 3 steps from L‐galactose (**19**) (66% yield, see Supporting Information); (a) AllylTMS, TMSOTf, MeCN, 0°C, 1 h; (b) K_2_CO_3_, MeOH, r.t., 4 h, 75% over 2 steps; (c) TIPSCl, Im, CH_2_Cl_2_, r.t., o. n., 67%; (d) MOMCl, DIPEA, 4‐DMAP, CH_2_Cl_2_, r.t., o. n., 94%; (e) TBAF, THF, 0°C, 2.5 h, 93%; (f) (COCl)_2_, DMSO, Et_3_N, CH_2_Cl_2_, −78°C, 1 h; (g) MeMgBr, Et_2_O, −78°C to r.t., o. n.; (h) DMP, NaHCO_3_, CH_2_Cl_2_/H_2_O, r.t., 1 h, 59% over 3 steps; (i) *t*‐BuLi, **35**, THF, −78°C, 1 h; (j) Burgess reagent, PhMe, 35°C, 3 h, 50% over 2 steps, 1:1 *endo*/*exo*; (k) PPTS, MeOH, r.t., 24 h; (l) TIPSOTf, pyr, CH_2_Cl_2_, 0°C, 1 h, 91% over 2 steps; (m) *B*‐Bromocatecholborane, CH_2_Cl_2_, −78°C, 1 h, 55%; (n) **37**, DCC, 4‐DMAP, THF, r.t., 2 h, 86%; (o) NaH, THF, 0°C, 15 min *then* **39**, THF, −78 to 0°C, 1 h, 68%, *E*/*Z*, 1:2.

The dihydropyran fragment was synthesized from L‐galactose (**19**), which was obtained from D‐galactose in six steps according to a slightly modified protocol [31] (see Supporting Information). Starting with L‐galactose (**19**), acetylated L‐galactal **18** was synthesized in three steps and subsequently subjected to a Ferrier rearrangement‐like *C*‐glycosylation with allyltrimethylsilane [32, 33, 34] and the resulting dihydropyran **18a** was deprotected under basic conditions, affording diol **33** in 75% over two steps (Scheme 3). Orthogonal MOM and TIPS protection, followed by TBAF‐mediated deprotection, allowed the synthesis of alcohol **34** in good yield over three steps. For installation of the methyl group of the *endo*‐double bond chain at C‐5, the use of a methyl ketone was essential. Accordingly, a sequence of Swern oxidation, Grignard addition with methylmagnesium bromide and subsequent Dess–Martin periodinane oxidation furnished methyl ketone **17** in 59% yield over three steps.

To introduce the alkyl side chain of (+)‐neosorangicin (**1**), iodide **35** was synthesized from acetaldehyde via a four‐step sequence, including a Brown crotylation to install both stereocenters at the C‐2 and C‐3 positions (based on the numbering of **1**). This was followed by TBS protection of the secondary alcohol, ozonolysis, and S_N_2 substitution of the tosylated intermediate with sodium iodide (see Supporting Information). If the stereochemistry at C‐2 and C‐3 proves to be *anti* in contrast to the proposed structure, the synthetic route can be readily adapted by adjusting the Brown crotylation conditions. Our first attempt to convert iodide **35** into the corresponding ylide and subject it to Wittig conditions failed. The synthesis of a benzothiazole‐sulfone and phenyltetrazole‐sulfone to perform a Julia–Kocienski olefination [35] was successful; however, the best outcome was an inseparable 1:1 mixture of the *E*/*Z* products (C4‐C5) in 20% yield. Finally, the introduction via organometallic addition was conducted: the use of *t*‐BuLi at low temperature was necessary to achieve a full consumption of the starting material. It was clear that only one diastereomeric alcohol was needed to conclude with a dehydration, and finally leading to the desired *endo*‐alkene. Our first attempt resulted in an acceptable yield of 60%, which was improved to 72% by modifying the protocol and adding *t*‐BuLi to the mixed reactants. Switching to THF as the solvent further increased the yield to 75%, setting the stage for the dehydration step. After testing several commercially available dehydration reagents like DAST, Martin's sulfurane, and two Burgess reagents, promising results were achieved with the latter reagents – however, the yield remained limited to 36% and an *exo*/*endo* mixture of 3:2. This ratio could not be improved to a significant amount, resulting in a 1:1 mixture of *exo*/*endo*‐products in combined 50% yield over two steps. Unfortunately, the undesired *exo*‐product could not be converted into the *endo*‐product by using different catalytic systems [36, 37, 38, 39].

With alkene **36** in hand, protecting group manipulations were necessary by switching from TBS to TIPS to afford TIPS ether **16** in good yield. This exchange was necessary, since conditions for the subsequent MOM‐deprotection also cleaved the TBS group, forming the corresponding diol. For future experiments, the TIPS group should be introduced directly from the iodide fragment to avoid losses in yield. The removal of the MOM‐protecting group was successfully achieved after extensive experimentation: with *B*‐bromocatecholborane at −95°C, the MOM‐group was cleaved in a moderate yield of 55%, while all other conditions, like HBr in different solvents, TMSBr or AcCl in MeOH, failed in our hands. The sequence continued with the synthesis of phosphonate **38** by using Steglich conditions with 2‐(diphenoxyphosphoryl)acetic acid (**37**), prepared from diphenyl phosphite in two steps (see Supporting Information). Based on our initial results, where Still–Gennari conditions [40] between an alkynal and another phosphonate only afforded the *E*‐isomer (not shown), the Ando variation [41, 42] between phosphonate **35** and aldehyde **39** (three steps from propargyl alcohol, see Supporting Information) allowed the synthesis of DHP‐fragment **7** in 68% yield with an *E*/*Z* ratio of 1:2.

### 2.3 BCO Fragment

**SCHEME 4:**
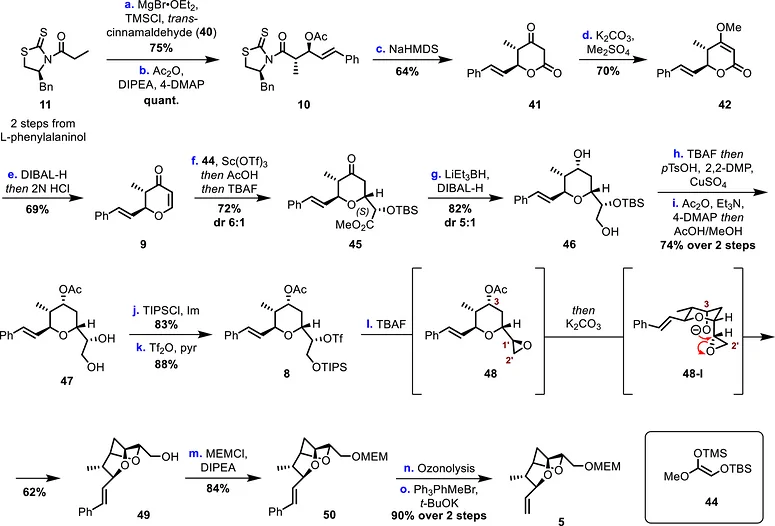
Synthesis of the BCO‐fragment **5**. Conditions: (a) (i) MgBr•OEt_2_, TMSCl, *trans*‐cinnamaldehyde, EtOAc, r.t., 48 h *then* 1N HCl, r.t., 1 h, 75%; (b) Ac_2_O, DIPEA, 4‐DMAP, CH_2_Cl_2_, −30°C, 1 h, quant.; (c) NaHMDS, THF, −78°C, upon completion, 64%; (c) K_2_CO_3_, Me_2_SO_4_, acetone, r.t., o. n., 70%; (e) DIBAL‐H, CH_2_Cl_2_, −78°C, 1 h *then* 2N HCl, r.t., 30 min, 69% over 2 steps; (f) **44**, Sc(OTf)_3_ *then* AcOH *then* TBAF, CH_2_Cl_2_, −78°C to r.t., upon completion, 72%, dr 6:1; (g) LiEt_3_BH, DIBAL‐H, THF, −78 to 0°C, 2 h, 82%, dr 5:1; (h) TBAF, DMF, r.t., 30 min *then p*TsOH•H_2_O, 2,2‐DMP, CuSO_4_, acetone, r.t., o. n.(i) Ac_2_O, Et_3_N, 4‐DMAP, THF, r.t, 4 h *then* AcOH/MeOH, 80°C, 4 h, 74% over 2 steps; (j) TIPSCl, Im, CH_2_Cl_2_, r.t., o. n., 83%; (k) Tf_2_O, pyr, CH_2_Cl_2_, −15°C, 20 min, 88%; (l) TBAF, THF, r.t., 15 min *then* K_2_CO_3_, MeOH, r.t., o. n., 62%; (m) MEMCl, DIPEA, CH_2_Cl_2_, r.t., o. n., 84%; (n) Ozonolysis, MeOH *then* Me_2_S, −78°C to r.t., o. n.; (o) Ph_3_PMeBr, *t*‐BuOK, THF, 0°C, 1.5 h, 90% over 2 steps; MEMCl = 2‐methoxyethoxymethyl chloride.

The BCO fragment was synthesized following our previously reported route, which has now been demonstrated to be readily scalable to gram quantities via the following sequence (Scheme 4) [16]. After the preparation of the Evans auxiliary from L‐phenylalanine, an Evans aldol reaction between the corresponding acylated auxiliary **11** and *trans*‐cinnamaldehyde (**40**) afforded *anti*‐Aldol product **10a** in 75% yield after hydrolysis of the TMS ether (see Supporting Information) [43]. Following this, quantitative acetylation of **10a** at −30°C afforded acetate **10**, which underwent an intramolecular Claisen‐type condensation upon treatment with NaHMDS at −78°C and furnished ketolactone **41** in moderate yield on a multi‐gram scale [44, 45, 46]. Notably, a minor by‐product was observed, where the enolate attacks at the auxiliary to afford a 5,8‐bicyclic lactam [46]. After methylation with dimethylsulfate, subsequent reduction of the lactone with DIBAL‐H followed by exposure to HCl provided pyranone **9** in 69% yield [47, 48]. Next, we envisioned a Mukaiyama–Michael addition between ketal **44**, accessible in two steps from methyl glycolate (**43**) [49], and pyranone **9** [50, 51, 52]. Best results were achieved with 5 mol% of Sc(OTf)_3_ on a gram‐scale and in 72% yield to afford ester **45** with good selectivity (dr 6:1). Ensuring full consumption of pyranone **9** was essential, given the nearly identical R_f_ values of the starting material and ester **45**. This was achieved, using an excess of the ketal **44** and dropwise addition via syringe pump. To access 1,2‐diol **46**, a selective one‐pot double reduction of both the ketone and ester moieties was performed using lithium triethylborohydride and DIBAL‐H, respectively, affording diol **46** as an intermediate (82% yield, dr 5:1).

After deprotection of the TBS group using TBAF, the 1,2‐diol was protected as the acetonide in the same pot. Next, the remaining hydroxy group was acetylated with acetic acid anhydride, and the acetonide protecting group was cleaved by adding an AcOH/MeOH mixture to the reaction, yielding diol **47** in 74% over two steps using only one purification step. The primary hydroxy group was protected using TIPSCl, and the remaining hydroxy group was triflated with Tf_2_O at −15°C in good yield. The obtained unstable triflate **8** was used for the key transformation in the construction of the dioxabicyclo[3.2.1]octane moiety, for which a one‐pot procedure was established: subsequent deprotection of the TIPS group using TBAF at r.t., the formed alkoxide attacks at C‐1’ to eliminate the triflate and forming the corresponding epoxide **48**. After 15 min, dry K_2_CO_3_ was added, which cleaves the acetate group and therefore induces selective opening of the epoxide to provide dioxabicyclo[3.2.1]octane **49** in 62% yield on a ca. 3 g scale. With compound **49** in hand, the primary alcohol was protected as the MEM ether **50**, and the side chain, originating from the Evans aldol reaction, was transformed into the terminal alkene via an ozonolysis/Wittig olefination sequence, furnishing BCO fragment **5** in 76% yield over the final three steps. Overall, BCO‐fragment **5** was synthesized in 3.5% yield over 15 steps from acylated auxiliary **11**.

### 2.4 Endgame

**SCHEME 5:**
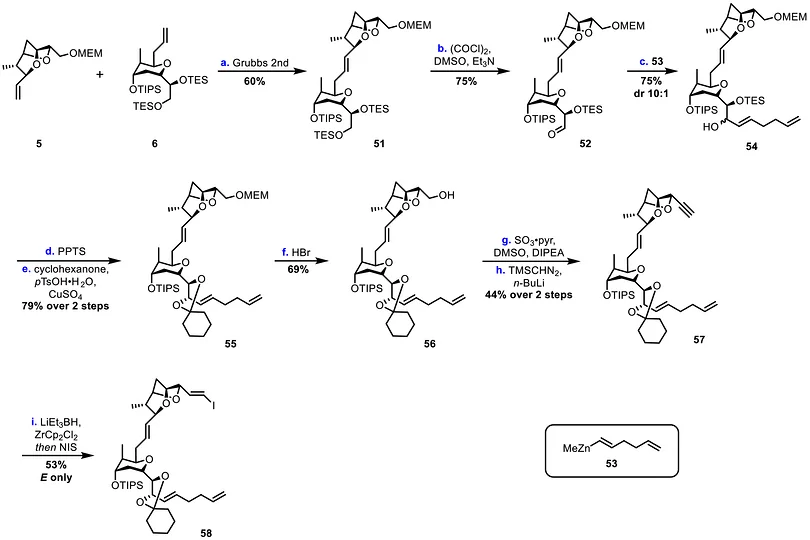
Synthesis of the western part of (+)‐neosorangicin A (**1**). Conditions: (a) Grubbs second, PhMe, r.t., 18 h, 60%; (b) (COCl)_2_, DMSO, Et_3_N, −78°C to r.t., 1.5 h, 75%; (c) **53**, THF, −90 to −20°C, 75%, 2.5 h, dr 10:1; (d) PPTS, THF/MeOH, r.t., 3 h; (e) cyclohexanone, *p*TsOH•H_2_O, CuSO_4_, CH_2_Cl_2_, r.t., 1 h, 79% over 2 steps; (f) HBr, THF, 0°C, 16 h, 69%; (g) SO_3_•pyr; DMSO, DIPEA, CH_2_Cl_2,_ 0°C; 2 h (h) TMSCHN_2_, *n*‐BuLi, THF, −78 to 0°C, 1 h, 44% over 2 steps; (i) LiEt_3_BH, ZrCp_2_Cl_2_, THF, 0°C, 1 h *then* NIS, r.t., 30 min, 53%, *E* only.

For the synthesis of the BCO‐THP fragment **51**, we envisioned an olefin metathesis catalyzed by the Grubbs second catalyst on gram‐scale (Scheme 5). After a promising result on milligram‐scale (67%), our first gram‐scale experiment however, showed up to 45% of the homodimer of the THP fragment, which was used in slight excess (1.2 eq.). Changing the solvent to dichloromethane and heating did not lead to an improved yield, but could lower the amount of by‐product (40% and 22% respectively). Then we tested with portionwise addition of the Grubbs catalyst on gram‐scale, affording 55% of the metathesis product **51**. However, it was observed that simultaneous addition of both the THP fragment and the catalyst over 6 h were the optimal conditions affording olefin **51** in 60% yield, while the dimer was formed in <10% (^1^H NMR). Notably, the homodimer of the THP fragment can be recycled by consecutive ozonolysis and Wittig olefination. Next, a selective Swern oxidation of the primary silyl ether to the corresponding aldehyde **52** was successfully conducted in 75% yield [53]. This was followed by a Cram‐chelate‐controlled addition [54, 55] of organozinc derivative **53**, derived from 5‐hexen‐1‐ol via Takai olefination and treatment with *t*‐BuLi/ZnMe_2_ (see Supporting Information) furnishing alcohol **54** in good yield as a diastereomeric mixture with a dr of 10:1 [11]. The mixture was carried forward due to difficulties in separating the diastereomers, but can be separated by flash chromatography after the following two steps. For stereochemical assignment, removal of the adjacent protecting group followed by acetonide formation enabled derivatization of the resulting diols. Analysis of the major acetonide derivative by NOE experiments allowed the assignment of the relative configuration of the major diastereomer.

At this point, the change of protecting groups towards a 1,2‐diol protecting group was necessary. After unsatisfactory results when using (*i*Pr)_2_Si(OTf)_2_ (diisopropylsilyl bis(trifluoromethanesulfonate)) and 2,2‐DMP in terms of yield and stability for the following MEM‐deprotection, the diol was successfully protected using cyclohexanone and PPTS, providing cyclohexylidene ketal **55** in 79% yield over two steps. Notably, this protecting group should be labile towards a late‐stage global deprotection simultaneously as TIPS. The following MEM‐deprotection was found to be challenging, but it was finally achieved when using trace amounts of hydrogen bromide in acetic acid, leading to alcohol **56** in 69% yield. For the synthesis of the required *E*‐vinyl iodide **58**, the hydroxy group was first transformed into the corresponding aldehyde via Parikh–Doering oxidation, followed by a Colvin rearrangement [56], affording alkyne **57** in moderate yield. To gain reproducible results for the Colvin rearrangement, we found that traces of residual amine of the Parikh–Doering oxidation significantly lowered the yield and must therefore be removed prior to attempting the rearrangement. In a second step, the alkyne **57** was converted into the *E*‐vinyl iodide **58** using the in situ generated Schwartz reagent and treatment of the intermediate with *N*‐iodosuccinimide, affording the desired *E*‐vinyl iodide **58** in 53% yield as a single isomer. This provided the western part of (+)‐neosorangicin A (**1**) in a convergent fashion.

**SCHEME 6:**
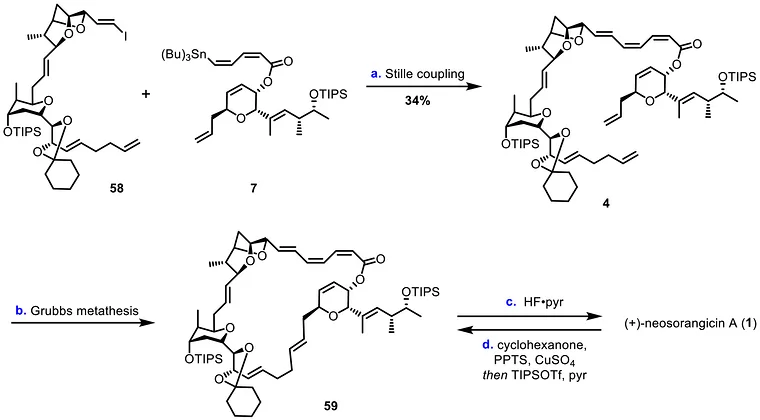
Endgame of the relay approach towards (+)‐neosorangicin A (**1**). Conditions: (a) Ph_2_PO_2_NBu_4_, PdCl_2_(PhCN)_2_, DMF, r.t., 4 h, 34%; (b) Grubbs second, CH_2_Cl_2_, r.t., o. n.; (c) HF•pyr, THF, r.t., 2 d; (d) cyclohexanone, PPTS, CuSO_4_ *then* TIPSOTf, pyr.

With fragment **58** and DHP‐fragment **7** in hand, we investigated the Stille coupling of those molecules. Using PdCl_2_(PhCN)_2_ and tetrabutylammonium diphenylphosphinate, which was used to suppress isomerization of the sensitive triene system, we were able to isolate the coupled product **4** in 34% yield (Scheme 6). The compound was found to be prone to isomerization in the NMR solvent and rapid decomposition – allowing only for the structural confirmation by ^1^H NMR and HRMS analysis. With the remaining material of product **4**, we attempted the final Grubbs‐catalyzed ring‐closing metathesis on a small scale at ambient temperature, but were only able to confirm the successful reaction by consistent HRMS data for macrolide **59**. Despite this, we were able to use a small amount of naturally isolated (+)‐neosorangicin (**1**) and subjected it to an excess of first cyclohexanone/PPTS/CuSO_4_ followed by TIPSOTf/pyr to synthesize the similarly protected intermediate **59** proving the utility of the used reaction conditions. The formation of the compound was confirmed by TLC and HRMS; however, due to its instability, NMR characterization was not possible, and the material was used without further purification. The obtained material of **59** was then treated with HF•pyr and stirred for two days, which resulted in global deprotection and formation of (+)‐neosorangicin A (**1**) as indicated by TLC and HRMS. This result shows that the initially planned synthetic route and the global deprotection of all protecting groups are achievable and provides access to the natural product via a relay approach.

## 3 Conclusion

In summary, a selective and convergent synthetic route to the three key fragments of the macrolide antibiotic (+)‐neosorangicin A (**1**) has been developed. Starting from commercially available or known building blocks, the fragments were prepared in 16–18 linear steps. The tetrahydropyran (**6**) and dioxabicyclo[3.2.1]octane (**5**) fragments were synthesized using established stereoselective transformations, while the DHP fragment **7** was accessible from L‐galactose (**19**) and transformed into **7** by an Ando olefination after a 1,2‐addition/dehydration at a methyl ketone. Assembly of the THP–BCO fragment **51** via Grubbs metathesis enabled a late‐stage Stille coupling between *E*‐vinyl iodide **58** and the DHP fragment **7**, which was accomplished on a small scale and confirmed by HRMS. A relay approach was employed to probe the final stages of the synthesis. Treatment of a small amount of isolated natural (+)‐neosorangicin A (**1**) with an excess of cyclohexanone and TIPSOTf, followed by global deprotection, led to regeneration of the natural product, as confirmed by TLC and HRMS, supporting the feasibility of the proposed endgame sequence. However, given the reported instability of (+)‐neosorangicin A (**1**), this transformation continues to represent a significant synthetic challenge.

## Conflicts of Interest

The authors declare no conflicts of interest.

## Supporting information




**Supporting File 1**: The supporting information includes experimental procedures and analytical data.
