# Supplementary material for: Relay Approach: A Convergent Synthesis of Key Fragments en route to (+)‐Neosorangicin A
Source: Chemistry. 2026 Jul 2;32(32):e71360. doi: 10.1002/chem.71360 (PMC13505654; doi:10.1002/chem.71360)

Supporting Information

Relay Approach: A Convergent Synthesis of Key Fragments En Route to (+)-Neosorangicin A

Marvin Wenninger, Maxim Munt, Le Chang, Rositsa Stoykova, Roberto Ojcius, Oliver Spieß, and Dieter Schinzer

Otto von Guericke University Magdeburg, Germany, dieter.schinzer@ovgu.de

Table of Content

1. General S2

2. Experimental Procedures and NMR spectra S3

1. General

Solvents were dried by standard procedures and redistilled under nitrogen atmosphere prior to use or were purchased in an appropriate water free quality and used as obtained. All reactions were run under nitrogen and in oven-dried glassware unless otherwise stated. Commercially available compounds (abcr, Acros, Aldrich, Fluka, Merck, and TCI) were used without purification. For reactions that require heating, an oil bath was used. The products were purified by flash chromatography on Merck silica gel 60 (40-63 µm). POLYGRAM SIL G/UV254 prefabricated TLC plates with fluorescent indicator from Macherey-Nagel have been used for the analytical thin layer chromatography (TLC). The separated substances were detected by irradiation with UV light with a wavelength of 254 nm or staining with vanillin stain (6 g vanillin, 200 mL ethanol and 1 mL conc. H_2SO_4) or potassium permanganate stain (2 g $KMnO_4$, 10 g Na_2CO_3, 200 mL water) and subsequent warming with a heat gun. Electrospray ionization (ESI) and electron ionisation (EI) mass spectra were recorded on Finnigan MAT 95 and Waters Xevo G2-TOF spectrometers. ^1H and ^{13}C NMR spectra were recorded on Brucker AVIII 400 and Brucker AVI 600 spectrometers. Chemical shifts (δ) are reported in ppm from tetramethylsilane, referenced to the solvent resonance resulting from incomplete deuteration (^1H NMR = $CDCl_3$: δ 7.26, acetone-d_6: δ 2.05, D_2O: δ 4.79; ^{13}C NMR = $CDCl_3$: δ 77.16, acetone-d_6: δ 29.8). Data are reported as follows: chemical shift, multiplicity (s = singlet, d = doublet, t = triplet, br = broad, m = multiplet, and combinations of those), coupling constants *J* (Hz) and integration. Optical rotations were recorded on an Anton Paar MCP150 polarimeter. Infrared (IR) spectra were recorded on Brucker Vertex 70v. Bands are characterized as strong (s), medium (m), weak (w) or broad (br).

2. Experimental Procedures and NMR spectra

Aldehyde 20

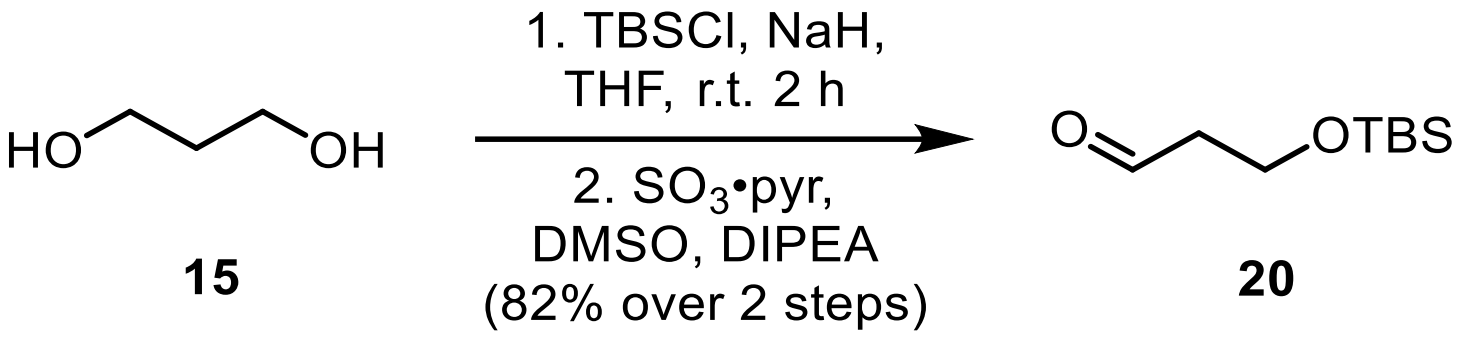

1,3-Propanediol (**15**) (8.0 g, 7.6 mL, 105.2 mmol, 1.0 eq.), dissolved in 50 mL dry THF was added dropwise to a suspension of NaH (60% in mineral oil, 4.2 g, 105.2 mmol, 1.0 eq.) in 70 mL dry THF. After stirring at r.t. for 1 h, TBSCl (15.9 g, 105.2 mmol, 1.0 eq.), dissolved in 40 mL dry THF, was added dropwise. Stirring was continued for 1 h, the reaction was quenched with 150 mL sat. $NaHCO_3$ solution and the mixture was extracted with Et_2O (3 x 50 mL). The combined organic phase was washed with sat. NH_4Cl solution, water and saturated NaCl solution, dried over $MgSO_4$ and concentrated in vacuo, affording 18.9 g (96% crude yield) of a slightly yellow oil, which was used without further purification in the next step. The crude material (18.9 g, 99.3 mmol, 1.0 eq.) was dissolved in 50 mL dry CH_2Cl_2 and cooled to 0 °C. DIPEA (50.0 mL, 287 mmol, 2.9 eq.) and DMSO (40.0 mL, 563 mmol, 5.7 eq.) were added, followed by portionwise addition of SO_3•pyridine (31.6 g, 198.6 mmol, 2.0 eq.). The resulting mixture was stirred for 2 h at 0 °C, before quenching with sat. NH_4Cl solution (100 mL) and diluting with CH_2Cl_2 (100 mL). The layers were separated and the organic layer was washed with water and sat. NaCl solution. The combined organic layer was dried over $MgSO_4$, filtered and concentrated under reduced pressure and subsequently purified by flash chromatography on silica (pentane/Et_2O, 10:1), yielding aldehyde **20** (16.2 g, 86.0 mmol, 82% over 2 steps) as a colorless, aromatic oil.

$\mathbf{R_f}$ = 0.59 (pentane/Et_2O, 10:1), $KMnO_4$, UV (-).

^{1}**H NMR** (400 MHz, $CDCl_3$) δ [ppm] = 9.77 (t, *J* = 2.0 Hz, 1H), 3.96 (t, *J* = 6.0 Hz, 2H), 2.57 (td, *J* = 6.7, 3.8 Hz, 2H), 0.86 (s, 9H), 0.04 (s, 6H).

^{13}**C NMR** (101 MHz, $CDCl_3$) δ [ppm] = 202.1, 57.5, 46.6, 25.9, -5.4.

Alcohol 21

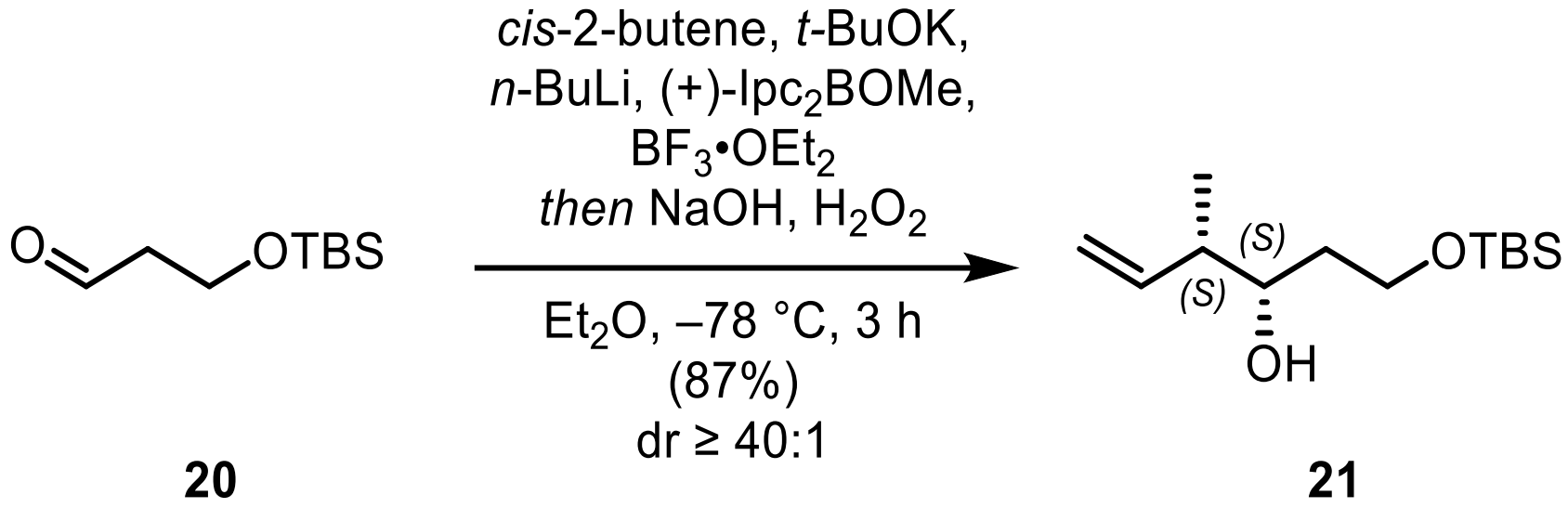

A three-necked flask was cooled to –78 °C, 10 mL dry THF were added and *cis*-2-butene (9.10 g, 160.5 mmol, 5.2 eq.) was condensed (tare weighing using an analytical balance). Then, *t*-BuOK (1M in THF, 40.0 mL, 40.0 mmol, 1.3 eq.) was added at –60 °C and the mixture was stirred for 10 min before slowly adding *n*-BuLi (2.5M in hexane, 15.3 mL, 38.2 mmol, 1.2 eq.). The solution was warmed up to –40 °C, stirred at this temperature for 20 min, before cooling again to –78 °C. Next, (+)-Ipc_2BOMe (13.95 g, 44.1 mmol, 1.4 eq.), dissolved in 20 mL dry THF, was added dropwise. Immediately thereafter, BF_3•Et_2O (7.0 mL, 55.2 mmol, 1.8 eq.) was added dropwise, and the mixture was stirred at –78 °C for 15 min. Subsequently, aldehyde **20** (5.81 g, 30.9 mmol, 1.0 eq.), dissolved in 20 mL dry THF, was added dropwise over 15 min and the resulting mixture was stirred at –78 °C for 3 h. The mixture was then warmed to –5 °C, followed by slow addition of 36.0 mL of 3M NaOH solution (exothermic!) and 12.5 mL H_2O_2 solution (30 wt%) (exothermic!), while vigorously stirring and keeping the interal temperature below +5 °C. The mixture was stirred at r.t. overnight, diluted with 150 mL water and the layers were separated. The organic layer was washed with water (2 x 100 mL), and the combined aqueous phase was extracted with Et_2O (3 x 100 mL). The combined organic phase was washed with sat. NaCl solution (100 mL), dried over $MgSO_4$, filtered and concentrated under reduced pressure. The crude material was purified by flash chromatography (pentane/Et_2O, 9:1), to furnish homoallylic alcohol **21** (6.54 g, 26.8 mol, 87%, dr ≥ 40:1) as a colorless oil.
Note: The starting material and product have almost the same R_f value, therefore traces of unreacted aldehyde are still found in the columned fractions. These impurities are mostly below 5%, but the yields were re-calculated based on the NMR data.
R_f = 0.39 (pentane/Et_2O, 9:1), vanillin: violet, UV (-).

^{1}H NMR (400 MHz, $CDCl_3$) δ [ppm] = 5.78 (ddd, *J* = 17.2, 10.4, 7.7 Hz, 1H), 5.10 – 4.99 (m, 2H), 3.90 (dt, *J* = 9.7, 4.7 Hz, 1H), 3.80 (ddd, *J* = 10.1, 8.8, 3.9 Hz, 1H), 3.67 (ddd, *J* = 9.0, 6.2, 2.3 Hz, 1H), 3.36 (s, 1H), 2.33 – 2.19 (m, 1H), 1.74 – 1.54 (m, 2H), 1.06 (d, *J* = 6.8 Hz, 3H), 0.90 (s, 9H), 0.07 (s, 6H).

^{13}C NMR (151 MHz, $CDCl_3$) δ [ppm] = 141.3, 114.9, 75.6, 63.2, 44.1, 35.6, 26.0, 18.3, 15.4, -5.4, -5.4.

IR (ATR) $\tilde{\nu}$ (cm^{-1}) = 3455 (b), 3079 (w), 2955 (m), 2929 (m), 2858 (m), 1640 (w), 1470 (m), 1415 (m), 1388 (m), 1362 (m), 1254 (s), 1080 (s), 1005 (m), 911 (s), 833 (s), 775 (s), 729 (m), 663 (m).

HRMS (ESI) (*m/z*) calcd. for $[C_{13}H_{28}O_2SiH]^+$ = $[M+H]^+$: 245.1937, found: 245.1930.

Specific Rotation $[\alpha]_{589}^{24}$= –6.4° (c = 1.1, $CHCl_3$).

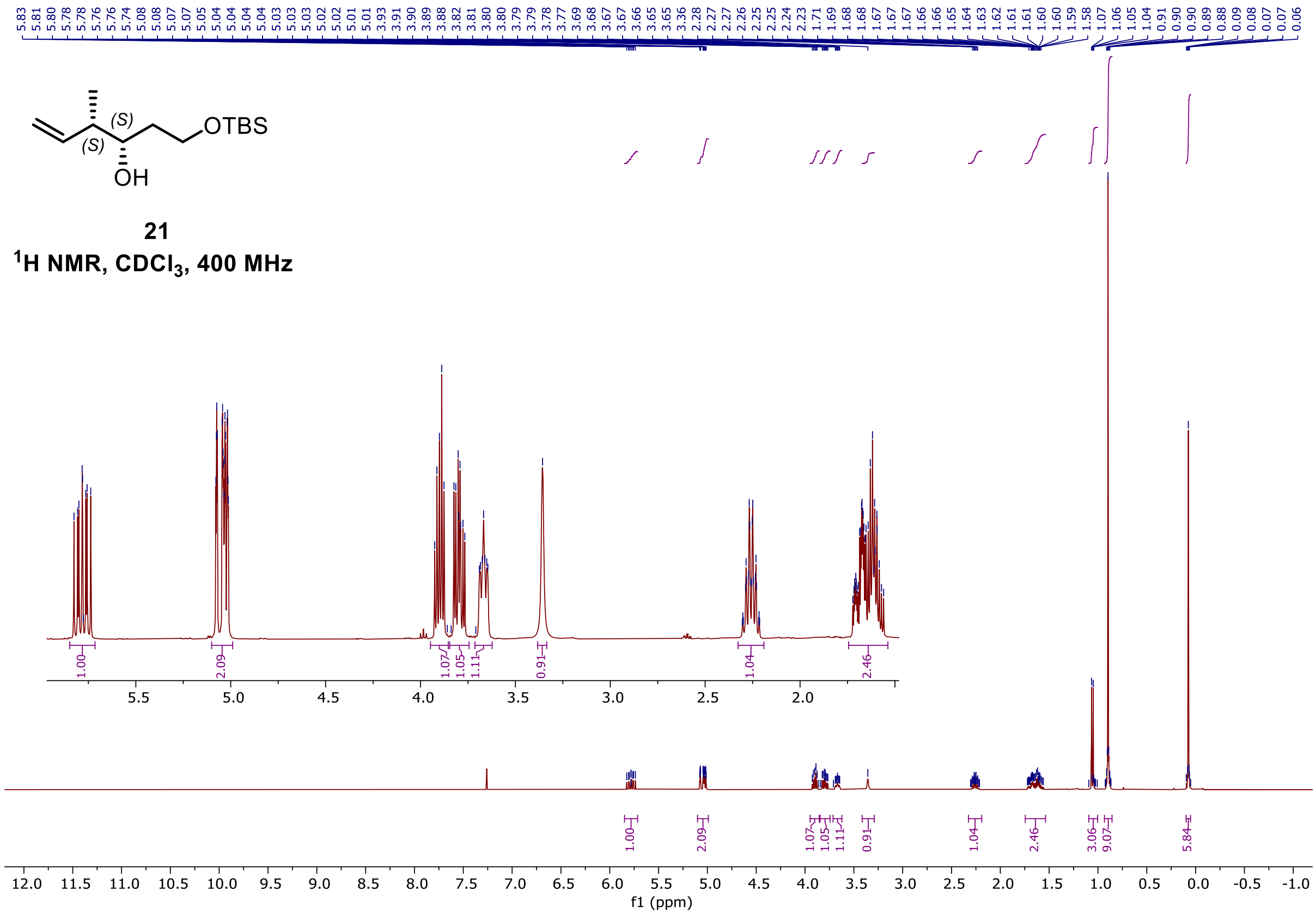

21
^{1}H NMR, $CDCl_3$, 400 MHz

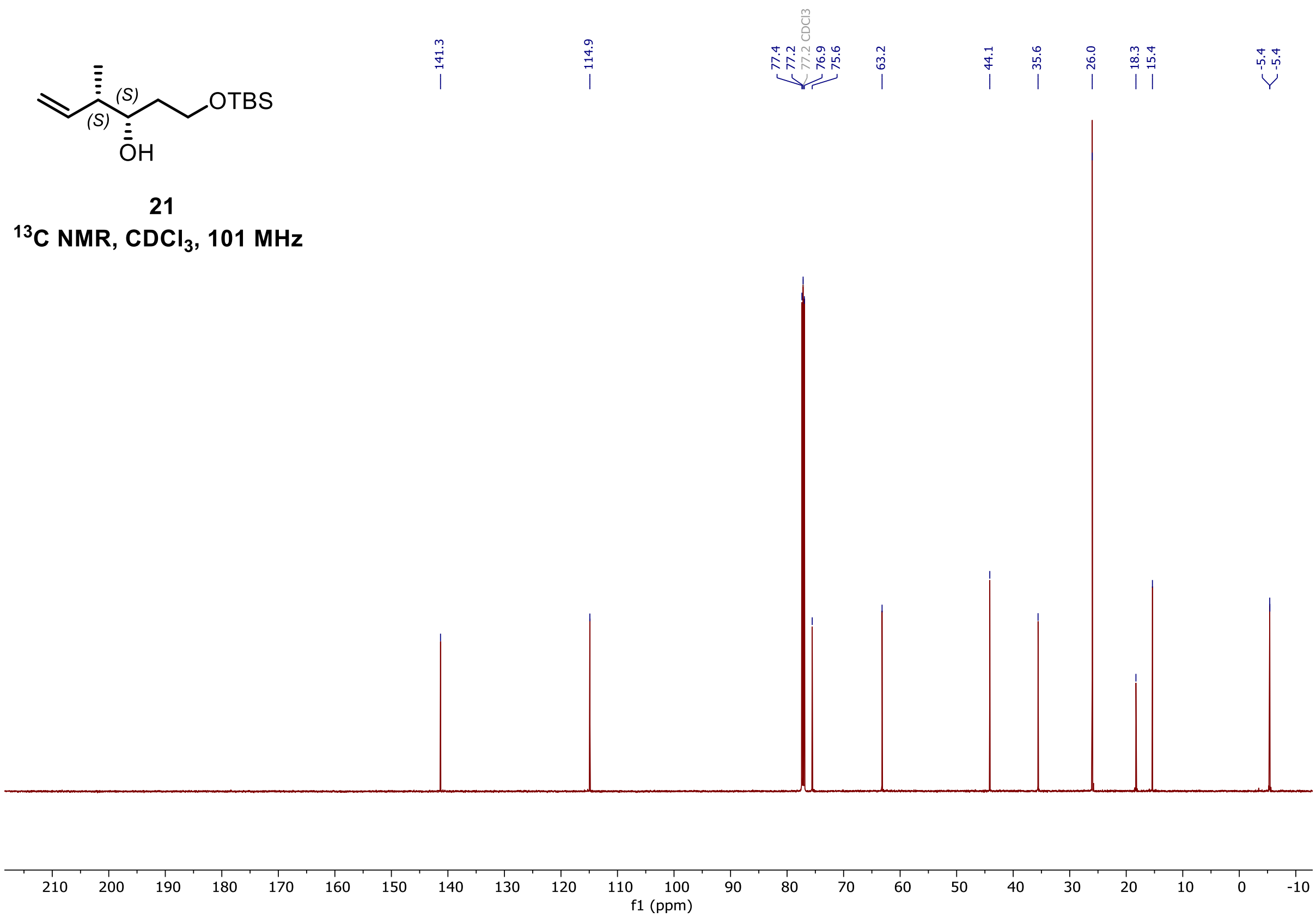

21
^{13}C NMR, $CDCl_3$, 101 MHz

Triol 14

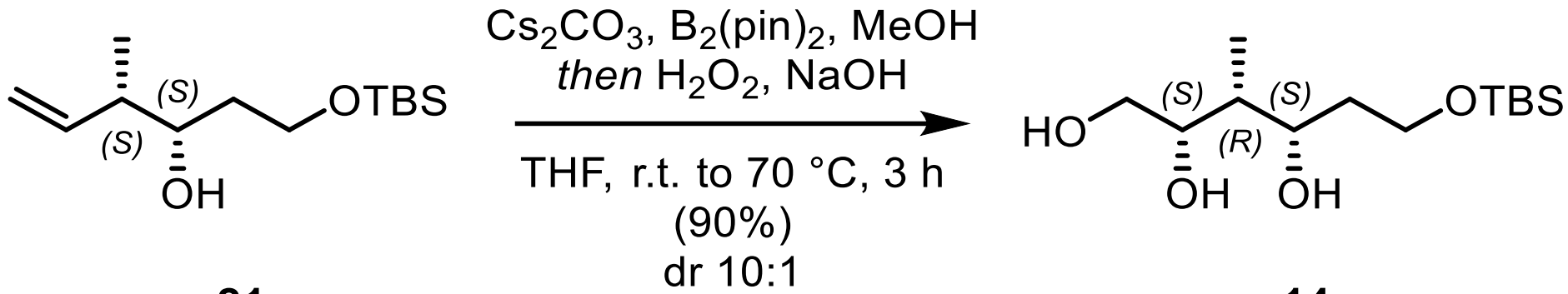

21 **14**

A 1 L three-necked flask was charged with 250 mL dry THF, before addition of Cs_2CO_3 (4.7 g, 14.5 mmol, 0.35 eq.) and bis(pinacolato)diboron (13.1 g, 51.2 mmol, 1.25 eq.) and the mixture was stirred at r.t. for 10 min. Homoallyl alcohol **21** (10.1 g, 41.3 mmol, 1.0 eq.) and dry MeOH (28.3 mL, 700 mmol, 17.0 eq.) were added and the reaction mixture was stirred at 70 °C for 3 h. After completion of the reaction, the mixture was cooled to 0 °C. Then, 3M NaOH (25 mL) and H_2O_2 (35 wt%, 20 mL) were slowly added dropwise, keeping the inner temperature below +5 °C. The reaction mixture was allowed to warm up to room temperature overnight. It was then cooled again to 0 °C and sat. $Na_2S_2O_3$ (50 mL) solution was slowly added. After warming up to r.t. and diluting with sat. NH_4Cl solution (100 mL), the aqueous phase was extracted with EtOAc (3 x 100 mL). The combined organic layer was washed with sat. NaCl solution, dried over Na_2SO_4 and concentrated under reduced pressure and stored in the fridge overnight. The by-product (pinacol) crystallized out and was filtered off, allowing to use the crude product without any further purification for the next step. For analytical reasons, flash chromatography on silica (pentane/EtOAc, 1:1 to 100% EtOAc) was performed, affording triol **14** (10.3 g, 37.0 mmol, 90%, dr 10:1) as a highly viscous, colorless liquid.

$\mathbf{R_f}$ = 0.48 (EtOAc), vanillin: violet, UV (-).

^{1}H NMR (400 MHz, $CDCl_3$) δ [ppm] = 4.16 (dt, *J* = 10.2, 2.0 Hz, 1H), 3.93 (ddd, *J* = 10.0, 4.5, 3.3 Hz, 1H), 3.88 – 3.71 (m, 3H), 3.54 (dd, *J* = 11.2, 4.1 Hz, 1H), 1.90 (dtd, *J* = 14.8, 10.5, 4.5 Hz, 1H), 1.72 – 1.61 (m, 1H), 1.45 (dtd, *J* = 14.4, 3.0, 1.8 Hz, 1H), 0.95 (d, *J* = 7.1 Hz, 3H), 0.90 (s, 9H), 0.09 (d, *J* = 1.5 Hz, 6H).

^{13}C NMR (101 MHz, $CDCl_3$) δ [ppm] = 75.2, 74.7, 64.0, 63.5, 41.1, 35.9, 26.0, 18.2, 7.9, -5.4, -5.5.

IR (ATR) ṽ (cm^{-1}) = 3372 (b), 2929 (m), 2857 (m), 1463 (s), 1253 (m), 1085 (w), 833 (s), 774 (w).

HRMS (ESI) (*m/z*) calcd. for $[C_{13}H_{30}O_4SiH]^+$ = $[M+H]^+$: 279.1997, found: 279.1989.

Specific Rotation $[\alpha]_{589}^{24}$= +7.2° (c = 1.2, $CHCl_3$).

14

^{1}H NMR, CDCl$_3$, 400 MHz

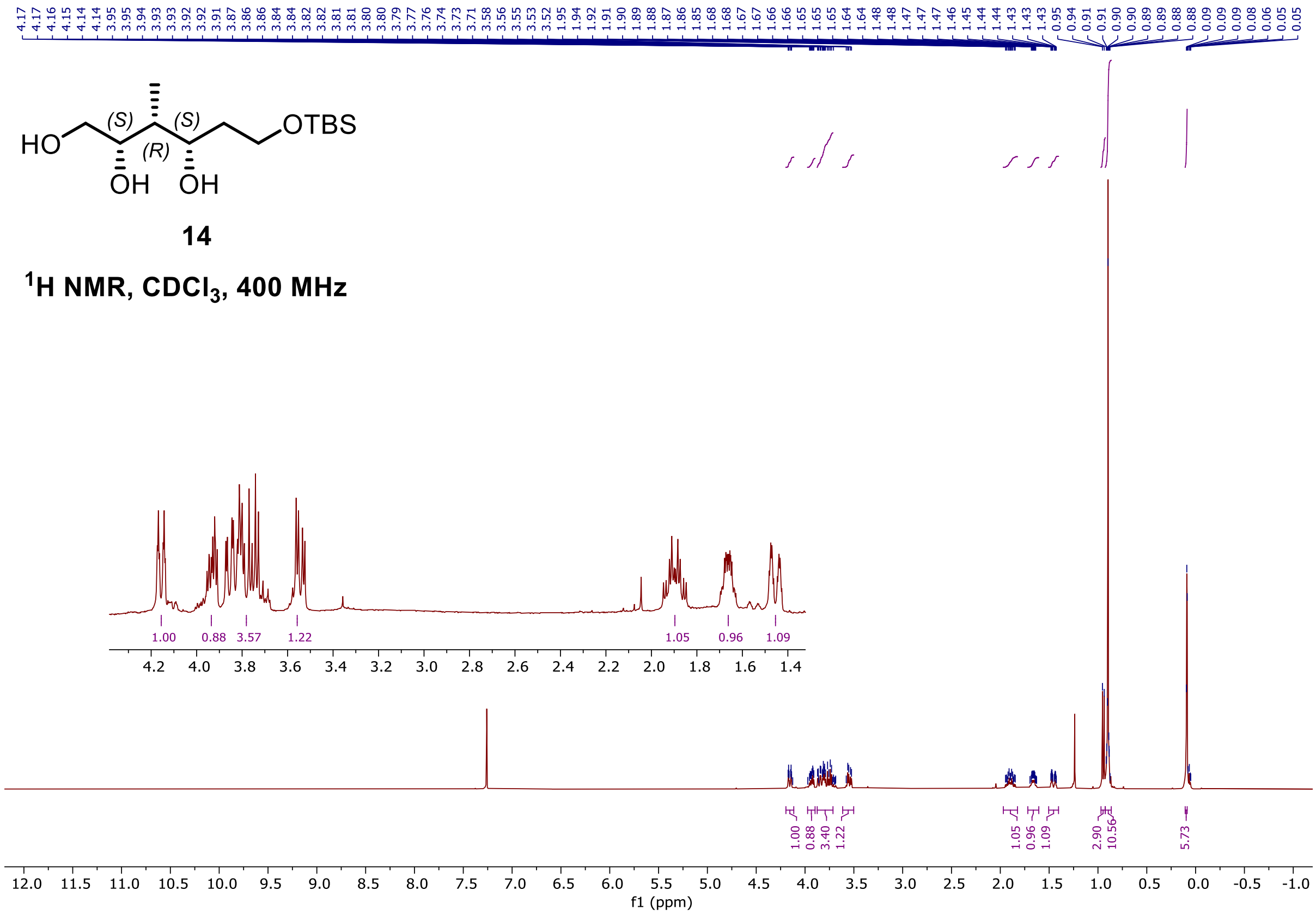

14

^{13}C NMR, CDCl$_3$, 101 MHz

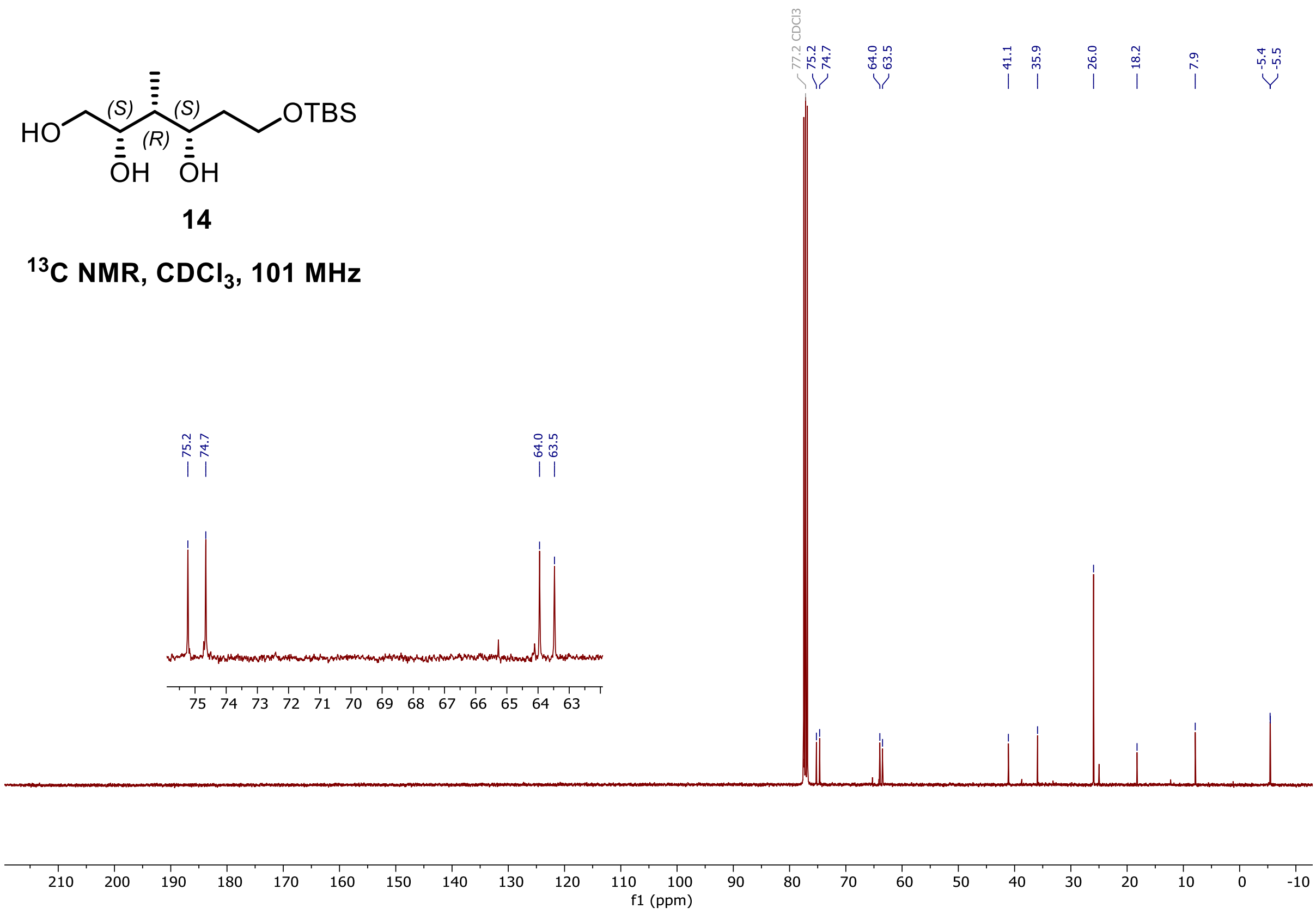

Diol S14a

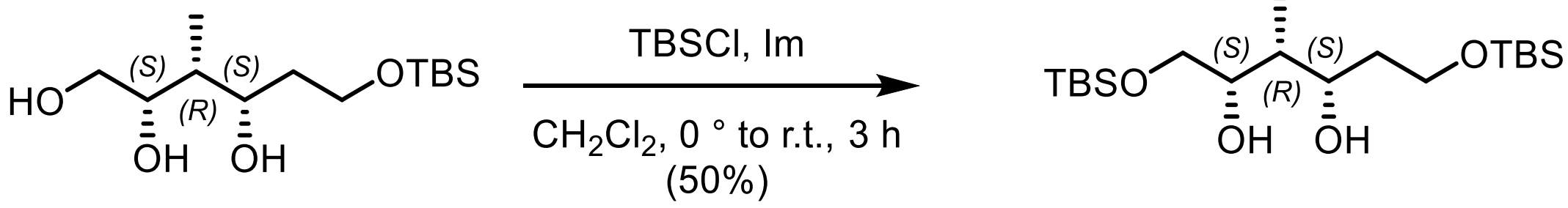

14 **S14a**

A solution of triol **14** (96 mg, 0.35 mmol, 1.0 eq.) and imidazole (32 mg, 0.47 mmol, 1.3 eq.) in 5 mL dry CH_2Cl_2 was cooled to 0 °C. TBSCl (64 mg, 0.42 mmol, 1.2 eq,), dissolved in 2 mL dry CH_2Cl_2, was slowly added and the mixture was stirred at r.t. for 3 h. After quenching with sat. $NaHCO_3$ and extraction with CH_2Cl_2, the combined organic phases were washed with water and sat. NaCl. After drying over $MgSO_4$ and evaporation of the solvent, the residue was purified by flash chromatography on silica (pentane/diethyl ether, 2:1) to afford diol **S14a** (67 mg, 0.17 mmol, 50%) as a colorless oil.

R_f = 0.7 (pentane/diethyl ether 1:1), vanillin: blue, UV (-).

^{1}H NMR (400 MHz, $CDCl_3$) δ [ppm] = 4.11 (q, *J* = 10.7 Hz, 1H), 4.03 (dt, *J* = 9.5, 3.4 Hz, 1H), 3.92 – 3.75 (m, 2H), 3.59 (dd, *J* = 6.3, 2.5 Hz, 1H), 3.47 (q, *J* = 7.0 Hz, 1H), 1.89 – 1.76 (m, 1H), 1.68 – 1.58 (m, 1H), 1.58 – 1.48 (m, 1H), 0.93 (d, *J* = 7.3 Hz, 3H), 0.89 (m, 18H), 0.89 (m, 12H).

Acetonide S14b

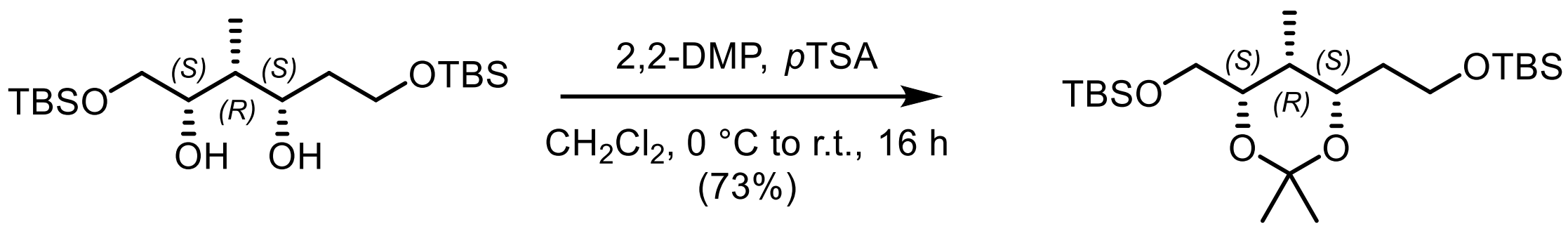

S14a **S14b**

To a 0 °C cooled solution diol **S14a** (67 mg, 0.17 mmol, 1.0 eq.) and 2,2-DMP (320 µL, 2.57 mmol, 15.1 eq,) in 5 mL CH_2Cl_2, was added *p*TSA•H_2O (4.3 mg, 0.017 mmol, 10 mol%). After stirring for 16 h at room temperature, Et_3N (100 µL) was added and the resulting mixture was directly subjected to column chromatography on silica (pentane/diethyl ether, 30:1) to yield acetonide **S14b** (54 mg, 0.13 mmol, 73%) as acolorless oil.

R_f = 0.4 (pentane/diethyl ether, 30:1), vanillin: blue, UV (-).

^{1}H NMR (600 MHz, $CDCl_3$) δ [ppm] = 4.05 (ddd, *J* = 8.8, 4.2, 2.1 Hz, 1H), 3.95 (td, *J* = 6.8, 2.5 Hz, 1H), 3.65 – 3.57 (m, 2H), 3.55 (dd, *J* = 9.6, 6.3 Hz, 1H), 3.47 (dd, *J* = 9.6, 6.3 Hz, 1H), 1.69 – 1.62 (m, 1H), 1.54 – 1.45 (m, 1H), 1.37 (s, 3H), 1.31 (s, 3H), 1.17 (m, 1H), 0.83 (s, 9H), 0.82 (s, 9H), 0.78 (d, *J* = 6.7 Hz, 3H), 0.00 (s, 6H), -0.01 (s, 6H).

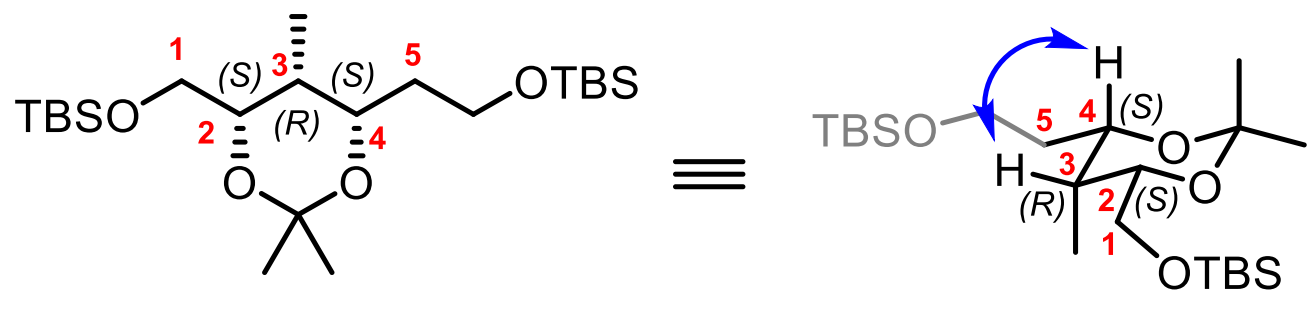

S14b $\equiv$ $^{3}J^{\text{ H3-H4}}$ = 2.2 Hz

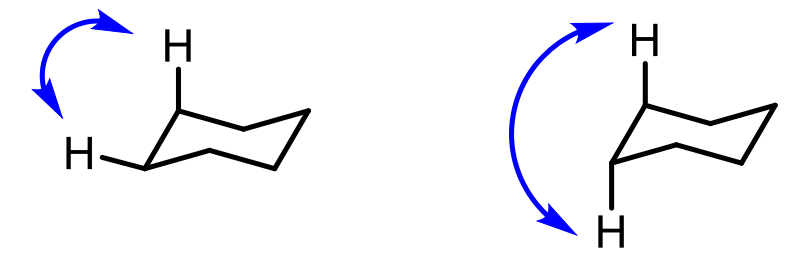

$^{3}J^{\text{ ax-eq}}$ = 2–4 Hz $^{3}J^{\text{ ax-ax}}$ = 6.5–15 Hz

Range for coupling constants taken from: https://organicchemistrydata.org/

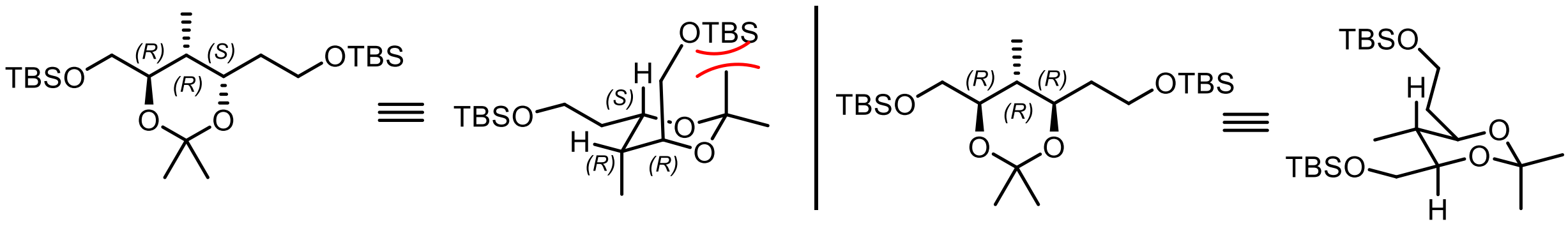

Note: Since both, Brown-crotylation (here: *syn*-selective) and Morken-dihydroxylation (*syn*-selective), are selective reactions due to their corresponding borane-intermediate, the configuration of **S14b** was confirmed by the $^{3}J^{\text{ H3-H4}}$ = 2.2 Hz coupling constant. If the crotylation would have yielded the *syn*-product, a following *anti*-dihydroxylation would result in the (*R*),(*R*),(*S*)-configuration with H2-H3 being axial-equatorial oriented, but would result in a sterically hindered chair conformation (bottom, left). In the case of first the *anti*-crotylated molecule, the subsequent *syn*-selective Morken-dihydroxylation would result in an axial-axial coupling between H3-H4 and therefore be in the range of 6.5–15 Hz (bottom, right). This confirms the structure of **S14b** and the (*S*),(*R*),(*S*)-configuration.

Acetonide 22

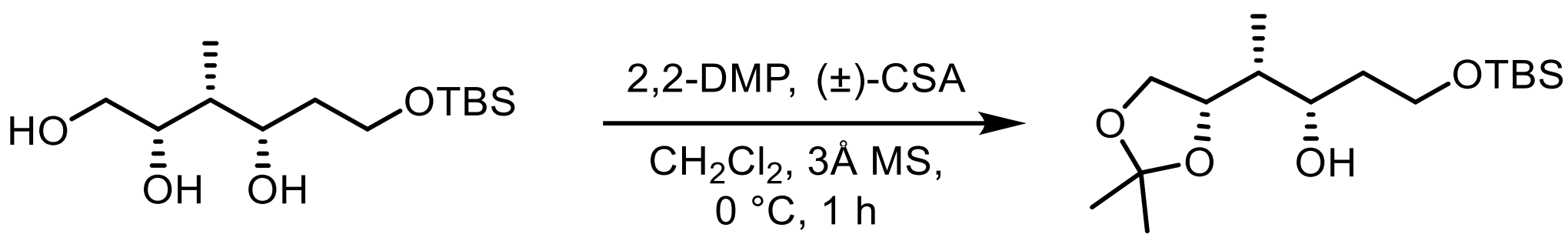

14 **22**

Triol **14** (2.42 g, 8.7 mmol, 1.0 eq.) and 2 g powdered 3Å molecular sieve were suspended in 50 mL dry CH_2Cl_2. The mixture was cooled to 0 °C. and 2,2-dimethoxypropane (1.6 mL, 13.0 mmol, 1.5 eq.) and (±)-CSA (100 mg, 0.43 mmol, 5 mol%) were added successively. The ice bath was removed and the reaction mixture was stirred for 1 h at r.t. The reaction was carefully quenched with sat. $NaHCO_3$ solution (60 mL) and the mixture was filtered through a silica pad. The layers were separated and the aqueous phase was extracted with CH_2Cl_2 (2 x 75 mL). The combined organic layer was washed with NaCl solution (100 mL), dried over $MgSO_4$ and concentrated in vacuo. The crude material was purified by flash chromatography (pentane/ EtOAc, 7: 1), affording acetonide **22** (2.19 g, 6.9 mmol, 79%) as a clear oil.

R_f = 0.64 (pentane/EtOAc, 7:1), vanillin: blue, UV (-).

^{1}H NMR (400 MHz, $CDCl_3$) δ [ppm] = 4.18 (dt, *J* = 7.5, 6.0 Hz, 1H), 4.07 (dd, *J* = 8.1, 6.2 Hz, 1H), 3.93 – 3.84 (m, 2H), 3.79 (ddd, *J* = 10.1, 9.2, 3.8 Hz, 1H), 3.71 (t, *J* = 7.8 Hz, 1H), 3.38 (br s, 1H), 1.77 (dtd, *J* = 14.2, 9.4, 4.7 Hz, 1H), 1.71 – 1.61 (m, 1H), 1.61 – 1.50 (m, 1H), 1.40 (s, 3H), 1.35 (s, 3H), 1.00 (d, *J* = 6.9 Hz, 3H), 0.89 (s, 9H), 0.07 (s, 6H).

^{13}C NMR (101 MHz, $CDCl_3$) δ [ppm] = 108.7, 78.2, 73.7, 68.0, 63.0, 41.3, 36.0, 26.7, 26.0, 25.7, 18.3, 9.4, -5.3, -5.4.

IR (ATR) ṽ (cm^{-1}) = 3446 (b), 2930 (m), 2858 (m), 1463 (w), 1369 (w), 1252 (m), 1215 (m), 1087 (s), 1057 (s), 833 (s), 775 (s), 663 (w), 511 (w).

HRMS (ESI) (*m/z*) calcd. for $[C_{16}H_{34}O_4SiH]^+$ = $[M+H]^+$: 319.2305, found: 319.2301.

Specific Rotation $[\alpha]_{589}^{25}$= +1.74° (c = 1.15, $CHCl_3$).

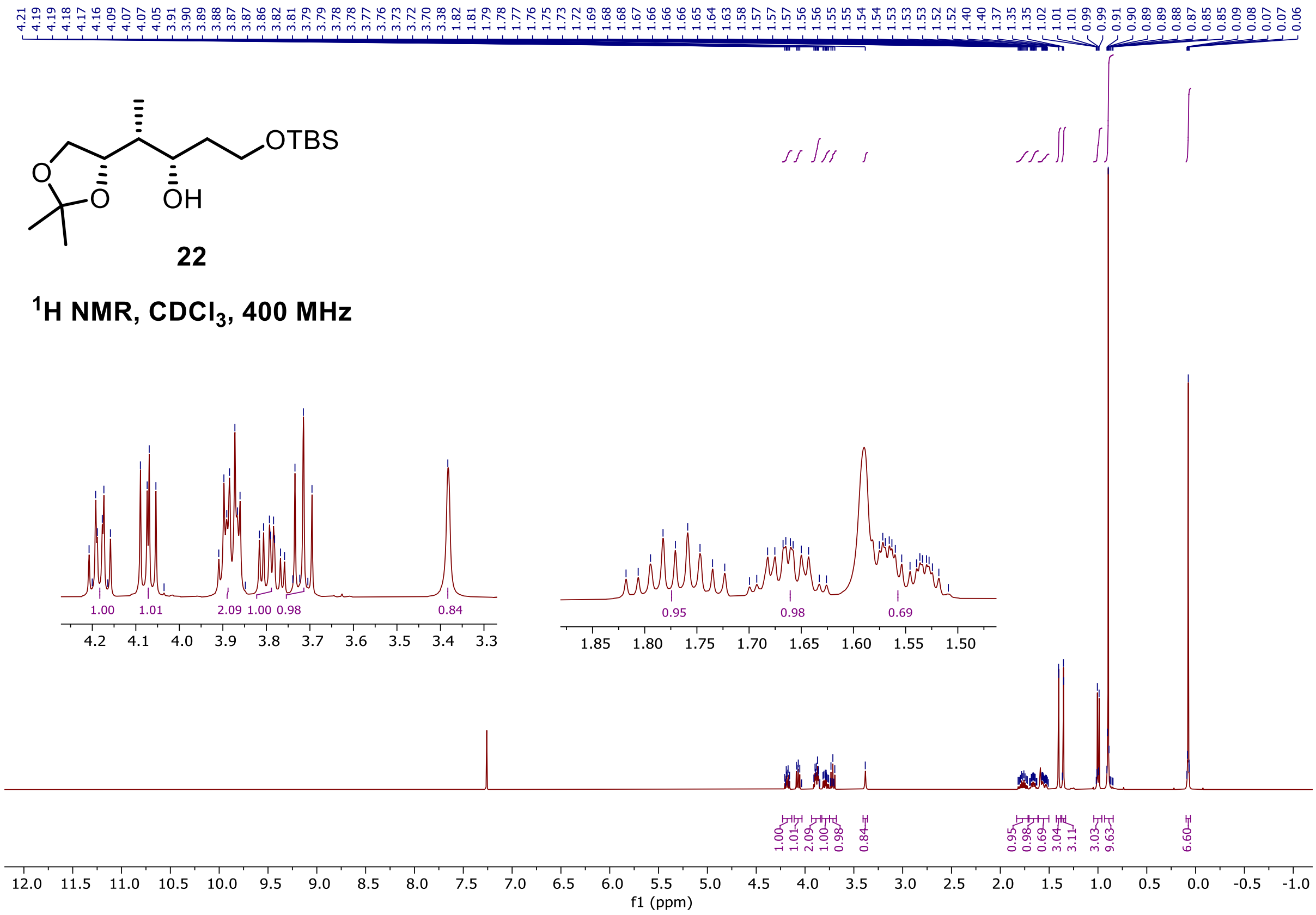

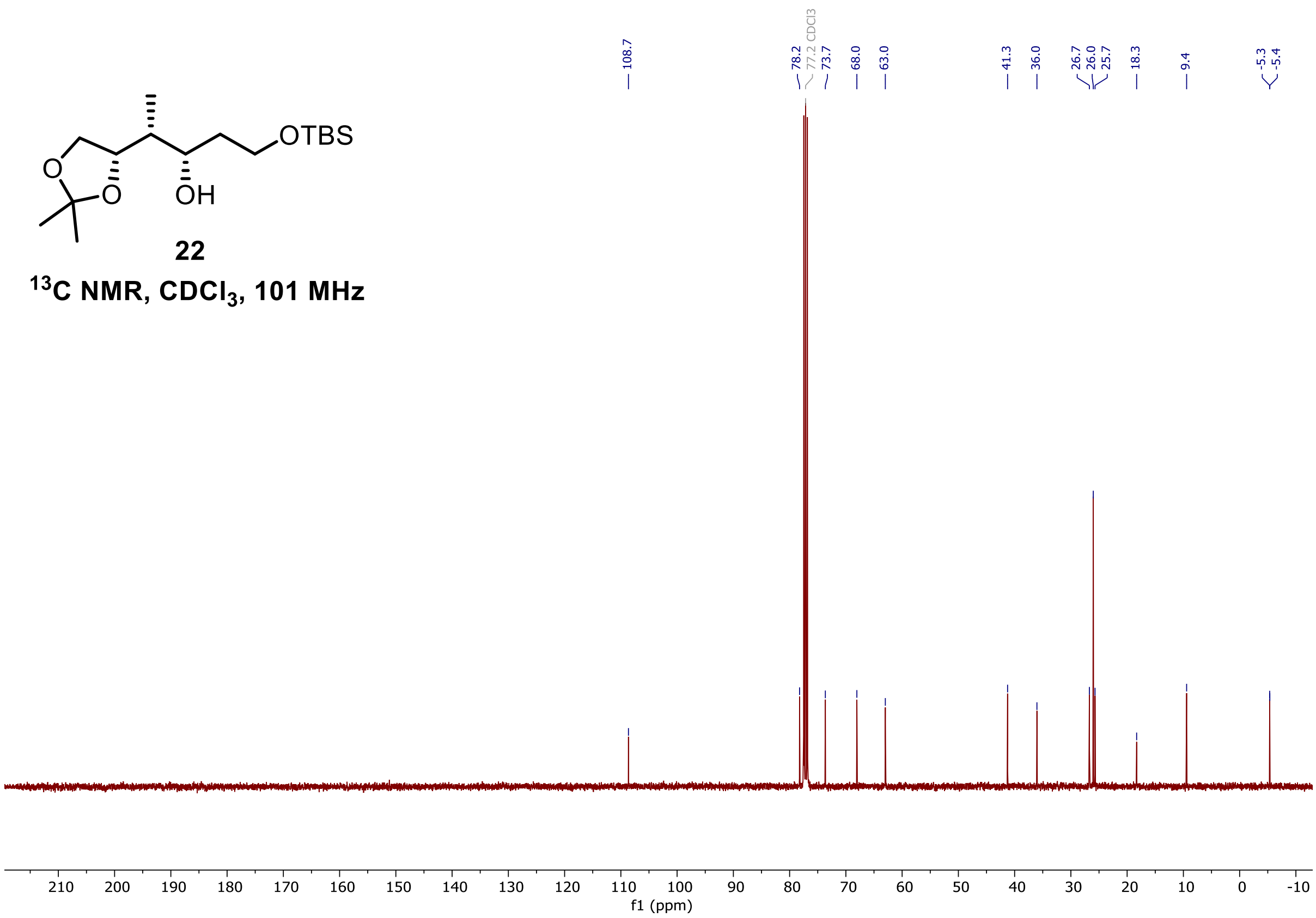

Acetonide 23a

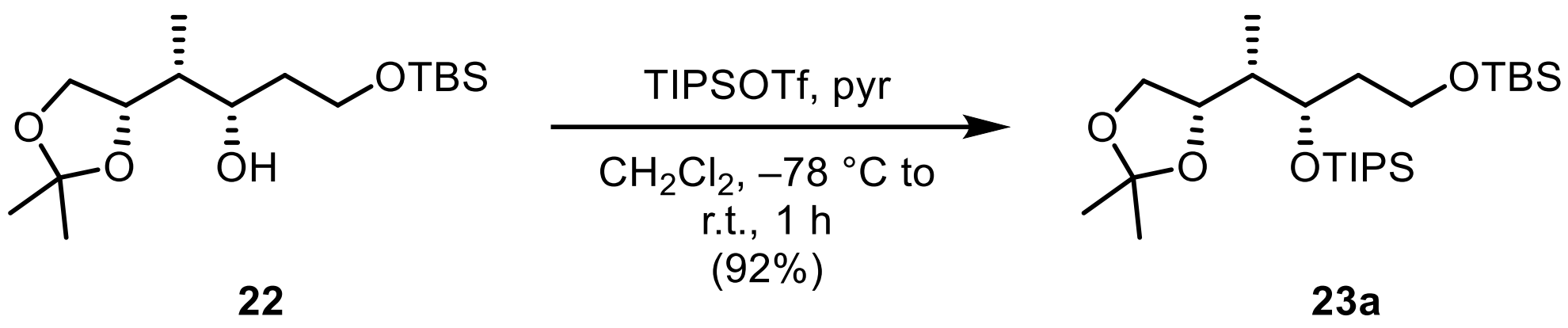

Alcohol **22** (8.5 g, 26.7 mmol, 1.0 eq.) and pyridine (6.5 mL, 80.0 mmol, 3.0 eq.) were dissolved in 200 mL dry CH_2Cl_2. The solution was cooled to –78 °C and TIPSOTf (14.0 mL, 53.4 mmol, 2.0 eq.) was added dropwise. The reaction mixture was slowly warmed to r.t. and stirred for 1 h. After completion of the reaction, it was quenched with sat. $NaHCO_3$ solution, the layers were separated, and the aqueous phase was extracted with CH_2Cl_2 (3 x 50 mL). The combined organic layer was washed with sat. NH_4Cl solution, sat. $NaHCO_3$ solution, sat. NaCl solution and dried over $MgSO_4$. After concentration, the crude product can be used directly for the next step. For analysis, it was purified by flash chromatography (pentane/Et_2O, 15:1), affording acetonide **23a** (11.7 g, 24.6 mmol, 92%) as a colorless oil.

R_f = 0.79 (pentan/Et_2O, 25:1), vanillin: blue, UV (-).

^{1}H NMR (600 MHz, $CDCl_3$) δ [ppm] = 4.16 (td, *J* = 7.6, 5.9 Hz, 1H), 4.04 (dd, *J* = 8.0, 5.9 Hz, 1H), 3.94 (td, *J* = 6.5, 2.3 Hz, 1H), 3.68 – 3.59 (m, 3H), 1.79 – 1.71 (m, 2H), 1.72 – 1.64 (m, 1H), 1.38 (s, 3H), 1.35 (s, 3H), 1.07 (s, 3H), 1.06 (s, 18H), 0.99 (d, *J* = 6.9 Hz, 3H), 0.87 (s, 9H), 0.03 (d, *J* = 1.6 Hz, 6H).

^{13}C NMR (151 MHz, $CDCl_3$) δ [ppm] = 108.4, 77.4, 71.4, 68.6, 59.9, 41.5, 37.0, 27.0, 26.0, 25.9, 18.4, 18.4, 13.1, 10.9, -5.3, -5.3.

IR (ATR) ṽ (cm^{-1}) = 2931 (m), 2866 (m), 1463 (w), 1368 (w), 1252 (m), 1093 (s), 1045 (s), 940 (w), 832 (s), 774 (s), 676 (s), 510 (w).

HRMS (ESI) (*m/z*) calcd. for $[C_{25}H_{54}O_4Si_2H]^+$ = $[M+H]^+$: 475.3639, found: 475.3630.

Specific Rotation $[\alpha]^{25}_{589}$= –2.9° (c = 1.05, $CHCl_3$).

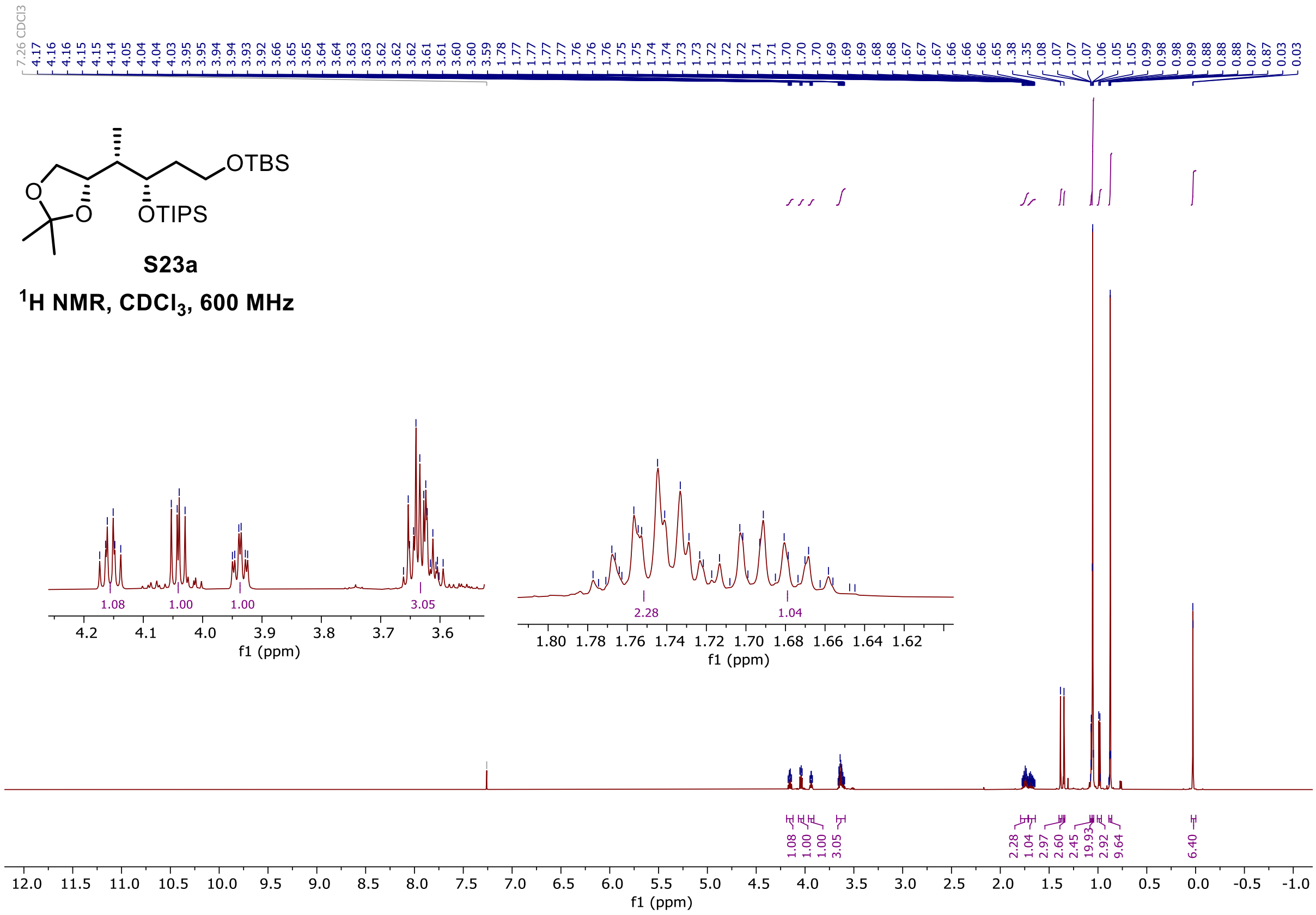

S23a

^{1}H NMR, $CDCl_3$, 600 MHz

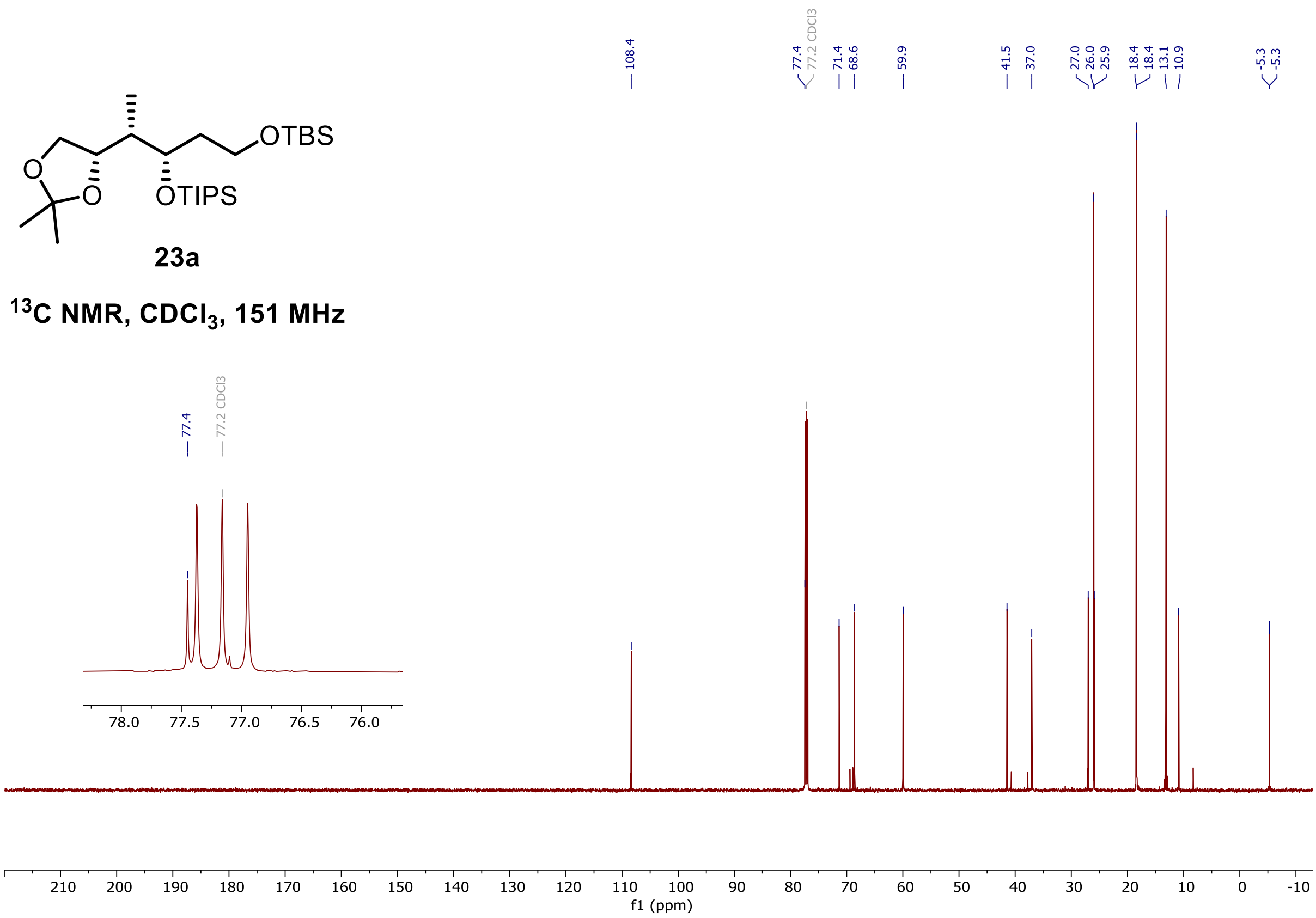

23a

^{13}C NMR, $CDCl_3$, 151 MHz

Alcohol 23

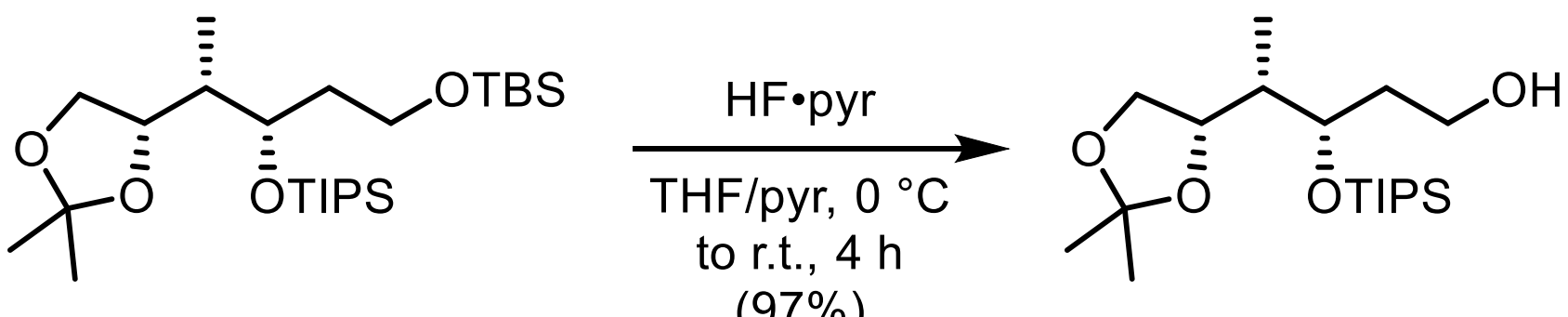

23a **23**

A polypropylene reaction vessel was charged with 200 mL dry THF, 30 mL pyridine and acetonide **23a** (11.7 g, 24.6 mmol, 1.0 eq.). The mixture was cooled to 0 °C and HF•pyridine (approx. 70% HF, 18.0 mL, 139.9 mmol, 5.7 eq.) was added dropwise. The reaction mixture was brought to r.t. and stirred for 4 h. Next, sat. NH_4Cl solution (200 mL) was added dropwise and the mixture was stirred for additional 15 min. The phases were separated and the aqueous phase was extracted with Et_2O (2 x 100 mL). The combined organic phase was washed with sat. $NaHCO_3$ solution, sat. NaCl solution and dried over $MgSO_4$. After concentration, the crude material was purified by flash chromatography (pentane/Et_2O, 3:2), yielding alcohol **23** as a colorless oil (8.66 g, 24.0 mmol, 97%).

R_f = 0.73 (pentane/Et_2O, 2:1), vanillin: blue, UV (-).

^{1}H NMR (600 MHz, $CDCl_3$) δ [ppm] = 4.24 (dt, *J* = 7.7, 5.9 Hz, 1H), 4.02 (dd, *J* = 8.0, 6.2 Hz, 1H), 3.95 (ddd, *J* = 7.0, 5.5, 3.0 Hz, 1H), 3.73 (td, *J* = 6.4, 1.4 Hz, 2H), 3.65 (t, *J* = 7.9 Hz, 1H), 1.88 – 1.72 (m, 4H), 1.39 (s, 3H), 1.35 (s, 3H), 1.07 (s, 21H), 0.98 (d, *J* = 7.0 Hz, 3H).

^{13}C NMR (151 MHz, $CDCl_3$) δ [ppm] = 108.6, 76.2, 73.0, 68.3, 60.4, 41.6, 36.3, 26.8, 25.8, 18.4, 13.1, 11.1.

IR (ATR) ṽ (cm^{-1}) = 3436 (m), 2944 (s), 2868 (s), 2724 (w), 1464 (m), 1380 (s), 1369 (s), 1216 (s), 1061 (s), 883 (s).

HRMS (ESI) (*m/z*) calcd. for $[C_{19}H_{40}O_4SiH]^+$ = $[M+H]^+$: 361.2774, found: 361.2773.

Specific Rotation $[\alpha]_{589}^{25}$ = –16.5° (c = 1.0, $CHCl_3$).

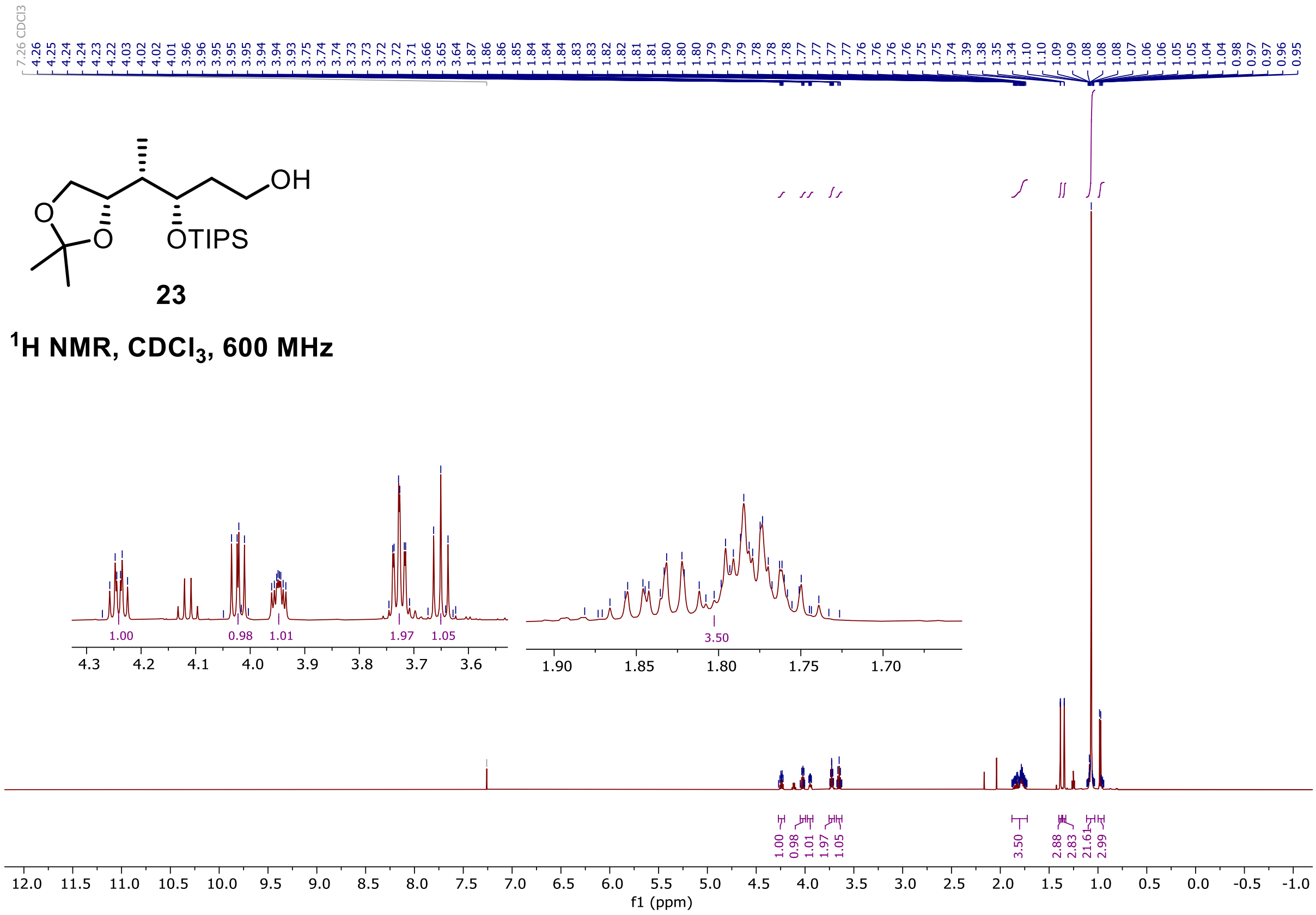

23

^{1}H NMR, $CDCl_3$, 600 MHz

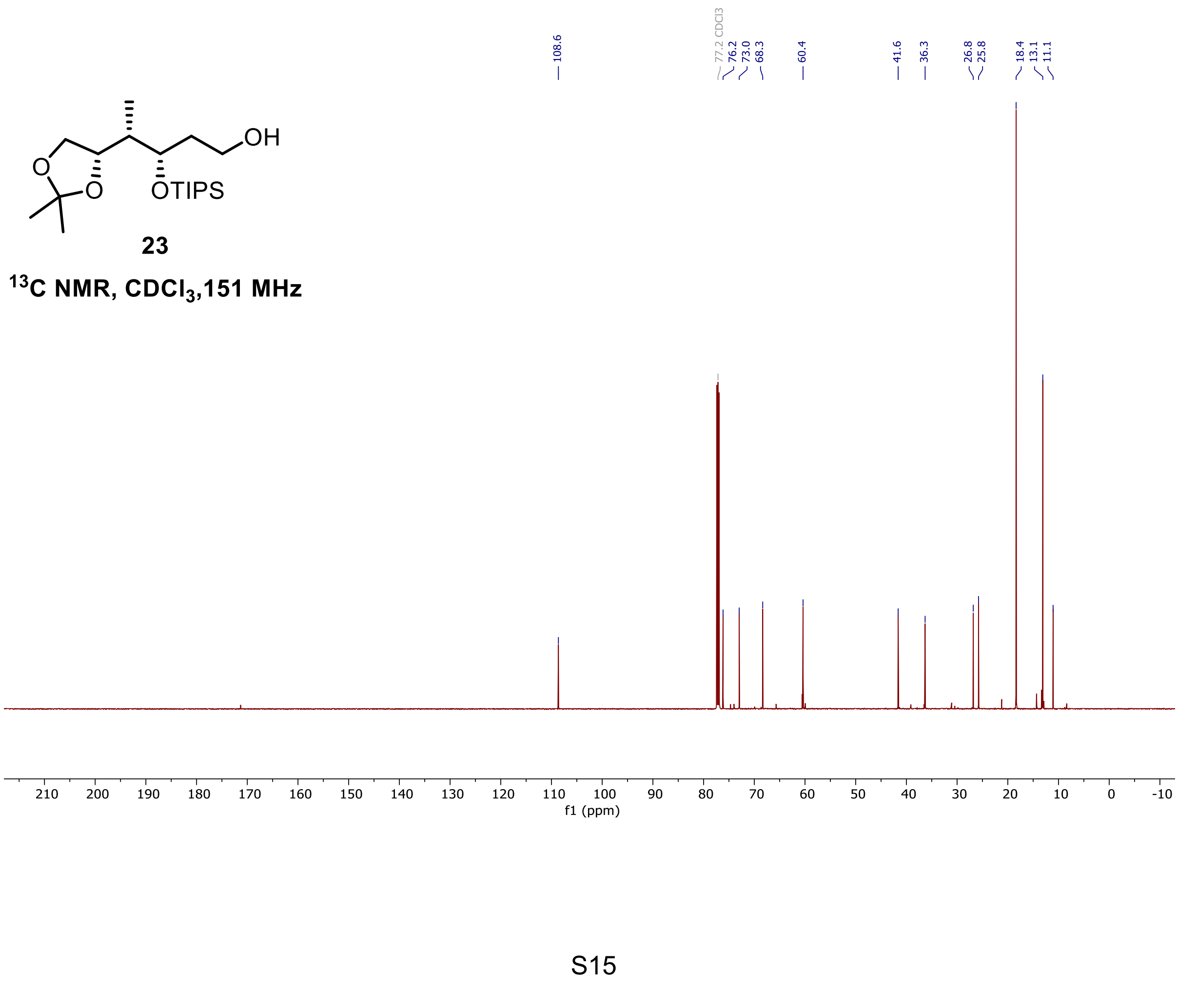

23

^{13}C NMR, $CDCl_3$,151 MHz

Aldehyde 24

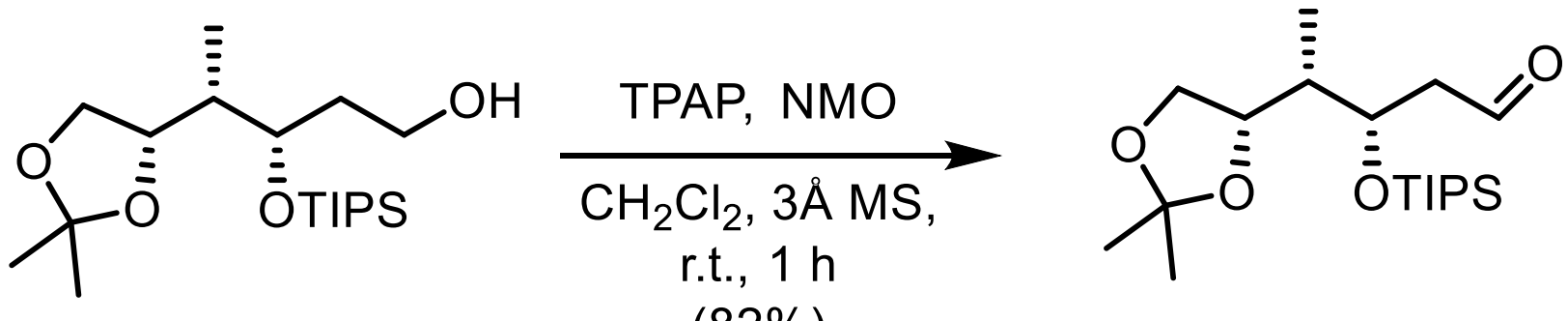

23 **24**

In a round-bottom flask, alcohol **23** (3.12 g, 8.7 mmol, 1.0 eq.), 4 g powdered 3Å molecular sieve and NMO monohydrate (2.0 g, 14.8 mmol, 1.7 eq.) were dissolved in 80 mL dry CH_2Cl_2 and stirred for 30 min at r.t. Then, TPAP (150 mg, 0.43 mmol, 5 mol%) was added and the black solution was stirred for 30 min at r.t. The reaction mixture was filtered through a silica pad and washed with 100 mL of CH_2Cl_2. The solvent was removed and the crude product was purified by flash chromatography (pentane/Et_2O, 7:1) providing aldehyde **24** (2.54 g, 7.1 mmol, 82%) as a colorless liquid.

Note: The aldehyde decomposes fast in solution within a few hours at r.t. Therefore, only the major peaks are noted and picked in the corresponding ^{13}C spectra.

R_f = 0.24 (pentane/Et_2O, 10:1), vanillin: blue, UV (-).

^{1}H NMR (600 MHz, $CDCl_3$) δ [ppm] = 9.83 (t, *J* = 2.0 Hz, 1H), 4.33 (ddt, *J* = 7.5, 6.3, 4.0 Hz, 2H), 4.02 (dd, *J* = 8.0, 6.4 Hz, 1H), 3.65 (t, *J* = 7.9 Hz, 1H), 2.72 – 2.61 (m, 2H), 1.83 (dqd, *J* = 10.4, 7.2, 3.7 Hz, 1H), 1.37 (s, 3H), 1.34 (s, 3H), 1.05 (s, 21H), 0.93 (d, *J* = 7.0 Hz, 3H).

^{13}C NMR (151 MHz, $CDCl_3$) δ [ppm] = 202.1, 108.9, 77.8, 74.7, 68.2, 48.0, 42.0, 25.7, 18.2, 12.1.

IR (ATR) ṽ (cm^{-1}) = 2944 (s), 2868 (s), 2724 (w), 1728 (m), 1659 (w), 1463 (s), 1381 (s), 1248 (m), 1093 (s), 882 (s).

HRMS (ESI) (*m/z*) calcd. for $[C_{19}H_{38}O_4SiH]^+$ = $[M+H]^+$: 373.2774, found: 373.2783.

Specific Rotation $[\alpha]_{546}^{20}$= –9.8° (c = 1.1, $CHCl_3$).

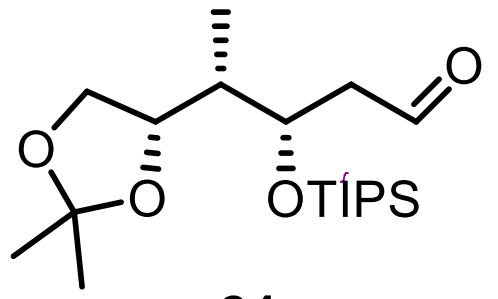

24

^{1}H NMR, CDCl$_3$, 600 MHz

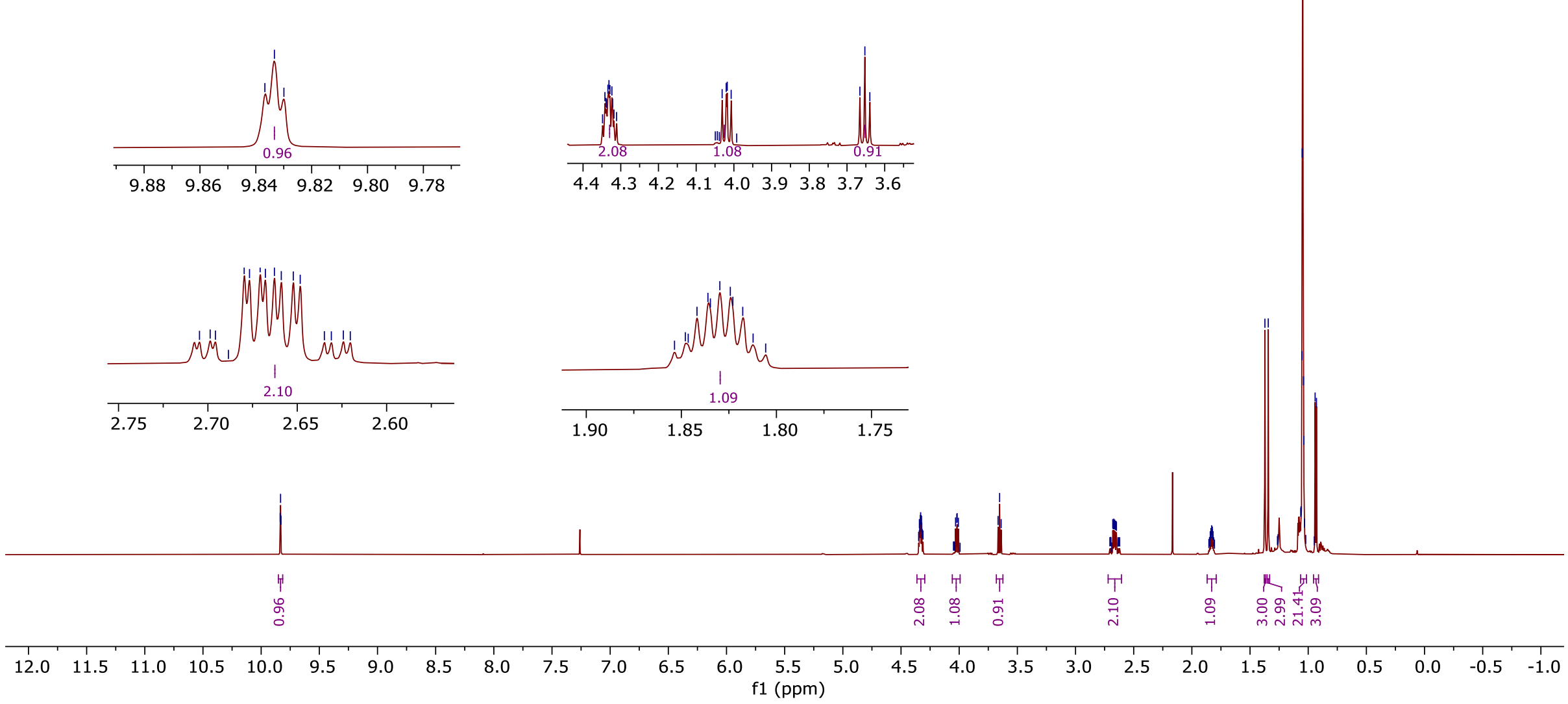

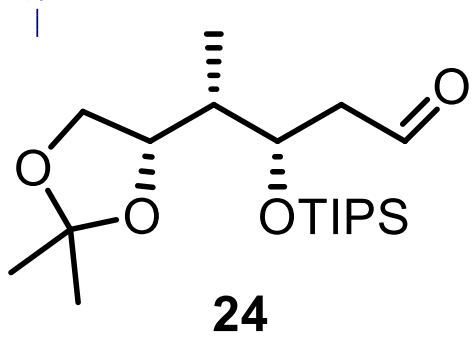

24

^{13}C NMR, CDCl$_3$, 151 MHz

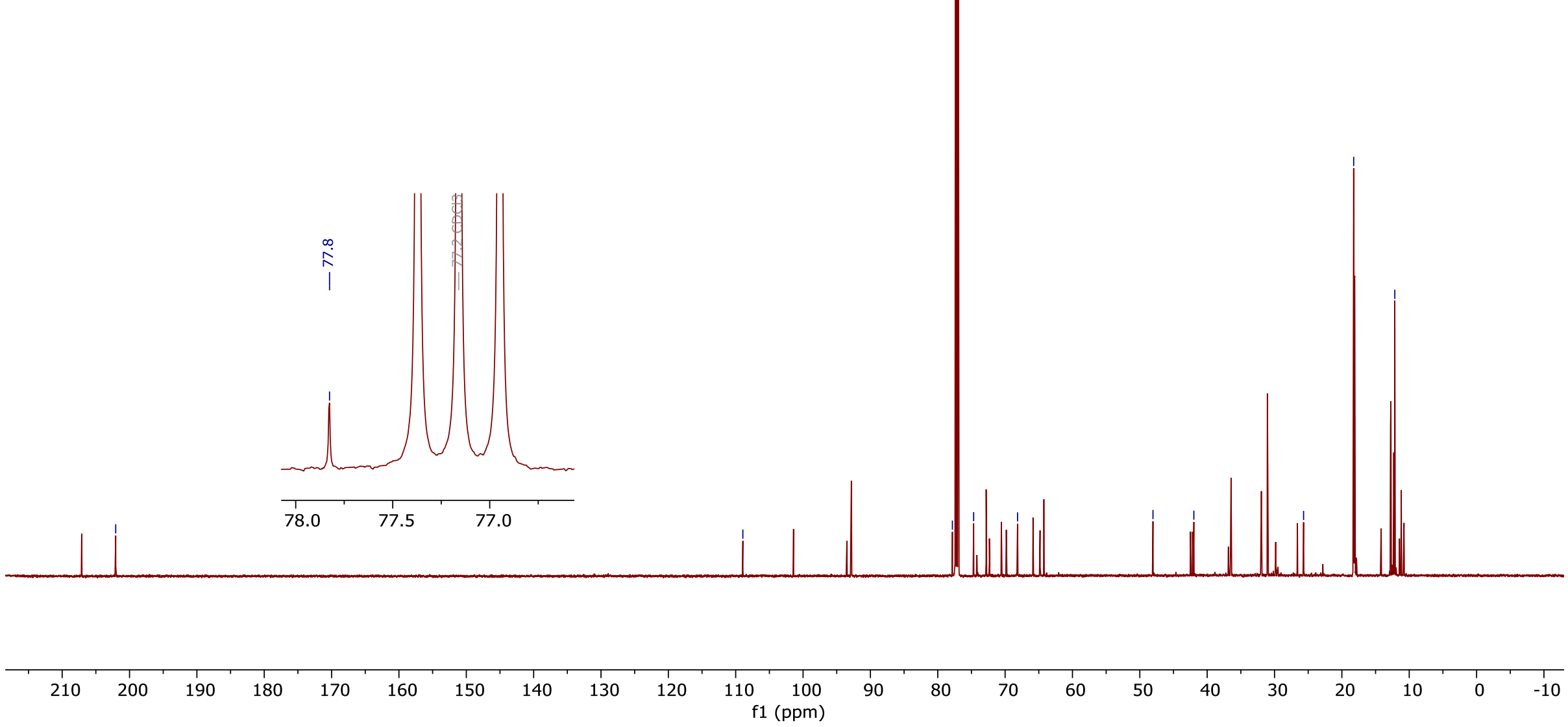

Methyl ester 26

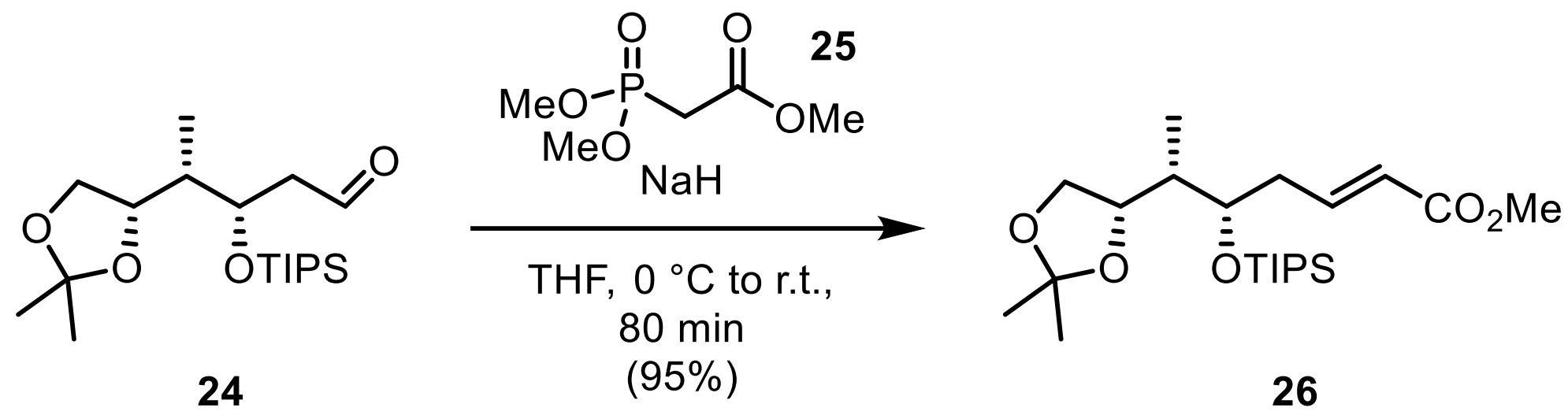

NaH (60% in mineral oil, 246 mg, 6.2 mmol, 1.1 eq.) was suspended in 25 mL dry THF and cooled to 0 °C. Next, trimethyl phosphonoacetate (**25**) (0.96 mL, 5.9 mmol, 1.1 eq.) was added dropwise and the mixture was stirred at 0 °C for 20 min, before dropwise addition of a solution of aldehyde **24** (2.0 g, 5.6 mmol, 1.0 eq.) in 20 mL dry THF. The reaction mixture was stirred for 1 h at r.t., quenched with sat. NH_4Cl solution (50 mL), followed by the addition of water (50 mL). The phases were separated and the aqueous phase was extracted with Et_2O (2 x 70 mL). The combined organic phase was washed with sat. NaCl solution, dried over Na_2SO_4 and concentrated under reduced pressure. The crude product was purified by flash chromatography and afforded methyl ester **26** (2.2 g, 5.3 mmol, 95%), as a colorless oil. For practical reasons, the purification of this step can be avoided.

R_f = 0.50 (pentane/Et_2O, 10:1), vanillin: blue/violet, UV (+).

^{1}H NMR (600 MHz, $CDCl_3$) δ [ppm] = 6.93 (ddd, *J* = 15.4, 8.0, 7.1 Hz, 1H), 5.86 (dt, *J* = 15.6, 1.5 Hz, 1H), 4.19 (dt, *J* = 7.7, 6.2 Hz, 1H), 4.02 (dd, *J* = 8.0, 6.1 Hz, 1H), 3.87 (ddd, *J* = 6.9, 6.0, 2.9 Hz, 1H), 3.72 (s, 3H), 3.61 (t, *J* = 7.9 Hz, 1H), 2.50 (dddd, *J* = 14.8, 8.2, 6.9, 1.4 Hz, 1H), 2.41 (dddd, *J* = 14.5, 7.4, 6.1, 1.6 Hz, 1H), 1.75 – 1.67 (m, 1H), 1.38 (s, 4H), 1.34 (s, 3H), 1.05 (s, 21H), 0.98 (d, *J* = 7.0 Hz, 3H).

^{13}C NMR (151 MHz, $CDCl_3$) δ [ppm] = 166.9, 146.1, 123.1, 108.7, 76.3, 73.9, 68.3, 51.6, 41.8, 37.5, 26.8, 25.7, 18.3, 18.3, 13.0, 10.6.

IR (ATR) ṽ (cm^{-1}) = 2944 (s), 2892 (m), 2867 (s), 1726 (s), 1658 (w), 1462 (m), 1436 (w), 1370 (m), 1323 (m), 1211 (m), 1166 (s), 1040 (s), 882 (s), 677 (s), 510 (w).

HRMS (ESI) (*m/z*) calcd. for $[C_{22}H_{42}O_5SiH]^+$ = $[M+H]^+$: 415.2880, found: 415.2878.

Specific Rotation $[\alpha]_{589}^{20}$ = –6.0° (c = 0.4, $CHCl_3$).

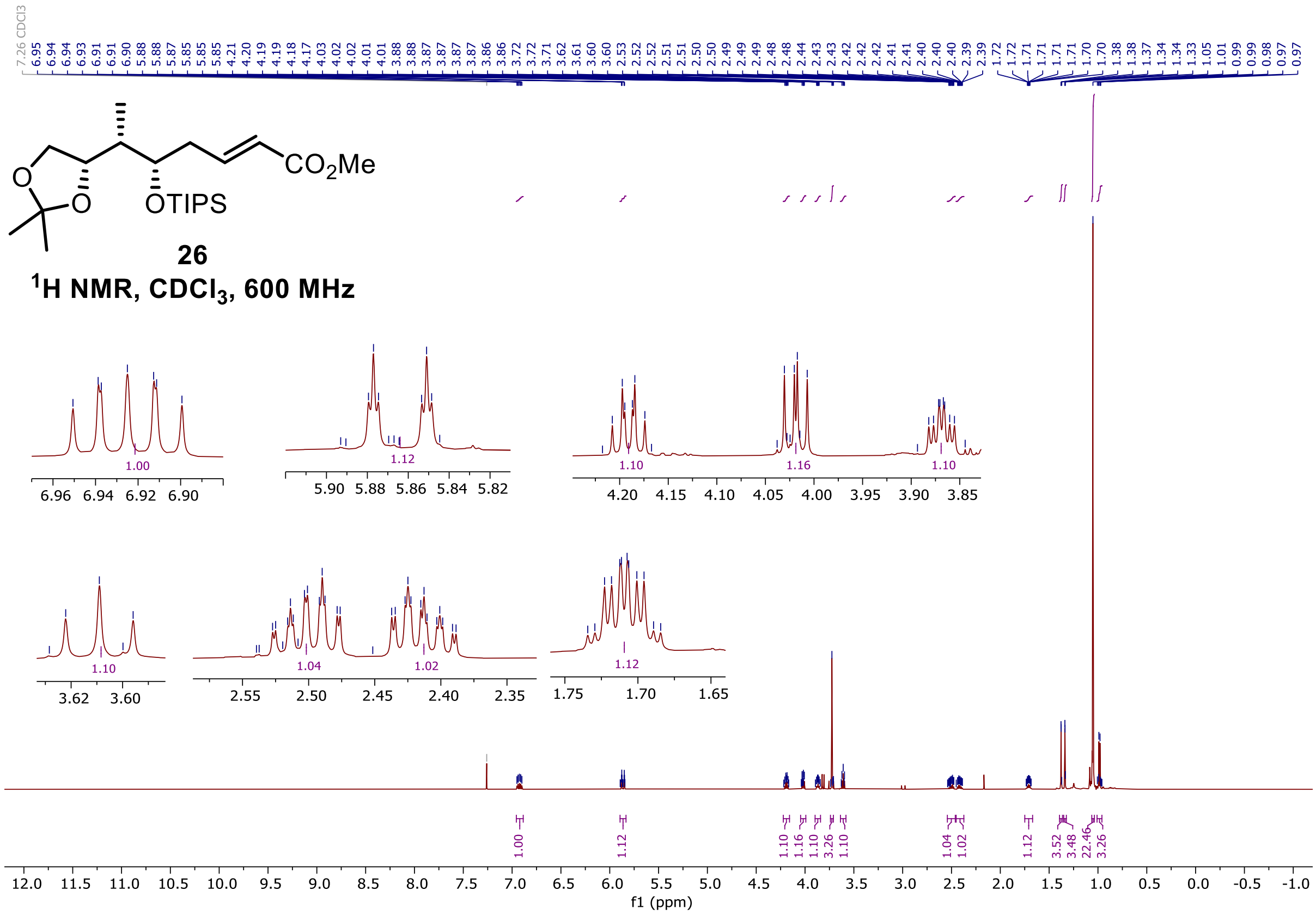
CO2Me
OTIPS
26
1H NMR, CDCl3, 600 MHz
f1 (ppm)

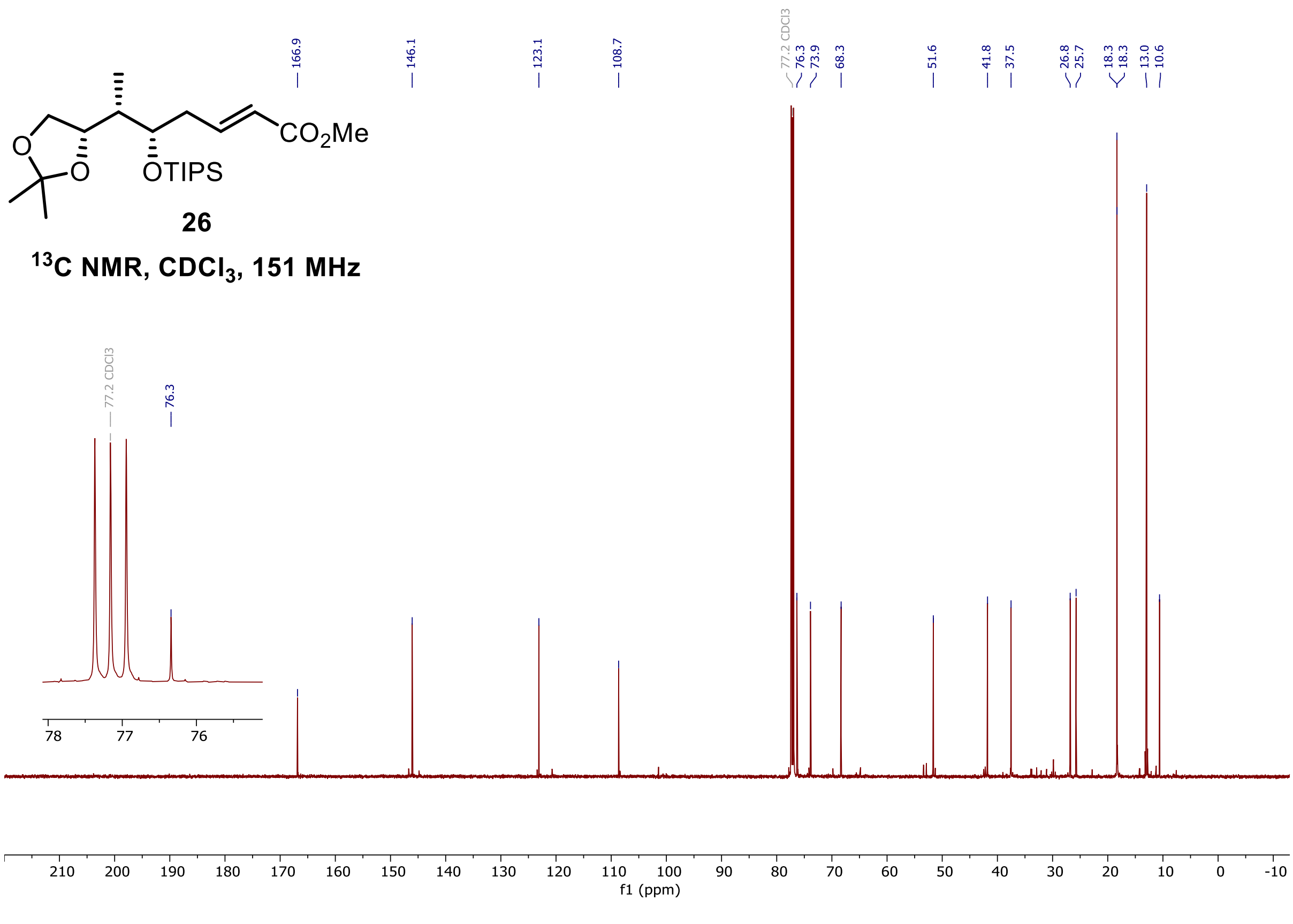
CO2Me
OTIPS
26
13C NMR, CDCl3, 151 MHz
f1 (ppm)

Allylic alcohol 27

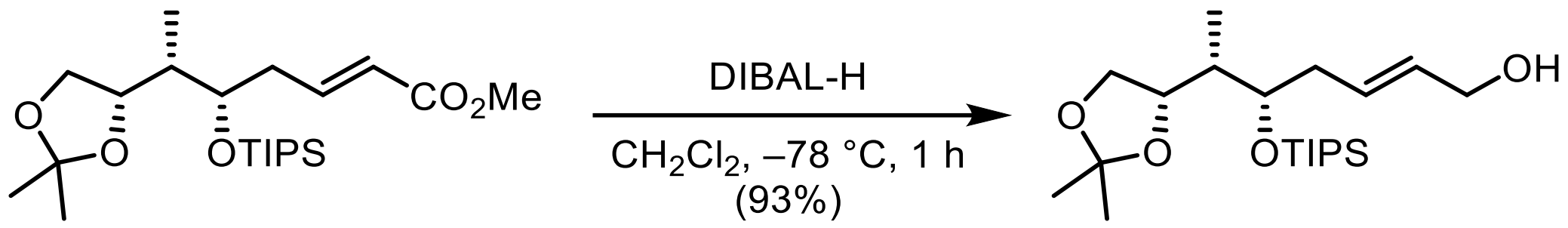

26 **27**

Ester **26** (2.3 g, 5.6 mmol, 1.0 eq.) was dissolved in 40 mL dry CH_2Cl_2 and cooled to –78 °C. DIBAL-H (1M in CH_2Cl_2, 12.2 mL, 12.2 mmol, 2.2 eq) was added dropwise and stirring was continued for 1 h. The mixture was warmed to r.t. and 20 mL MeOH was added dropwise. Then, 100 mL of sat. potassium/sodium tartrate solution (100 mL) and CH_2Cl_2 (100 mL) were added slowly. The gel-like mixture was stirred at r.t. for 1 h, the phases were separated and the aqueous phase was extracted with CH_2Cl_2 (2 x 50 mL). The combined organic phase was washed with sat. NaCl solution and dried over Na_2SO_4. After filtration and concentration, the crude product was purified by flash chromatography (pentane/Et_2O, 3:1), yielding allylic alcohol **27** (2.0 g, 5.2 mmol, 93%) as a colorless oil.

R_f = 0.17 (pentane/Et_2O, 3:1), vanillin: dark blue, UV (-).

^{1}H NMR (600 MHz, $CDCl_3$) δ [ppm] = 5.74 – 5.57 (m, 2H), 4.15 (td, *J* = 7.6, 6.0 Hz, 1H), 4.11 – 4.07 (m, 2H), 4.03 (dd, *J* = 7.9, 6.0 Hz, 1H), 3.78 – 3.74 (m, 1H), 3.61 (t, *J* = 7.8 Hz, 1H), 2.39 – 2.31 (m, 1H), 2.31 – 2.25 (m, 1H), 1.75 – 1.67 (m, 3H), 1.38 (s, 3H), 1.34 (s, 3H), 1.05 (s, 18H), 1.00 (d, *J* = 6.9 Hz, 3H).

^{13}C NMR (151 MHz, $CDCl_3$) δ [ppm] = 131.7, 129.1, 108.4, 77.4, 74.2, 68.5, 63.7, 41.4, 37.7, 27.0, 25.8, 18.4, 18.4, 13.0, 10.6.

IR (ATR) ṽ (cm^{-1}) = 3404 (br), 2941 (s), 2892 (m), 2866 (s), 1744 (w), 1462 (m), 1379 (m), 1248 (m), 1215 (m), 1159 (m), 1111 (s), 1039 (s), 1011 (s), 971 (s), 918 (w), 882 (s), 735 (w), 676 (s), 508 (w).

HRMS (ESI) (*m/z*) calcd. for $[C_{21}H_{42}O_4SiH]^+$ = $[M+H]^+$: 387.2931, found: 387.2949.

Specific Rotation $[\alpha]_{589}^{20}$= +3.0° (c = 0.28, $CHCl_3$).

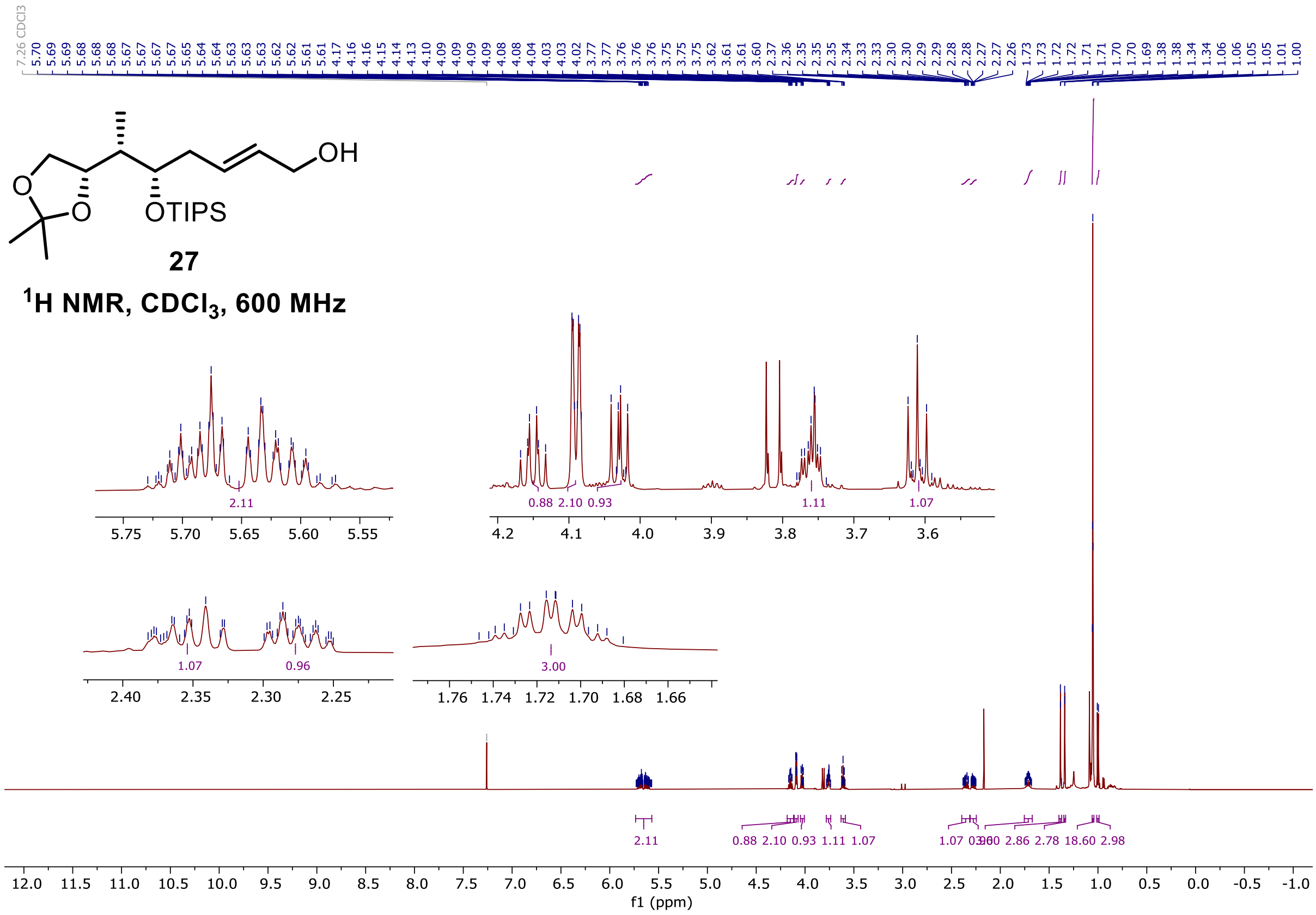
OH
OTIPS
O
O
27
^{1}H NMR, CDCl$_3$, 600 MHz
2.11
0.88 2.10 0.93
1.11
1.07
1.07
0.96
3.00
2.11
0.88 2.10 0.93 1.11 1.07
1.07 0.96 3.00 2.86 2.78 18.60 2.98
f1 (ppm)

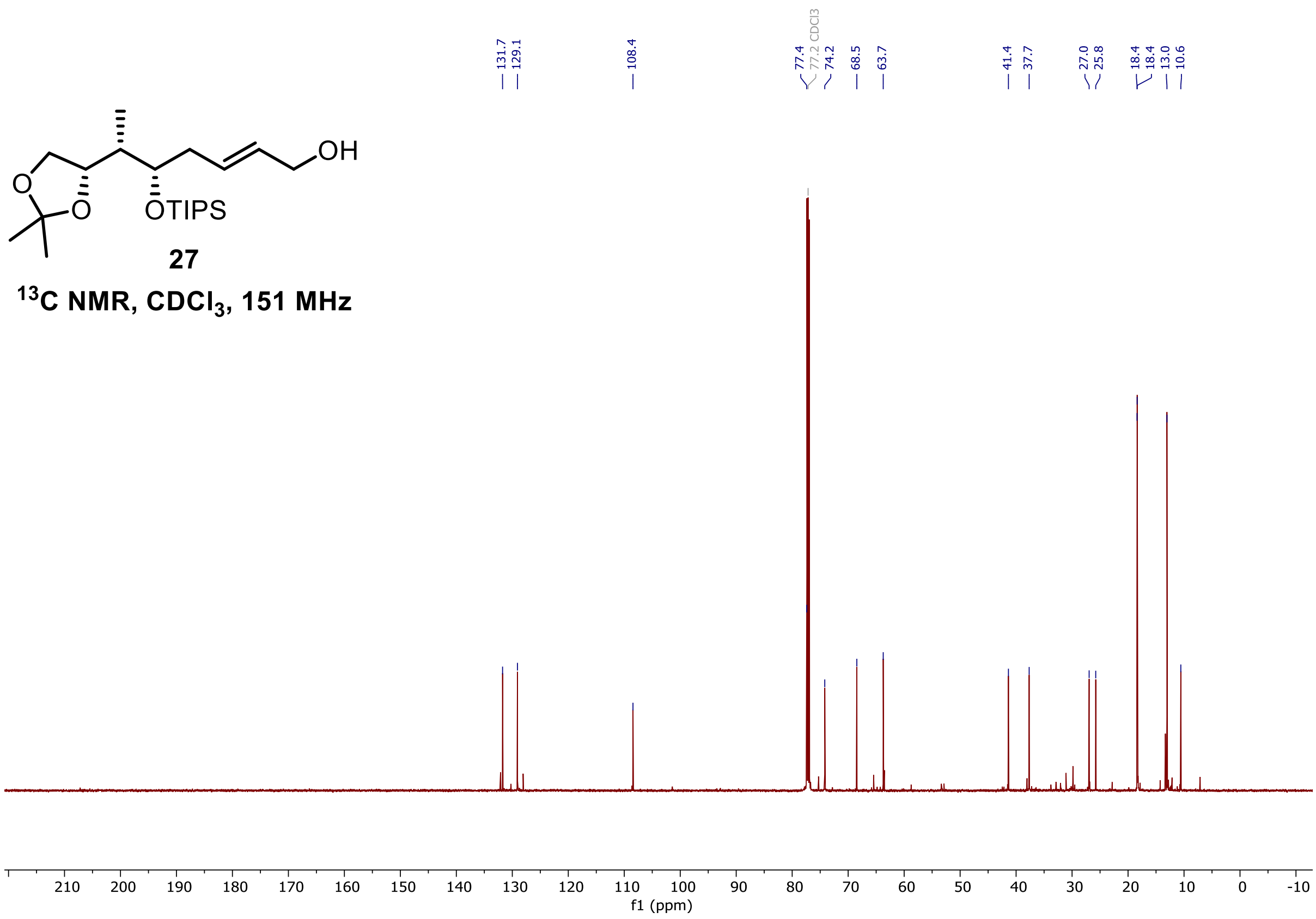
131.7
129.1
108.4
77.4
77.2 CDCl3
74.2
68.5
63.7
41.4
37.7
27.0
25.8
18.4
18.4
13.0
10.6
OH
OTIPS
O
O
27
^{13}C NMR, CDCl$_3$, 151 MHz
f1 (ppm)

Epoxide 13

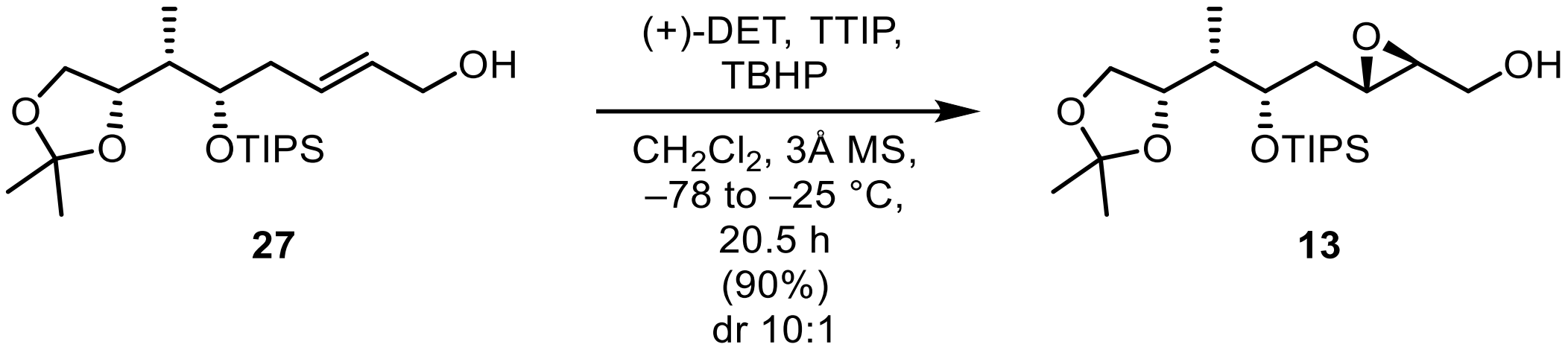

In a round-bottom flask, 4 g powdered 3Å molecular sieve, titanium isopropoxide (TTIP) (2.8 mL, 9.4 mmol, 1.1 eq.) and (+)-diethyl tartrate (2.8 mL, 13.3 mmol, 1.6 eq.) were dissolved in 70 mL dry CH_2Cl_2. The reaction mixture was cooled to –78 °C and stirred for 30 min. Then, allylic alcohol **27** (3.3 g, 8.5 mmol, 1.0 eq.), dissolved in 20 mL dry CH_2Cl_2, was added dropwise and the mixture was stirred for additional 20 min. Subsequently, *tert*-butyl hydroperoxide (TBHP) solution (5.5 M in decane, 3.9 mL, 21.3 mmol, 2.5 eq.) was slowly added and the mixture was allowed to warm up to –25 °C at which temperature it was stirred for 20 h. After completion of the reaction, the molecular sieve was filtered off, the filtrate was quenched with water (2 mL) and diluted with EtOAc (100 mL). The heterogeneous mixture was stirred vigorously for 30 min and then filtered through a short pad of Celite. After removal of the solvent, the crude product was purified by flash chromatography (pentane/Et_2O, 1:1) to obtain epoxide **13** (3.1 g, 7.7 mmol, 90%, dr 10:1) as a colorless oil.

R_f = 0.48 (pentane/Et_2O, 1:1), vanillin: violet, UV (-).

^{1}H NMR (600 MHz, $CDCl_3$) δ [ppm] = 4.24 (dt, *J* = 8.0, 5.8 Hz, 1H), 4.01 (dd, *J* = 8.0, 6.2 Hz, 1H), 3.97 (ddd, *J* = 7.9, 5.0, 2.8 Hz, 1H), 3.89 (ddd, *J* = 12.6, 5.5, 2.6 Hz, 1H), 3.67 – 3.57 (m, 2H), 3.07 (ddd, *J* = 8.1, 3.6, 2.3 Hz, 1H), 2.95 (dt, *J* = 4.5, 2.4 Hz, 1H), 1.91 – 1.80 (m, 2H), 1.59 (ddd, *J* = 14.6, 8.0, 5.1 Hz, 1H), 1.37 (s, 3H), 1.33 (s, 3H), 1.06 (s, 21H), 0.95 (d, *J* = 7.1 Hz, 3H).

^{13}C NMR (151 MHz, $CDCl_3$) δ [ppm] = 108.7, 75.7, 73.2, 68.3, 61.7, 58.9, 53.6, 41.9, 36.2, 26.8, 25.8, 18.3, 13.0, 10.8.

IR (ATR) ṽ (cm^{-1}) = 3468 (br), 2980 (m), 2942 (m), 2867 (m), 1743 (s), 1463 (m), 1370 (m), 1249 (s), 1216 (s), 1129 (s), 1091 (s), 1054 (s), 1016 (s), 882 (m), 864 (m), 677 (m), 607 (w), 508 (w).

HRMS (ESI) (*m/z*) calcd. for $[C_{21}H_{42}O_5SiH]^+$ = $[M+H]^+$: 403.2880, found: 403.2876.

Specific Rotation $[\alpha]_{589}^{21}$ = –15.0° (c = 0.26, $CHCl_3$).

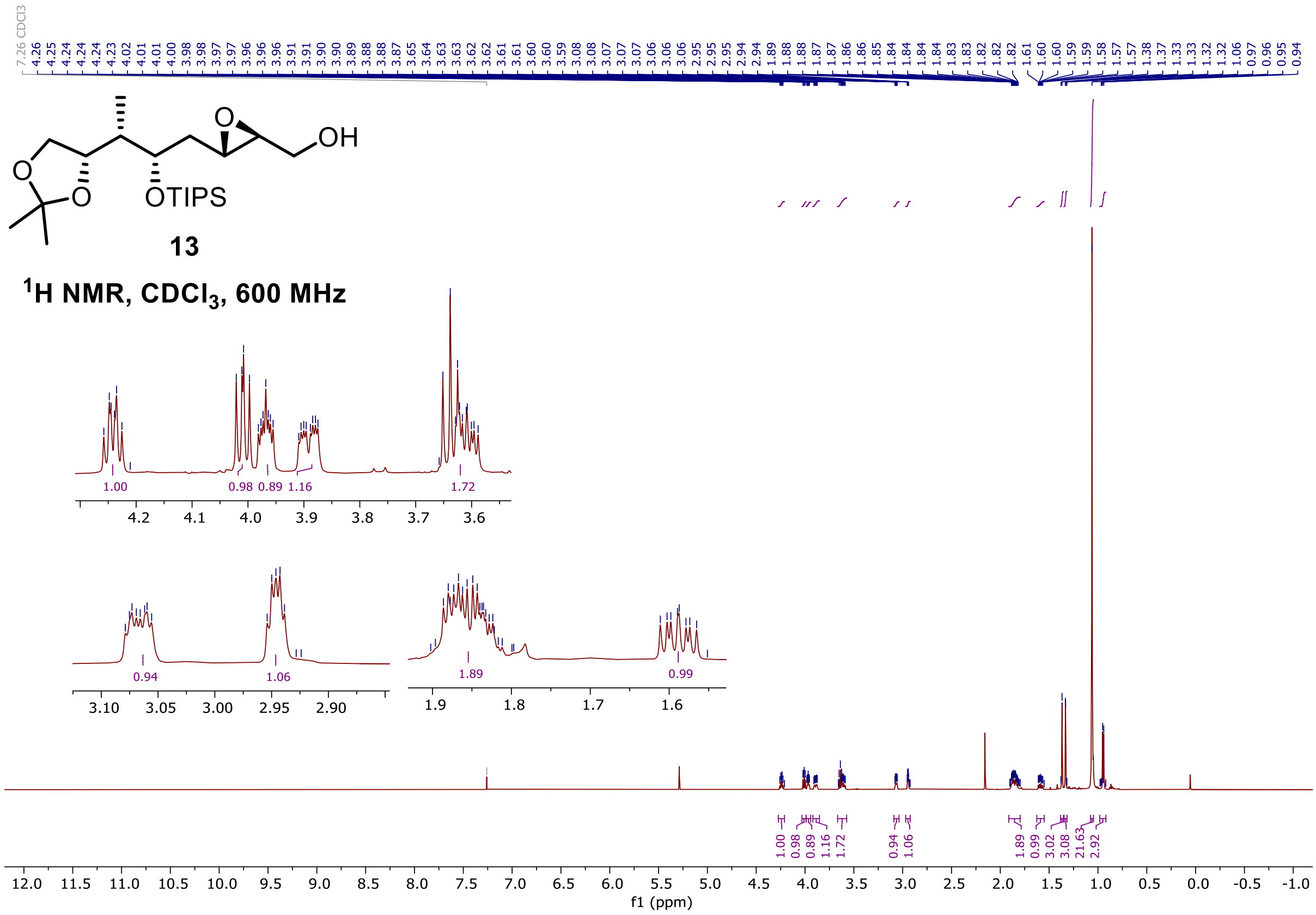

OTIPS
OH
13
¹H NMR, CDCl3, 600 MHz
7.26 CDCl3
1.00 0.98 0.89 1.16 1.72 0.94 1.06 1.89 0.99 3.02 3.08 21.63 2.92
f1 (ppm)

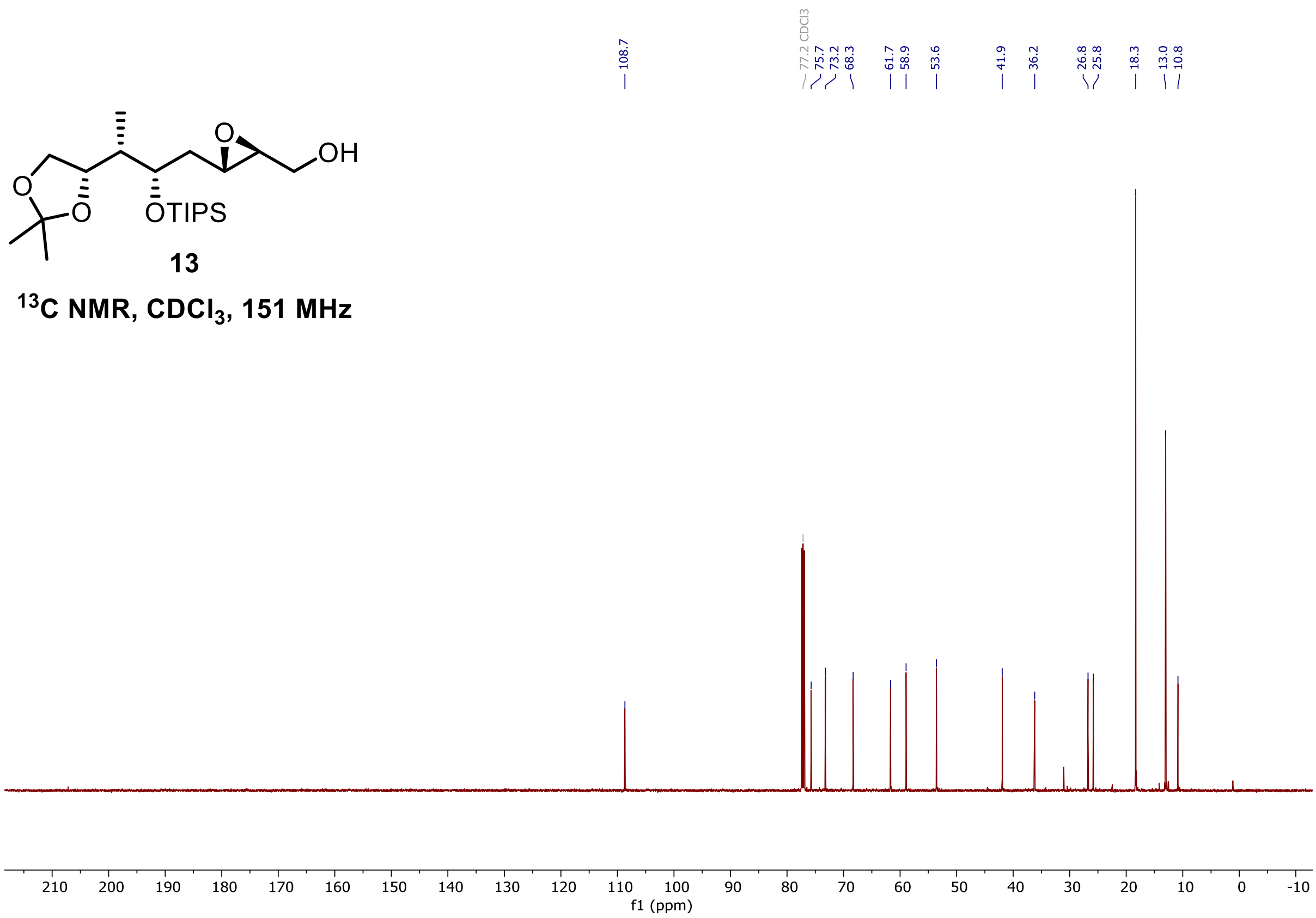

OTIPS
OH
13
¹³C NMR, CDCl3, 151 MHz
108.7
77.2 CDCl3
75.7
73.2
68.3
61.7
58.9
53.6
41.9
36.2
26.8
25.8
18.3
13.0
10.8
f1 (ppm)

Triol 28

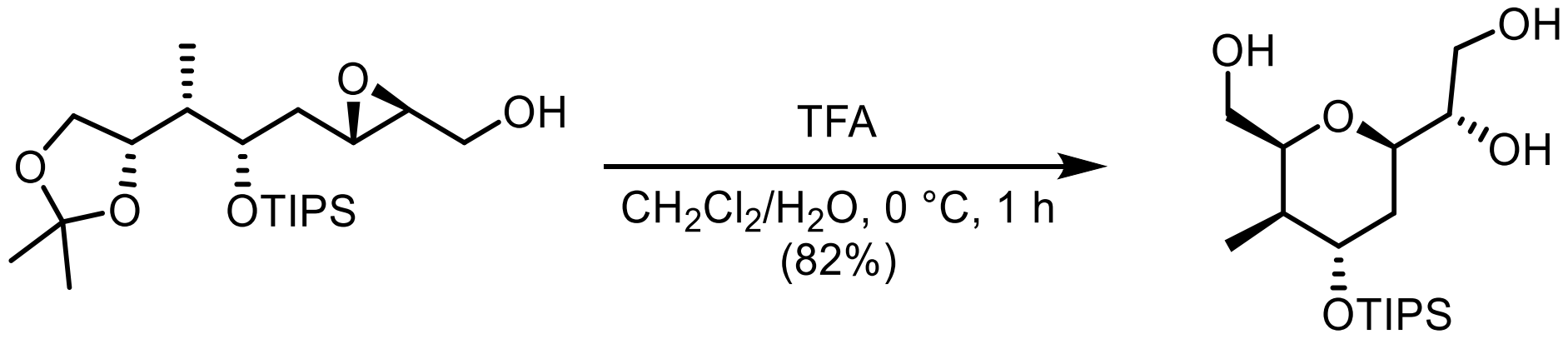

13 **28**

In a round-bottom flask, epoxide **13** (3.1 g, 7.7 mmol, 1.0 eq.) was dissolved in 70 mL dry CH_2Cl_2 and 4 mL of water. The mixture was cooled to 0 °C before addition of 4 mL TFA, which had been pre-cooled to 0 °C, and stirred at 0 °C for 1 h. After completion of the reaction, sat. $NaHCO_3$ solution (100 mL) was added dropwise and the mixture was stirred for another 10 min. The phases were separated, the aqueous phase was extracted with CH_2Cl_2 (3 x 50 mL), the combined organic phase was washed with sat. NaCl solution, dried over Na_2SO_4 and concentrated under reduced pressure. The crude product can be used for the next step without any further purification.
For analytical reasons, the crude material was purified by flash chromatography (100% EtOAc), affording triol **28** (2.3 g, 6.3 mmol, 82%) as a colorless, highly viscous oil.

R_f = 0.34 (EtOAc), vanillin: brown/violet, UV (-).

^{1}H NMR (600 MHz, $CDCl_3$) δ [ppm] = 4.11 – 4.02 (m, 2H), 3.93 (q, *J* = 2.9 Hz, 1H), 3.79 (dt, *J* = 13.2, 7.0 Hz, 2H), 3.73 – 3.63 (m, 3H), 3.56 – 3.39 (m, 2H, 1 x OH), 1.71 (ddd, *J* = 14.4, 12.3, 2.5 Hz, 1H), 1.67 – 1.61 (m, 1H), 1.45 (d br, *J* = 13.4 Hz, 1H), 1.13 – 0.98 (m, 21H), 0.83 (d, *J* = 7.2 Hz, 3H).

^{13}C NMR (151 MHz, $CDCl_3$) δ [ppm] = 75.8, 74.3, 73.6, 70.6, 64.5, 62.8, 37.2, 30.2, 18.2, 12.3, 11.4.

IR (ATR) ṽ (cm^{-1}) = 3337 (br), 2941 (s), 2890 (m), 2866 (s), 1677 (m), 1462 (m), 1384 (m), 1342 (w), 1247 (w), 1202 (m), 1092 (s), 1050 (s), 997 (s), 950 (m), 881 (s), 817 (m), 800 (m), 722 (m), 678 (s), 658 (s), 502 (w), 462 (w).

HRMS (ESI) (*m/z*) calcd. for $[C_{18}H_{38}O_5SiH]^+$ = $[M+H]^+$: 363.2567, found: 363.2564.

Specific Rotation $[\alpha]_{589}^{21}$= +12.2° (c = 0.22, $CHCl_3$).

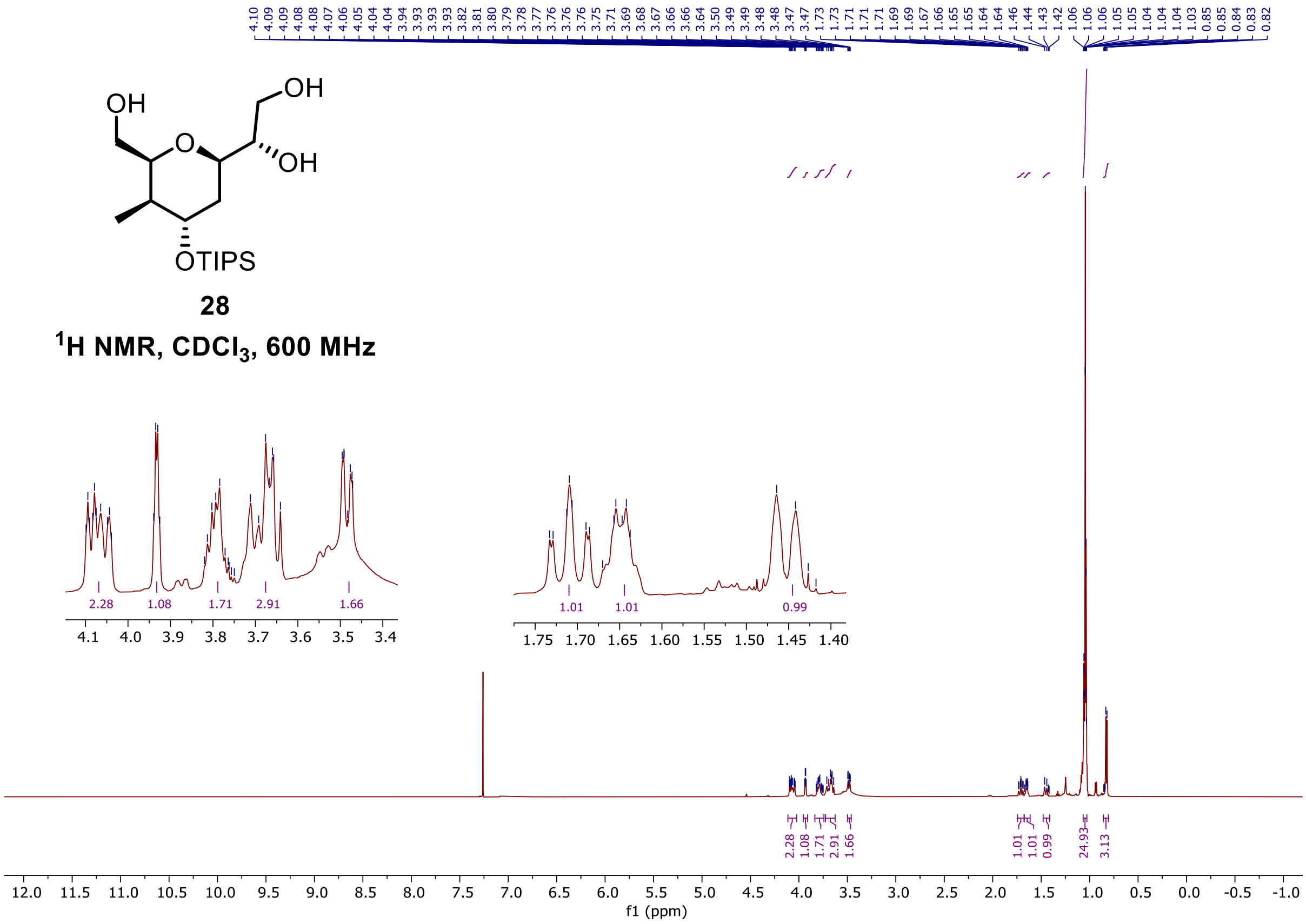

28

^{1}H NMR, $CDCl_3$, 600 MHz

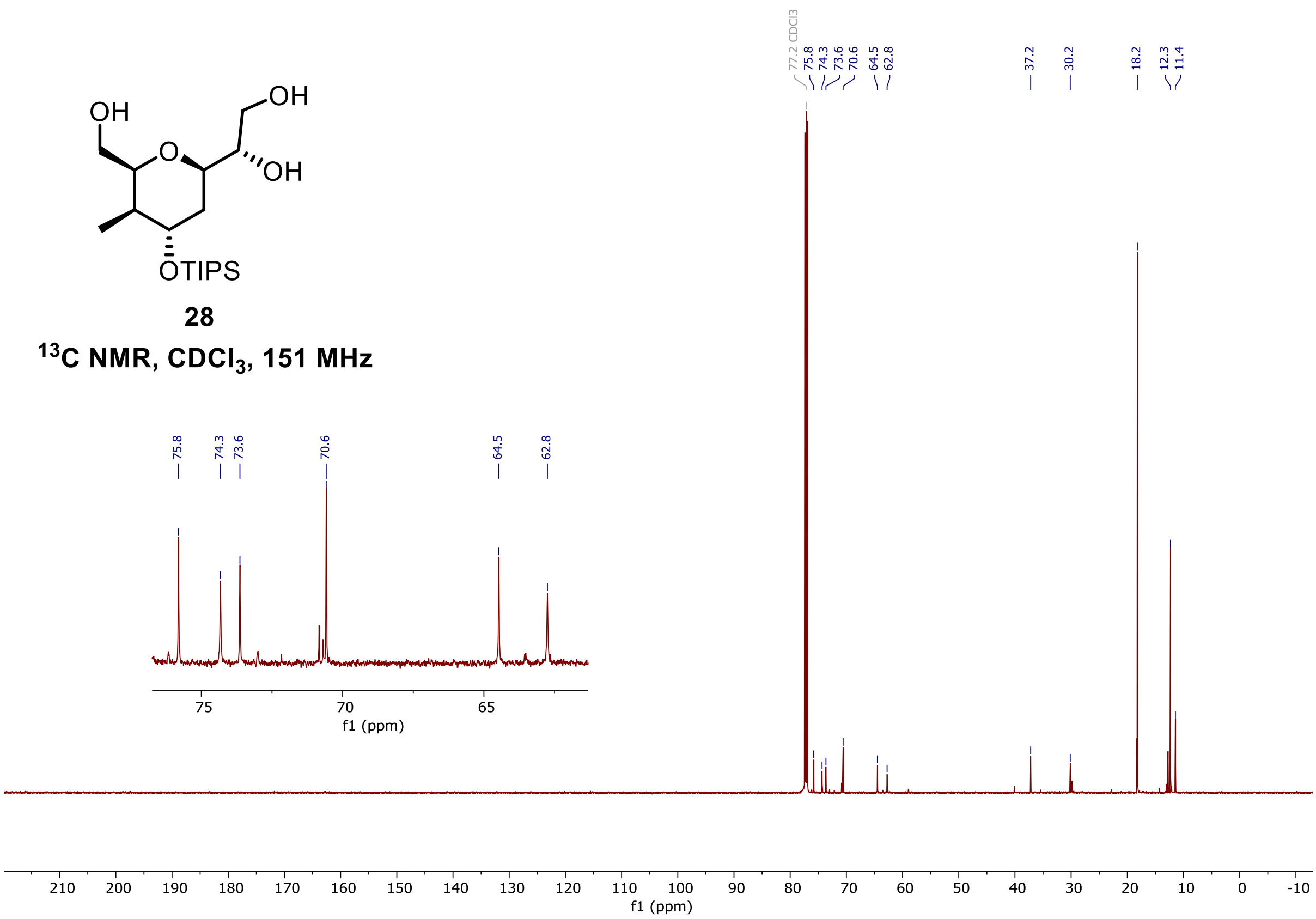

28

^{13}C NMR, $CDCl_3$, 151 MHz

Alcohol 29

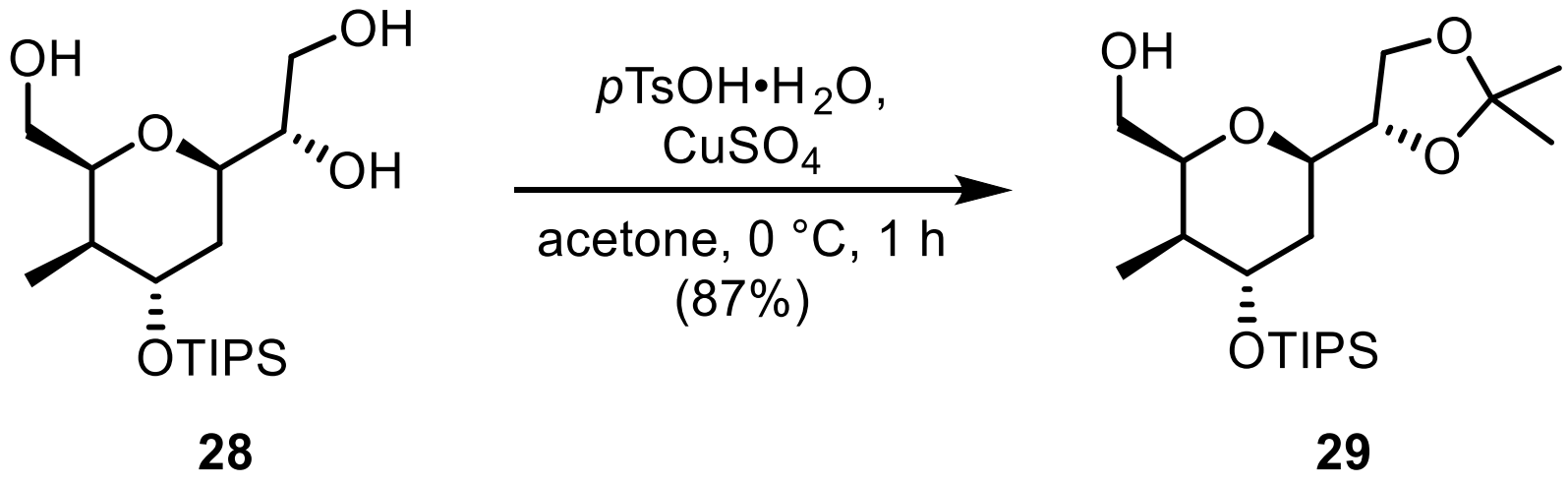

In a round-bottom flask, triol **28** (3.1 g, 8.6 mmol, 1.0 eq.) was dissolved in 75 mL dry acetone, before addition of powdered copper(II) sulfate (2.8 g, 17.5 mmol, 2.1 eq.) and subsequent cooling to 0 °C. Next, *p*TsOH•H_2O (325 mg, 1.7 mmol, 20 mol%) was added and the reaction mixture was stirred for 1 h at 0 °C. After completion of the reaction, stirring was stopped for 30 min for precipitation of the copper(II) sulfate. After decantation, the reaction mixture was diluted with Et_2O and washed with sat. $NaHCO_3$ solution, water and sat. NaCl solution. The organic layer was dried over $MgSO_4$, filtered and concentrated in vacuo. The residue was purified by flash chromatography (pentane/Et_2O, 3:2) to provide acetonide **29** (3.0 g, 7.5 mmol, 87%) as a colorless liquid.

R_f = 0.54 (pentane/Et_2O, 1:1), vanillin: brown, UV (-).

^{1}H NMR (600 MHz, $CDCl_3$) δ [ppm] = 4.09 – 4.03 (m, 2H), 3.99 (td, *J* = 6.5, 5.1 Hz, 1H), 3.95 – 3.89 (m, 2H), 3.78 (ddd, *J* = 11.6, 6.6, 2.3 Hz, 1H), 3.66 (dd, *J* = 11.5, 9.1 Hz, 1H), 3.43 (dd, *J* = 11.5, 3.3 Hz, 1H), 1.92 (s, 1H), 1.71 – 1.62 (m, 2H), 1.59 (ddd, *J* = 13.9, 11.5, 2.5 Hz, 1H), 1.38 (s, 3H), 1.34 (s, 3H), 1.07 – 1.01 (m, 21H), 0.84 (d, *J* = 7.2 Hz, 3H).

^{13}C NMR (151 MHz, $CDCl_3$) δ [ppm] = 109.5, 78.2, 75.3, 73.4, 70.7, 66.8, 64.5, 37.3, 31.0, 26.6, 25.4, 18.2, 12.3, 11.5.

IR (ATR) ṽ (cm^{-1}) = 3453 (b), 2941 (m), 2867 (m), 1463 (m), 1370 (m), 1252 (m), 1216 (m), 1146 (m), 1091 (s), 1064 (s), 995 (m), 946 (m), 919 (w), 881 (s), 845 (s), 819 (m), 756 (m), 711 (m), 678 (s), 505 (m).

HRMS (ESI) (*m/z*) calcd. for $[C_{21}H_{42}O_5SiH]^+$ = $[M+H]^+$: 403.2880, found: 403.2873.

Specific Rotation $[\alpha]^{21}_{589}$= +17.2° (c = 0.23, $CHCl_3$).

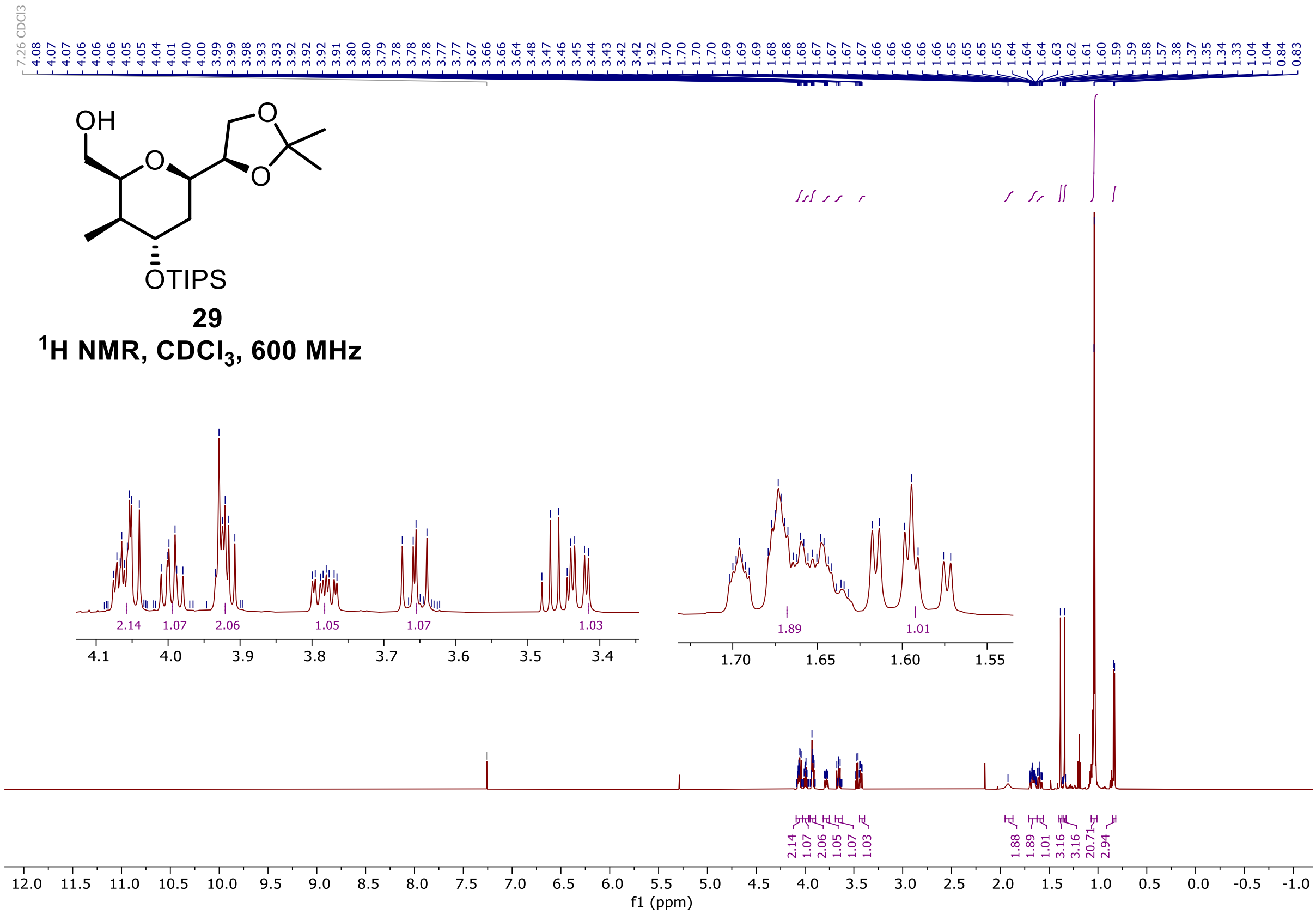

29

^{1}H NMR, $CDCl_3$, 600 MHz

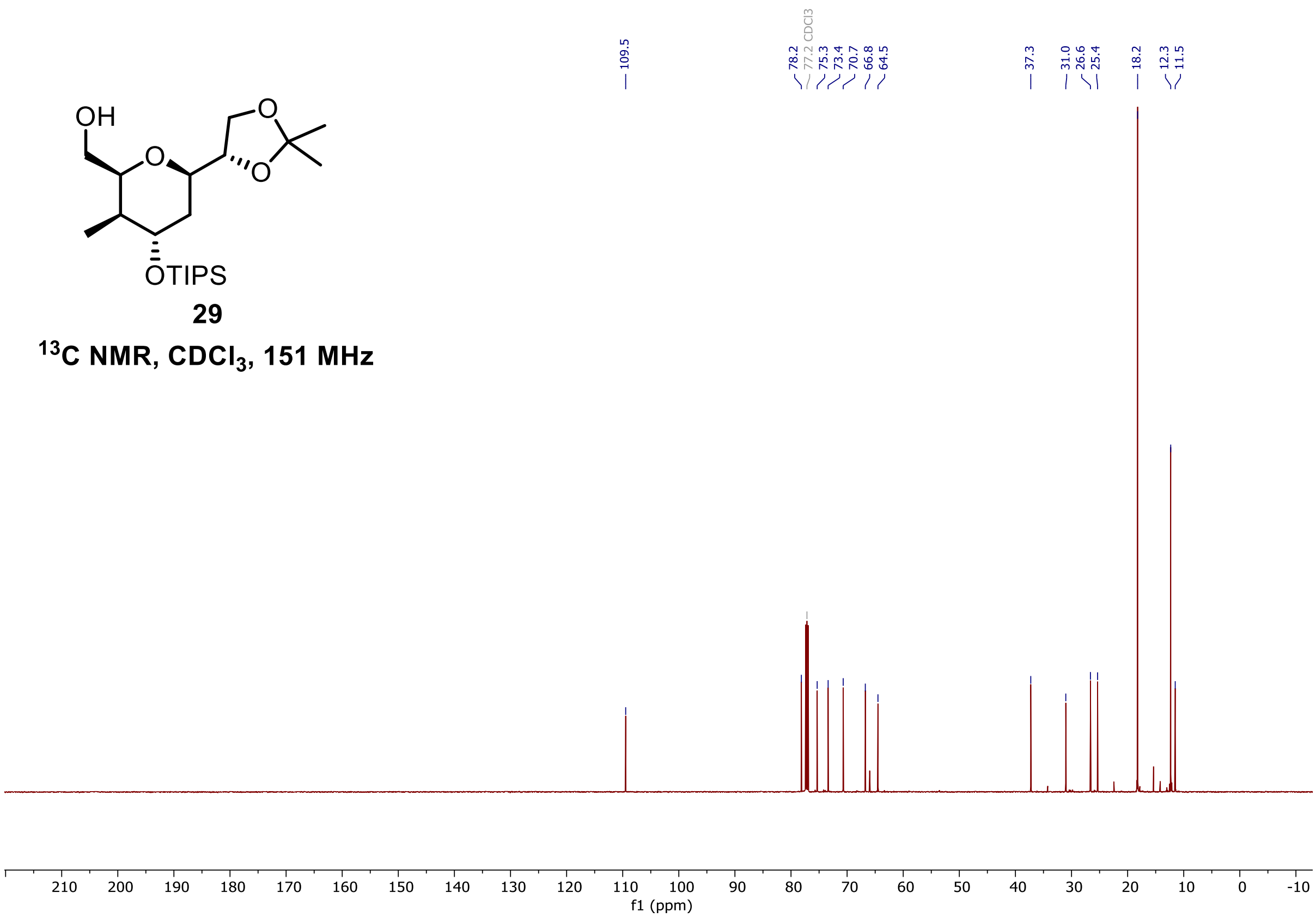

29

^{13}C NMR, $CDCl_3$, 151 MHz

Triflate 29a

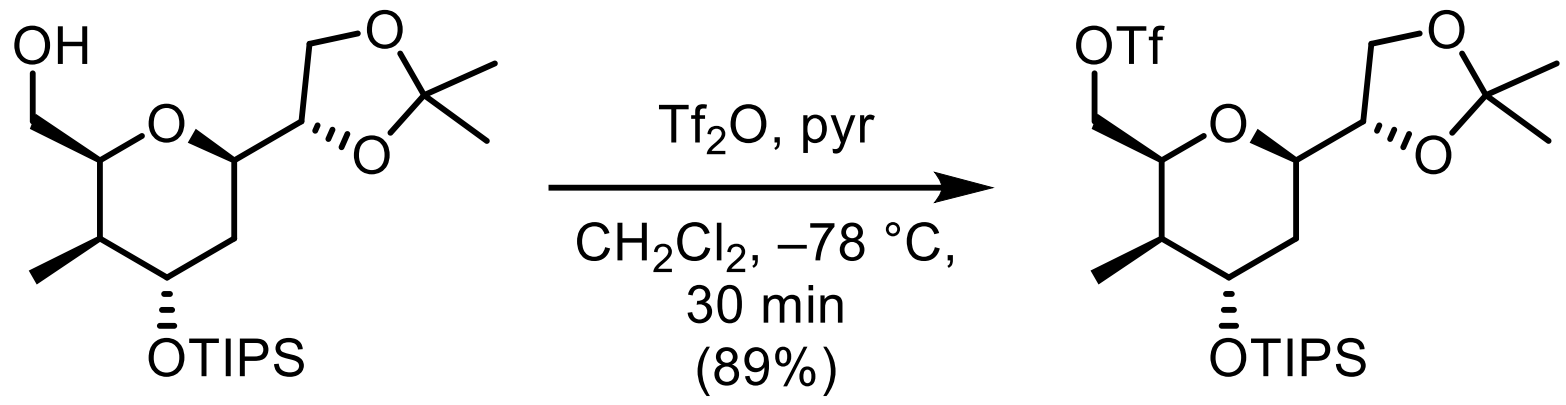

29 **29a**

Acetonide **29** (270 mg, 0.67 mmol, 1.0 eq.) was dissolved in 10 mL dry CH_2Cl_2, before addition of pyridine (165 µL, 2.05 mmol, 3.0 eq). The mixture was cooled to –78 °C and Tf_2O (170 µL, 1.01 mmol, 1.5 eq) was added dropwise and the resulting mixture was stirred at –78 °C for 15 min. The reaction solution was slowly warmed up to 0 °C, filtered ice-cold through a silica pad and washed with a pentane/Et_2O solvent mixture (15:1). The solvent was removed under reduced pressure to afford triflate **29a** (320 mg, 0.60 mmol, 89%) as a colorless oil.

Note: The triflate is stable for a few weeks in the refrigerator but decomposes within a week at r.t. Therefore, it should be used directly after the preparation.

$\mathbf{R_f}$ = 0.38 (pentane/Et_2O, 15:1), vanillin: violet, UV (-).

^{1}H NMR (600 MHz, $CDCl_3$) δ [ppm] = 4.48 (dd, *J* = 10.5, 8.6 Hz, 1H), 4.35 (dd, *J* = 10.5, 3.3 Hz, 1H), 4.30 (ddd, *J* = 8.6, 3.4, 2.4 Hz, 1H), 4.09 – 4.02 (m, 1H), 4.01 – 3.91 (m, 3H), 3.75 (ddd, *J* = 11.7, 7.2, 2.3 Hz, 1H), 1.77 (dddd, *J* = 13.8, 3.5, 2.3, 1.2 Hz, 1H), 1.75 – 1.68 (m, 1H), 1.63 – 1.55 (m, 1H), 1.38 (s, 3H), 1.34 (s, 3H), 1.09 – 0.99 (m, 21H), 0.88 (d, *J* = 7.1 Hz, 3H).

^{13}C NMR (151 MHz, $CDCl_3$) δ [ppm] = 119.8, 117.7, 109.5, 77.9, 77.6, 73.8, 71.8, 70.3, 67.0, 37.1, 31.0, 26.7, 25.4, 18.2, 12.3, 11.0.

IR (ATR) ṽ (cm^{-1}) = 2943 (m), 2891 (m), 2868 (m), 1463 (w), 1414 (s), 1382 (m), 1246 (s), 1203 (s), 1145 (s), 1110 (m), 1068 (s), 1046 (s), 997 (m), 945 (s), 881 (s), 845 (m), 818 (m), 797 (m), 761 (w), 710 (w), 680 (s), 657 (m), 613 (s), 501 (m), 463 (w).

HRMS (ESI) (*m/z*) The exact mass could not be detected using various ionization methods (ESI/EI).

Specific Rotation $[\alpha]_{589}^{21}$= +7.0° (c = 5.4, $CHCl_3$).

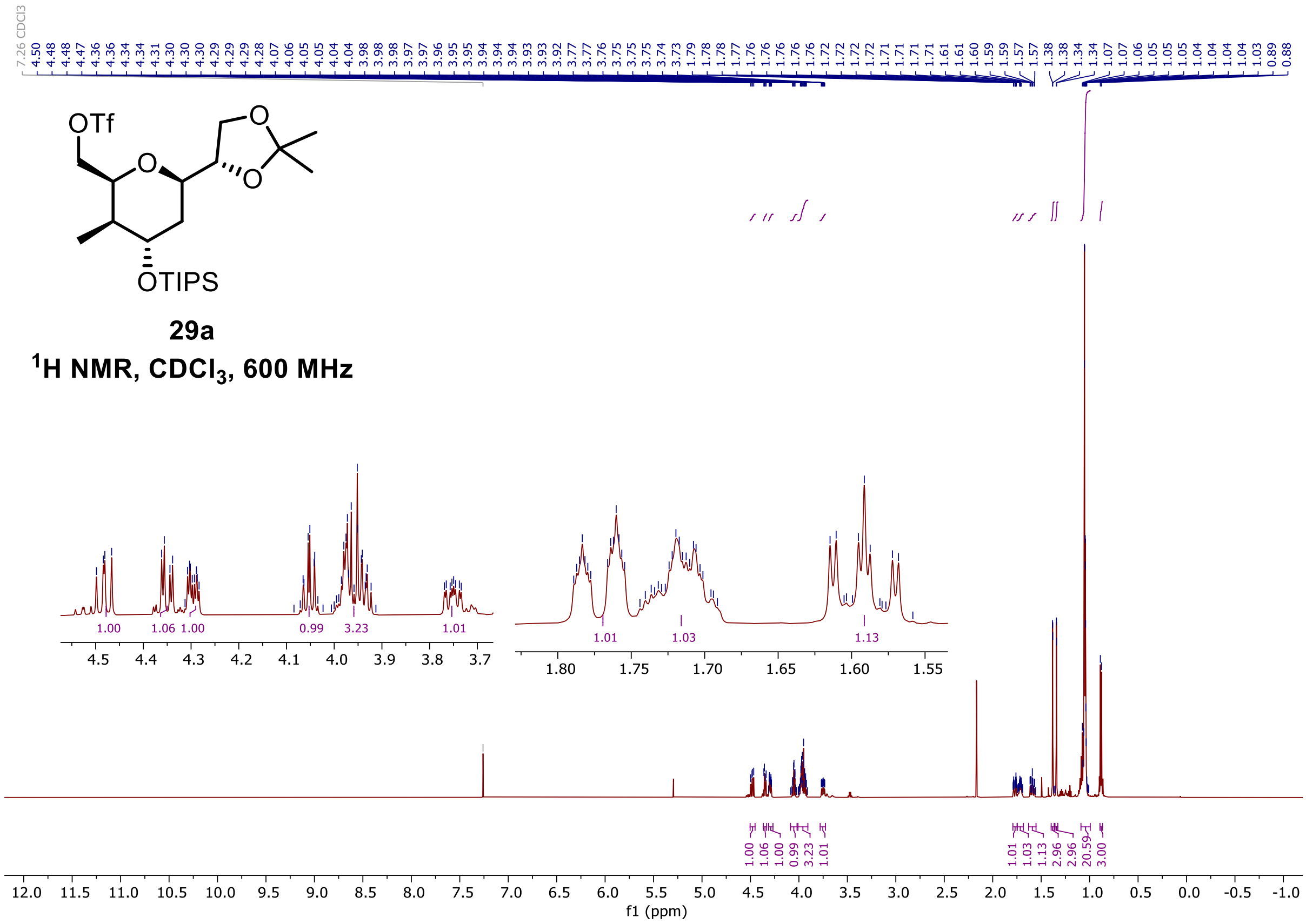

29a

^{1}H NMR, CDCl$_3$, 600 MHz

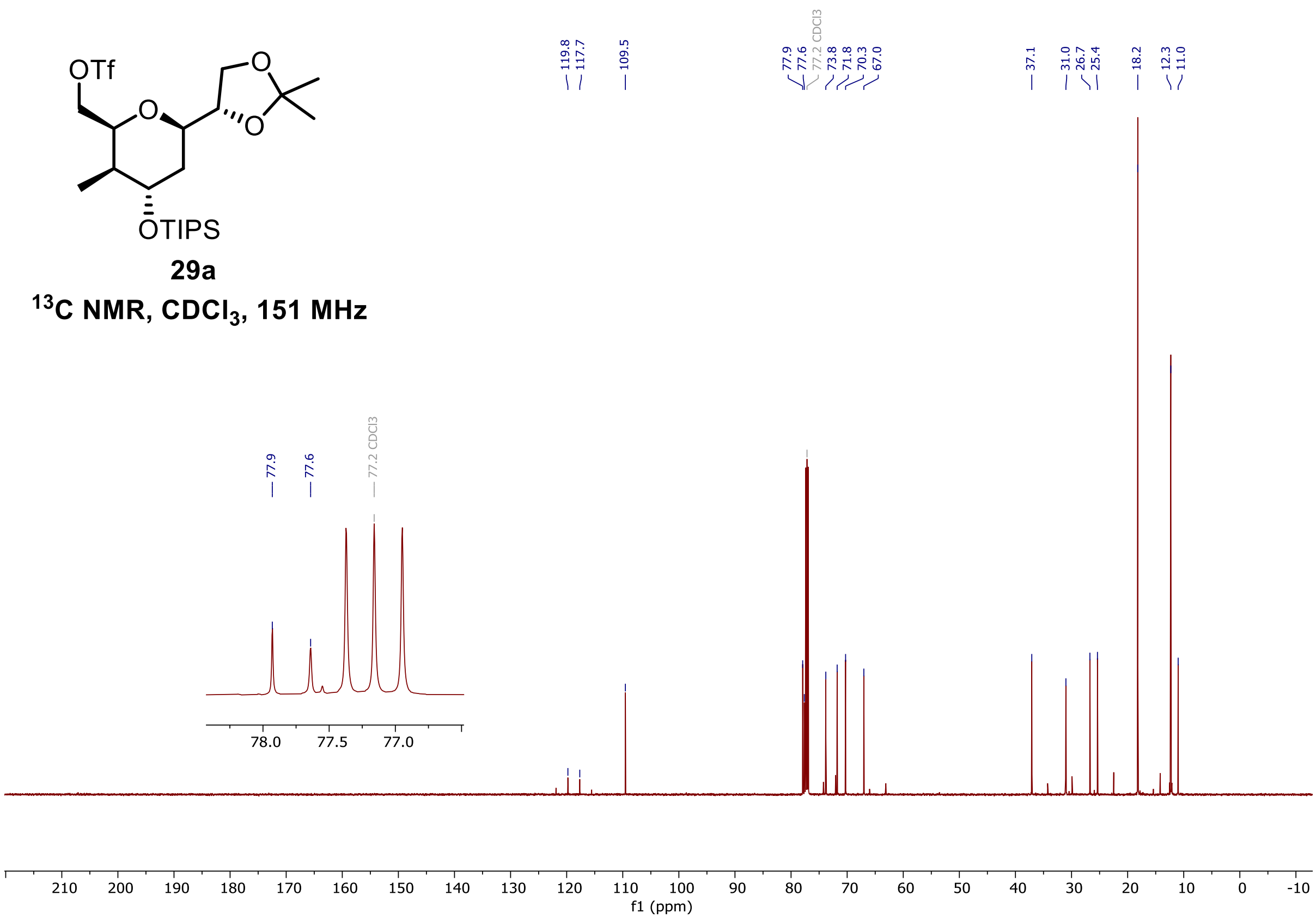

29a

^{13}C NMR, CDCl$_3$, 151 MHz

Nitrile 12

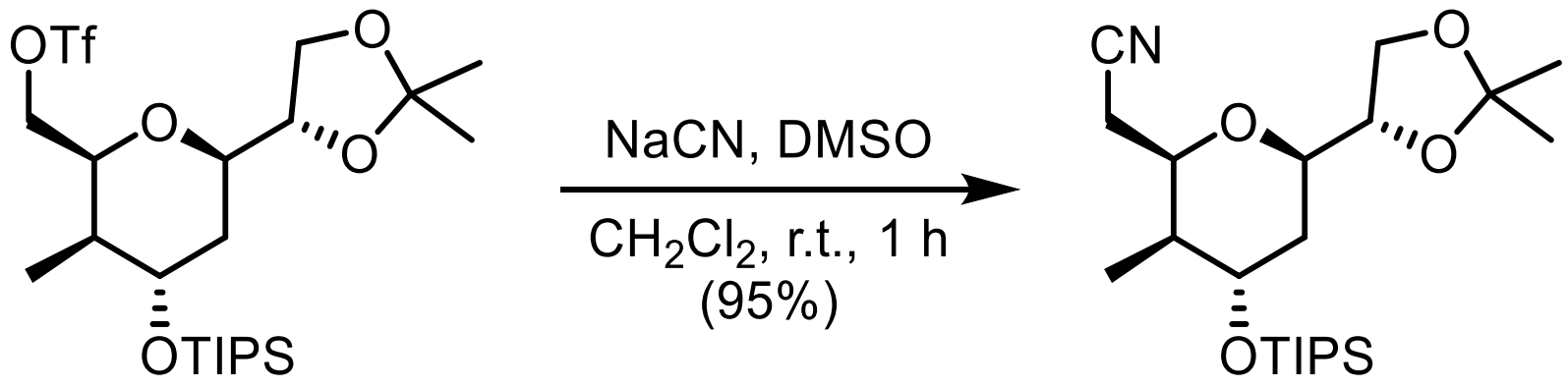

29a **12**

In a round-bottom flask, NaCN (88 mg, 1.80 mmol, 3.0 eq.) was dissolved in 20 mL dry DMSO. A solution of triflate **29a** (320 mg, 0.60 mmol, 1.0 eq.) in 3 mL dry CH_2Cl_2 was added dropwise and the reaction mixture was stirred for 1 h at r.t. After quenching with sat. $NaHCO_3$ solution (20 mL) and diluting with Et_2O (100 mL), the layers were separated and the organic layer was washed with water and sat. NaCl solution, dried over Na_2SO_4 and concentrated in vacuo. The crude product was purified by flash chromatography (pentane/Et_2O, 5:1) to afford nitrile **12** (234 mg, 0.57 mmol, 95%) as a white, crystalline solid.

$\mathbf{R_f}$ = 0.42 (pentane/Et_2O, 5:1), vanillin: grey, UV (-).

^{1}H NMR (600 MHz, $CDCl_3$) δ [ppm] = 4.27 (ddd, *J* = 8.6, 6.4, 2.4 Hz, 1H), 4.05 (td, *J* = 5.4, 2.0 Hz, 1H), 4.01 (q, *J* = 3.0 Hz, 1H), 3.98 – 3.92 (m, 2H), 3.79 (ddd, *J* = 11.7, 6.8, 2.3 Hz, 1H), 2.54 (dd, *J* = 16.6, 8.3 Hz, 1H), 2.35 (dd, *J* = 16.7, 6.5 Hz, 1H), 1.77 (dtd, *J* = 6.9, 2.8, 1.2 Hz, 1H), 1.72 (dddd, *J* = 13.8, 3.5, 2.3, 1.2 Hz, 1H), 1.57 (ddd, *J* = 14.0, 11.6, 2.5 Hz, 1H), 1.39 (s, 3H), 1.34 (s, 3H), 1.05 (dt, *J* = 5.4, 1.6 Hz, 21H), 0.89 (d, *J* = 7.2 Hz, 3H).

^{13}C NMR (151 MHz, $CDCl_3$) δ [ppm] = 117.6, 109.6, 77.9, 74.1, 70.4, 70.3, 66.8, 38.5, 30.5, 26.7, 25.4, 21.6, 18.2, 18.2, 12.3, 10.5.

IR (ATR) ṽ (cm^{-1}) = 3407 (br), 2942 (m), 2891 (m), 2866 (m), 1712 (w), 1463 (m), 1416 (w), 1384 (w), 1242 (m), 1209 (s), 1146 (m), 1067 (s), 1030 (s), 953 (m), 881 (s), 819 (m), 678 (s), 640 (s), 614 (m), 500 (w), 461 (w).

HRMS (ESI) (*m/z*) calcd. for $[C_{22}H_{41}NO_4SiH]^+$ = $[M+H]^+$: 412.2883, found: 412.2882.

Specific Rotation $[\alpha]_{589}^{21}$= +17.0° (c = 0.35, $CHCl_3$).

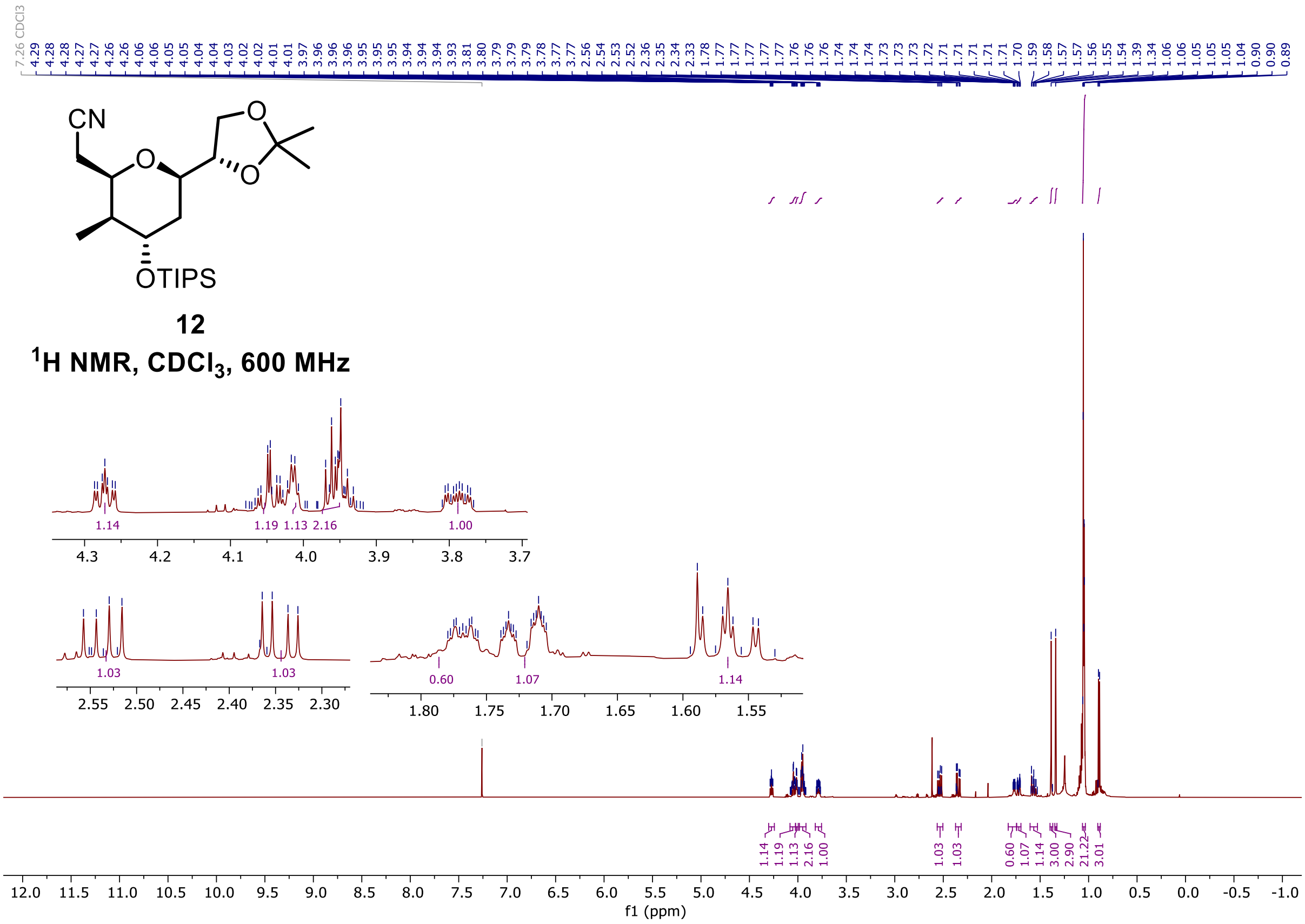
CN
O
O
O
OTIPS
12
1H NMR, CDCl3, 600 MHz
f1 (ppm)

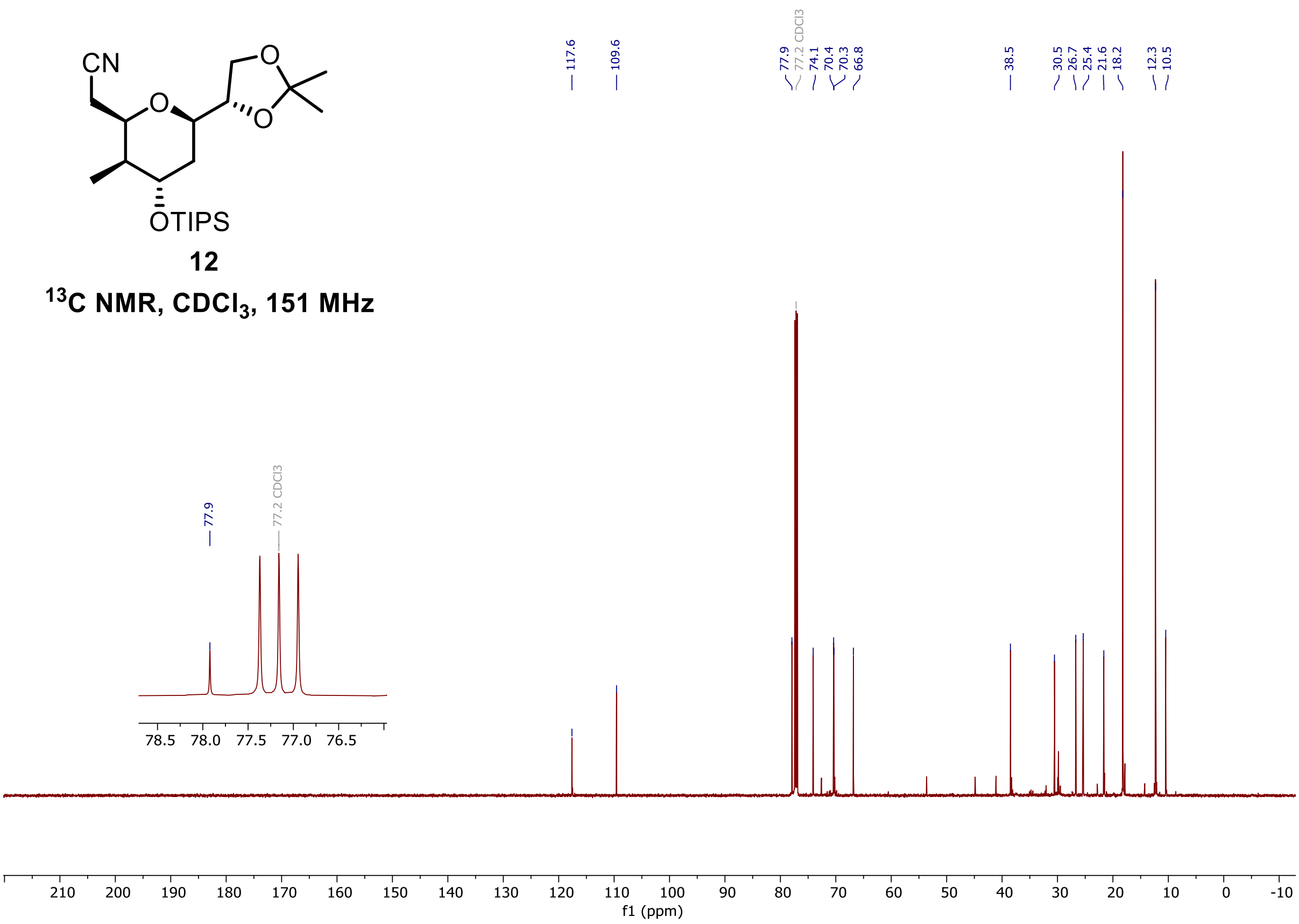
CN
O
O
O
OTIPS
12
13C NMR, CDCl3, 151 MHz
f1 (ppm)

Aldehyde 30

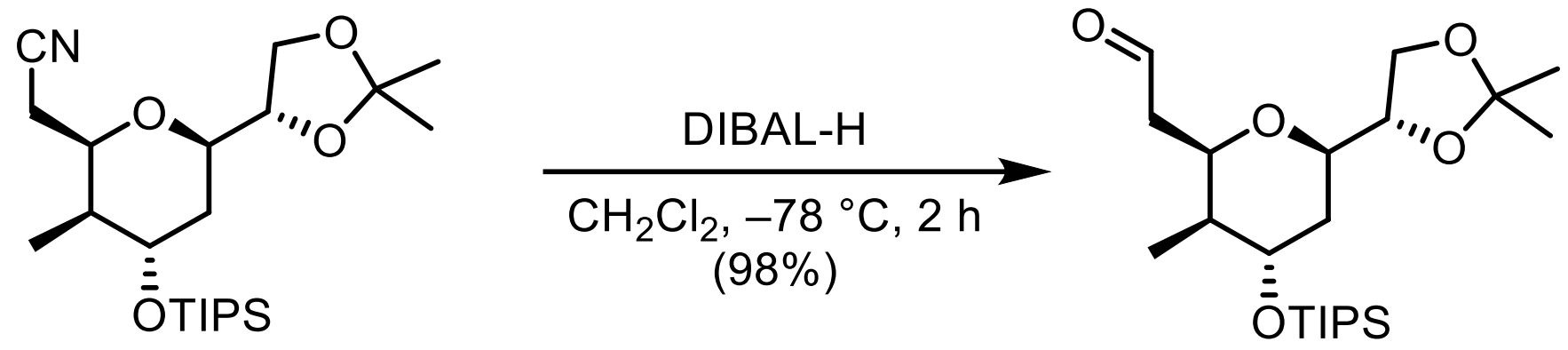

12 **30**

Nitrile **12** (295 mg, 0.72 mmol, 1.0 eq.) was dissolved in 10 mL dry toluene and cooled to –78 °C. DIBAL-H (1M in CH_2Cl_2, 0.93 mL, 0.93 mmol, 1.3 eq.) was added dropwise and the reaction mixture was warmed up to –20 °C and stirred for 2 h. After completion of the reaction, dry MeOH (12 mL) was added dropwise, and the mixture was warmed to r.t. Subsequently, sat. Seignette salt solution (30 mL) and CH_2Cl_2 (50 mL) were added and the resulting gel-like mixture was vigorously stirred for 1 h. The layers were separated, and the aqueous layer was extracted with CH_2Cl_2 (2 x 30 mL). The combined organic layer was washed with sat. NaCl solution and dried over Na_2SO_4. After removal of the solvent under reduced pressure, the crude product was purified by flash chromatography (pentane/Et_2O, 4:1) to yield aldehyde **30** (290 mg, 700 µmol, 98%) as a colorless liquid.

$\mathbf{R_f}$ = 0.38 (pentane/Et_2O, 4:1), vanillin: grey, UV (-).

^{1}H NMR (600 MHz, $CDCl_3$) δ [ppm] = 9.77 (s, 1H), 4.52 (dt, *J* = 10.1, 3.1 Hz, 1H), 4.04 – 3.97 (m, 2H), 3.95 – 3.86 (m, 2H), 3.79 (ddd, *J* = 11.7, 6.8, 2.2 Hz, 1H), 2.59 (ddd, *J* = 16.3, 10.1, 2.7 Hz, 1H), 2.24 (dt, *J* = 16.2, 1.2 Hz, 1H), 1.76 – 1.70 (m, 1H), 1.68 – 1.60 (m, 1H), 1.57 (ddd, *J* = 13.9, 11.6, 2.5 Hz, 1H), 1.38 (s, 3H), 1.33 (s, 3H), 1.09 – 1.03 (m, 21H), 0.89 (d, *J* = 7.2 Hz, 3H).

^{13}C NMR (151 MHz, $CDCl_3$) δ [ppm] = 201.8, 109.5, 78.1, 73.8, 70.6, 69.7, 66.9, 46.8, 39.2, 30.9, 26.7, 25.4, 18.2, 12.3, 11.3.

IR (ATR) ṽ (cm^{-1}) = 2961 (m), 2941 (m), 2890 (m), 2867 (m), 1462 (m), 1380 (m), 1253 (m), 1216 (m), 1147 (m), 1066 (s), 1013 (m), 996 (m), 945 (w), 881 (s), 847 (m), 818 (w), 734 (w), 717 (w), 679 (s), 657 (m), 506 (w).

HRMS (ESI) (*m/z*) calcd. for $[C_{22}H_{42}O_5SiH]^+$ = $[M+H]^+$: 415.2880, found: 415.2879.

Specific Rotation $[\alpha]_{589}^{21}$= +14.0° (c = 0.41, $CHCl_3$).

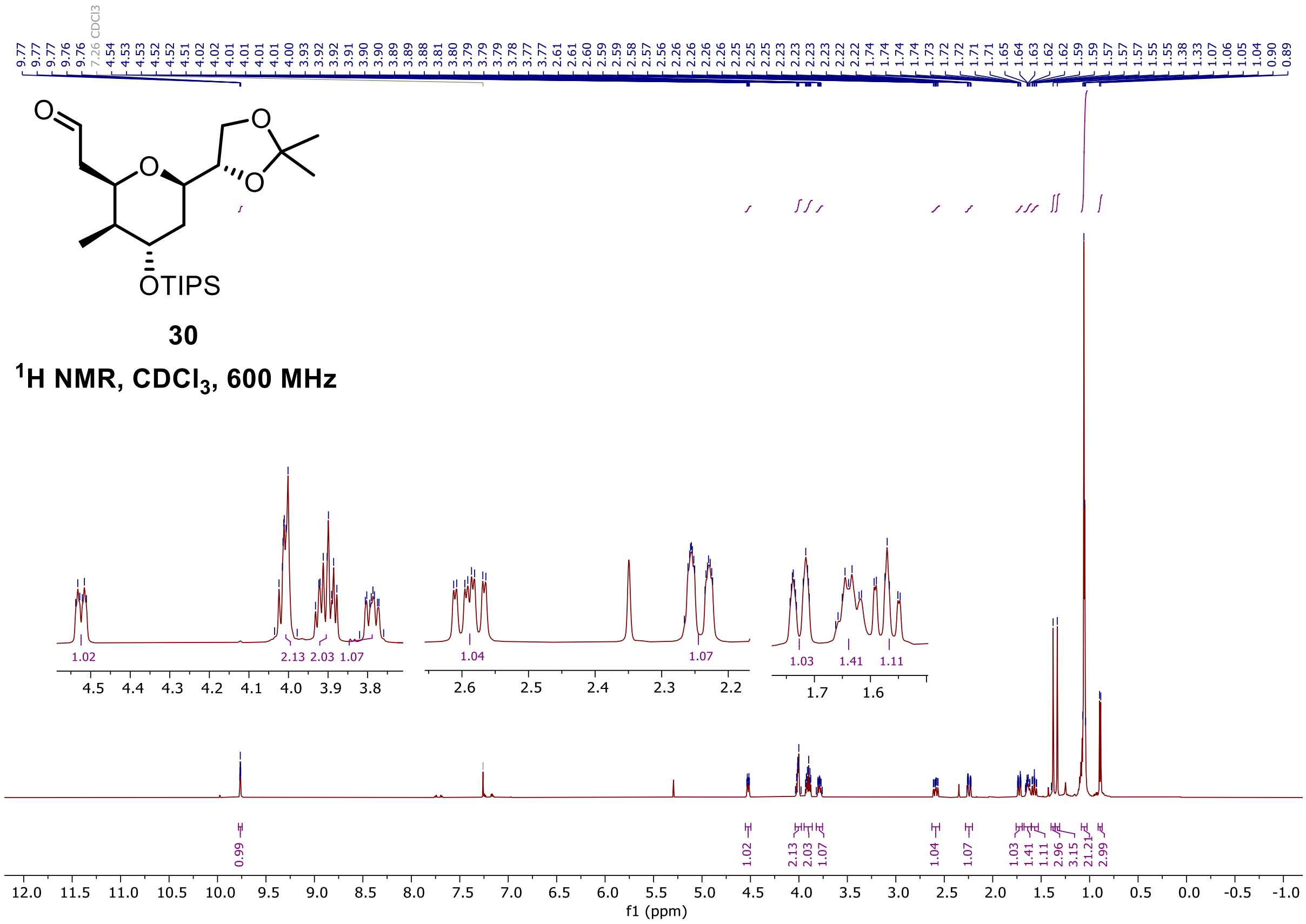

30

^{1}H NMR, $CDCl_3$, 600 MHz

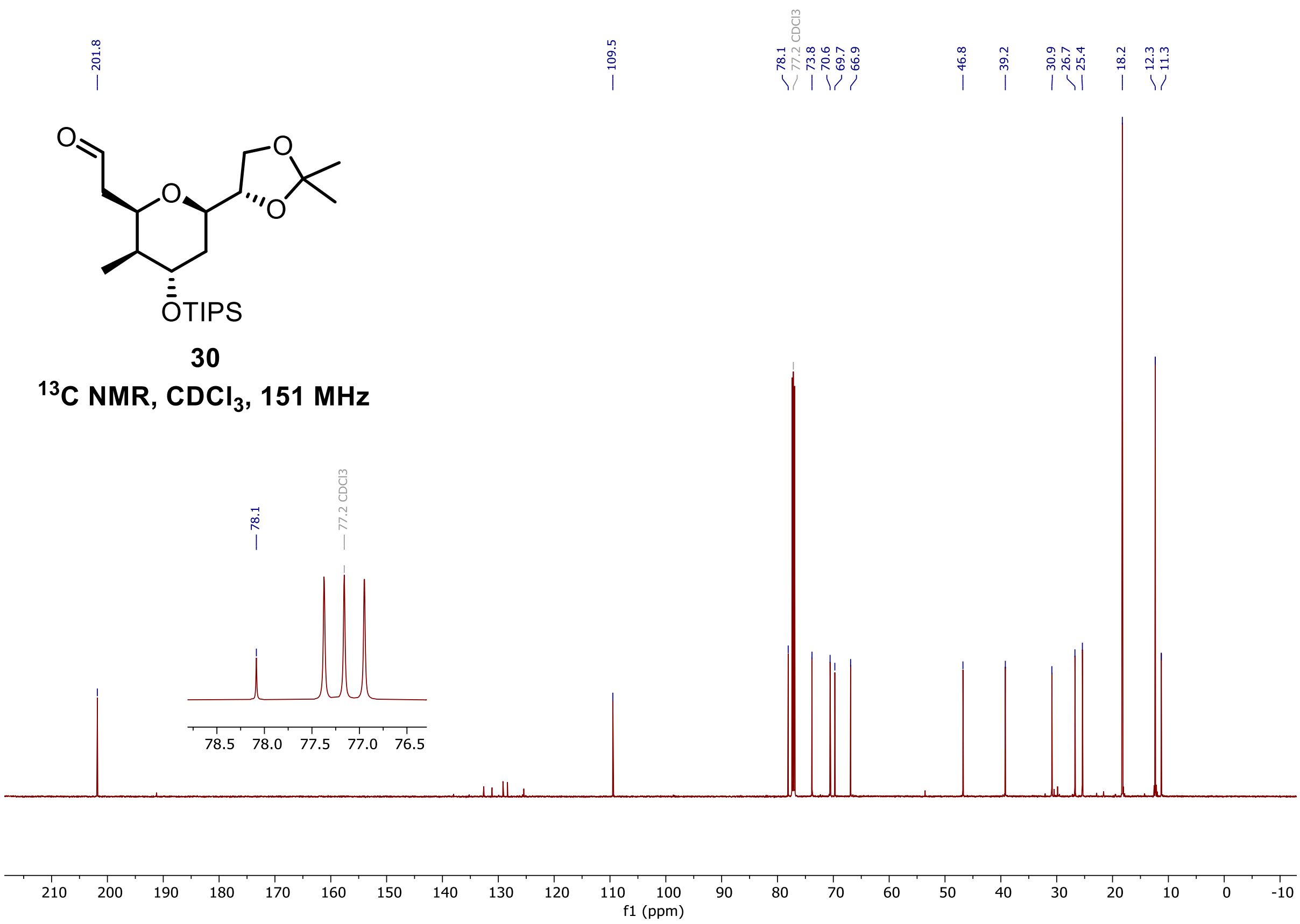

30

^{13}C NMR, $CDCl_3$, 151 MHz

Alkene 31

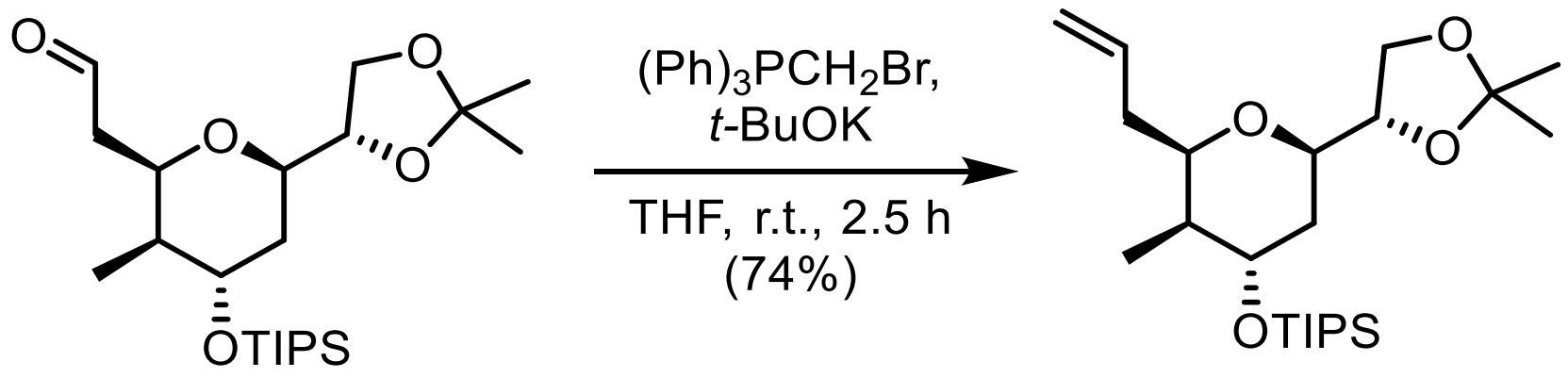

30 **31**

Pre-dried methyltriphenylphosphonium bromide (725 mg, 2.03 mmol, 3.0 eq.) was dissolved in 20 mL dry THF. Next, *t*-BuOK (213 mg, 1.90 mmol, 2.8 eq.), dissolved in 5 mL dry THF, was slowly added and the resulting mixture was stirred for 2 h at r.t. Then, aldehyde **30** (279 mg, 0.67 mmol, 1.0 eq.), dissolved in 5 mL dry THF, was added dropwise to the yellow solution. The reaction mixture was stirred for additional 30 min, before it was quenched with sat. NH_4Cl solution (30 mL). The phases were separated, and the aqueous phase was extracted with Et_2O (2 x 30 mL). The combined organic phase was washed with sat. NaCl solution, dried over Na_2SO_4 and concentrated under reduced pressure. The crude material was purified by flash chromatography (pentane/Et_2O, 15:1) to provide alkene **31** (205 mg, 0.50 mmol, 74%) as a colorless oil.

$\mathbf{R_f}$ = 0.66 (pentane/Et_2O, 9:1), vanillin: blue, UV (-).

^{1}H NMR (600 MHz, $CDCl_3$) δ [ppm] = 5.82 – 5.72 (m, 1H), 5.11 – 4.98 (m, 2H), 4.04 (dd, *J* = 8.4, 6.4 Hz, 1H), 4.00 – 3.90 (m, 4H), 3.71 (ddd, *J* = 11.6, 5.2, 2.2 Hz, 1H), 2.33 – 2.25 (m, 1H), 2.04 (dddt, *J* = 14.3, 7.8, 6.6, 1.2 Hz, 1H), 1.70 (dddd, *J* = 13.6, 3.4, 2.3, 1.3 Hz, 1H), 1.62 (qtd, *J* = 7.0, 2.6, 1.2 Hz, 1H), 1.55 (ddd, *J* = 13.9, 11.6, 2.6 Hz, 1H), 1.39 (s, 3H), 1.34 (s, 3H), 1.11 – 1.00 (m, 21H), 0.86 (d, *J* = 7.2 Hz, 3H).

^{13}C NMR (151 MHz, $CDCl_3$) δ [ppm] = 135.3, 116.4, 109.4, 78.4, 73.9, 73.7, 70.9, 67.0, 38.5, 37.1, 31.4, 26.8, 25.5, 18.2, 12.4, 10.9.

IR (ATR) ṽ (cm^{-1}) = 2924 (m), 2866 (m), 1463 (w), 1433 (w), 1380 (w), 1369 (w), 1254 (w), 1216 (w), 1068 (s), 995 (m), 914 (w), 882 (m), 846 (m), 819 (w), 742 (m), 679 (s), 501 (m).

HRMS (ESI) (*m/z*) calcd. for $[C_{23}H_{44}O_4SiH]^+$ = $[M+H]^+$: 413.3087, found: 413.3086.

Specific Rotation $[\alpha]^{21}_{589}$= +14.0° (c = 0.5, $CHCl_3$).

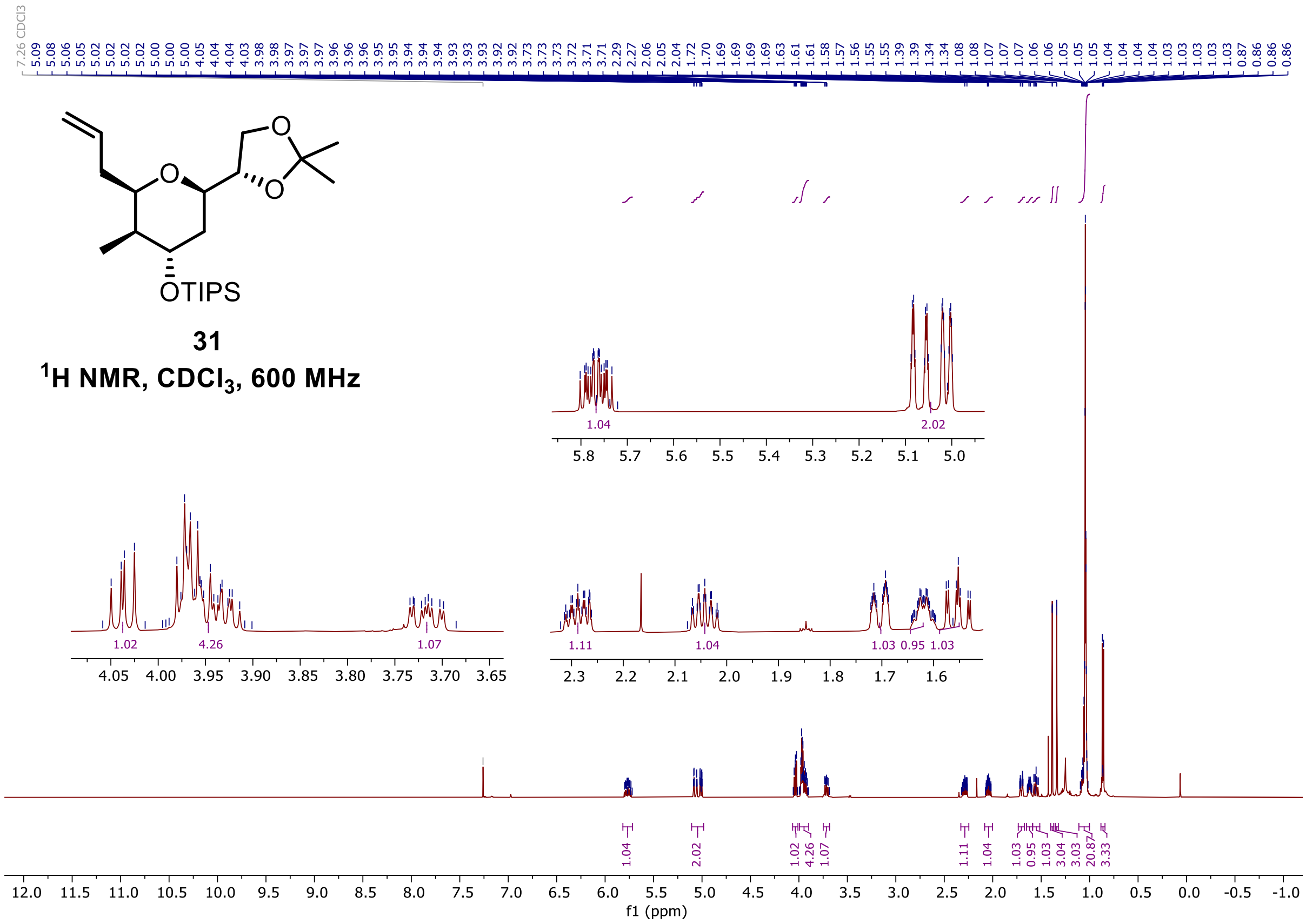

31

^{1}H NMR, $CDCl_3$, 600 MHz

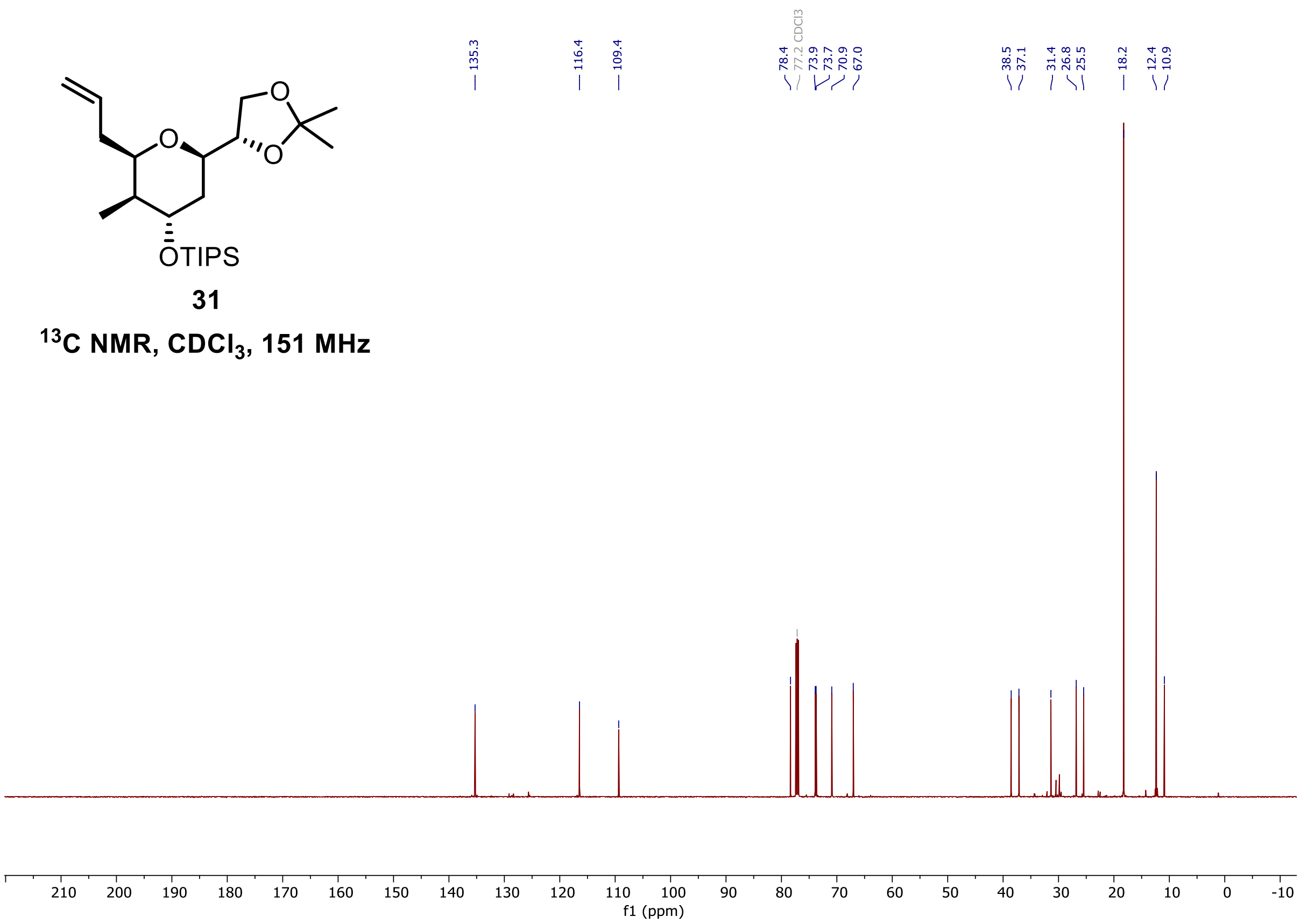

31

^{13}C NMR, $CDCl_3$, 151 MHz

Diol 32

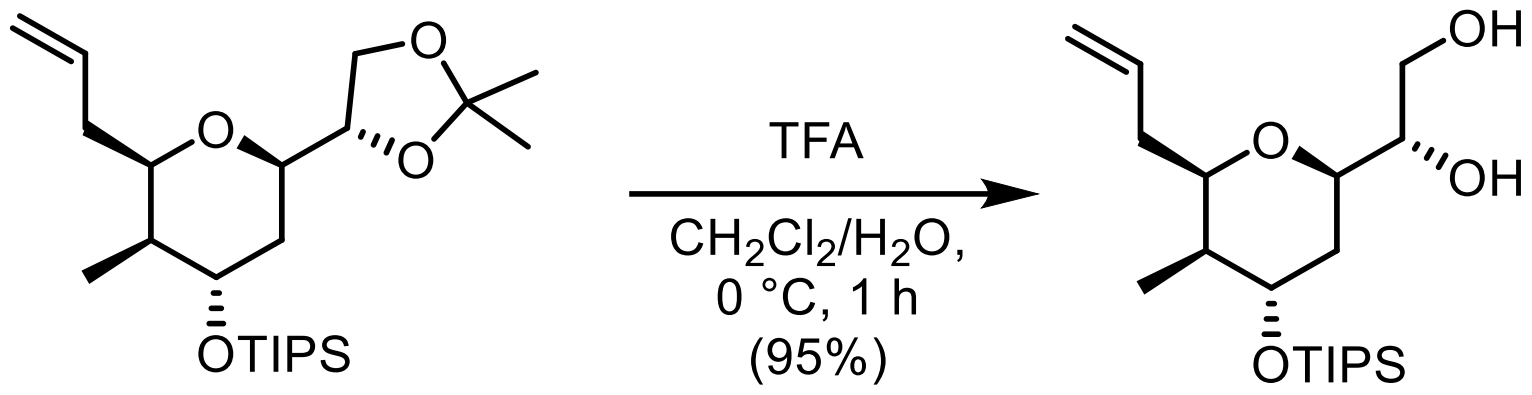

31 **32**

Alkene **31** (500 mg, 1.21 mmol, 1.0 eq.) was dissolved in 20 mL CH_2Cl_2, 0.85 mL water, and the resulting mixture was cooled to 0 °C. Then, pre-cooled TFA (0.85 mL) was added and the reaction mixture was stirred for 1 h at 0 °C. After completion, sat. $NaHCO_3$ solution (30 mL) was added and the reaction warmed up to r.t. The mixture was further diluted with CH_2Cl_2 and water, before the layers were separated. The aqueous layer was extracted with CH_2Cl_2 (2 x 30 mL), the organic layer was dried over Na_2SO_4, filtered and concentrated in vacuo. The crude material can be used without further purification, however for analytical reasons, it was purified by flash chromatography (pentane/Et_2O, 1:1) to afford diol **32** (430 mg, 1.15 mmol, 95%) as a colorless, highly viscous oil.

$\mathbf{R_f}$ = 0.20 (pentane/Et_2O, 1:1), vanillin: blue, UV (-).

^{1}**H NMR** (600 MHz, $CDCl_3$) δ [ppm] = 5.80 – 5.70 (m, 1H), 5.11 – 4.98 (m, 2H), 4.01 – 3.93 (m, 3H), 3.75 (dd, *J* = 11.4, 5.2 Hz, 1H), 3.67 (dd, *J* = 11.4, 4.0 Hz, 1H), 3.57 (dt, *J* = 5.2, 4.1 Hz, 1H), 2.28 (dddt, *J* = 14.4, 8.0, 6.4, 1.5 Hz, 1H), 2.10 – 2.01 (m, 1H), 1.73 – 1.59 (m, 2H), 1.53 – 1.45 (m, 1H), 1.12 – 0.99 (m, 21H), 0.86 (d, *J* = 7.2, 3H).

^{13}**C NMR** (151 MHz, $CDCl_3$) δ [ppm] = 135.0, 116.9, 75.4, 74.0, 73.5, 70.8, 63.9, 38.5, 37.2, 30.5, 18.2, 12.3, 10.9.

IR (ATR) ṽ (cm^{-1}) = 3420 (br), 2941 (m), 2866 (m), 1642 (w), 1463 (m), 1382 (w), 1247 (w), 1090 (s), 1054 (s), 994 (m), 913 (m), 881 (s), 819 (w), 726 (m), 678 (s), 654 (s), 502 (w).

HRMS (ESI) (*m/z*) calcd. for $[C_{20}H_{40}O_4SiH]^+$ = $[M+H]^+$: 373.2774, found: 373.2783.

Specific Rotation $[\alpha]_{589}^{25}$ = +20.0° (c = 0.8, $CHCl_3$).

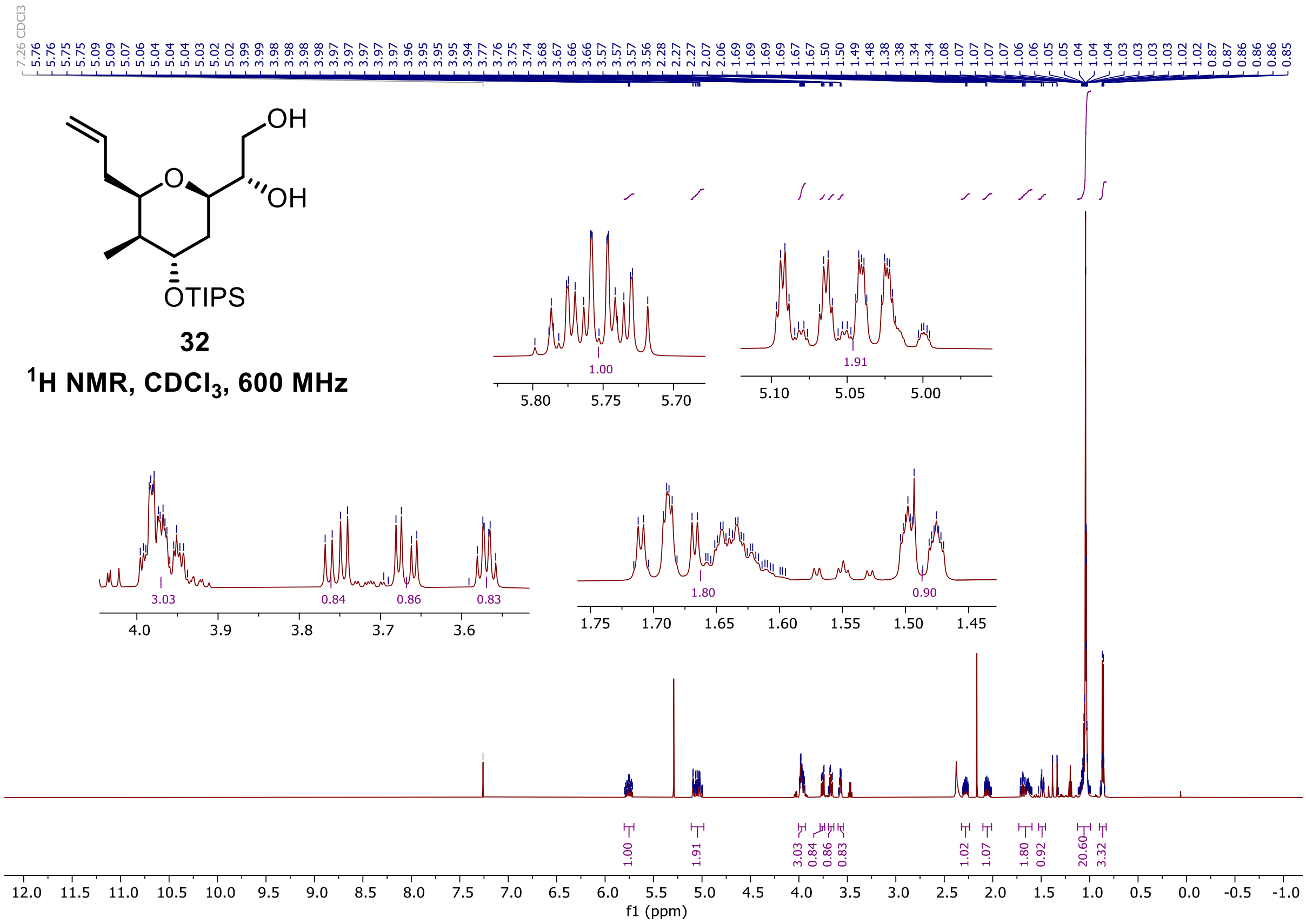
OH
OH
O
OTIPS
32
^{1}H NMR, $CDCl_3$, 600 MHz
f1 (ppm)

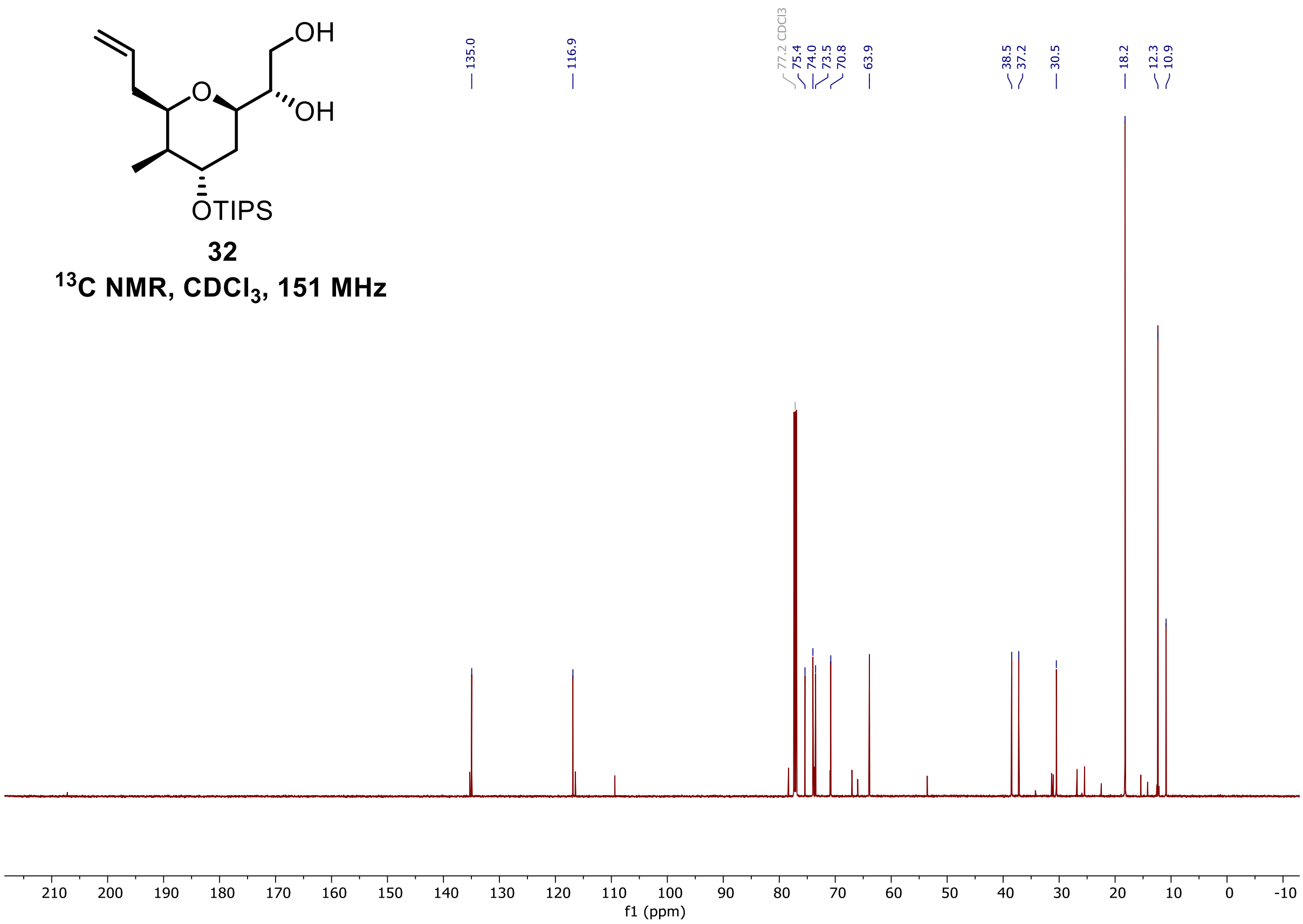
OH
OH
O
OTIPS
32
^{13}C NMR, $CDCl_3$, 151 MHz
135.0
116.9
77.2 CDCl3
75.4
74.0
73.5
70.8
63.9
38.5
37.2
30.5
18.2
12.3
10.9
f1 (ppm)

Silylether 6 (THP fragment)

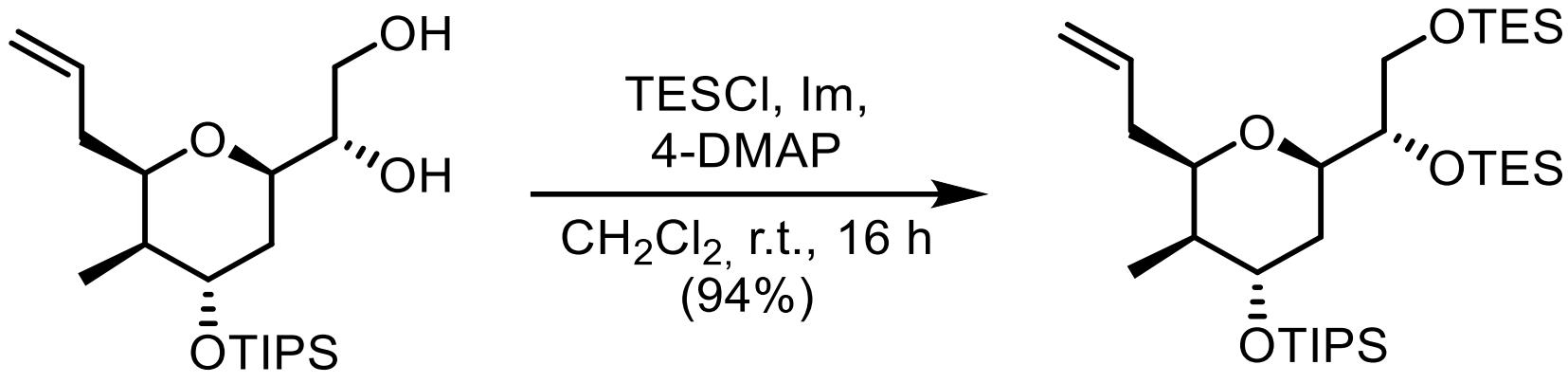

32 **6**

A round-bottom flask was charged with diol **32** (430 mg, 1.15 mmol, 1.0 eq.) and 20 mL dry CH_2Cl_2. Subsequently, 4-DMAP (7 mg, 60 µmol, 5 mol%), imidazole (314 mg, 4.6 mmol, 4.0 eq.) and TESCl (purity 95%, 0.45 mL, 2.55 mmol, 2.2 eq.) were added and the reaction mixture was stirred for 16 h at r.t. Then, sat. NH_4Cl solution (50 mL) was added dropwise, and the layers were separated. The aqueous phase was extracted with CH_2Cl_2 (2 x 30 mL), dried over Na_2SO_4 and concentrated under reduced pressure. The crude product was purified by flash chromatography (pentane) to yield silylether **6** (650 mg, 1.08 mmol, 94%) as a colorless oil.

$\mathbf{R_f}$ = 0.25 (pentane/Et_2O, 50:1), vanillin: dark blue, UV (-).

^{1}H NMR (400 MHz, $CDCl_3$) δ [ppm] = 5.86 – 5.71 (m, 1H), 5.11 – 4.96 (m, 2H), 3.97 (q, *J* = 2.9 Hz, 1H), 3.93 (ddd, *J* = 7.7, 6.6, 2.3 Hz, 1H), 3.79 (ddd, *J* = 11.6, 4.9, 2.3 Hz, 1H), 3.69 (q, *J* = 5.2 Hz, 1H), 3.61 (dd, *J* = 10.1, 5.1 Hz, 1H), 3.47 (dd, *J* = 10.1, 6.0 Hz, 1H), 2.35 – 2.19 (m, 1H), 2.03 (dddt, *J* = 14.2, 7.8, 6.5, 1.2 Hz, 1H), 1.69 (ddd, *J* = 14.0, 11.6, 2.6 Hz, 1H), 1.62 – 1.47 (m, 2H), 1.04 (d, *J* = 3.0 Hz, 21H), 0.97 – 0.89 (m, 18H), 0.86 (d, *J* = 7.2 Hz, 3H), 0.65 – 0.49 (m, 12H).

^{13}C NMR (101 MHz, $CDCl_3$) δ [ppm] = 135.8, 116.1, 76.2, 73.6, 72.9, 71.4, 64.8, 38.6, 37.4, 29.2, 18.3, 12.4, 10.9, 7.1, 6.9, 6.6, 5.2, 4.5.

IR (ATR) ṽ (cm^{-1}) = 2954 (m), 2912 (m), 2875 (m), 1461 (w), 1414 (w), 1380 (w), 1238 (w), 1143 (m), 1073 (s), 1004 (s), 912 (w), 882 (m), 835 (w), 803 (m), 726 (s), 678 (s).

HRMS (ESI) (*m/z*) calcd. for $[C_{32}H_{68}O_4Si_3H]^+$ = $[M+H]^+$: 601.4504, found: 601.4509.

Specific Rotation $[\alpha]_{589}^{20}$= +15.1° (c = 0.86, $CHCl_3$).

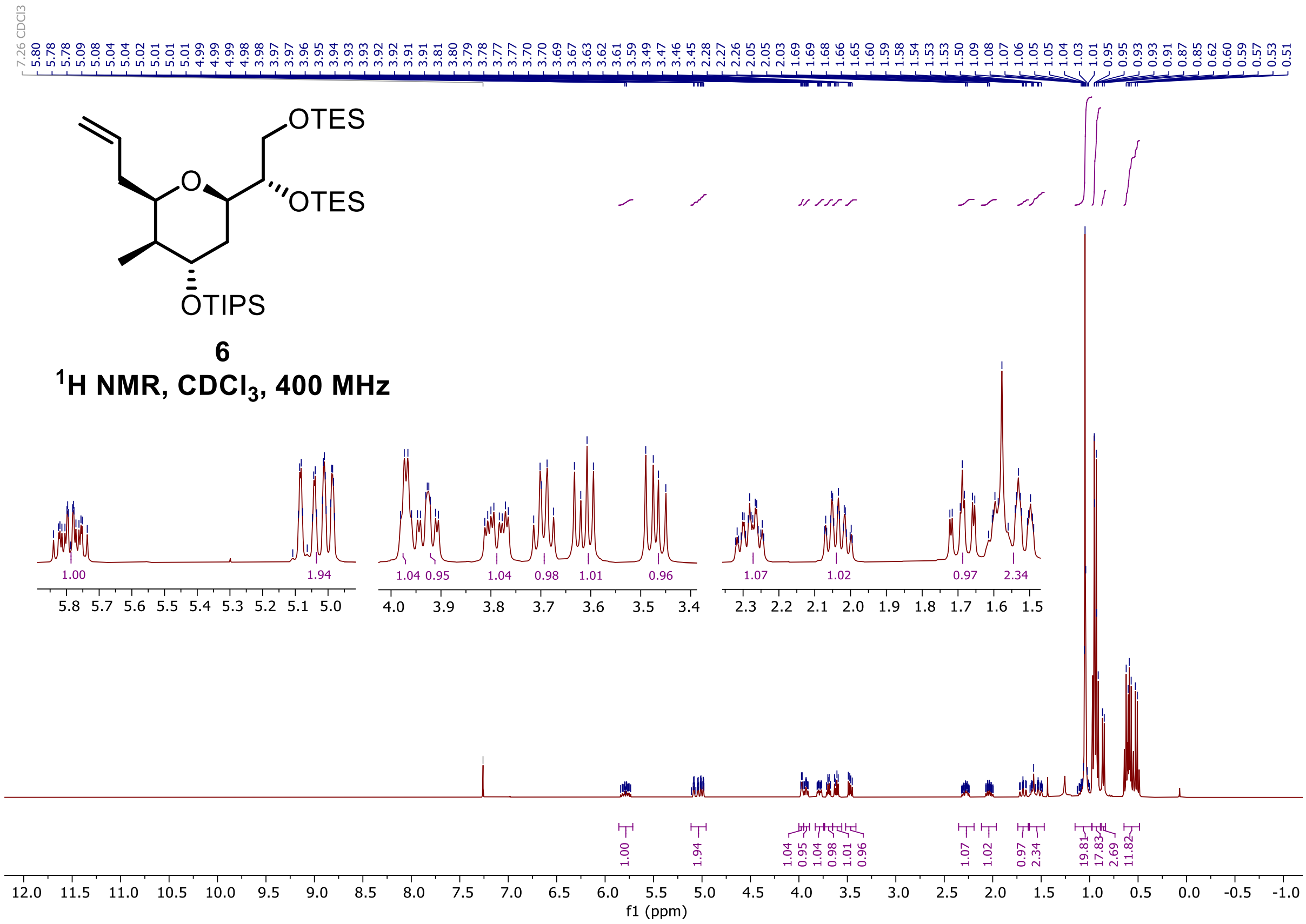
OTES
OTES
O
OTIPS
6
¹H NMR, CDCl₃, 400 MHz
7.26 CDCl3
1.00 1.94 1.04 0.95 1.04 0.98 1.01 0.96 1.07 1.02 0.97 2.34 19.81 17.83 2.69 11.82
f1 (ppm)

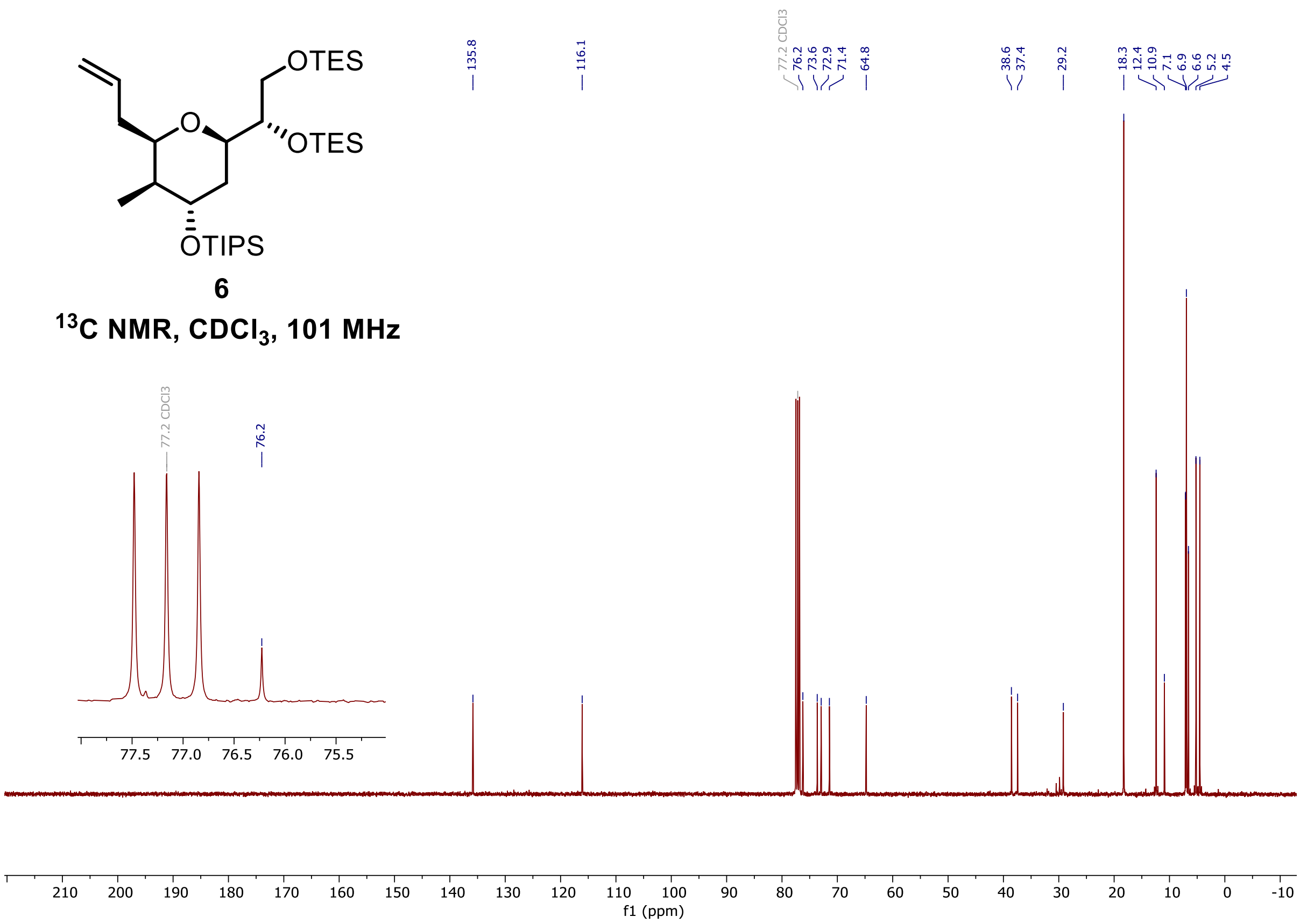
OTES
OTES
O
OTIPS
6
¹³C NMR, CDCl₃, 101 MHz
135.8
116.1
77.2 CDCl3
76.2
73.6
72.9
71.4
64.8
38.6
37.4
29.2
18.3
12.4
10.9
7.1
6.9
6.6
5.2
4.5
f1 (ppm)

Pentaacetate S4

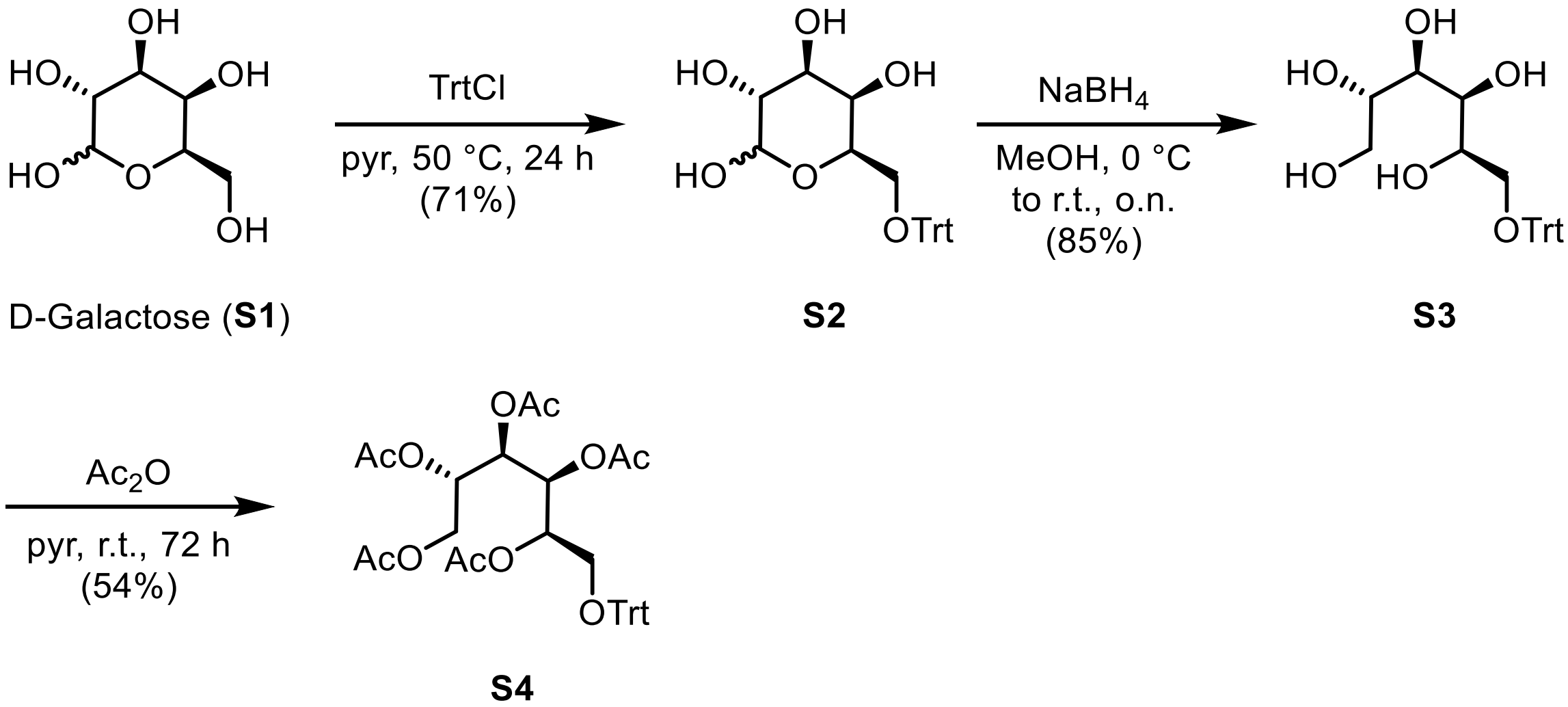

D-Galactose (**S1**) (145.4 g, 807.1 mmol, 1.5 eq.) was dissolved in 400 mL pyridine and heated to 50 °C before trityl chloride (150.0 g, 538.1 mol, 1.0 eq.) was added. The reaction was stirred for 24 h at 50 °C, cooled to r.t. and diluted with 750 mL 1-butanol. After washing the organic layer with water (3x) and sat. NaCl solution (3x), it was dried over Na_2SO_4 and concentrated to afford crude **S2** (243.93 g, 575.2 mmol, 71% based on D-galactose).
Compound **S2** (238.33 g, 564.1 mmol, 1.0 eq.) was dissolved in 730 mL dry MeOH and cooled to 0 °C. Next, $NaBH_4$ (31.9 g, 843.2 mmol, 1.5 eq.) was slowly added and the resulting mixture was stirred overnight at r.t. The mixture was quenched with 0.05M HCl (1 L), resulting in the formation of a precipitate. The latter was dissolved in EtOAc, washed again with 0.05M HCl (2x) and was further washed with sat. $NaHCO_3$ solution and sat. NaCl solution. After drying over Na_2SO_4 and removal of the solvent, the crude material was filtered through a short silica pad (10% MeOH in EtOAc) to obtain crude **S3** (202.32 g, 476.6 mmol, 85%).
Crude **S3** (181.6 g, 427.8 mmol, 1.0 eq.) was dissolved in 615 mL pyridine at r.t., before addition of acetic acid anhydride (615 mL, 6.51 mol, 15.2 eq.) and 4-DMAP (2.31 g, 18.9 mmol, 4 mol-%). The mixture was stirred at r.t. for 72 h before it was cooled to 0 °C and 700 mL MeOH were carefully added. The solvent was removed under reduced pressure and the crude was re-dissolved in 700 mL EtOAc. The organic phase was washed with 1N HCl (2x), sat. $NaHCO_3$ solution (2x), sat. NaCl solution (2x), dried over $MgSO_4$, filtered and concentrated under reduced pressure. The crude product was re-crystallized from warm EtOH (1.5 L) and left in the fridge overnight. After drying, compound **S4** (146.9 g, 231.5 mmol, 54%) was collected as a slightly yellow solid.

$\mathbf{R_f}$ = 0.66 (pentane/Et_2O, 1:2), $KMnO_4$, UV (+)

^{1}H NMR (600 MHz, $CDCl_3$) δ [ppm] = 7.43 – 7.27 (m, 14H), 7.25 – 7.17 (m, 5H), 5.43 (dd, *J* = 9.9, 2.1 Hz, 1H), 5.34 (ddd, *J* = 7.3, 5.9, 2.0 Hz, 1H), 5.31 (dd, *J* = 9.9, 2.0 Hz, 1H), 5.18 (ddd, *J* = 7.2, 5.0, 2.0 Hz, 1H), 4.25 (dd, *J* = 11.7, 5.0 Hz, 1H), 3.84 (dd, *J* = 11.6, 7.4 Hz, 1H), 3.11 (dd, *J* = 9.4, 7.3 Hz, 1H), 3.00 (dd, *J* = 9.5, 5.9 Hz, 1H), 2.13 (s, 3H), 2.08 (s, 3H), 2.07 (s, 3H), 2.01 (s, 3H), 1.81 (s, 3H).

^{13}C NMR (151 MHz, $CDCl_3$) δ [ppm] = 170.6, 170.4, 170.0, 169.4, 143.5, 128.8, 128.0, 127.3, 87.1, 69.0, 67.9, 67.7, 67.7, 62.3, 61.6, 21.0, 20.9, 20.8, 20.8, 20.5.

Alcohol S5

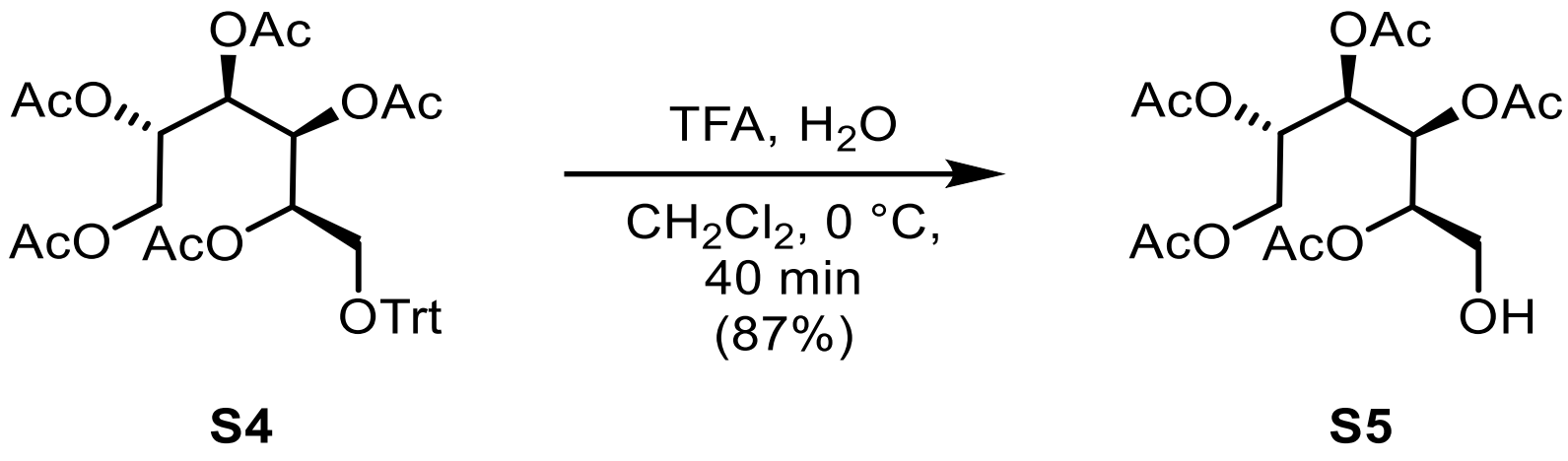

Compound **S4** (29.73 g, 46.8 mmol, 1.0 eq.) was dissolved in 150 mL CH_2Cl_2 and cooled to 0 °C. Next, water (9.0 mL, 500 mmol, 10.7 eq.) was added, followed by the addition of trifluoroacetic acid (45.0 mL, 684 mmol, 14.6 eq.) over a period of 20 min. The resulting mixture was stirred for 40 min at 0 °C (TLC control with pure Et_2O), before it was diluted with EtOAc (850 mL), washed with water (150 mL), sat. $NaHCO_3$ solution (2 x 300 mL) and sat. NaCl solution (300 mL). The combined organic layer was dried over Na_2SO_4, filtered and concentrated in vacuo. The crude material was purified by flash column chromatography (EtOAc/Et_2O, 1:1) to afford **S5** (15.97 g, 40.7 mmol, 87%) as a white solid.

$\mathbf{R_f}$ = 0.48 (Et_2O), $KMnO_4$, UV (-)

^{1}H NMR (400 MHz, $CDCl_3$) δ [ppm] = 5.41 – 5.32 (m, 2H), 5.25 (dd, *J* = 9.9, 1.7 Hz, 1H), 5.01 (ddd, *J* = 7.8, 6.0, 1.7 Hz, 1H), 4.26 (dd, *J* = 11.7, 4.5 Hz, 1H), 3.84 (dd, *J* = 11.7, 7.8 Hz, 1H), 3.55 (ddd, *J* = 11.8, 8.9, 6.1 Hz, 1H), 3.36 (ddd, *J* = 12.1, 6.2, 3.4 Hz, 1H), 2.65 (dd, *J* = 9.1, 5.0 Hz, 1H), 2.13 (s, 3H), 2.09 (s, 3H), 2.05 (s, 6H), 2.00 (s, 3H).

^{13}C NMR (101 MHz, $CDCl_3$) δ [ppm] = 171.8, 170.6 (2C), 170.4, 169.8, 69.9, 67.9, 67.8, 67.4, 62.4, 59.8, 20.9, 20.8, 20.7 (2C), 20.6.

Aldehyde S6

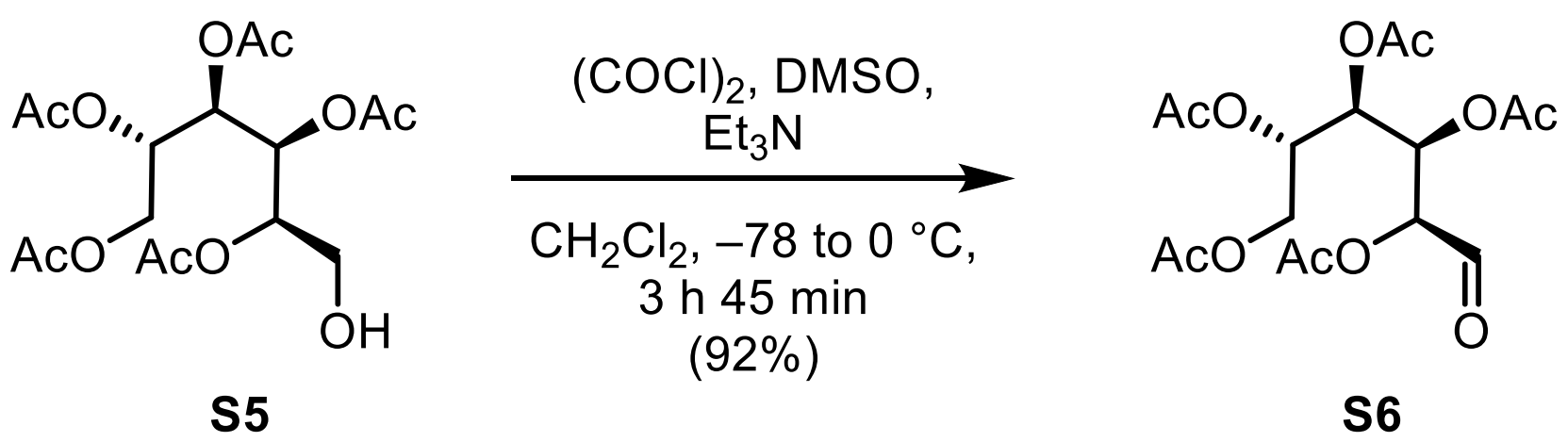

A three-neck flask was subjected with oxalyl chloride (2.30 mL, 26.8 mmol, 1.5 eq.), dissolved in 140 mL dry CH_2Cl_2 and cooled to –78 °C. Next, a solution of DMSO (2.60 mL, 36.6 mmol, 2.0 eq.) in 28 mL dry CH_2Cl_2 was added and the mixture was stirred for 45 min at –78 °C. Subsequently, alcohol **S5** (7.09 g, 18.1 mmol, 1.0 eq.), diluted in 70 mL dry CH_2Cl_2, was added, while keeping the internal temperature below –60 °C. After stirring for 1 h at –78 °C, Et_3N (17.5 mL, 126.2 mmol, 7.0 eq.) was added and the mixture was stirred for 2 h while warming to 0 °C. The mixture was quenched with 1N HCl (75 mL), the layers were separated and the organic layer was washed with 1N HCl (2 x 75 mL), sat. $NaHCO_3$ solution (2 x 75 mL) and sat. NaCl solution (75 mL). The organic layer was dried over Na_2SO_4, filtered and concentrated in

vacuo to yield aldehyde **S6** as a beige solid (6.50 g, 16.7 mmol, 92%), which was used without further purification.

R_f = 0.25 (Et_2O), $KMnO_4$, UV (-)

^{1}H NMR (400 MHz, $CDCl_3$) δ [ppm] = 2.03 (s, 3H), 2.04 (s, 3H), 2.10 (s, 3H), 2.11 (s, 3H), 2.21 (s, 3H), 3.90 (dd, *J* = 11.6, 7.4 Hz, 1H), 4.27 (dd, *J* = 11.6, 5.2 Hz, 1H), 5.26 (d, *J* = 1.9 Hz, 1H), 5.36 (ddd, *J* = 7.3, 5.1, 2.0 Hz, 1H), 5.47 (dd, *J* = 9.9, 2.0 Hz, 1H), 5.64 (dd, *J* = 9.9, 1.9 Hz, 1H), 9.45 (s, 1H).

^{13}C NMR (101 MHz, $CDCl_3$) δ [ppm] = δ 20.3, 20.5, 20.5, 20.6, 20.7, 61.9, 66.2, 67.4, 67.5, 75.7, 126.5, 127.3, 127.9, 127.9, 129.6, 169.4, 169.7, 170.0, 170.1, 170.4, 193.4.

L-Galactose (19)

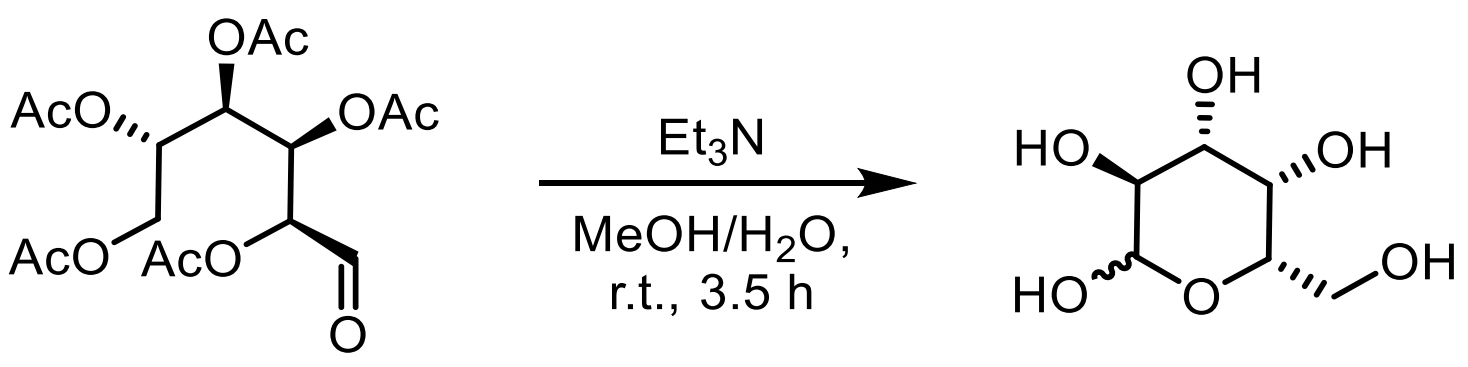

Aldehyde **S6** (6.50 g, 16.7 mmol, 1.0 eq.) was dissolved in 60 mL dry MeOH at r.t., before Et_3N (14.4 mL, 103.2 mmol, 6.2 eq.) and water (72 mL) were added. The initial formed white precipitate gets dissolved over time, indicating the progress of the reaction. After 3.5 h at r.t., the reaction was washed with EtOAc (3 x 75 mL) and the aqueous layer, containing the product, was concentrated in vacuo (water bath: 50 °C) to afford the crude L-galactose (**19**) as a brown oil (5.19 g). The material was used without any further purification.

R_f = 0.35 (EtOAc/MeOH/H_2O, 5:5:2), $KMnO_4$, UV (-)

^{1}H NMR (600 MHz, D_2O) δ [ppm] = 5.26 (d, *J* = 3.8 Hz, 1H), 4.58 (d, *J* = 7.9 Hz, 2H), 4.11 – 4.07 (m, 1H), 3.98 (dd, *J* = 3.4, 1.2 Hz, 1H), 3.93 (dd, *J* = 3.5, 1.0 Hz, 2H), 3.87 – 3.78 (m, 3H), 3.78 – 3.67 (m, 0H), 3.64 (dd, *J* = 10.0, 3.6 Hz, 3H), 3.53 – 3.46 (m, 2H).

^{13}C NMR (151 MHz, D_2O) δ [ppm] = 98.2, 96.4, 92.2, 75.1, 72.8, 71.8, 70.4, 69.3, 69.1, 68.7, 68.3, 61.1, 60.9.

Pentaacetylated L-galactose S7

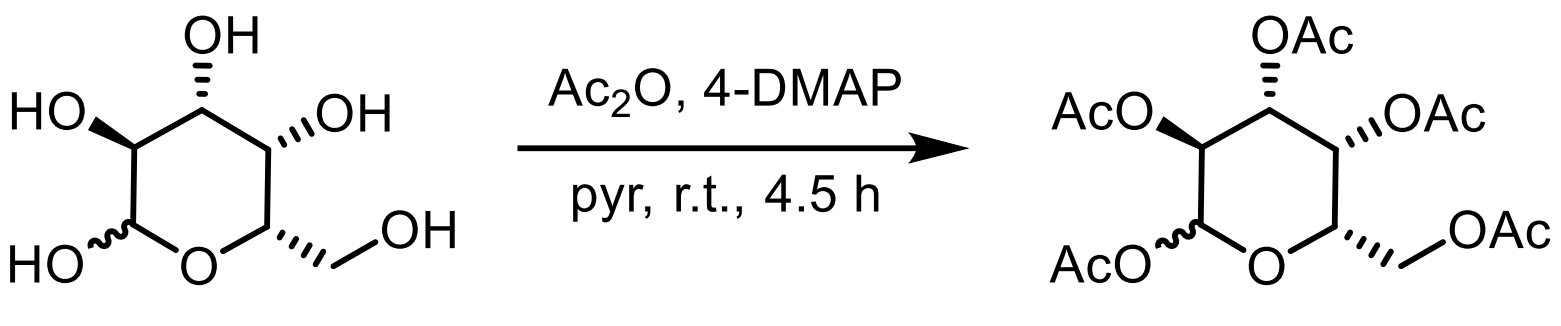

L-Galactose (1**9**) **S7**

The crude L-galactose (**19**) (calcd. 3.0 g, 16.7 mmol, 1.0 eq.) was dissolved in pyridine (16.0 mL, 198.2 mmol, 11.9 eq.) at r.t. and 4-DMAP (110 mg, 0.9 mmol, 6 mol-%) was added subsequently. After addition of Ac_2O (12.0 mL, 127.0 mmol, 7.6 eq.), the reaction was stirred for 4.5 h at r.t. Then, 18 mL MeOH and 80 mL EtOAc were added (exothermic!), the mixture was washed with water (50 mL), 1N HCl (3 x 50 mL), sat. $NaHCO_3$ solution (2 x 50 mL) and sat. NaCl solution (50 mL). The organic layer was dried over Na_2SO_4, filtered and concentrated to afford crude pentaacetylated L-galactose **S7** as a brown oil (6.50 g), which was used without further purification.

R_f = 0.96 (EtOAc/MeOH/H_2O, 5:5:2), $KMnO_4$, UV (-)

^{1}H NMR (600 MHz, $CDCl_3$) δ [ppm] = 6.39 – 6.36 (m, 1H), 6.32 (d, *J* = 4.6 Hz, 1H), 6.18 (s, 1H), 5.70 (d, *J* = 8.3 Hz, 2H), 5.55 (dd, *J* = 7.3, 6.3 Hz, 1H), 5.50 (q, *J* = 1.4 Hz, 1H), 5.42 (dd, *J* = 3.5, 1.2 Hz, 2H), 5.39 – 5.28 (m, 7H), 5.11 – 5.05 (m, 3H), 4.39 – 4.25 (m, 4H), 4.23 – 4.07 (m, 8H), 2.16 (s, 7H), 2.12 (s, 11H), 2.04 (s, 25H), 1.99 (s, 7H).

^{13}C NMR (151 MHz, $CDCl_3$) δ [ppm] = 171.3, 170.5, 170.5, 170.5, 170.3, 170.3, 170.1, 170.0, 169.5, 169.1, 99.2, 93.2, 92.3, 89.9, 82.3, 80.7, 79.2, 76.9, 76.5, 75.4, 73.5, 71.8, 71.0, 70.5, 69.4, 68.9, 67.9, 67.6, 67.5, 66.9, 66.6, 62.7, 62.2, 61.4, 61.2, 60.5, 21.2, 21.2, 21.0, 20.9, 20.8, 20.8, 20.8, 20.7, 14.3.

Bromide S8

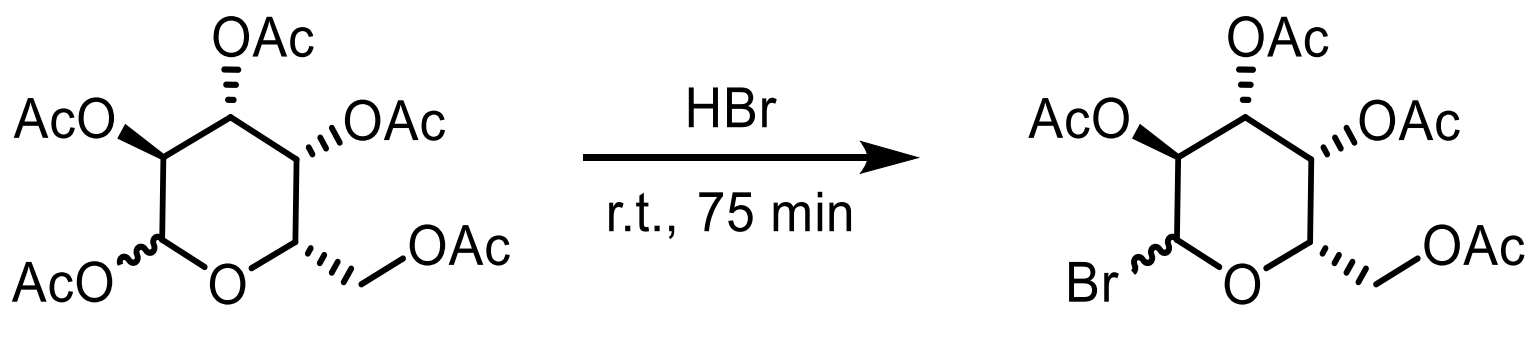

S7 **S8**

The crude compound **S7** (6.50 g) was treated with HBr (33% wt in AcOH, 18.0 mL, 99.1 mmol, 6.0 eq.) and the resulting mixture was stirred for 75 min at r.t. Then, CH_2Cl_2 (150 mL) was added and the organic layer was washed with water (75 mL), sat. $NaHCO_3$ solution (4 x 75 mL) and sat. NaCl solution (75 mL). The organic layer was dried over $MgSO_4$, filtered and concentrated to yield compound **S8** as a brown oil (4.0 g), which was used for the next step without any further purification.

R_f = 0.52 (pentane/Et_2O), $KMnO_4$, UV (-)

^{1}H NMR (400 MHz, $CDCl_3$) δ [ppm] = 6.69 (dd, *J* = 4.0, 0.6 Hz, 1H), 5.52 (dd, *J* = 3.3, 1.4 Hz, 1H), 5.40 (dd, *J* = 7.3, 3.6 Hz, 1H), 5.05 (dd, *J* = 10.6, 3.8 Hz, 1H), 4.52 – 4.45 (m, 1H), 4.44 – 4.31 (m, 1H), 4.22 – 4.15 (m, 1H), 4.14 – 4.07 (m, 1H), 2.15 (s, 3H), 2.11 (s, 3H), 2.06 (s, 3H), 2.01 (s, 3H).

^{13}C NMR (101 MHz, $CDCl_3$) δ [ppm] = 170.5, 170.2, 170.0, 169.9, 88.3, 71.2, 68.1, 67.9, 67.1, 61.0, 20.9, 20.9, 20.8, 20.7, 20.7.

Acetylated L-galactal 18

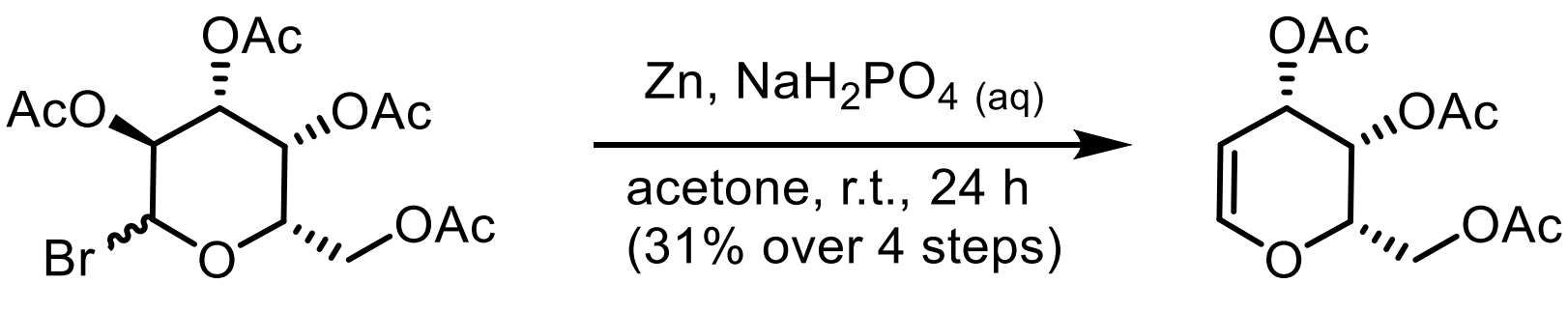

S8 **18**

The crude compound **S8** (4.0 g) was dissolved in 12.0 mL acetone and 6.0 mL sat. aq. NaH_2PO_4 solution at r.t. Next, zinc powder (8.0 g, 122.6 mmol) was added subsequently and stirring was continued at r.t. for 24 h. The mixture was filtered, washed with EtOAc (50 mL) and the organic layer was washed with water (25 mL), sat. $NaHCO_3$ solution (25 mL) and sat. NaCl solution (25 mL). The organic layer was dried over $MgSO_4$, filtered and concentrated in vacuo. After purification by flash column chromatography (CH_2Cl_2/MeOH, 100:1 → 80:1 → 70:1), acetylated L-galactal **18** (1.40 g, 5.14 mmol, 31% over 4 steps) was obtained as a slightly yellow oil.

$\mathbf{R_f}$ = 0.92 (CH_2Cl_2/MeOH, 20:1), vanillin: purple, UV (-)

^{1}H NMR (400 MHz, $CDCl_3$) δ [ppm] = 2.03 (s, 3H), 2.08 (s, 3H), 2.13 (s, 3H), 4.16 – 4.36 (m, 3H), 4.73 (ddd, *J* = 6.3, 2.7, 1.5 Hz, 1H), 5.43 (dt, *J* = 4.6, 1.7 Hz, 1H), 5.55 (dq, *J* = 5.6, 1.9 Hz, 1H), 6.46 (dd, *J* = 6.3, 1.8 Hz, 1H).

^{13}C NMR (101 MHz, $CDCl_3$) δ [ppm] = 170.7, 170.4, 170.3, 145.6, 99.0, 72.9, 64.0, 63.9, 62.1, 21.0, 20.9, 20.8.

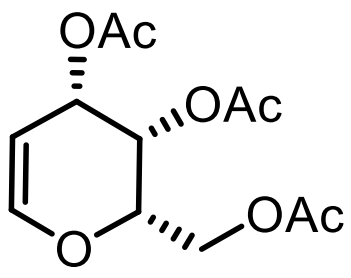

18

^{1}H NMR, $CDCl_3$, 400 MHz

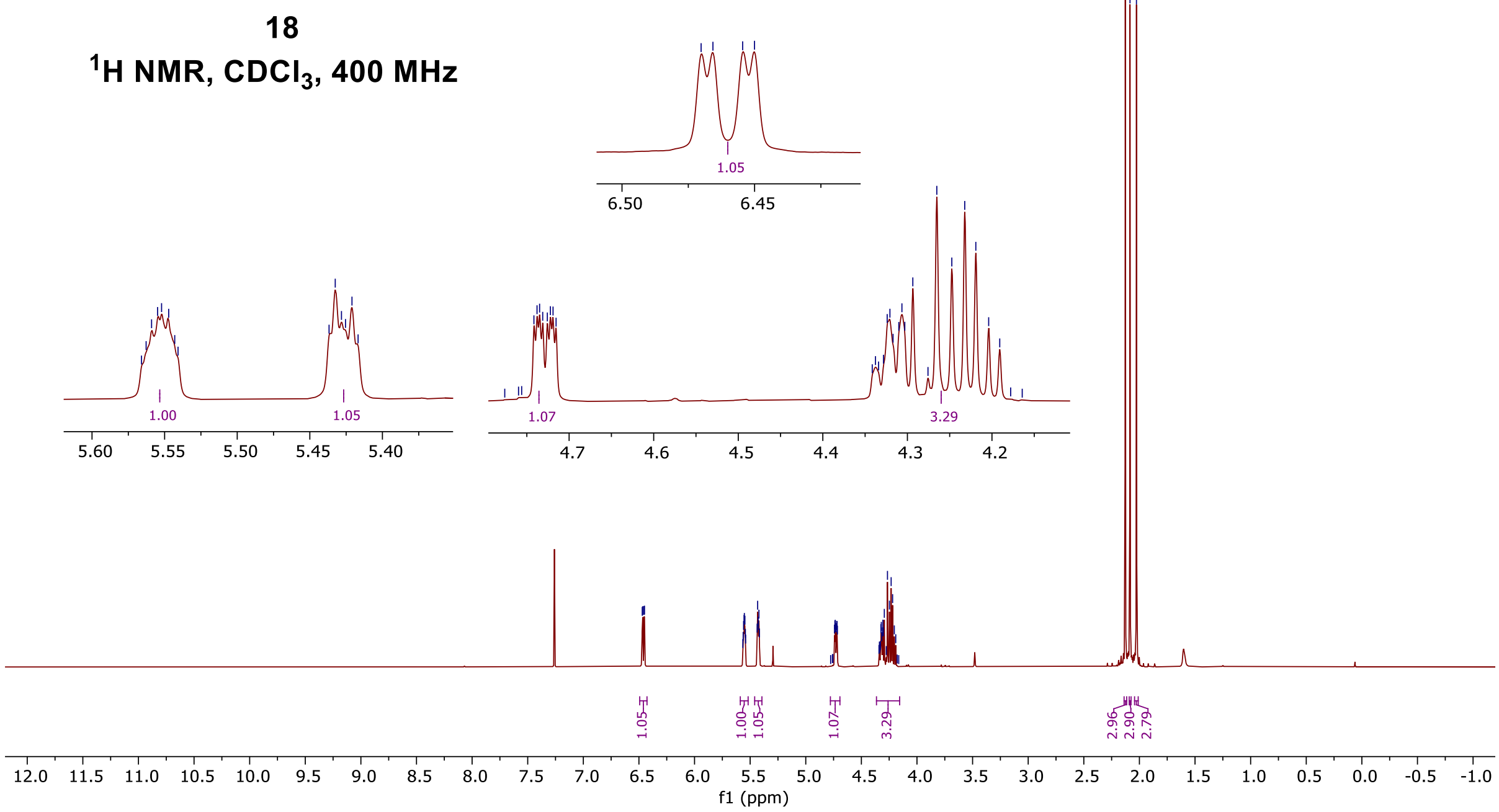

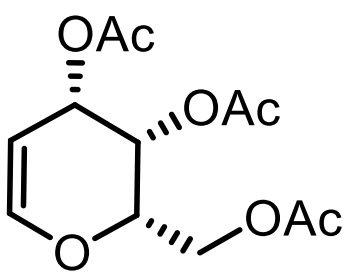

18

^{13}C NMR, $CDCl_3$, 101 MHz

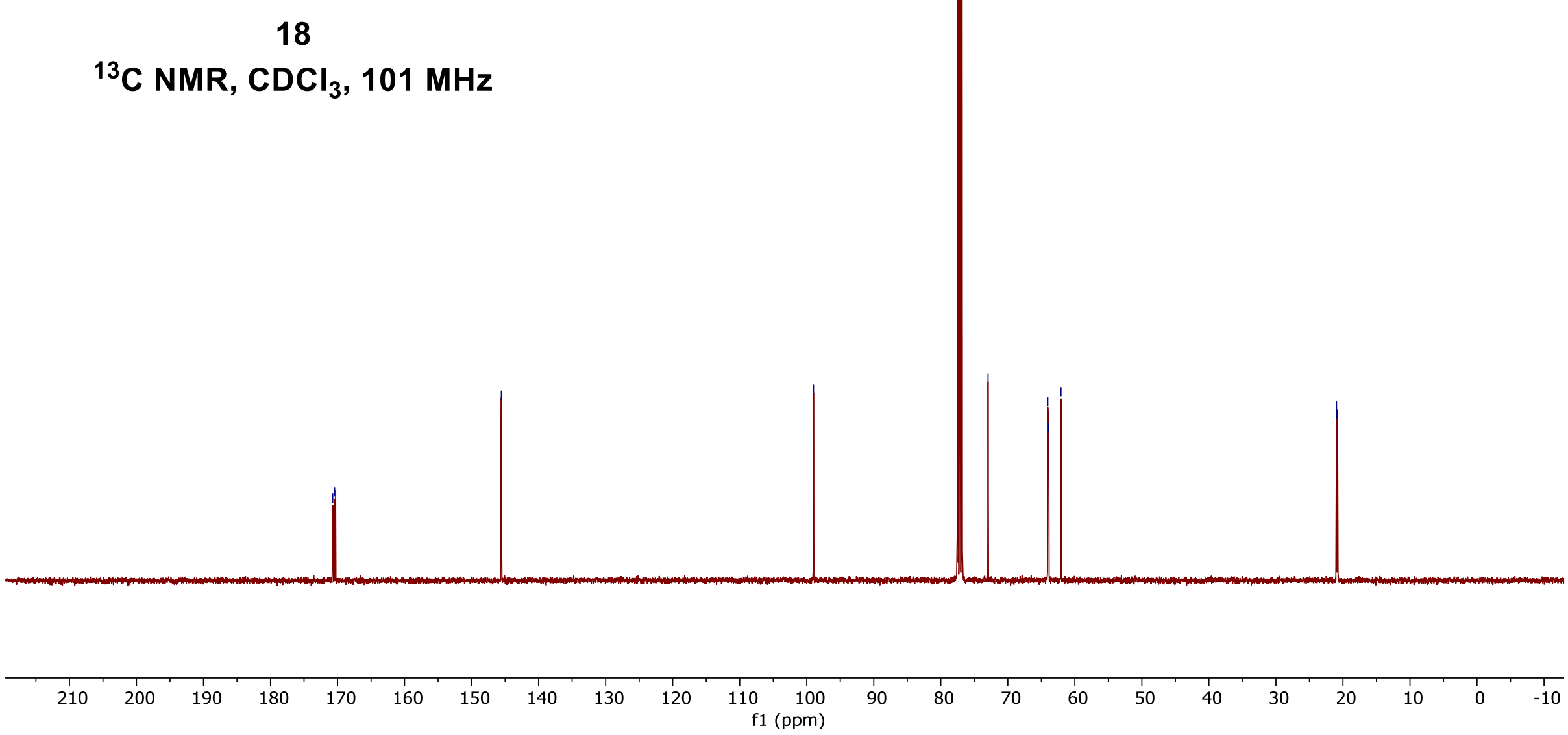

Diacetate 18a

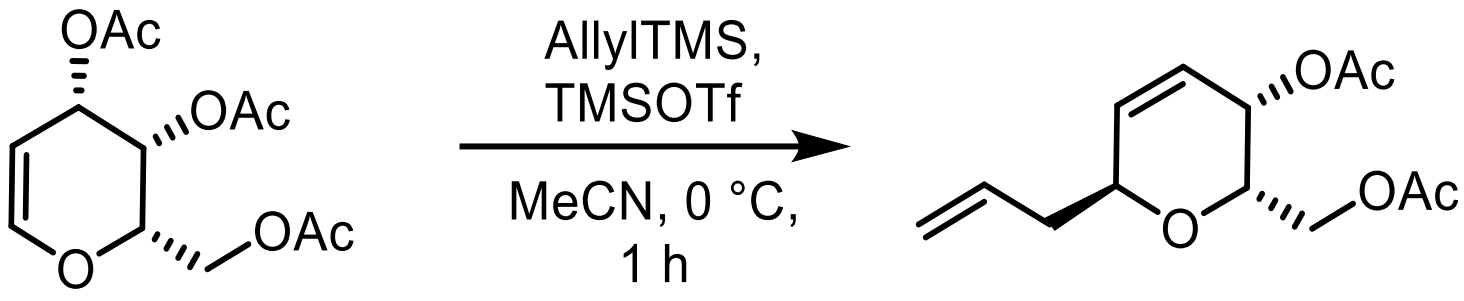

18 **18a**

A solution of L-galactal **18** (1.38 g, 5.07 mmol, 1.0 eq.) in 13 mL dry MeCN was cooled to 0°C and allyltrimethylsilane (1.20 mL, 7.55 mmol, 1.5 eq.) and trimethylsilyltriflate (1.10 mL, 6.08 mmol, 1.2 eq.) were added subsequently. After 1 h at 0 °C, aq. sat. $NaHCO_3$ solution (10 mL) was added and the mixture was extracted with EtOAc (3 x 25 mL). The combined organic layers were washed with sat. NaCl solution (25 mL), dried over $MgSO_4$, filtered and concentrated in vacuo. The crude product **18a** (brown-orange oil, 1.64 g) was used without further purification. However, an analytical sample was obtained by flash chromatography (pentane/Et_2O, 3:1).

R_f = 0.73 (pentane/EtOAc, 2:1), vanillin: blue, UV (-)

^{1}H NMR (600 MHz, $CDCl_3$) δ [ppm] = 6.04 (dd, *J* = 10.3, 3.0 Hz, 1H), 5.97 (ddd, *J* = 10.2, 5.1, 2.1 Hz, 1H), 5.83 (ddt, *J* = 17.2, 10.2, 7.0 Hz, 1H), 5.15 – 5.08 (m, 2H), 5.06 (dd, *J* = 5.2, 2.7 Hz, 1H), 4.34 (ddt, *J* = 8.4, 5.5, 2.6 Hz, 1H), 4.22 – 4.16 (m, 2H), 4.15 – 4.06 (m, 2H), 2.43 (dddt, *J* = 14.3, 8.3, 6.8, 1.4 Hz, 1H), 2.28 (dddt, *J* = 14.2, 7.1, 5.7, 1.3 Hz, 1H), 2.07 (s, 3H), 2.06 (s, 3H).

^{13}C NMR (151 MHz, $CDCl_3$) δ [ppm] = 170.7, 170.5, 134.8, 133.9, 122.0, 117.6, 72.2, 68.0, 63.8, 63.0, 36.7, 20.9, 20.8.

HRMS (ESI) (*m/z*) calcd. for $[C_{13}H_{18}O_5Na]^+$ = $[M+Na]^+$: Calcd. 277.1052, found 277.1058.

Specific Rotation $[\alpha]_{589}^{20}$ = +278° (c = 1.04, $CHCl_3$)

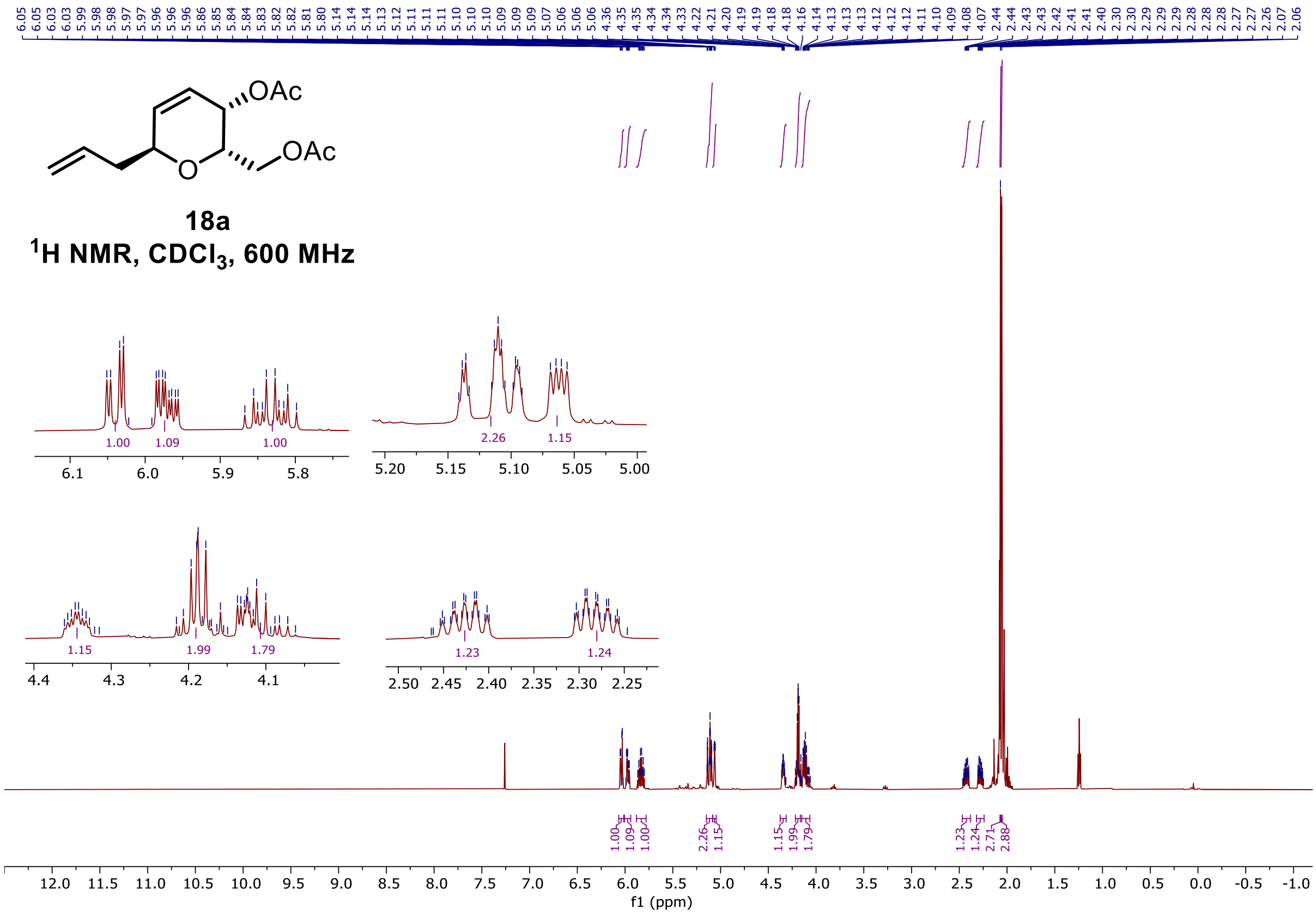
OAc
OAc
O
18a
^{1}H NMR, CDCl$_3$, 600 MHz
f1 (ppm)

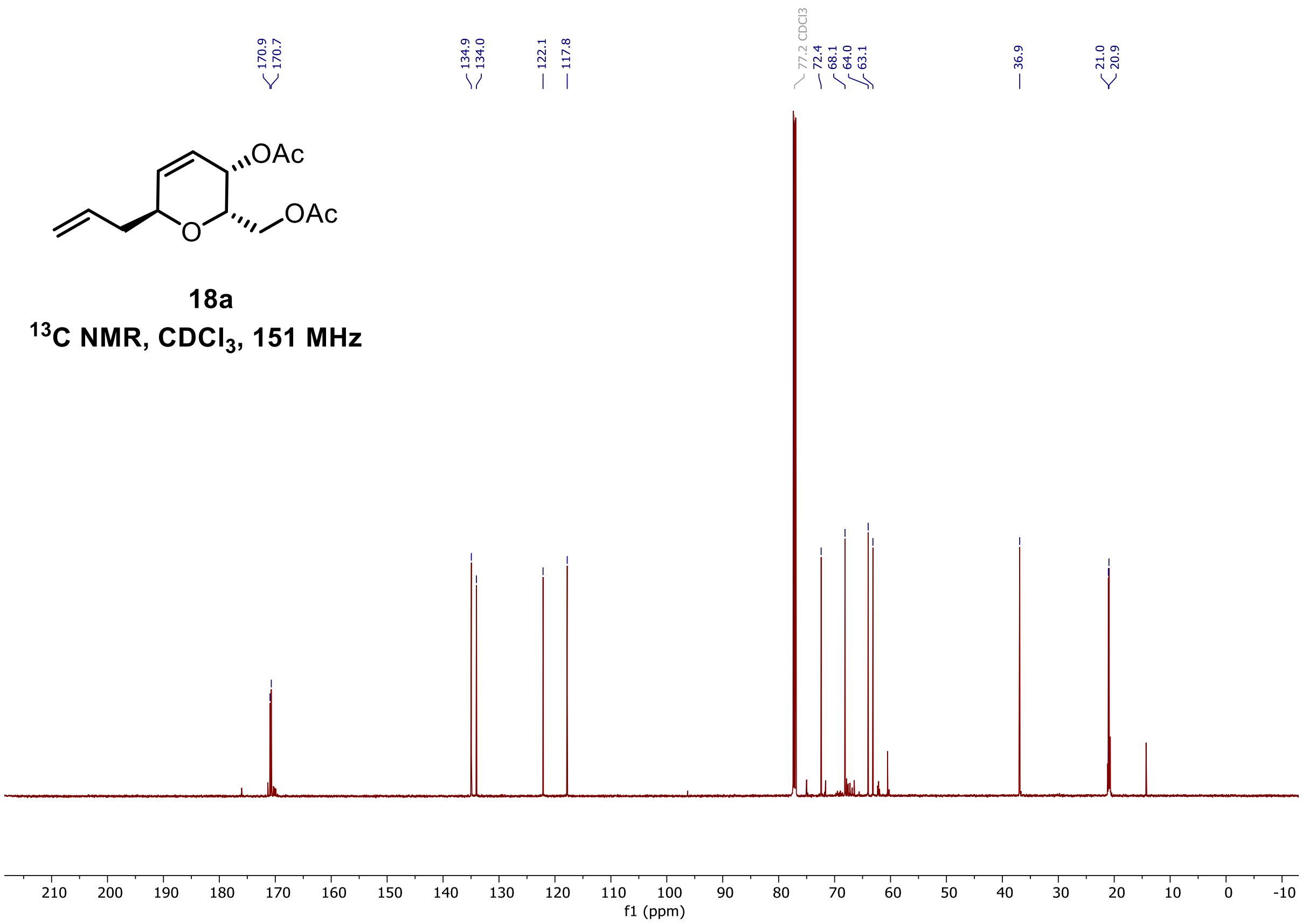
OAc
OAc
O
18a
^{13}C NMR, CDCl$_3$, 151 MHz
170.9
170.7
134.9
134.0
122.1
117.8
77.2 CDCl3
72.4
68.1
64.0
63.1
36.9
21.0
20.9
f1 (ppm)

Diol 33

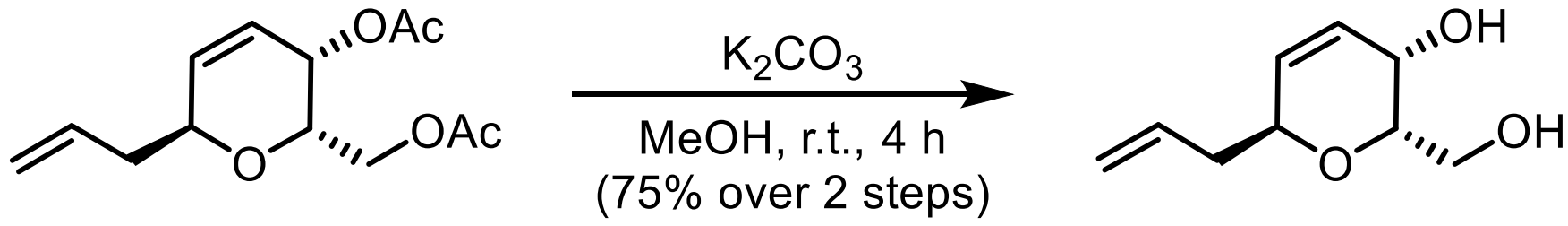

18a **33**

The crude **18a** (1.64 g) was dissolved in 18 mL MeOH and treated with K_2CO_3 (1.05 g, 7.61 mmol, 1.5 eq.) and stirred for 4 h at r.t. The reaction was diluted with sat. NH_4Cl solution (25 mL) and extracted with EtOAc (7 x 50 mL), the organic layer was washed with sat. NaCl solution (50 mL), dried over $MgSO_4$ and concentrated under reduced pressure. The residue was purified by flash chromatography on silica (pentane/EtOAc, 1:4) to yield diol **33** (648 mg, 3.81 mmol, 75% over 2 steps) as a slightly yellow oil.

R_f = 0.13 (pentane/EtOAc, 2:1), vanillin: dark blue, UV (-)

^{1}H NMR (400 MHz, $CDCl_3$) δ [ppm] = 6.02 (ddd, *J* = 10.3, 5.4, 1.9 Hz, 1H), 5.94 (dd, *J* = 10.2, 3.1 Hz, 1H), 5.83 (ddt, *J* = 17.1, 10.2, 7.0 Hz, 1H), 5.17 – 5.07 (m, 2H), 4.33 (ddt, *J* = 8.6, 5.5, 2.4 Hz, 1H), 3.96 – 3.84 (m, 2H), 3.84 – 3.73 (m, 2H), 2.57 (s, 1H), 2.49 – 2.35 (m, 1H), 2.28 (dtt, *J* = 12.8, 6.9, 1.3 Hz, 1H). One OH-group not detectable.

^{13}C NMR (101 MHz, $CDCl_3$) δ [ppm] = 134.4, 133.1, 126.3, 117.8, 72.9, 71.7, 63.2, 63.1, 36.9.

HRMS (ESI) (*m/z*) calcd. for $[C_9H_{14}O_3Na]^+$ = $[M+Na]^+$: calcd. 193.0841, found 193.0853.

Specific Rotation $[\alpha]_{589}^{20}$ = +216° (c = 0.873, $CHCl_3$)

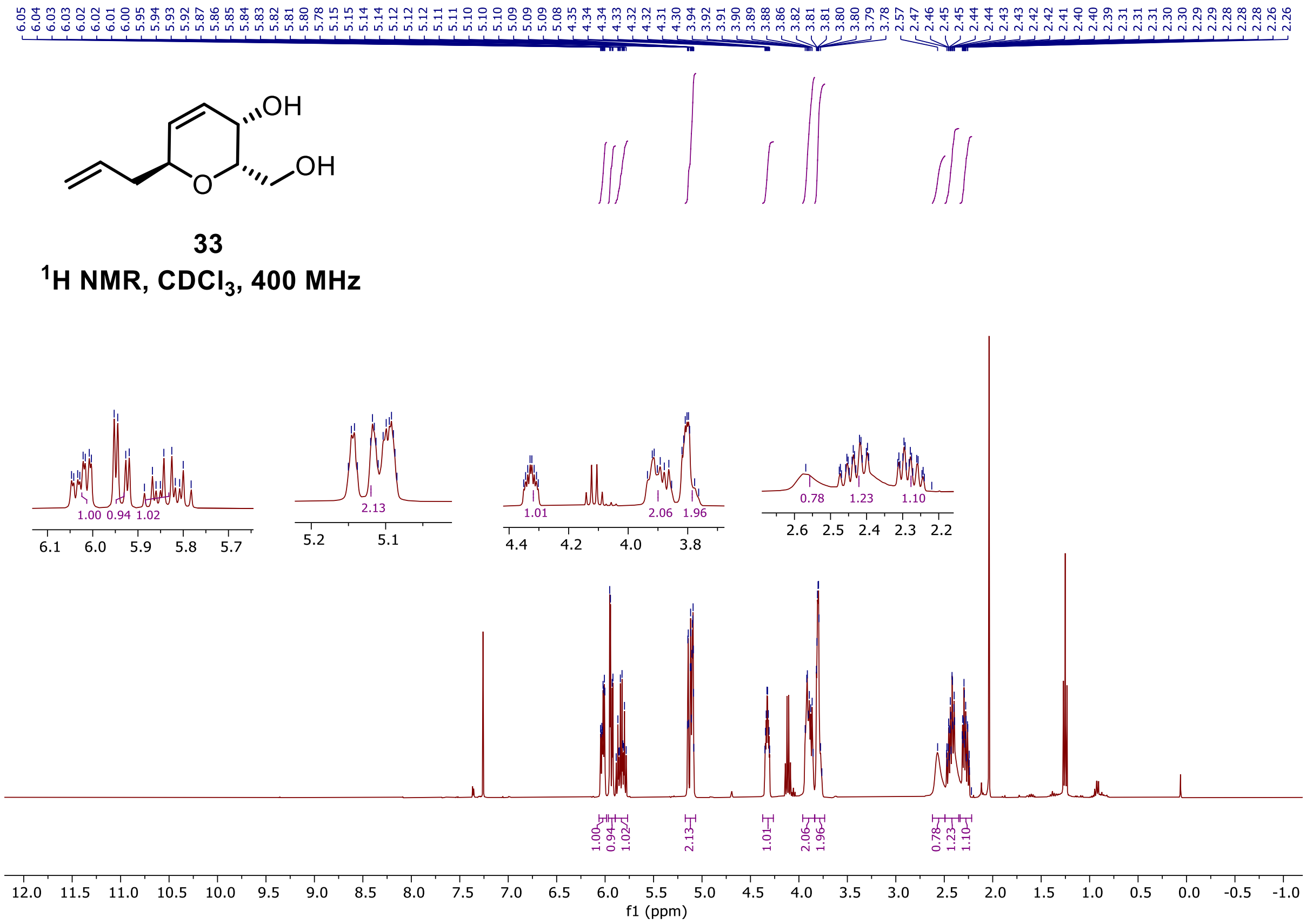

33

^{1}H NMR, $CDCl_3$, 400 MHz

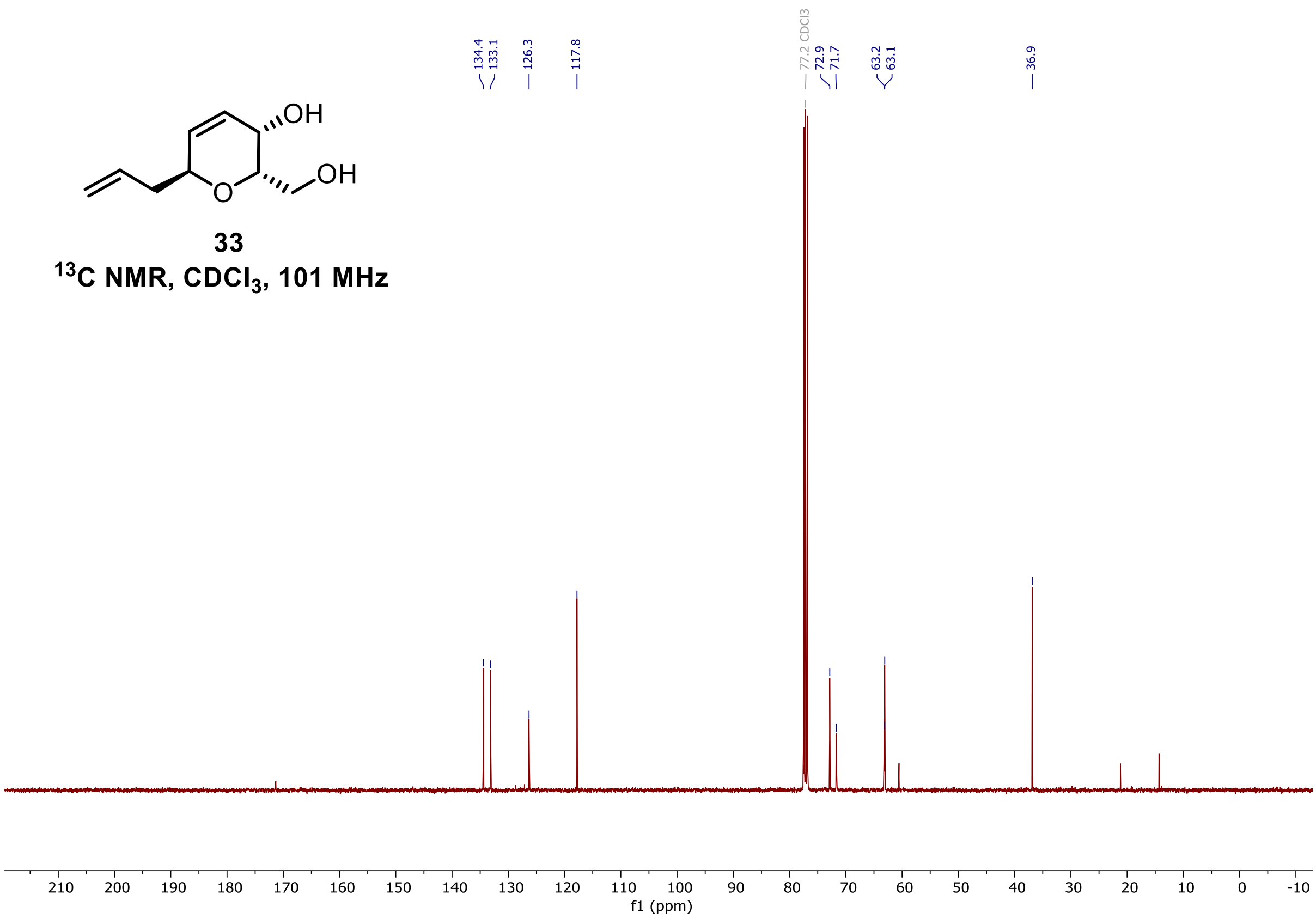

33

^{13}C NMR, $CDCl_3$, 101 MHz

TIPS ether 34a

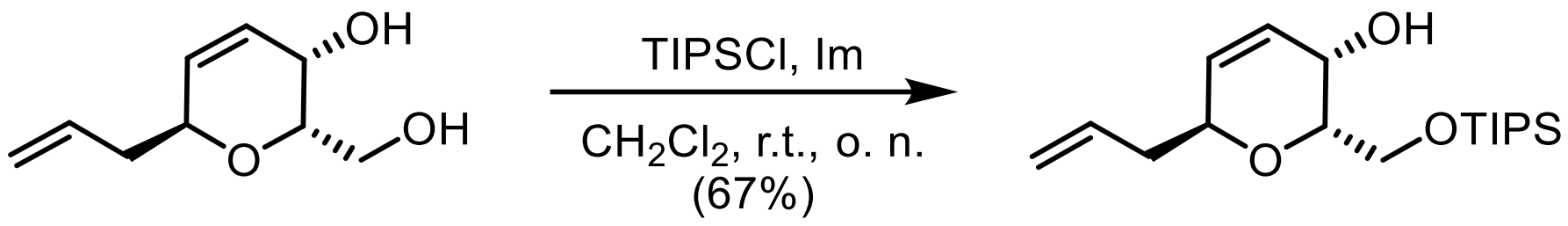

33 **34a**

To a solution of diol **33** (3.63 g, 21.4 mmol, 1.0 eq.) in 110 mL dry CH_2Cl_2 were added imidazole (2.18 g, 32.0 mmol, 1.5 eq.) and TIPSCl (6.9 mL, 32.0 mmol, 1.5 eq.). The mixture was stirred overnight at r.t., quenched with sat. $NaHCO_3$ solution, and the mixture was extracted with CH_2Cl_2. The combined organic layer was dried over $MgSO_4$, and the solvent was evaporated under reduced pressure. The residue was purified by flash chromatography (pentane/Et_2O, 4:1) to yield alcohol **34a** (4.69 g, 14.4 mmol, 67%) as a colorless oil.

$\mathbf{R_f}$ = 0.80 (Et_2O), vanillin: dark blue, UV (-).

^{1}H NMR (400 MHz, $CDCl_3$) δ [ppm] = 6.06 (ddd, *J* = 10.2, 5.4, 2.1 Hz, 1H), 5.92 (dd, *J* = 10.2, 3.2 Hz, 1H), 5.90 – 5.79 (m, 1H), 5.16 – 5.04 (m, 2H), 4.27 (ddt, *J* = 8.4, 5.5, 2.6 Hz, 1H), 3.99 (br s, 1H), 3.97 – 3.90 (m, 1H), 3.89 – 3.79 (m, 2H), 2.44 (dddt, *J* = 14.0, 8.1, 6.7, 1.4 Hz, 1H), 2.27 (dddt, *J* = 14.4, 7.2, 5.9, 1.2 Hz, 1H), 2.10 (d, *J* = 8.0 Hz, 1H), 1.17 – 1.00 (m, 21H).

^{13}C NMR (101 MHz, $CDCl_3$) δ [ppm] = 134.6, 132.8, 126.6, 117.5, 73.0, 71.9, 63.2, 62.4, 37.2, 18.1, 12.0.

IR (ATR) ṽ (cm^{-1}) = 3436 (br), 2953 (m), 2929 (m), 2885 (m), 2856 (m), 1642 (w), 1470 (m), 1362 (m), 1253 (m), 1186 (w), 1089 (s), 1047 (s), 996 (m), 914 (m), 866 (m), 833 (s), 776 (s), 743 (m), 665 (w).

HRMS (ESI) (*m/z*) calcd. for $[C_{18}H_{34}O_3Si]^+ = [M+H]^+$: 327.2355, found: 327.2358.

Specific Rotation $[\alpha]_{589}^{20}$= +141.0° (c = 0.77, $CHCl_3$).

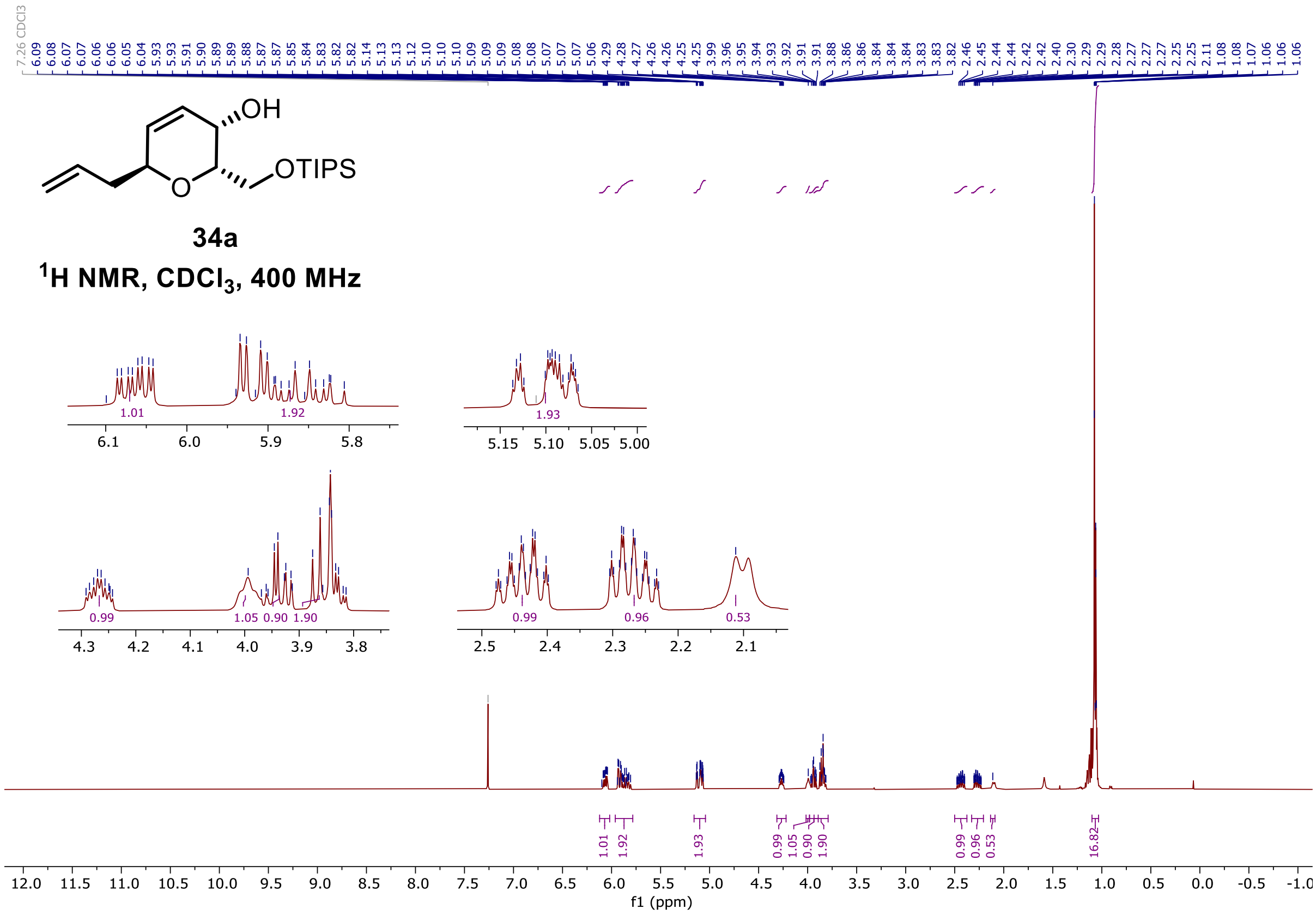
7.26 CDCl3
OH
OTIPS
O
34a
1H NMR, CDCl3, 400 MHz
f1 (ppm)

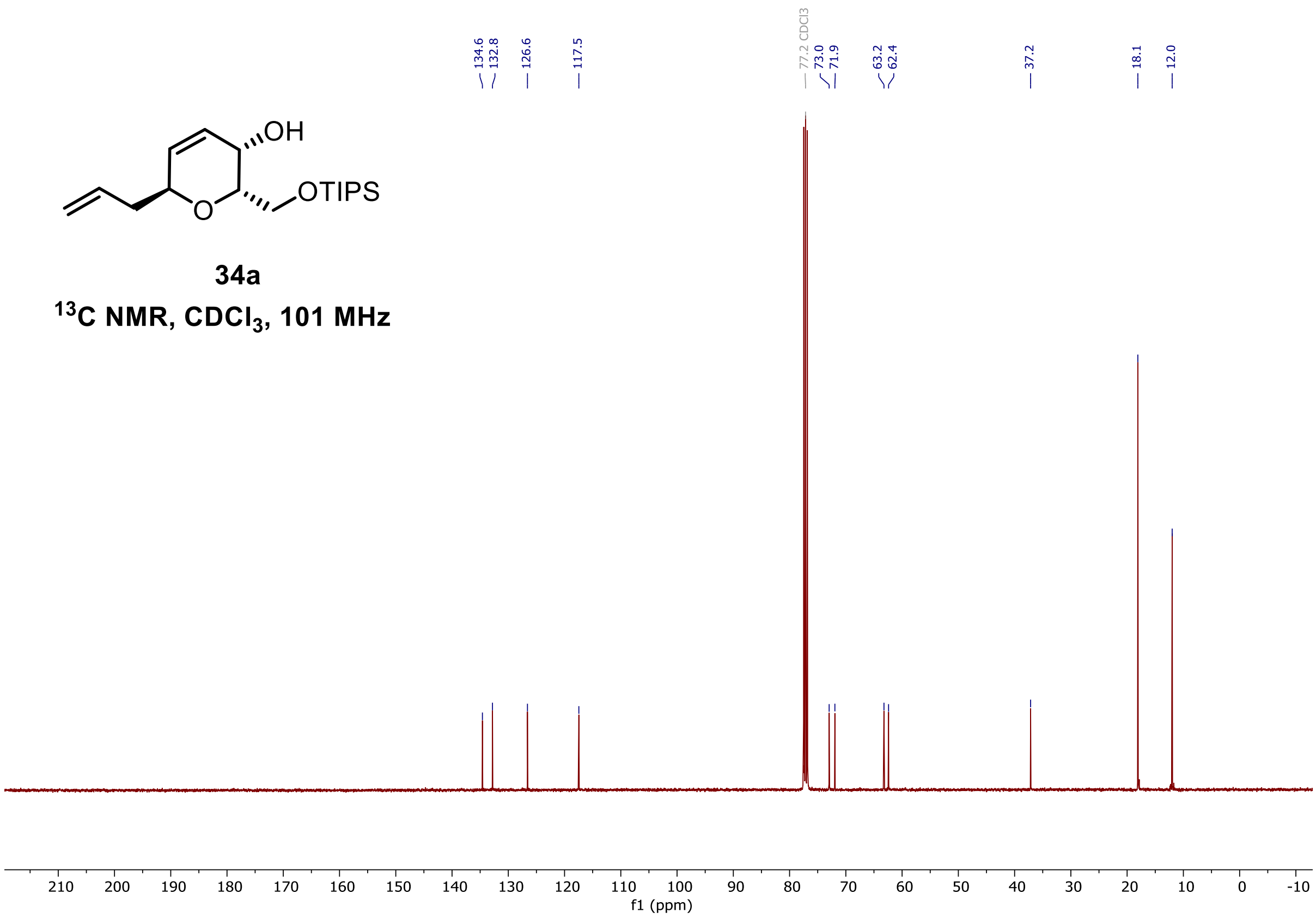
134.6
132.8
126.6
117.5
77.2 CDCl3
73.0
71.9
63.2
62.4
37.2
18.1
12.0
OH
OTIPS
O
34a
13C NMR, CDCl3, 101 MHz
f1 (ppm)

MOM ether 34b

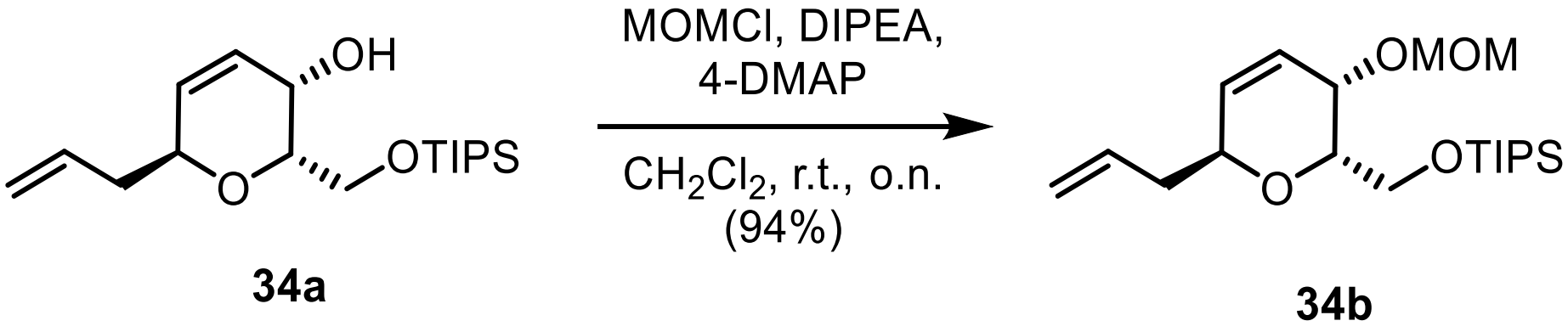

To a solution of alcohol **34a** (4.69 g, 14.4 mmol) in 100 mL dry CH_2Cl_2 were added DIPEA (3.76 mL, 21.6 mmol, 1.5 eq.), 4-DMAP (88 mg, 0.72 mmol, 5 mol%), and MOMCl (1.62 mL, 21.0 mmol, 1.5 eq.). After stirring overnight at r.t., sat. $NaHCO_3$ solution was added and the mixture was extracted with CH_2Cl_2. The combined organic layers were washed with sat. NaCl solution, dried over Na_2SO_4 and the solvent was evaporated. The residue was purified by flash chromatography (pentane/Et_2O, 5:1) to yield dihydropyran **34b** (5.01 g, 13.5 mmol, 94%) as a colorless oil.

R_f = 0.75 (pentane/Et_2O, 5:1), vanillin: dark blue, UV (-).

^{1}H NMR (400 MHz, $CDCl_3$) δ [ppm] = 6.05 (ddd, *J* = 10.2, 5.4, 2.1 Hz, 1H), 5.95 (dd, *J* = 10.3, 3.0 Hz, 1H), 5.85 (ddt, *J* = 17.1, 10.1, 7.0 Hz, 1H), 5.15 – 5.03 (m, 2H), 4.81 – 4.69 (m, 2H), 4.31 (ddt, *J* = 8.4, 5.6, 2.6 Hz, 1H), 3.97 – 3.82 (m, 3H), 3.78 (dd, *J* = 8.3, 4.8 Hz, 1H), 3.39 (s, 3H), 2.44 (dddt, *J* = 14.1, 8.1, 6.7, 1.4 Hz, 1H), 2.28 (dddt, *J* = 14.4, 7.3, 6.0, 1.2 Hz, 1H), 1.06 (s, 21H).

^{13}C NMR (101 MHz, $CDCl_3$) δ [ppm] = 134.7, 133.3, 125.0, 117.4, 96.4, 72.6, 72.0, 67.0, 62.2, 55.6, 37.3, 18.1, 12.0,

IR (ATR) ṽ (cm^{-1}) = 2952 (w), 2929 (m), 2895 (w), 2857 (w), 1470 (w), 1253 (m), 1149 (m), 1086 (s), 1037 (s), 915 (w), 833 (s), 774 (s), 740 (m), 665 (w).

HRMS (ESI) (*m/z*) calcd. for $[C_{18}H_{35}O_3Si]^+$ = $[M+H]^+$: 371.2618, found: 371.2612.

Specific Rotation $[\alpha]_{589}^{20}$= +111.0° (c = 0.96, $CHCl_3$).

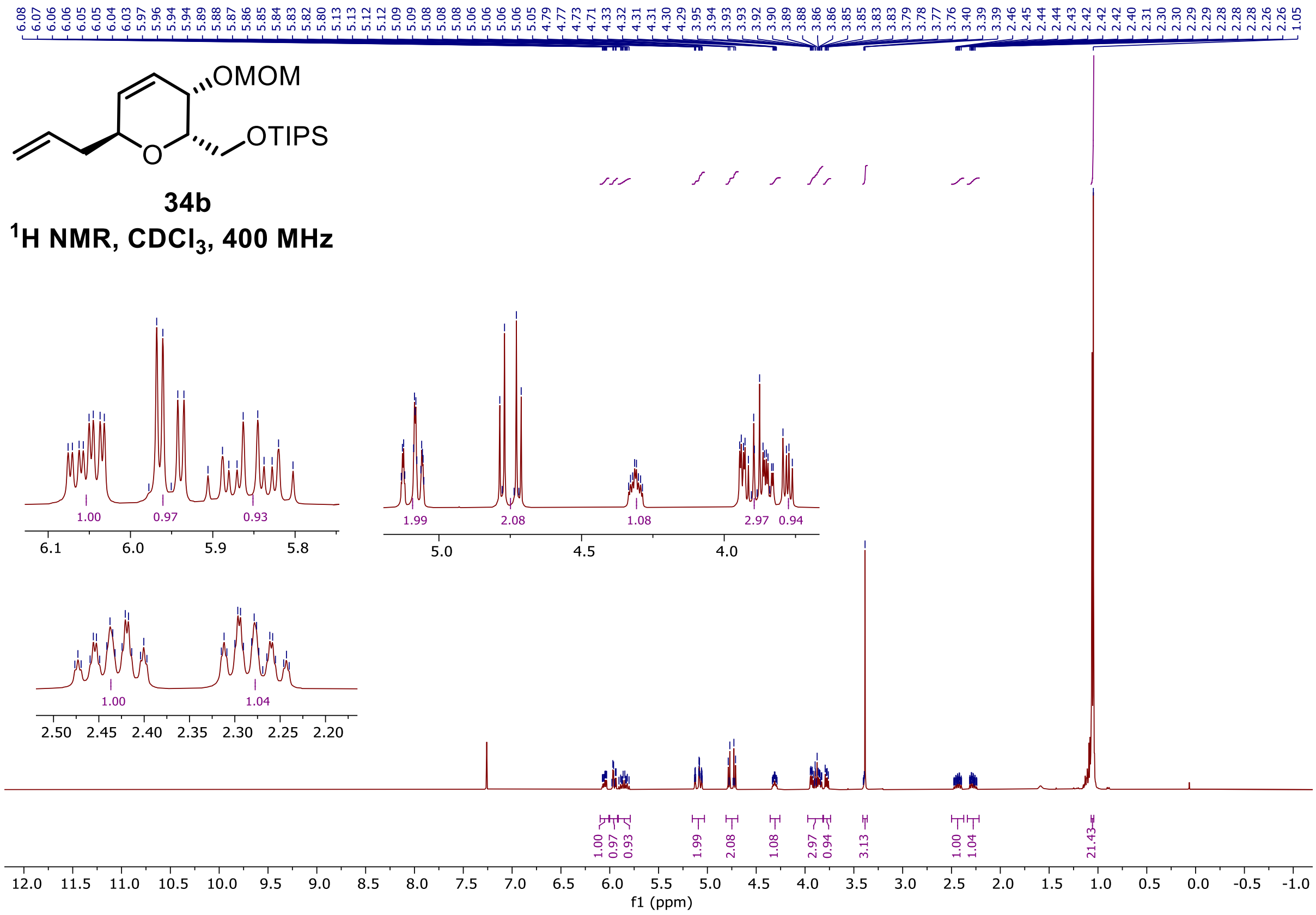
OMOM
OTIPS
O
34b
^{1}H NMR, CDCl$_3$, 400 MHz
f1 (ppm)

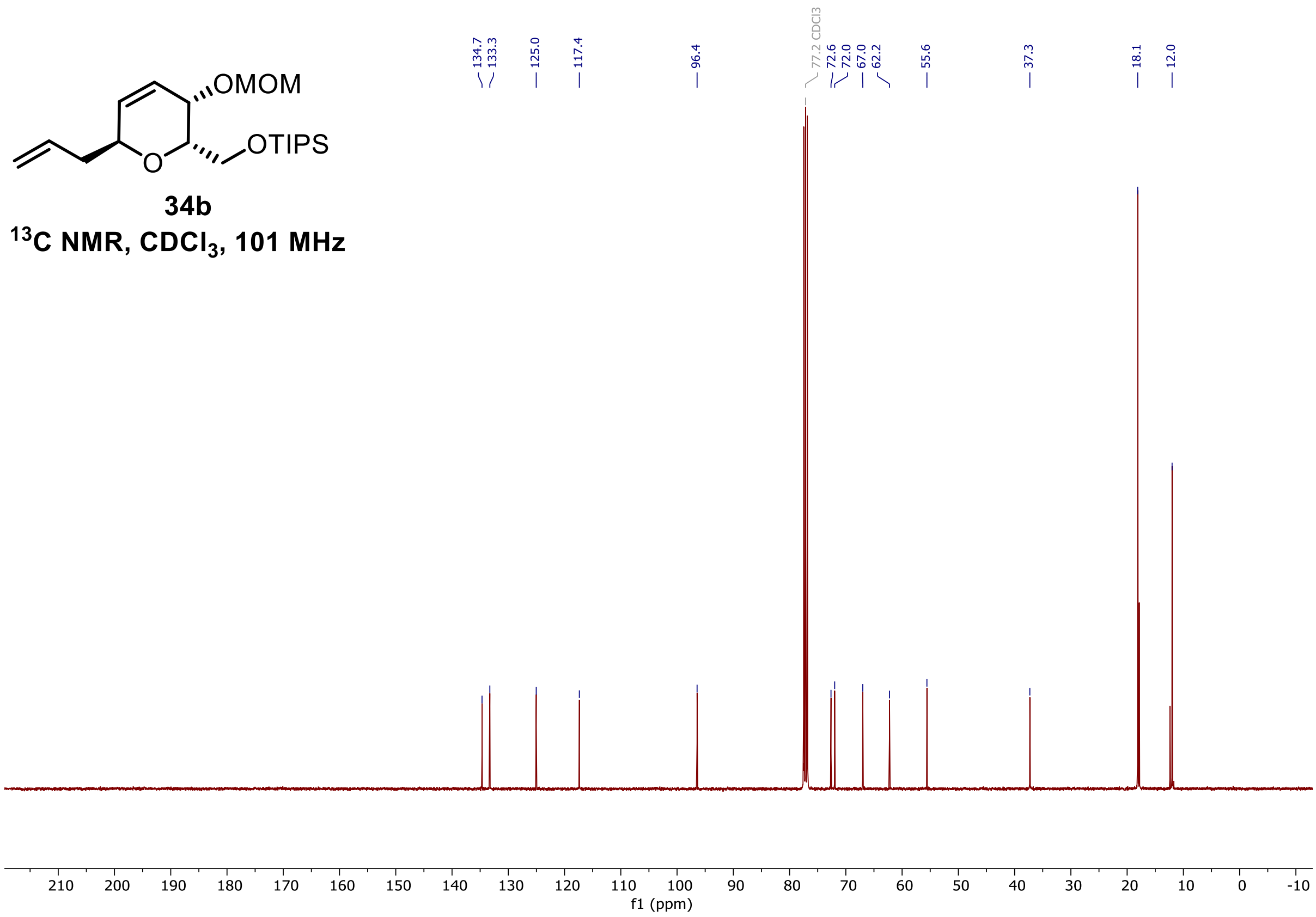
OMOM
OTIPS
O
34b
^{13}C NMR, CDCl$_3$, 101 MHz
134.7
133.3
125.0
117.4
96.4
77.2 CDCl3
72.6
72.0
67.0
62.2
55.6
37.3
18.1
12.0
f1 (ppm)

Alcohol 34

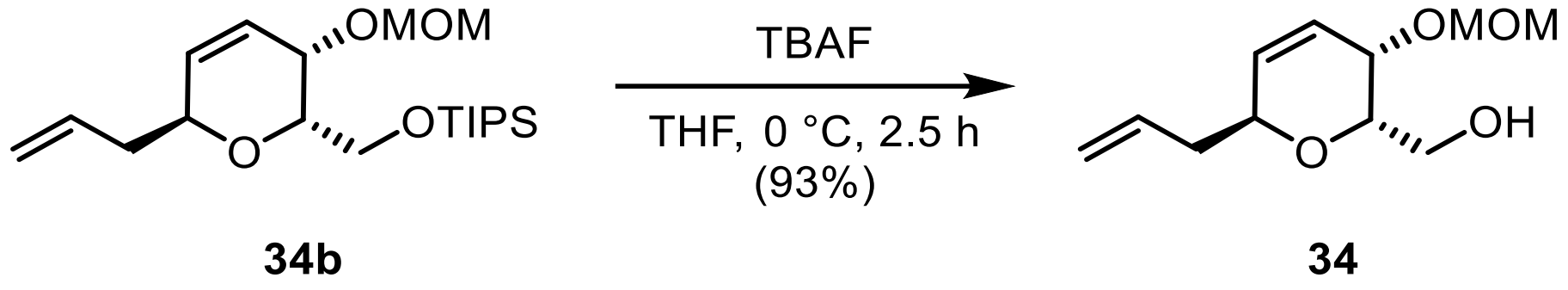

A solution of silyl ether **34b** (4.89 g, 13.2 mmol, 1.0 eq.) in 30 mL dry THF was treated with TBAF (1M in THF, 26.4 mL, 26.4 mmol, 2.0 eq.) at 0 °C. After 2.5 h, the reaction was quenched with sat. NH_4Cl solution and the mixture was extracted with Et_2O. The combined organic layer was dried over $MgSO_4$, and the solvent was evaporated under reduced pressure. The residue was purified by flash chromatography (pentane/Et_2O, 1:2) to yield alcohol **34** (2.63 g, 12.3 mmol, 93%) as a colorless oil.

R_f = 0.50 (Et_2O), vanillin: dark blue, UV (-).

^{1}H NMR (400 MHz, $CDCl_3$) δ [ppm] = 6.06 – 5.93 (m, 2H), 5.91 – 5.76 (m, 1H), 5.18 – 5.06 (m, 2H), 4.76 (dd, *J* = 6.7, 1.6 Hz, 1H), 4.64 (dd, *J* = 6.7, 1.3 Hz, 1H), 4.34 (ddd, *J* = 8.0, 5.8, 1.9 Hz, 1H), 3.93 – 3.81 (m, 1H), 3.74 (ddd, *J* = 11.1, 8.7, 4.4 Hz, 1H), 3.39 (d, *J* = 1.8 Hz, 3H), 2.43 (dddt, *J* = 14.2, 8.4, 7.1, 1.3 Hz, 1H), 2.31 – 2.22 (m, 2H).

^{13}C NMR (101 MHz, $CDCl_3$) δ [ppm] = 134.5, 133.5, 124.0, 117.7, 96.0, 72.1, 71.7, 67.9, 62.3, 55.9, 37.2.

IR (ATR) ṽ (cm^{-1}) = 3444 (br), 2931 (m), 2888 (m), 1641 (w), 1439 (w), 1397 (w), 1213 (w), 1148 (m), 1079 (s), 985 (s), 913 (s), 742 (m), 689 (w).

HRMS (ESI) (*m/z*) calcd. for $[C_{11}H_{18}O_4Na]^+$ = $[M+Na]^+$: 237.1103, found: 237.1105.

Specific Rotation $[\alpha]_{589}^{20}$= +298.0° (c = 1.55, $CHCl_3$).

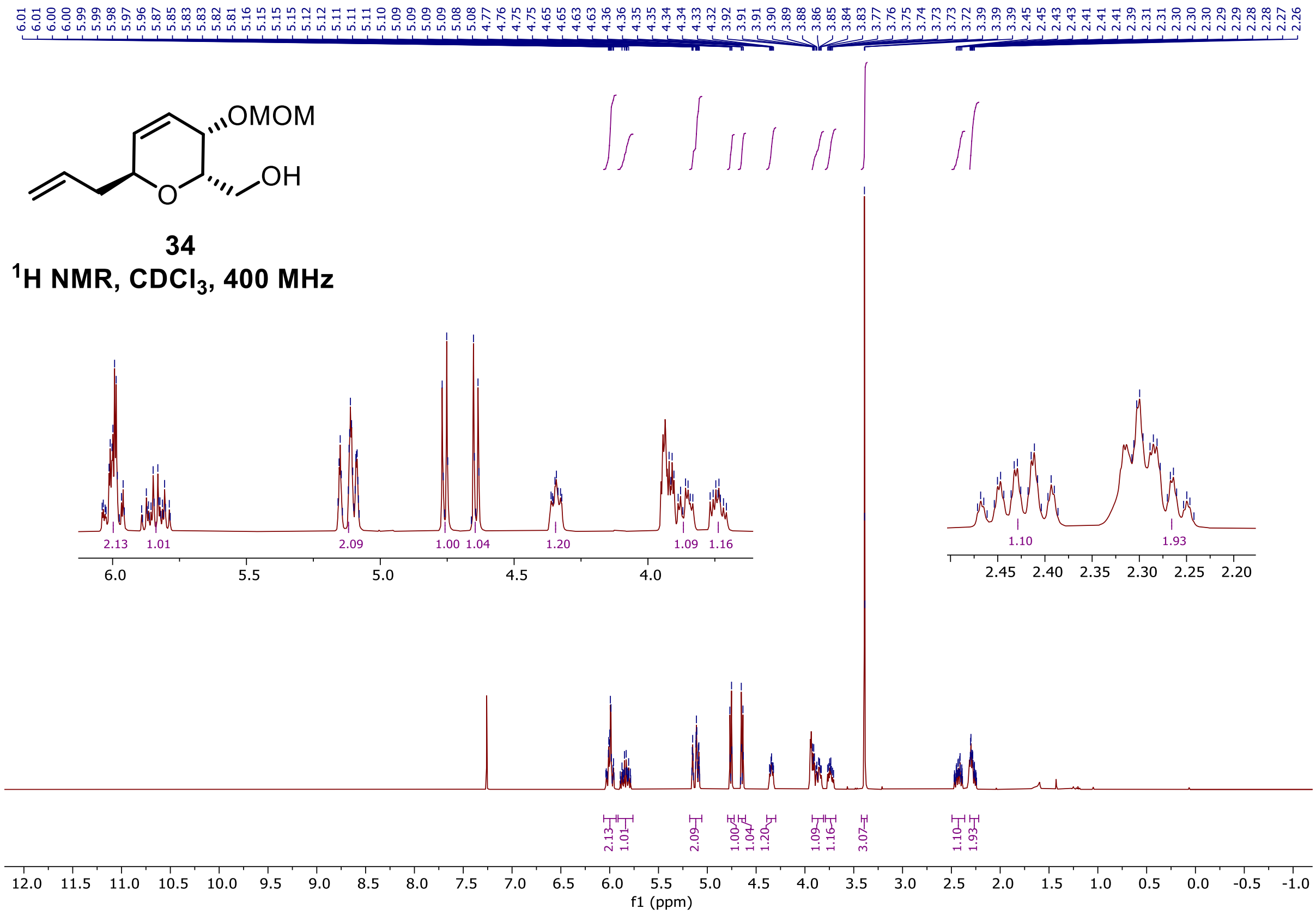

34

^{1}H NMR, CDCl$_3$, 400 MHz

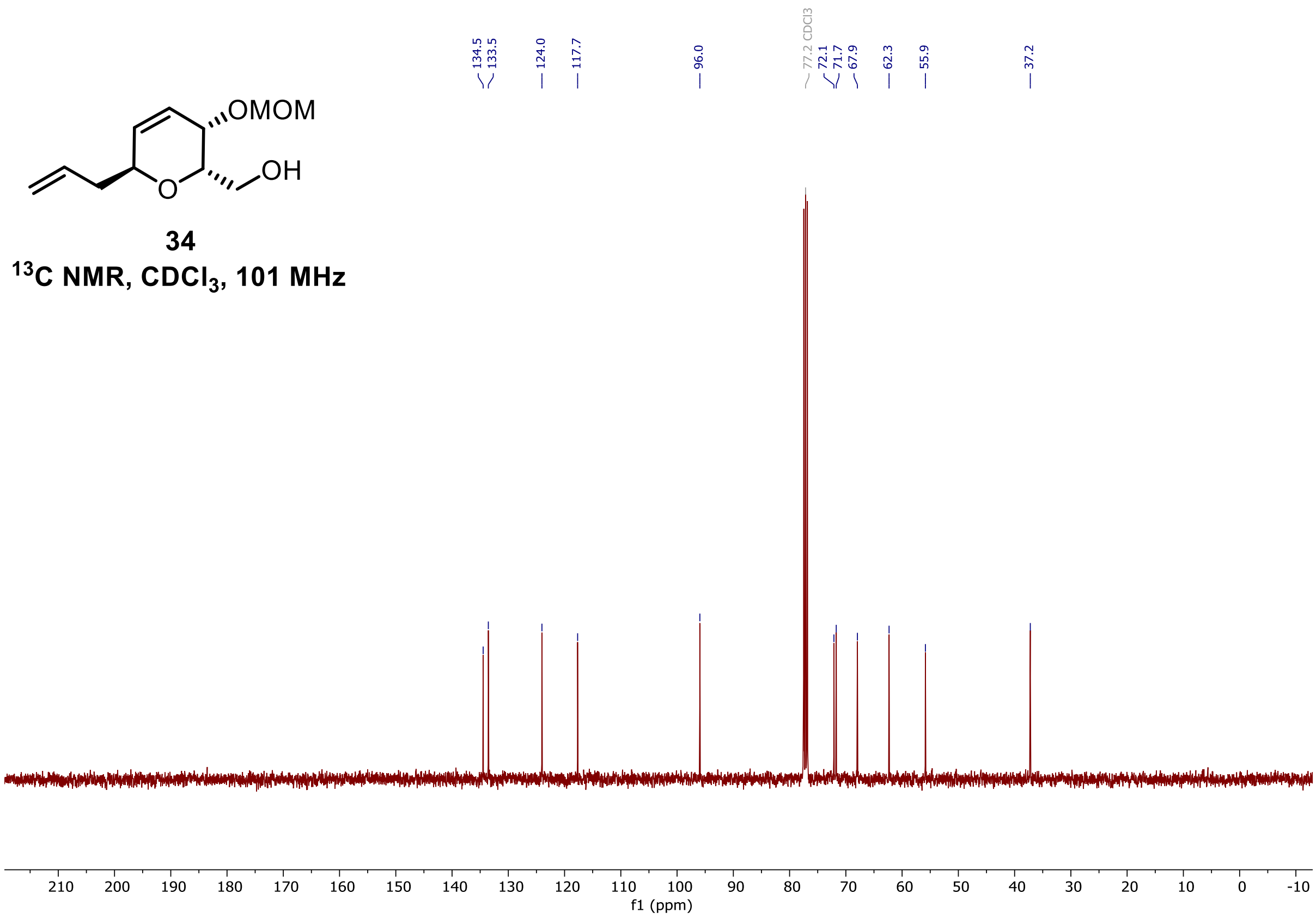

34

^{13}C NMR, CDCl$_3$, 101 MHz

Aldehyde 17a

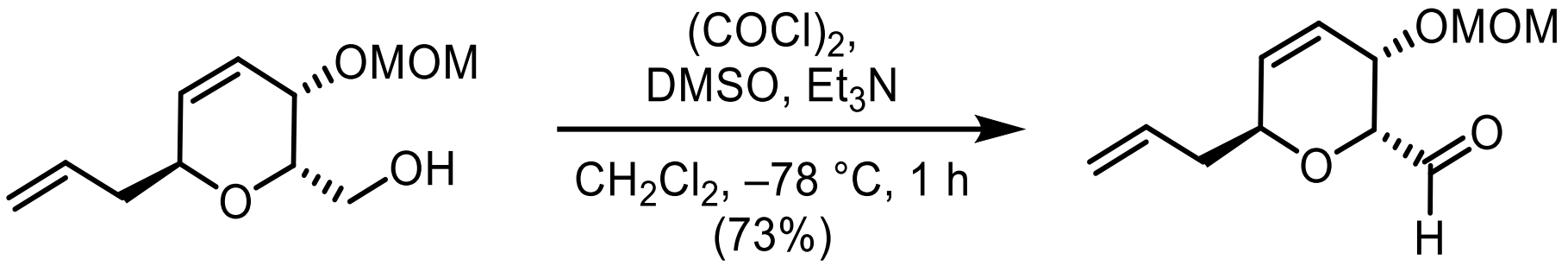

34 **17a**

A round bottom flask was charged with oxalyl chloride (2.1 mL, 24.5 mmol, 2.0 eq.), dissolved in 30 mL dry CH_2Cl_2, and cooled to –78 °C. DMSO (2.6 mL, 36.6 mmol, 3.0 eq.), dissolved in 10 mL dry CH_2Cl_2, was added slowly at –78 °C and the resulting mixture was stirred for 30 min. Next, alcohol **34** (2.63 g, 12.3 mmol, 1.0 eq.), dissolved in 10 mL dry CH_2Cl_2, was added slowly to the mixture and stirring was continued for 1 h at –78 °C. Subsequently, Et_3N (10.3 mL, 73.7 mmol, 6.0 eq.) was added slowly, the reaction was warmed up to r.t. and stirring was continued for additional 30 min. Upon completion of the reaction, the mixture was diluted with water, the layers were separated and the aqueous layer was extracted with CH_2Cl_2. The combined organic layer was washed with sat. NaCl solution, dried over Na_2SO_4 and concentrated in vacuo. The crude material was purified by flash chromatography (pentane/Et_2O, 3:1) to afford aldehyde **17a** (1.9 g, 9.0 mmol, 73%) as a colorless oil.

$\mathbf{R_f}$ = 0.50 (pentane/Et_2O, 1:1), vanillin: dark blue, UV (-).

^{1}H NMR (400 MHz, $CDCl_3$) δ [ppm] = 9.70 (s, 1H), 6.04 (ddd, *J* = 10.4, 4.4, 2.1 Hz, 1H), 5.96 (ddd, *J* = 10.5, 2.8, 0.6 Hz, 1H), 5.90 – 5.76 (m, 1H), 5.17 – 5.05 (m, 2H), 4.70 (d, *J* = 6.9 Hz, 1H), 4.63 (d, *J* = 6.9 Hz, 1H), 4.57 – 4.47 (m, 1H), 4.30 – 4.21 (m, 2H), 3.33 (s, 3H), 2.49 – 2.23 (m, 2H).

^{13}C NMR (101 MHz, $CDCl_3$) δ [ppm] = δ 201.5, 133.8, 133.2, 123.9, 118.0, 95.9, 77.5, 77.4, 77.2, 76.8, 76.5, 72.5, 67.8, 55.8, 37.6.

IR (ATR) ṽ (cm^{-1}) = 2932 (w), 2892 (m), 2825 (w), 2328 (w), 1736 (s), 1641 (w), 1440 (m), 1354 (m), 1149 (s), 1089 (s), 1032 (s), 916 (s), 742 (m), 636 (w), 555 (w).

HRMS (ESI) (*m/z*) calcd. for $[C_{11}H_{16}O_4Na]^+$ = $[M+Na]^+$: 235.0946, found: 235.0949.

Specific Rotation $[\alpha]^{20}_{589}$= +262.0° (c = 0.475, $CHCl_3$).

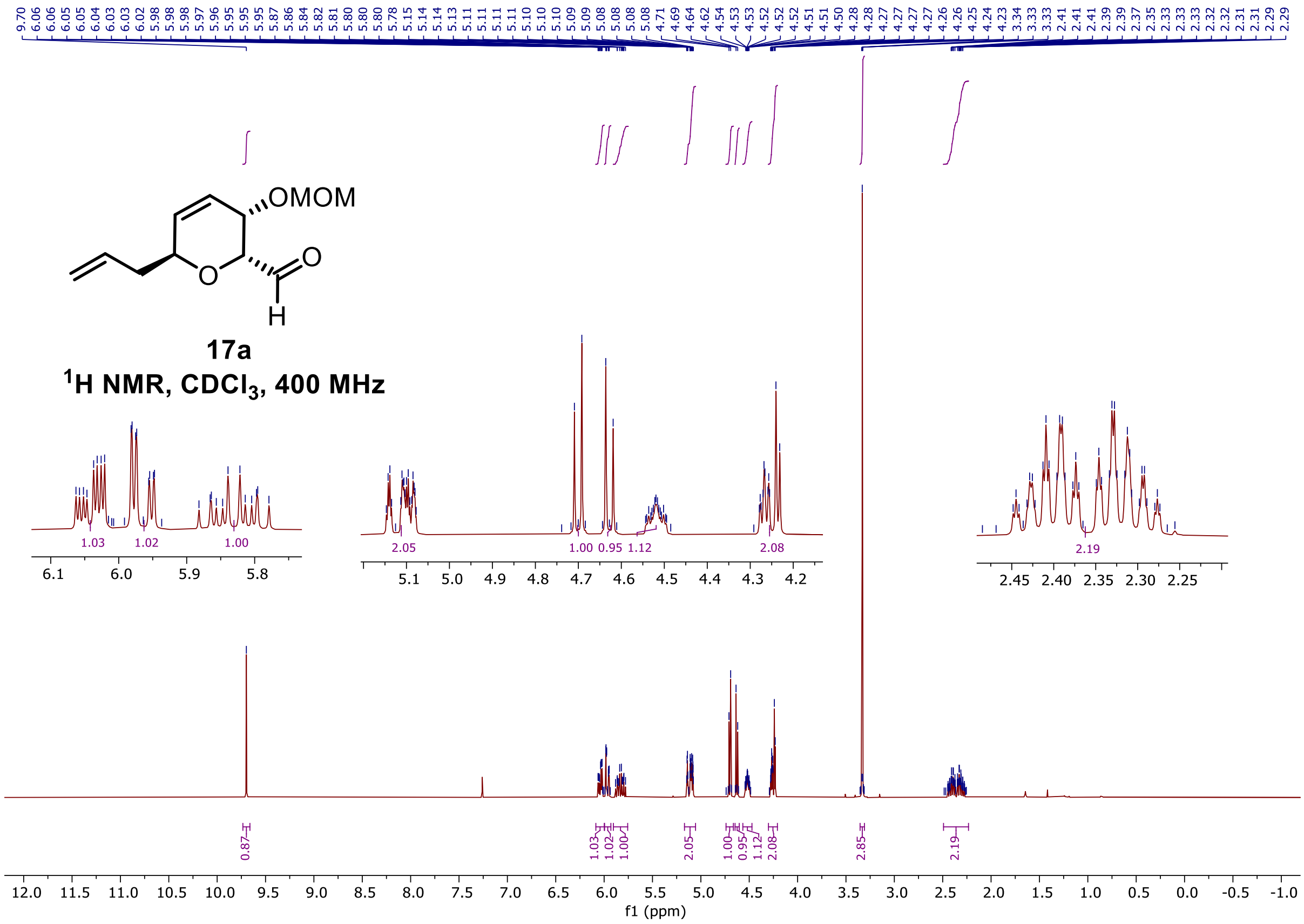

17a

^1H NMR, $CDCl_3$, 400 MHz

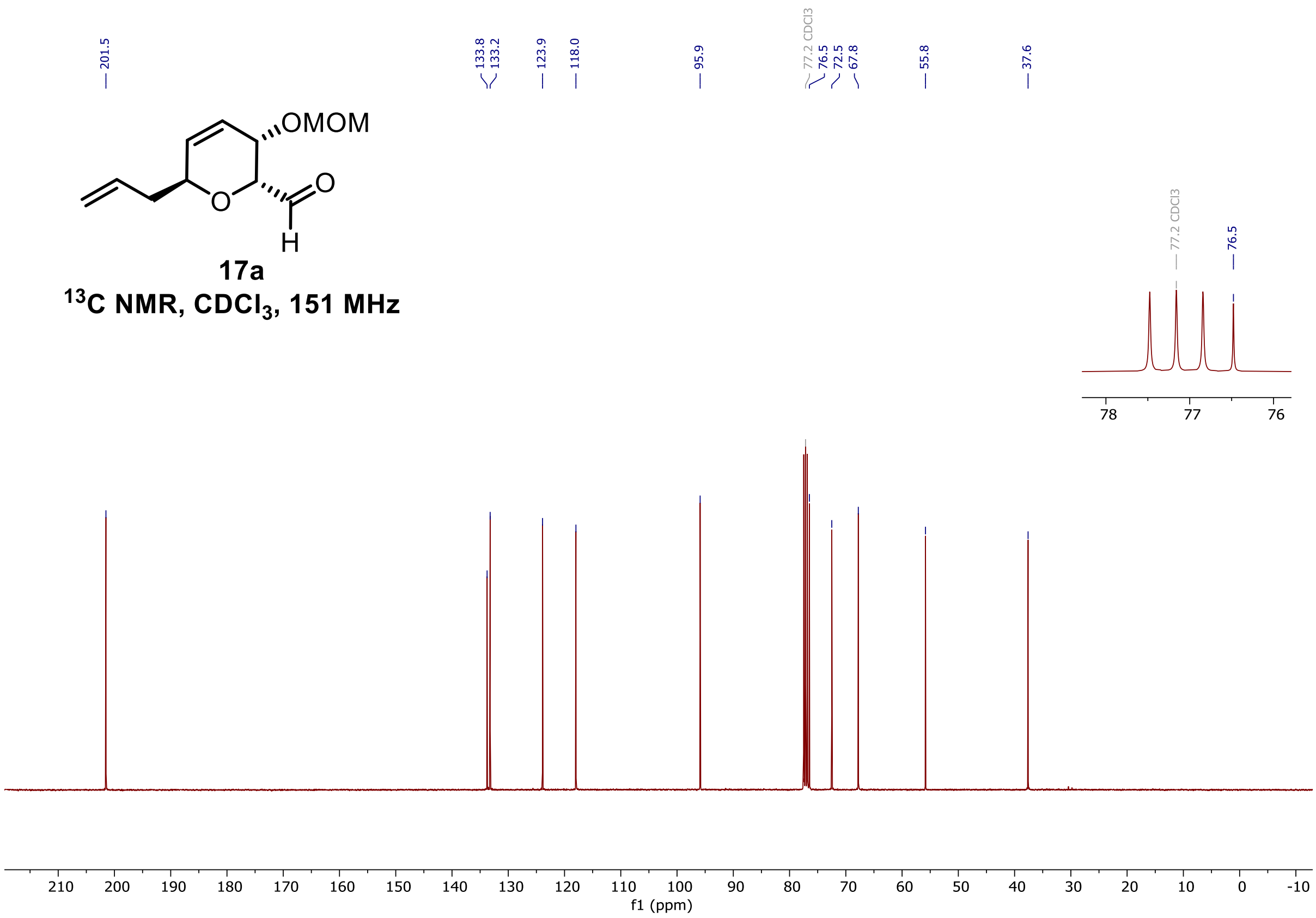

17a

^{13}C NMR, $CDCl_3$, 151 MHz

Methyl ketone 17

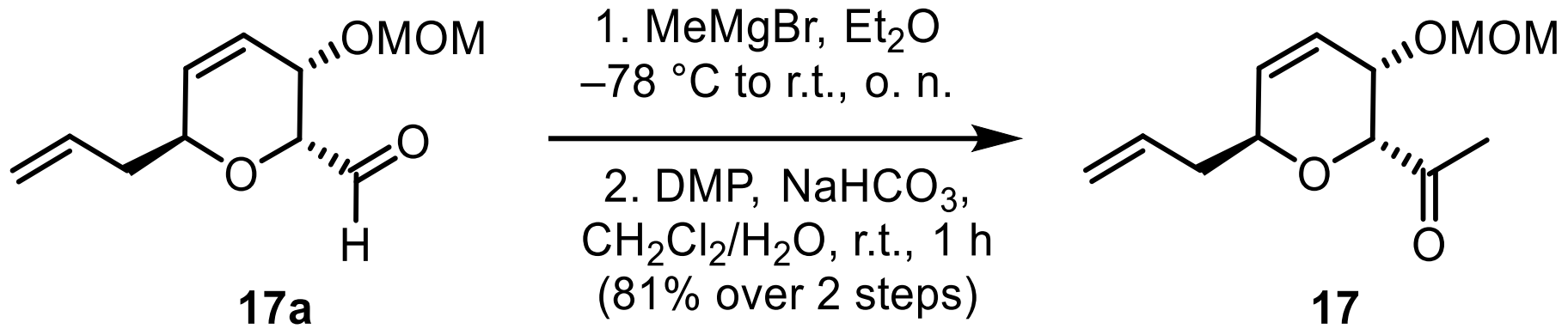

A solution of aldehyde **17a** (1.90 g, 9.0 mmol, 1.0 eq.) in 75 mL dry Et_2O was cooled to –78 °C and methylmagnesium bromide (3.0M in Et_2O, 9.0 mL, 27 mmol, 3.0 eq.) was added. The mixture was stirred overnight, while slowly warm to r.t., quenched with sat. $NaHCO_3$ solution, and the mixture was extracted with CH_2Cl_2. The combined organic layer was dried over $MgSO_4$, and the solvent was evaporated under reduced pressure. The crude alcohol (1.94 g, calcd. 8.5 mmol, ~95%) was used for the next step without further purification.

The crude alcohol (1.94 g, 8.5 mmol, 1.0 eq.) was dissolved in 80 mL dry CH_2Cl_2, saturated with water, before Dess-Martin periodinane (7.23 g, 17.1 mmol, 2.0 eq.) and $NaHCO_3$ (3.58 g, 42.6 mmol, 5.0 eq.) were added. After stirring at r.t. for 1 h, sat. $Na_2S_2O_3$ solution (100 mL) was added. The formed precipitate was dissolved by the addition of water and the layers were separated. The aqueous layer was extracted with CH_2Cl_2 and the combined organic layer was dried over $MgSO_4$, and the solvent was evaporated. The residue was purified by flash chromatography (pentane/Et_2O, 3:1) to afford methyl ketone **17** (1.65 g, 7.3 mmol, 81% over two steps) as a colorless oil.

R_f = 0.65 (pentane/Et_2O, 1:1), vanillin: dark blue, UV (-).

^{1}H NMR (400 MHz, $CDCl_3$) δ [ppm] = 6.07 (ddd, J = 10.3, 4.7, 1.9 Hz, 1H), 6.01 (dd, J = 10.3, 2.9 Hz, 1H), 5.85 (ddt, J = 17.1, 10.2, 7.0 Hz, 1H), 5.16 – 5.07 (m, 2H), 4.66 (d, J = 6.8 Hz, 1H), 4.59 (d, J = 6.8 Hz, 1H), 4.55 – 4.45 (m, 1H), 4.19 – 4.15 (m, 2H), 3.32 (s, 3H), 2.49 – 2.37 (m, 1H), 2.33 – 2.25 (obscured, m, 1H), 2.28 (s, 3H).

^{13}C NMR (101 MHz, $CDCl_3$) δ [ppm] = 209.0, 134.2, 133.7, 123.9, 117.7, 95.9, 77.0, 73.3, 67.6, 55.8, 36.7, 27.8.

IR (ATR) ṽ (cm^{-1}) = 2929 (w), 2892 (w), 2328 (w), 1715 (s), 1644 (w), 1417 (w), 1352 (m), 1322 (w), 1273 (w), 1211 (w), 1187 (w), 1150 (m), 1090 (s), 1031 (s), 954 (m), 916 (s), 734 (s), 645 (w), 546 (m).

HRMS (ESI) (*m/z*) calcd. for $[C_{12}H_{18}O_4Na]^+$ = $[M+Na]^+$: 249.1103, found: 249.1107.

Specific Rotation $[\alpha]^{20}_{589}$= +302.0° (c = 0.72, $CHCl_3$).

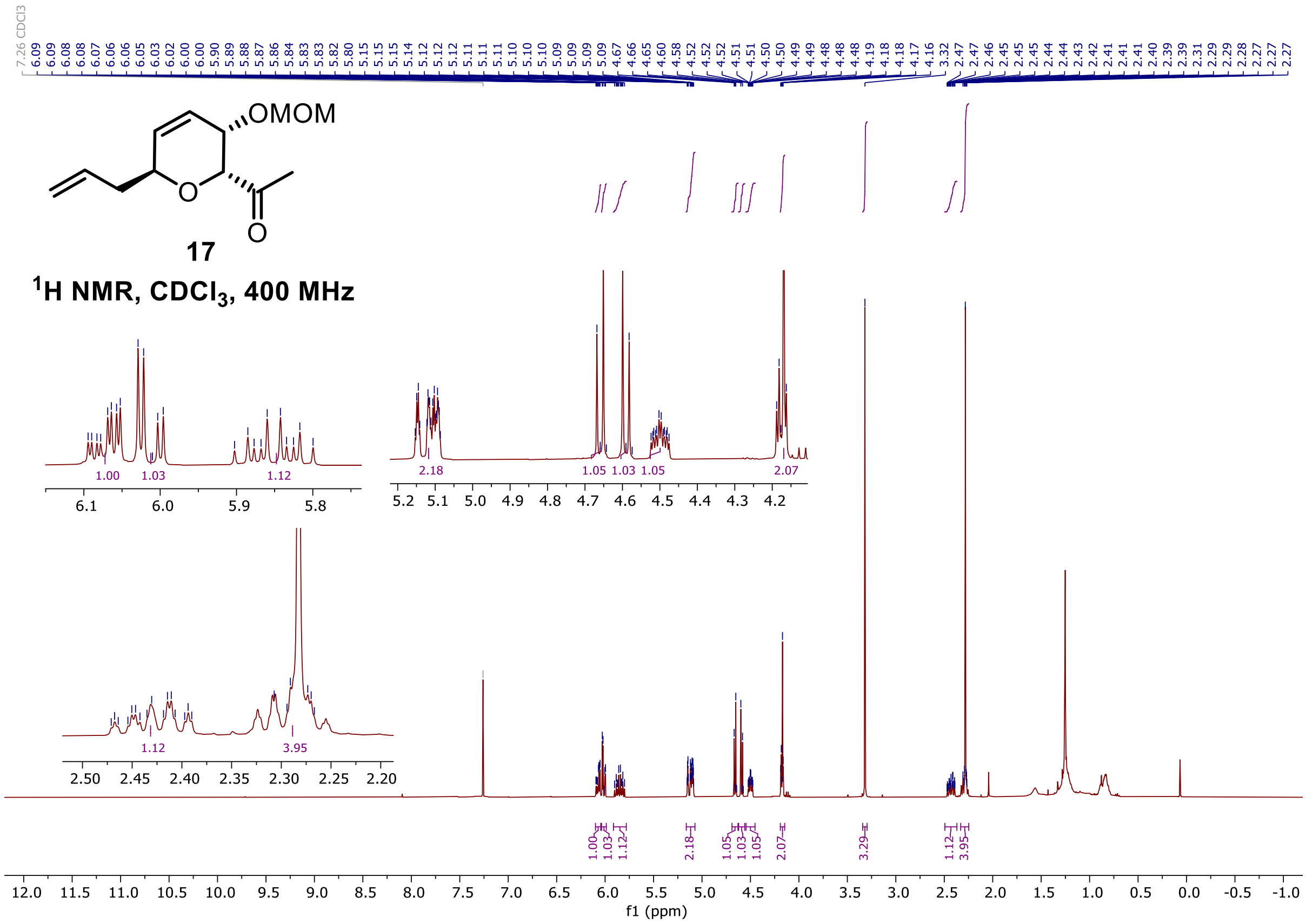
OMOM
17
1H NMR, CDCl3, 400 MHz
f1 (ppm)

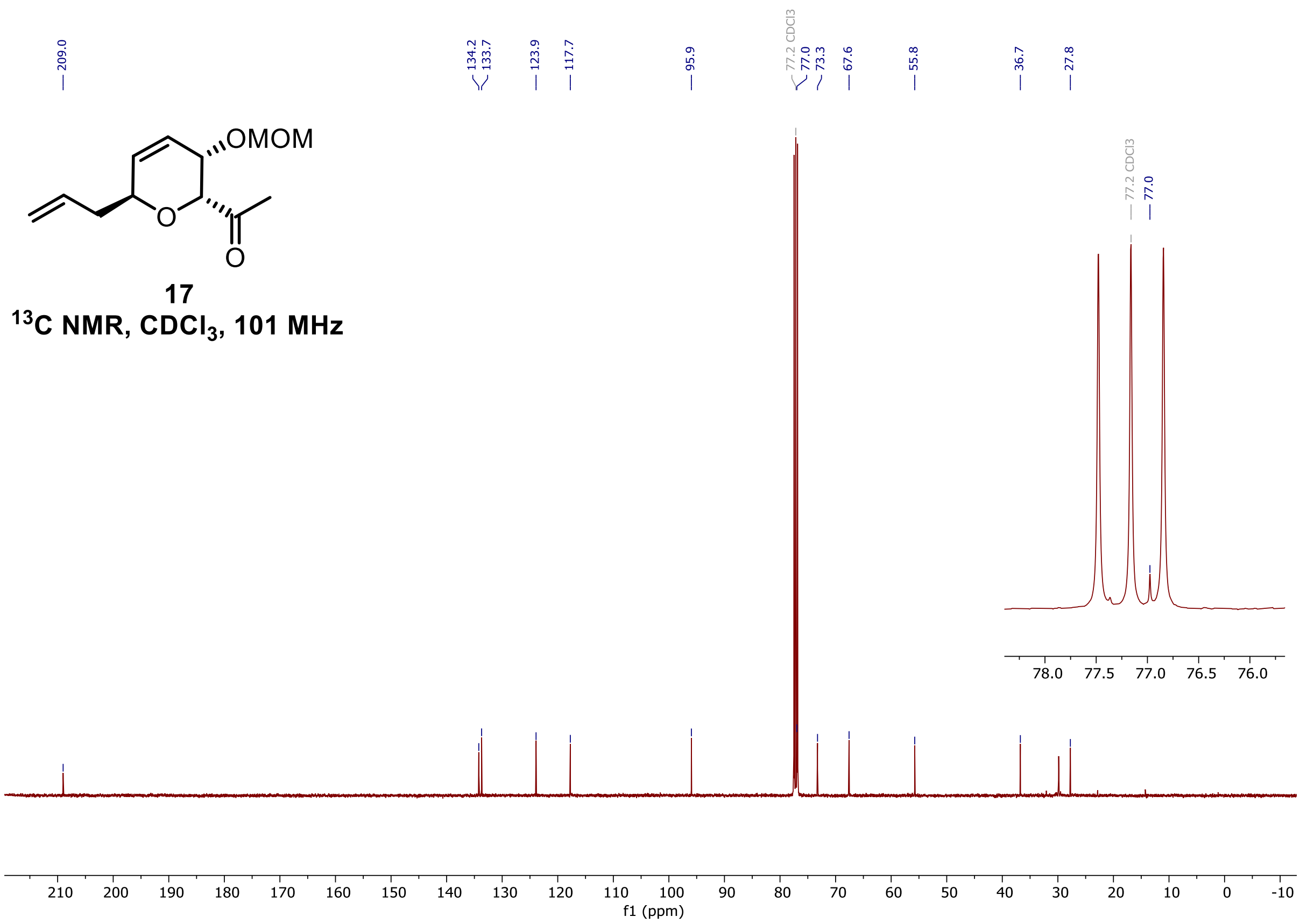
OMOM
17
13C NMR, CDCl3, 101 MHz
f1 (ppm)

Alkene 35a

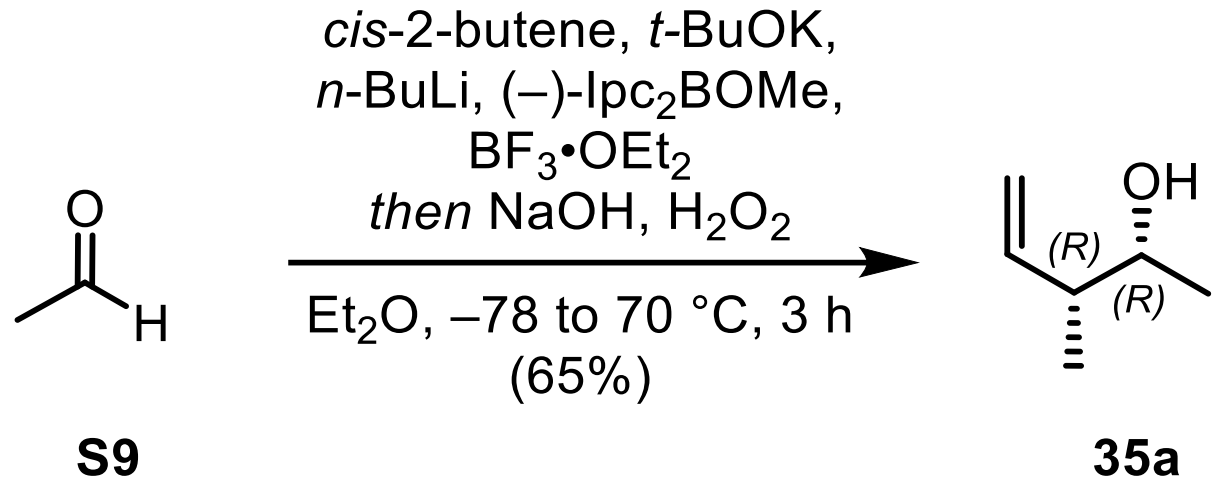

In a 250 mL three-necked flask, *cis*-2-butene (5.2 g, 92.9 mmol, 2.0 eq.) was condensed, before a –78 °C cooled solution of *t*-BuOK (5.3 g, 47.4 mmol, 1.0 eq.) in 50 mL dry THF, was added. Subsequently, *n*-BuLi (2.5M in *n*-hexane, 19.0 mL, 47.5 mmol, 1.0 eq.) was added dropwise at –78 °C. After addition, the yellow mixture was stirred at –40 °C for 20 min, before cooling again to –78 °C. Then, a solution of (–)-Ipc_2BOMe (18.0 g, 57 mmol, 1.2 eq.), dissolved in 50 mL dry Et_2O, was added dropwise while the color disappears. Next, BF_3•Et_2O (8.1 mL, 63.8 mmol, 1.35 eq.) was added in a dropwise fashion and the resulting mixture was stirred for 15 min. Acetaldehyde (**S9**) (5.3 mL, 94.8 mmol, 2.0 eq.), dissolved in 10 mL dry CH_2Cl_2, was added at –78 °C and the resulting reaction mixture was stirred for 3 h at –78 °C. After completion of the reaction, 3M NaOH (40 mL) was slowly added (exothermic!), followed by H_2O_2 solution (35% wt, 30 mL) and then heated to 70 °C for 1 h. After cooling to r.t., the mixture was diluted with Et_2O (200 mL) and the layers were separated. The organic layer was washed with water (100 mL), sat. NaCl solution (50 mL), followed by drying over Na_2SO_4. The crude material was carefully distilled with an upstream Vigreux column (60 – 100 °C for removal of solvent and remaining aldehyde, then 100 °C at 500 mbar for removal of solvent and by-products,
120 °C at 250 mbar up to 60 mbar for product collection). The product was further purified by column chromatography (pentane/Et_2O, 40:1 → 2:1) to yield the pure compound **35a** as a colorless liquid (3.1 g, 31.0 mmol, 65%).

$\mathbf{R_f}$ = 0.55 (pentane/EtOAc, 4:1), vanillin: blue/violet, UV (-).

^{1}H NMR (400 MHz, $CDCl_3$) δ [ppm] = 5.83 – 5.72 (m, 1H), 5.12 – 5.04 (m, 2H), 3.68 (p, *J* = 6.0 Hz, 1H), 2.28 – 2.18 (m, 1H), 1.58 (br, 1H), 1.34 (d, *J* = 6.4 Hz, 3H), 1.02 (d, *J* = 6.9 Hz, 3H).

^{13}C NMR (101 MHz, $CDCl_3$) δ [ppm] = 14.7, 19.9, 44.8, 70.8, 115.4, 140.4.

IR (ATR) ṽ (cm^{-1}) = 3361 (br), 3079 (w), 2970 (s), 2930 (m), 2876 (m), 1640 (w), 1454 (m), 1374 (m), 1088 (s), 1041 (m), 997 (s), 914 (s), 672 (m).

HRMS (ESI) (*m/z*) The exact mass could not be detected using various ionization methods (ESI/EI).

Specific Rotation $[\alpha]_{589}^{20}$= +21.0° (c = 2.0, $CHCl_3$).

TBS ether 35b

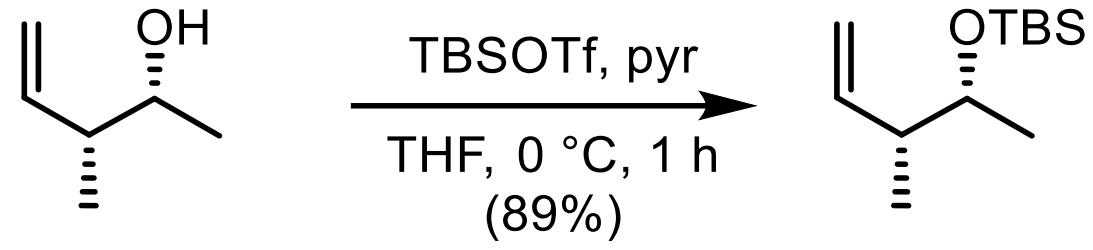

35a **35b**

A round-bottom flask was charged with alcohol **35a** (1.1 g, 11.0 mmol, 1.0 eq.), pyridine (3.5 mL, 43.9 mmol, 4.0 eq.) and 40 mL dry THF. The solution was cooled to 0 °C before TBSOTf (3.0 mL, 13.2 mmol, 1.2 eq.) was slowly added and the mixture was stirred at 0 °C for 1 h. After completion of the reaction, sat. $NaHCO_3$ solution (20 mL) was added dropwise and the mixture was stirred at r.t. for additional 30 min. The mixture was diluted with Et_2O (100 mL), the phases were separated, and the aqueous phase was extracted with Et_2O (50 mL). The organic phase was washed with water (50 mL) and sat. NaCl solution (50 mL), dried over Na_2SO_4, and the solvent was removed under reduced pressure. The crude product was purified by column chromatography (pentane) to afford TBS ether **35b** (2.1 g, 9.8 mmol, 89%) as a colorless oil.

R_f = 0.35 (pentane), vanillin: blue/violet, UV (-).

^{1}H NMR (400 MHz, $CDCl_3$) δ [ppm] = 5.80 (ddd, *J* = 7.4, 10.5, 17.2 Hz, 1H), 5.04 – 4.95 (m, 2H), 3.64 (quint, *J* = 6.2 Hz, 1H), 2.22 – 2.11 (m, 1H), 1.07 (d, *J* = 6.1 Hz, 3H), 0.98 (d, *J* = 6.8 Hz, 3H), 0.89 (s, 9H), 0.05 (d, *J* = 1.8 Hz, 6H).

^{13}C NMR (101 MHz, $CDCl_3$) δ [ppm] = -4.8, -4.3, 15.5, 18.1, 21.0, 25.9, 45.6, 72.0, 114.0, 141.6.

IR (ATR) ṽ (cm^{-1}) = 3652 (w), 2980 (s), 2927 (s), 2328 (w), 1461 (w), 1383 (m), 1260 (s), 1153 (m), 1072 (s), 1021 (m), 955 (m), 801 (s).

HRMS (ESI) (*m/z*) The exact mass could not be detected using various ionization methods (ESI/EI).

Specific Rotation $[\alpha]_{589}^{24}$ = +3.8° (c = 0.44, $CHCl_3$).

Alcohol 35c

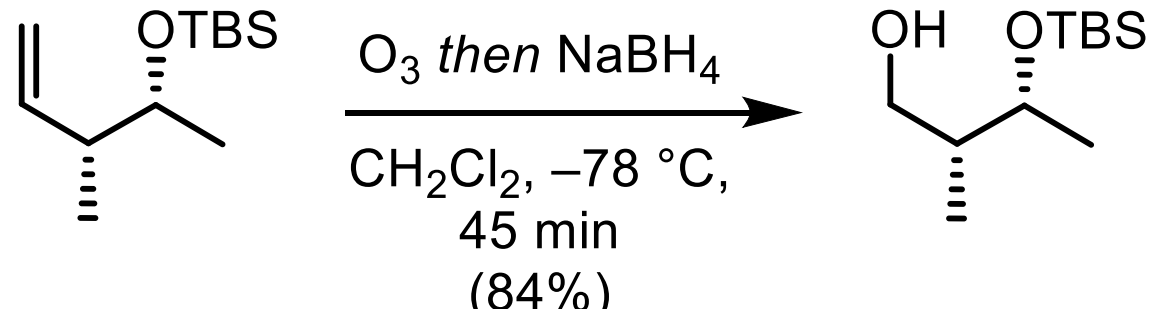

35b **35c**

TBS ether **35b** (2.1 g, 9.8 mmol, 1.0 eq.) was dissolved in 60 mL dry CH_2Cl_2 and 6 mL dry MeOH. The obtained solution was cooled to –78 °C and gassed with ozone until the solution turned blue. The mixture was then stirred for 15 min at –78 °C, before addition of $NaBH_4$ (1.1 g, 29.4 mmol, 3.0 eq.). After another 30 min of stirring at –78 °C, the mixture was slowly

warmed to 0 °C and sat. NH_4Cl (30 mL) was added dropwise. After reaching r.t., water (60 mL) was added and the layers were separated. The aqueous phase was extracted with CH_2Cl_2 (3 x 40 mL). The combined organic phase was washed with sat. NaCl solution (50 mL), dried over Na_2SO_4, and concentrated in vacuo. After purification by column chromatography (pentane/Et_2O, 3:1), alcohol **35c** (1.8 g, 8.2 mmol, 84%) was obtained as a colorless oil.

R_f = 0.50 (pentane/Et_2O, 3:1), vanillin: blue/grey, UV (-).

^{1}H NMR (400 MHz, $CDCl_3$) δ [ppm] = 3.89 (quintd, *J* = 6.3, 3.5 Hz, 1H), 3.29 (dd, *J* = 9.6, 6.5 Hz, 1H), 3.05 (dd, *J* = 9.5, 6.9 Hz, 1H), 1.66 (heptd, *J* = 6.8, 3.5 Hz, 1H), 1.02 (d, *J* = 6.3 Hz, 3H), 0.91 (d, *J* = 6.8 Hz, 3H), 0.81 (s, *J* = 9H), 0.06 (d, *J* = 4.7 Hz, 6H).

^{13}C NMR (101 MHz, $CDCl_3$) δ [ppm] = -4.8, -4.2, 13.1, 14.8, 18.0, 20.9, 25.8, 43.3, 70.1.

IR (ATR) ṽ (cm^{-1}) = 3343 (br), 2957 (m), 2930 (m), 2885 (m), 2858 (m), 1463 (m), 1379 (w), 1253 (m), 1092 (m), 1031 (s), 833 (s), 773 (s), 666 (m).

HRMS (ESI) (*m/z*) calcd. for $[C_{11}H_{26}O_2SiH]^+$ = $[M+H]^+$: 219.1780, found: 219.1779.

Specific Rotation $[\alpha]_{589}^{20}$= -8.5° (c = 0.35, $CHCl_3$).

Iodide 35

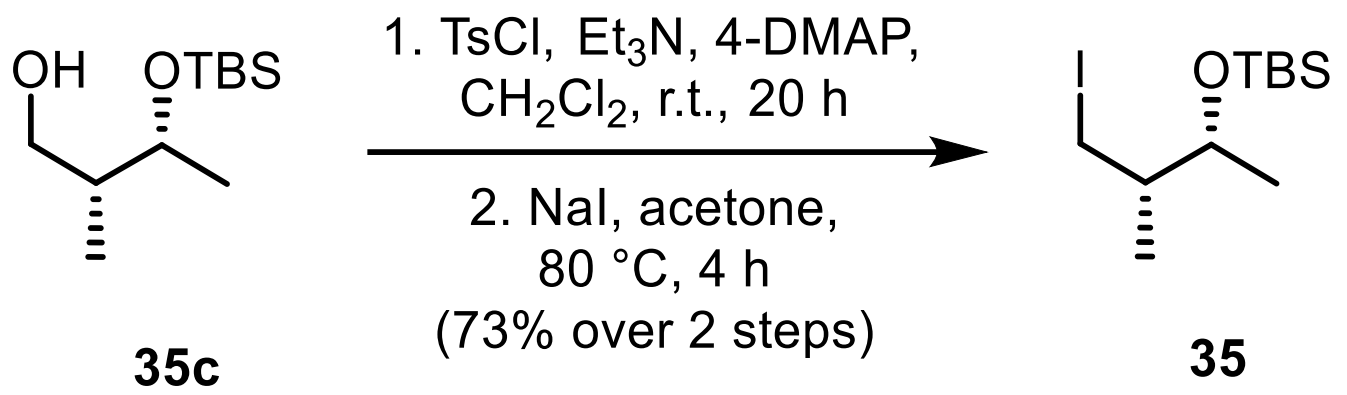

Alcohol **35c** (1.0 g, 4.58 mmol, 1.0 eq.), *p*-toluenesulfonyl chloride (1.05 g, 5.5 mmol, 1.2 eq.), Et_3N (1.3 mL, 9.3 mmol, 2.0 eq.) and 4-DMAP (56 mg, 0.46 mmol, 10 mol%) were dissolved in 60 mL dry CH_2Cl_2 and stirred for 20 h at r.t. After completion, sat. NH_4Cl solution (50 mL) was added and the mixture was stirred at r.t. for 1 h, before it was diluted with CH_2Cl_2 (200 mL). The layers were separated and the organic phase was washed with water (50 mL) and sat. NaCl solution (50 mL), then dried over Na_2SO_4 and concentrated under reduced pressure.

The crude intermediate tosylate was dissolved in 18 mL dry acetone together with NaI (3.43 g, 22.9 mmol, 5.0 eq.) and stirred at 80 °C for 4 h. After cooling, the mixture was diluted with EtOAc (200 mL) and washed with sat. $Na_2S_2O_3$ solution (50 mL), water (50 mL), sat. NaCl solution (50 mL) and dried over Na_2SO_4. The crude product was purified by column chromatography (pentane) to afford iodide **35** (1.1 g, 3.35 mmol, 73% yield over 2 steps) as a colorless liquid.

R_f = 0.35 (pentane), vanillin: light blue, UV (+).

^{1}H NMR (400 MHz, $CDCl_3$) δ [ppm] = 3.90 (qd, *J* = 6.3, 3.5 Hz, 1H), 3.30 (dd, *J* = 9.5, 6.3 Hz, 1H), 3.05 (dd, *J* = 9.5, 6.9 Hz, 1H), 1.67 (hd, *J* = 6.7, 3.5 Hz, 1H), 1.09 (d, *J* = 6.3 Hz, 3H), 0.98 (d, *J* = 6.7 Hz, 3H), 0.88 (s, 9H), 0.07 (s, 3H), 0.06 (s, 3H).

^{13}C NMR (101 MHz, $CDCl_3$) δ [ppm] = 70.2, 43.5, 26.0, 21.1, 18.2, 14.9, 13.1, -4.1, -4.6.

IR (ATR) ṽ (cm^{-1}) = 2956 (s), 2930 (s), 2886 (m), 2857 (m), 1463 (m), 1376 (m), 1253 (s), 1199 (m), 1144 (m), 1068 (s), 1016 (m), 956 (m), 835 (s), 811 (m), 774 (s), 667 (w).

HRMS (ESI) (*m/z*) The exact mass could not be detected using various ionization methods (ESI/EI).

Specific Rotation $[\alpha]_{589}^{22}$= -18.0° (c = 3.0, $CHCl_3$).

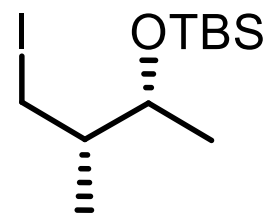

35

^{1}H NMR, $CDCl_3$, 400 MHz

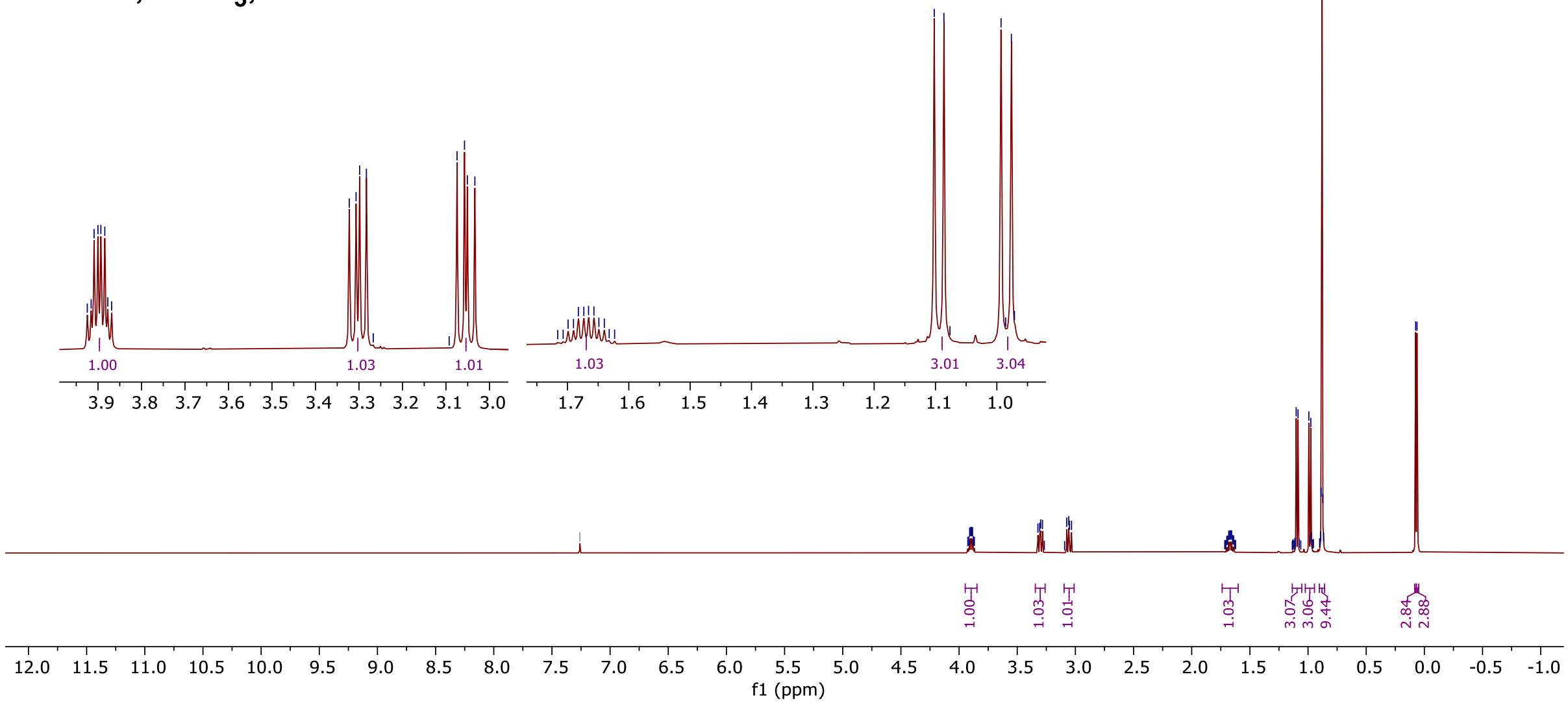

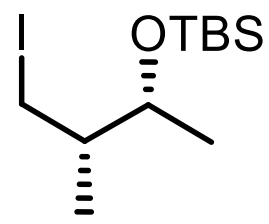

35

^{13}C NMR, $CDCl_3$, 101 MHz

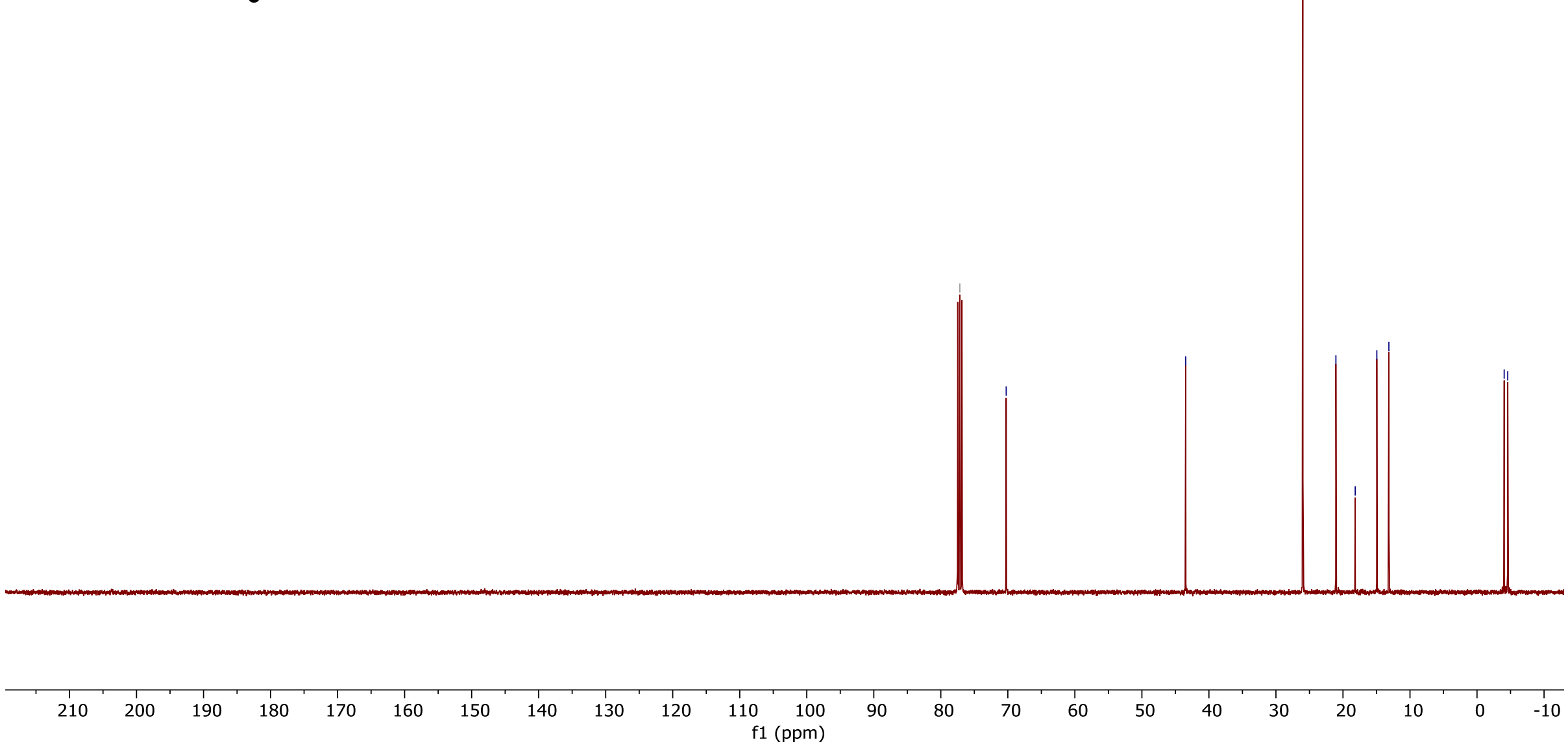

Alkene 36

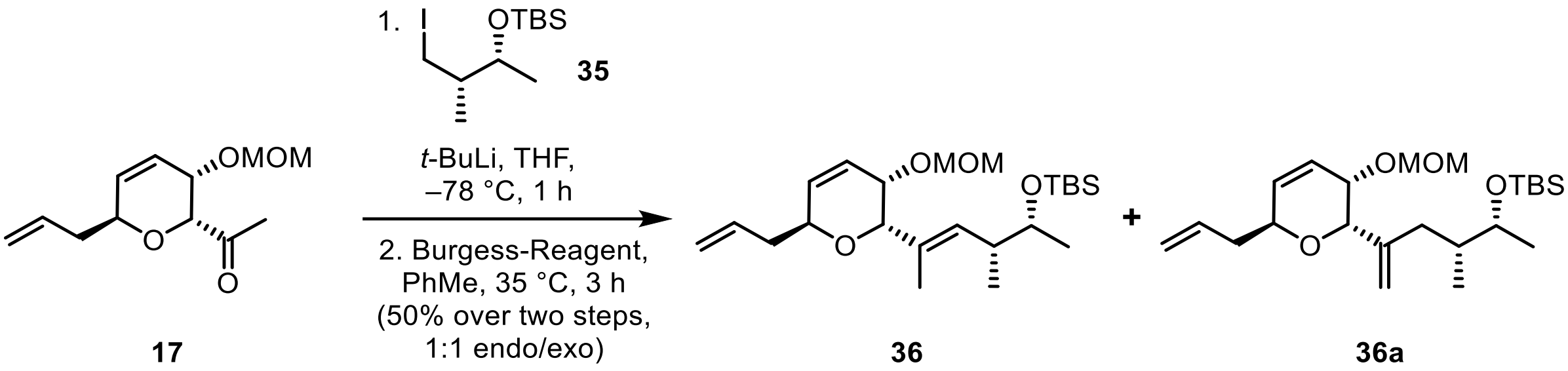

In a round-bottom flask, methyl ketone **17** (1.0 g, 4.4 mmol, 1.0 eq.) and iodide **35** (1.75 g, 5.3 mmol, 1.2 eq.) were dissolved in 50 mL dry THF and cooled to –78 °C. Next, *t*-BuLi (1.6M in pentane, 6.6 mL, 10.6 mmol, 2.4 eq.) was added slowly and the resulting mixture was stirred for 1 h at –78 °C. Subsequently, the reaction was quenched with sat. NH_4Cl solution (5 mL) and warmed to r.t. The mixture was further diluted with water and sat. NH_4Cl solution and extracted with Et_2O. The combined organic layer was washed with sat. NaCl solution, dried over Na_2SO_4, and concentrated in vacuo.
The crude alcohol mixture (~2.0 g) was dissolved in 40 mL dry toluene and treated with Burgess reagent (1.57 g, 6.6 mmol, 1.5 eq.) and heated to 35 °C. After 3 h, the reaction was quenched with water (50 mL) and sat. NaCl solution (50 mL), the layers were separated and the aqueous layer was extracted with Et_2O. The combined organic layer was dried over $MgSO_4$, concentrated in vacuo and purified by flash chromatography (pentane/Et_2O, 12:1) to afford *endo*-dihydropyran **36** (450 mg, 1.1 mmol, 25% over two steps) as a yellowish oil, along with its *exo*-isomer **36a** (25%).

$\mathbf{R_f}$ = 0.75 (pentane/Et_2O, 3:1), vanillin: violet, UV (-).

^{1}H NMR (600 MHz, $CDCl_3$) δ [ppm] = 6.04 (ddd, *J* = 10.2, 5.3, 2.1 Hz, 1H), 5.96 (dd, *J* = 10.3, 3.0 Hz, 1H), 5.84 (dddd, *J* = 16.7, 10.2, 7.7, 6.3 Hz, 1H), 5.38 (dp, *J* = 10.0, 1.4 Hz, 1H), 5.13 – 5.03 (m, 2H), 4.69 (d, *J* = 6.8 Hz, 1H), 4.57 (d, *J* = 6.8 Hz, 1H), 4.40 – 4.33 (m, 1H), 4.10 (s, 1H), 3.92 (dd, *J* = 5.3, 2.4 Hz, 1H), 3.54 (dq, *J* = 7.8, 6.1 Hz, 1H), 3.33 (s, 3H), 2.47 – 2.35 (m, 2H), 2.26 (dddt, *J* = 14.1, 7.6, 6.5, 1.1 Hz, 1H), 1.70 (s, 3H), 1.10 (d, *J* = 6.1 Hz, 3H), 0.96 (d, *J* = 6.6 Hz, 3H), 0.89 (s, 10H), 0.04 (d, *J* = 2.2 Hz, 6H).

^{13}C NMR (151 MHz, $CDCl_3$) δ [ppm] = 134.7, 133.4, 131.3, 129.6, 124.9, 117.2, 95.8, 74.6, 73.2, 73.1, 68.4, 55.6, 40.8, 37.1, 26.1, 22.3, 18.3, 17.6, 14.4, -4.1, -4.6.

IR (ATR) ṽ (cm^{-1}) = 2955 (m), 2929 (m), 2886 (m), 2857 (m), 1641 (w), 1463 (w), 1368 (w), 1253 (m), 1149 (m), 1091 (s), 1037 (s), 981 (m), 958 (m), 916 (m), 835 (s), 808 (m), 773 (s), 743 (m), 664 (w).

HRMS (ESI) (*m/z*) calcd. for $[C_{23}H_{42}O_4SiNa]^+$ = $[M+Na]^+$: 433.2756, found: 433.2760.

Specific Rotation $[\alpha]_{589}^{20}$= +171.0° (c = 0.54, $CHCl_3$).

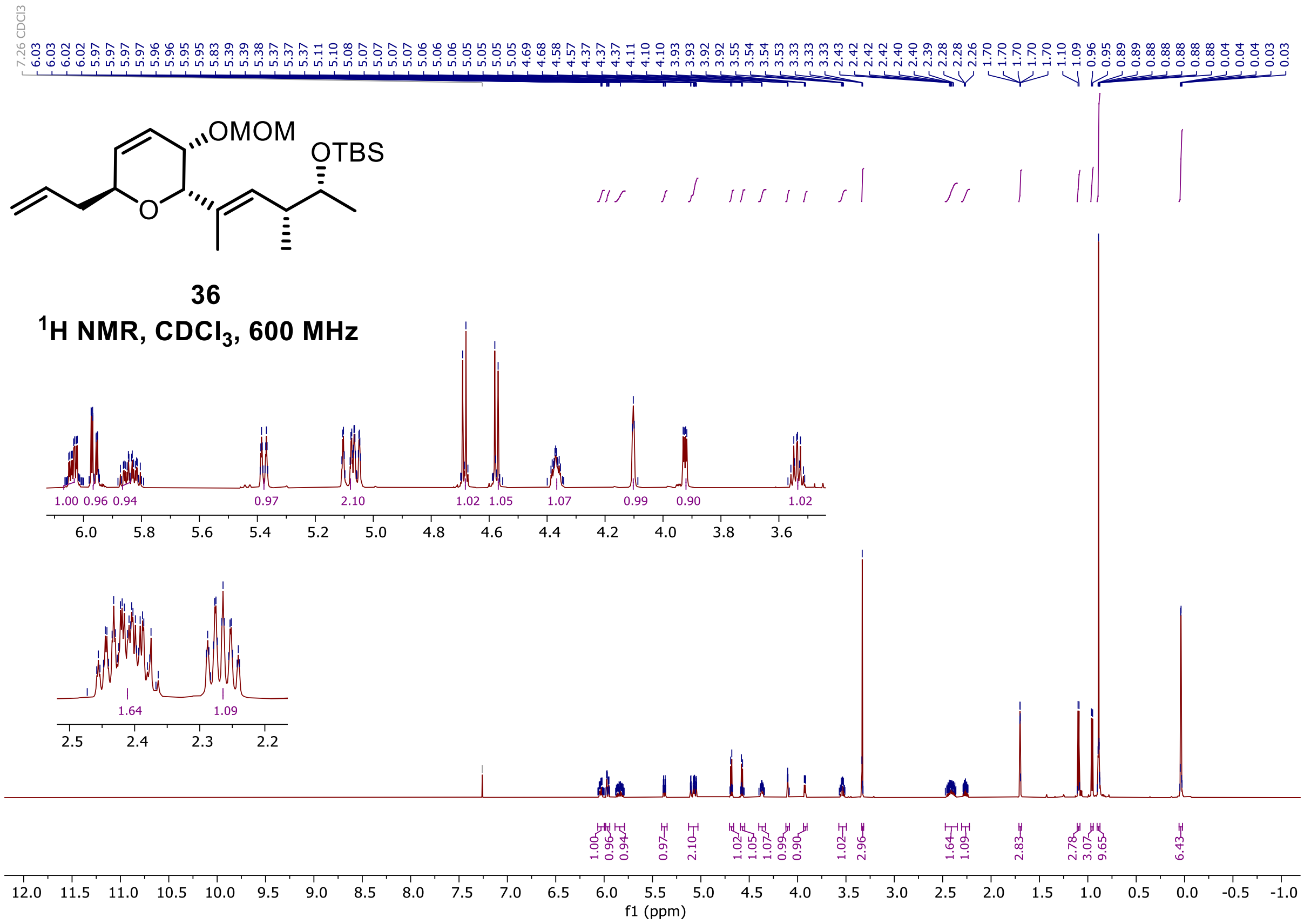

36

^{1}H NMR, $CDCl_3$, 600 MHz

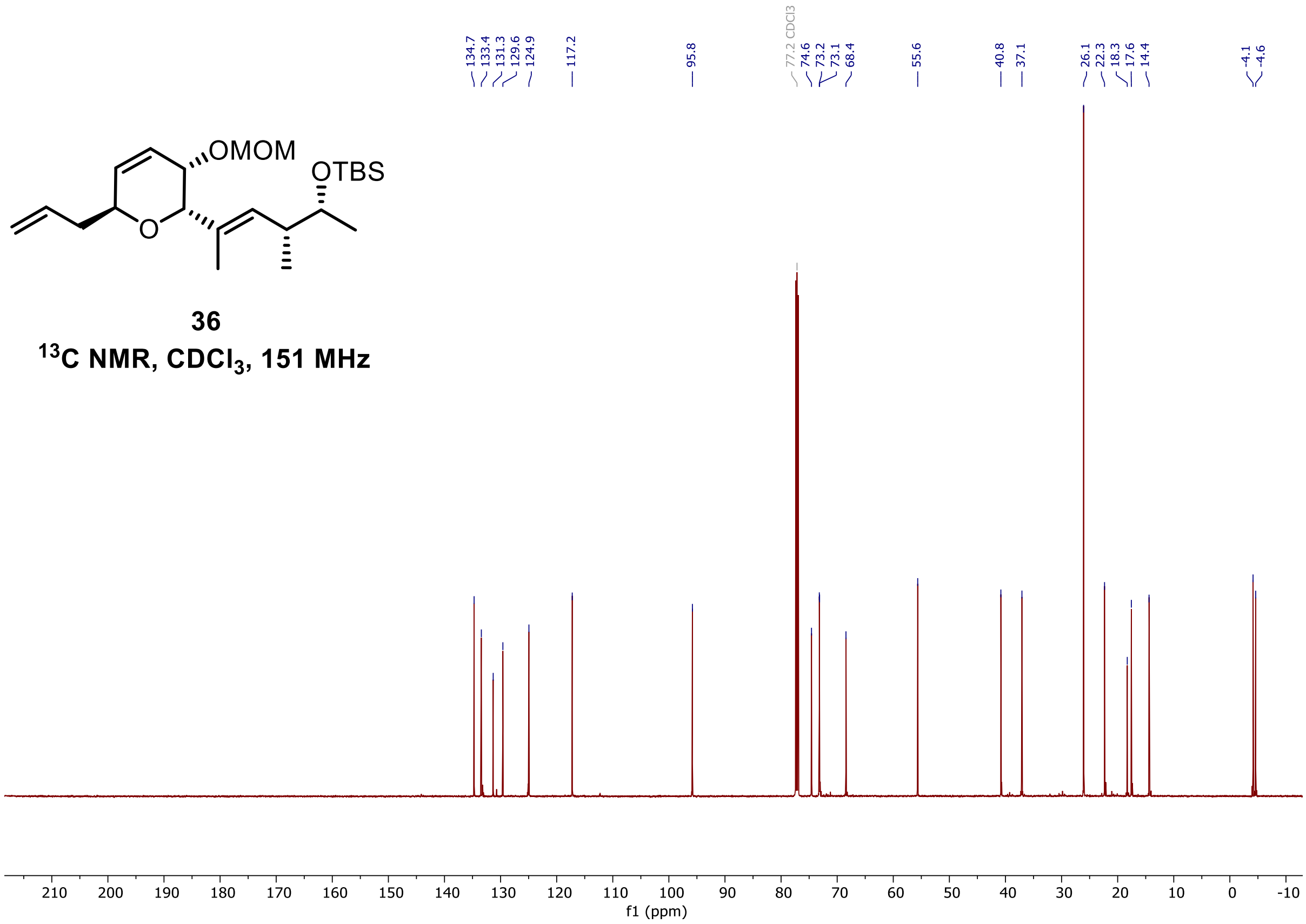

36

^{13}C NMR, $CDCl_3$, 151 MHz

TIPS ether 16

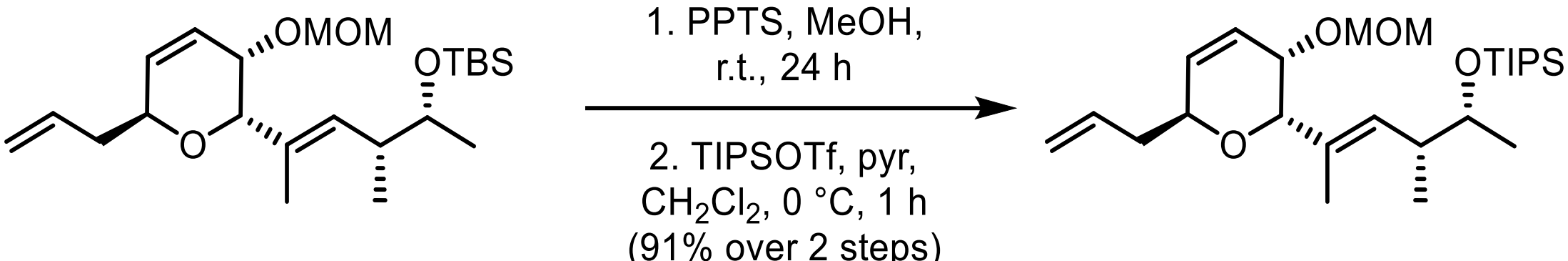

36 **16**

Dihydropyran **36** (240 mg, 0.585 mmol, 1.0 eq.) and PPTS (294 mg, 1.17 mmol, 2.0 eq,) were dissolved in 15 mL dry MeOH and stirred for 24 h at r.t. After completion of the reaction, it was diluted with EtOAc, washed with water and sat. NaCl solution, and the organic layer was dried over $MgSO_4$. The solvent was evaporated under reduced pressure to obtain the crude alcohol. The crude alcohol was dissolved in 10 mL dry CH_2Cl_2 and cooled to 0 °C, before addition of pyridine (142 µL, 1.76 mmol, 3.0 eq.) and TIPSOTf (314 µL, 1.17 mmol, 2.0 eq.). The mixture was stirred for 1 h at 0 °C, diluted with CH_2Cl_2, and the organic phase was washed with sat. NH_4Cl, water and sat. NaCl solution. After drying over $MgSO_4$ and evaporation of the solvent in vacuo, the crude product was purified by column chromatography (pentane/Et_2O, 10:1) to afford TIPS ether **16** (242 mg, 0.535 mmol, 91% over 2 steps) as a colorless oil.

R_f = 0.85 (pentane/Et_2O, 1:1), vanillin: dark blue, UV (-).

^{1}H NMR (400 MHz, $CDCl_3$) δ [ppm] = 6.04 (ddd, *J* = 10.2, 5.2, 2.0 Hz, 1H), 5.96 (dd, *J* = 10.3, 2.9 Hz, 1H), 5.84 (dddd, *J* = 16.7, 10.1, 7.7, 6.2 Hz, 1H), 5.42 (dp, *J* = 10.0, 1.4 Hz, 1H), 5.13 – 5.01 (m, 2H), 4.69 (d, *J* = 6.8 Hz, 1H), 4.58 (d, *J* = 6.8 Hz, 1H), 4.37 (ddt, *J* = 9.8, 6.5, 2.5 Hz, 1H), 4.12 (br s, 1H), 3.92 (dd, *J* = 5.1, 2.4 Hz, 1H), 3.81 – 3.70 (m, 1H), 3.34 (s, 3H), 2.51 – 2.37 (m, 2H), 2.27 (dddt, *J* = 14.1, 7.7, 6.5, 1.1 Hz, 1H), 1.72 (d, *J* = 1.3 Hz, 3H), 1.14 (d, *J* = 6.1 Hz, 3H), 1.07 (s, 22H), 1.00 (d, *J* = 6.7 Hz, 3H).

^{13}C NMR (101 MHz, $CDCl_3$) δ [ppm] = 134.8, 133.4, 131.4, 129.7, 125.0, 117.2, 95.9, 74.7, 73.2, 73.2, 68.7, 55.6, 41.0, 37.1, 22.2, 18.4, 18.4, 17.8, 17.4, 14.3, 12.9, 12.4.

IR (ATR) ṽ (cm^{-1}) = 2942 (s), 2891 (m), 2866 (s), 2328 (w), 1641 (w), 1463 (m), 1371 (m), 1149 (m), 1103 (s), 1038 (s), 996 (m), 956 (w), 916 (m), 882 (s), 837 (w), 739 (m), 675 (s).

HRMS (ESI) (*m/z*) calcd. for $[C_{26}H_{48}O_4SiH]^+$ = $[M+H]^+$: 453.3400, found: 453.3396.

Specific Rotation $[\alpha]^{20}_{589}$= +140.0° (c = 0.4, $CHCl_3$).

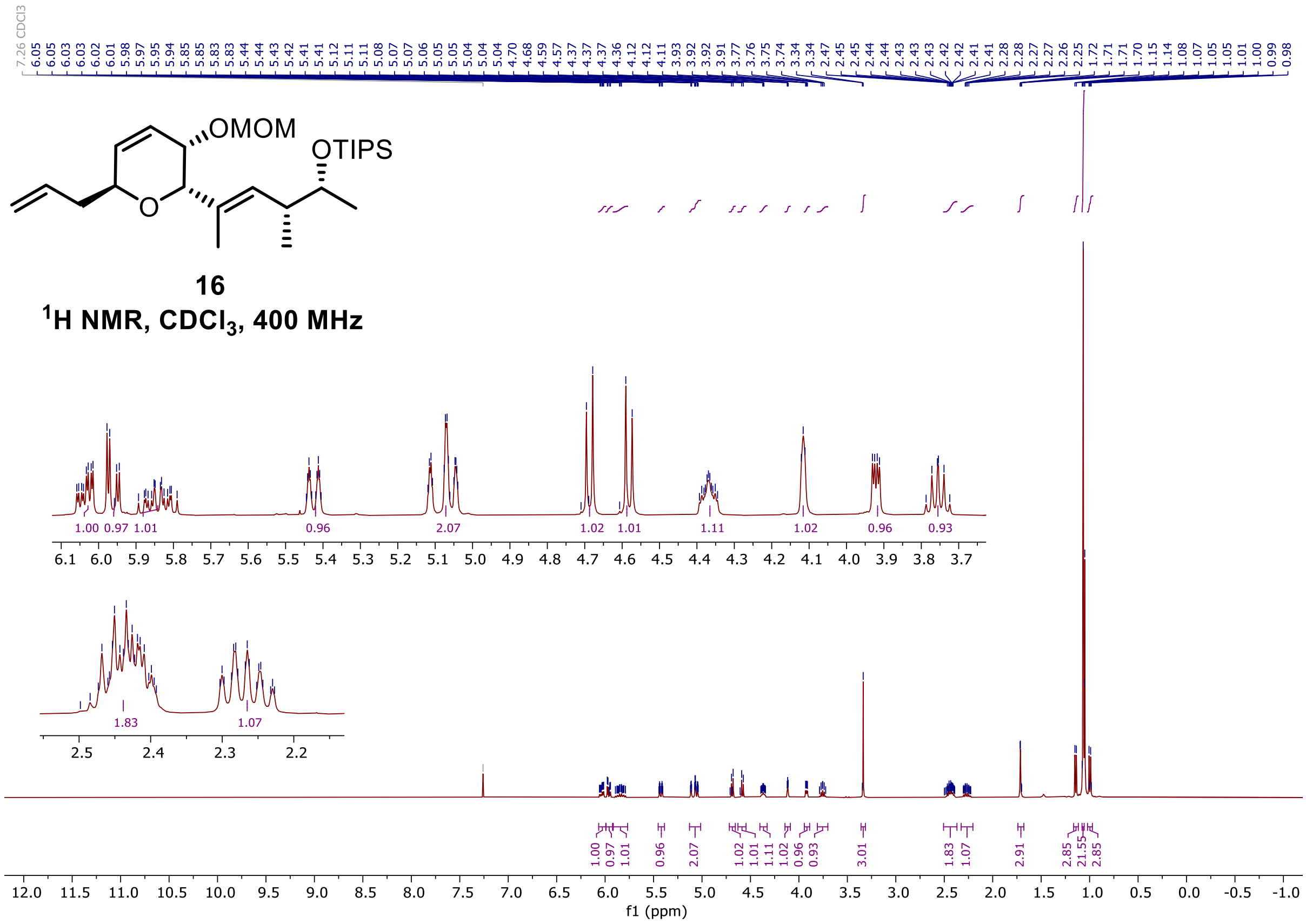

16

^{1}H NMR, CDCl$_3$, 400 MHz

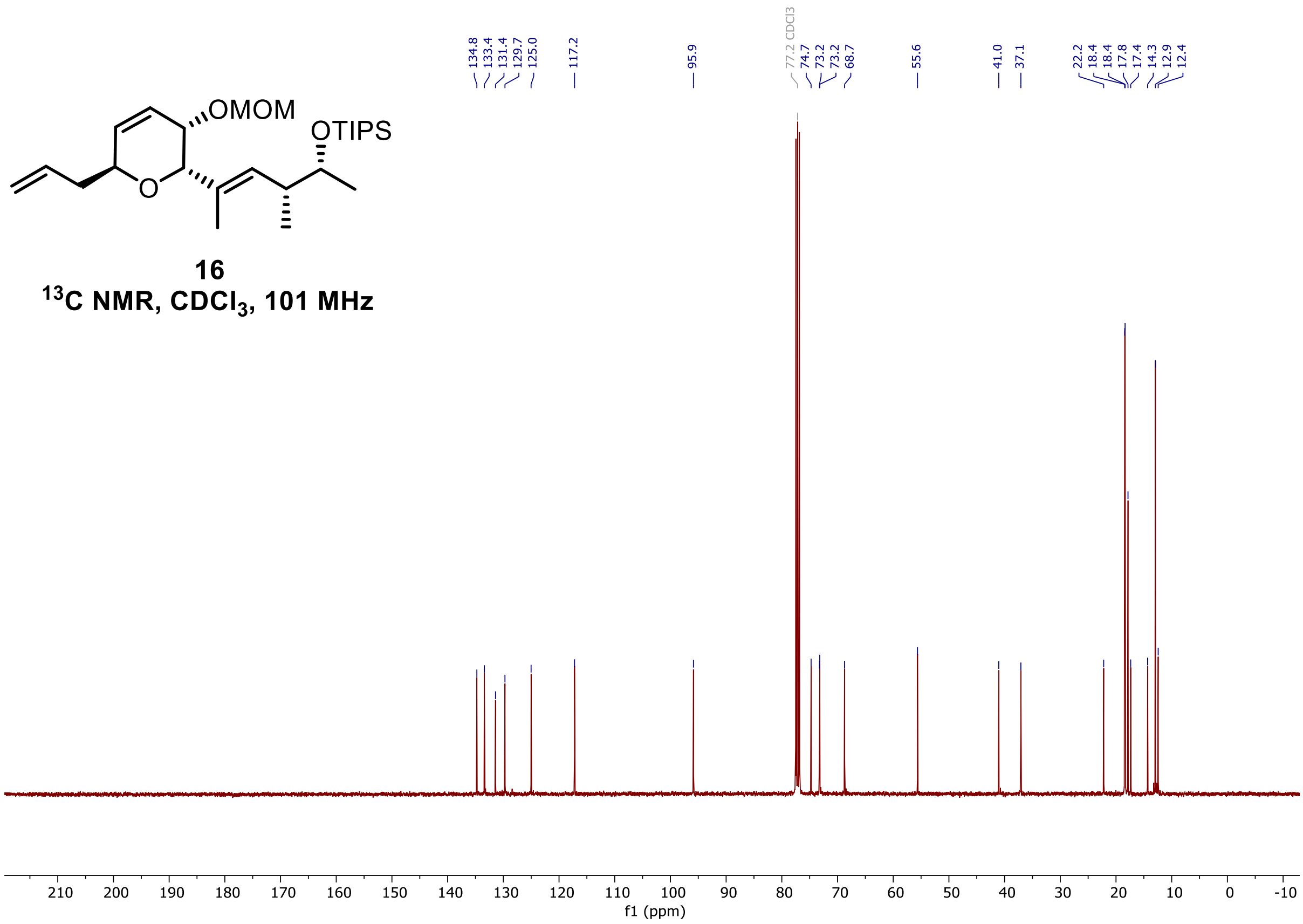

16

^{13}C NMR, CDCl$_3$, 101 MHz

Alcohol 38a

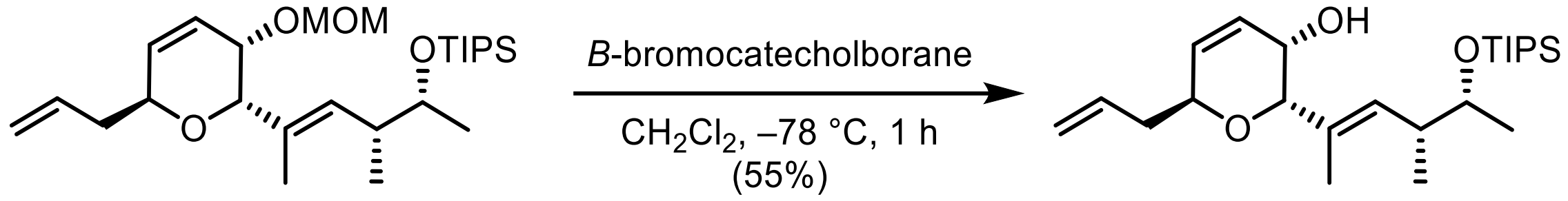

16 **38a**

TIPS ether **16** (130 mg, 0.287 mmol, 1.0 eq.) was dissolved in 5 mL dry CH_2Cl_2 and cooled to –78 °C. Subsequently, a solution of *B*-bromochatecholborane (114 mg, 0.573 mmol, 2.0 eq.), dissolved in 0.8 mL dry CH_2Cl_2, was added dropwise. The reaction was quenched after 1 h with water and extracted with CH_2Cl_2. The combined organic phase was washed with sat. NaCl solution, dried over $MgSO_4$ and the solvent was removed under reduced pressure. The crude material was purified by column chromatography (pentane/Et_2O, 4:1) to afford alcohol **38a** (64 mg, 0.157 mmol, 55%) as a colorless liquid.

Note: The yields differed from 50 – 65%, but only the 55% yield was reproducible.

$\mathbf{R_f}$ = 0.50 (pentane/Et_2O 2:1), vanillin: violet, UV (-).

^{1}H NMR (400 MHz, $CDCl_3$) δ [ppm] = 6.09 (ddd, *J* = 10.1, 5.6, 2.0 Hz, 1H), 5.94 (dd, *J* = 10.1, 3.2 Hz, 1H), 5.85 (dddd, *J* = 16.7, 10.2, 7.8, 6.3 Hz, 1H), 5.54 (dp, *J* = 10.2, 1.5 Hz, 1H), 5.15 – 5.03 (m, 2H), 4.35 (dddd, *J* = 8.2, 6.6, 3.1, 2.1 Hz, 1H), 4.09 (br s, 1H), 3.88 (dd, *J* = 5.6, 2.0 Hz, 1H), 3.81 (p, *J* = 6.1 Hz, 1H), 2.56 (dp, *J* = 10.1, 6.7 Hz, 1H), 2.46 (dddt, *J* = 14.1, 7.8, 6.2, 1.5 Hz, 1H), 2.35 – 2.23 (m, 1H), 1.69 (d, *J* = 1.4 Hz, 3H), 1.14 (d, *J* = 6.1 Hz, 3H), 1.06 (s, 21H), 1.00 (d, *J* = 6.8 Hz, 3H).

^{13}C NMR (101 MHz, $CDCl_3$) δ [ppm] = 134.7, 132.8, 131.0, 129.4, 126.5, 117.3, 74.5, 73.6, 73.1, 62.2, 40.4, 37.1, 21.0, 18.4, 18.3, 17.8, 17.3, 14.8, 12.8.

IR (ATR) ṽ (cm^{-1}) = 3455 (br), 2942 (s), 2866 (s), 2328 (w), 1641 (w), 1462 (m), 1372 (m), 1250 (w), 1183 (w), 1136 (m), 1082 (s), 1051 (s), 1026 (s), 953 (m), 915 (m), 882 (s), 779 (w), 742 (s), 676 (s), 460 (w).

HRMS (ESI) (*m/z*) calcd. for $[C_{24}H_{44}O_3SiH]^+$ = $[M+H]^+$: 409.3138, found: 409.3130.

Specific Rotation $[\alpha]_{589}^{20}$= +147.0° (c = 1.15, $CHCl_3$).

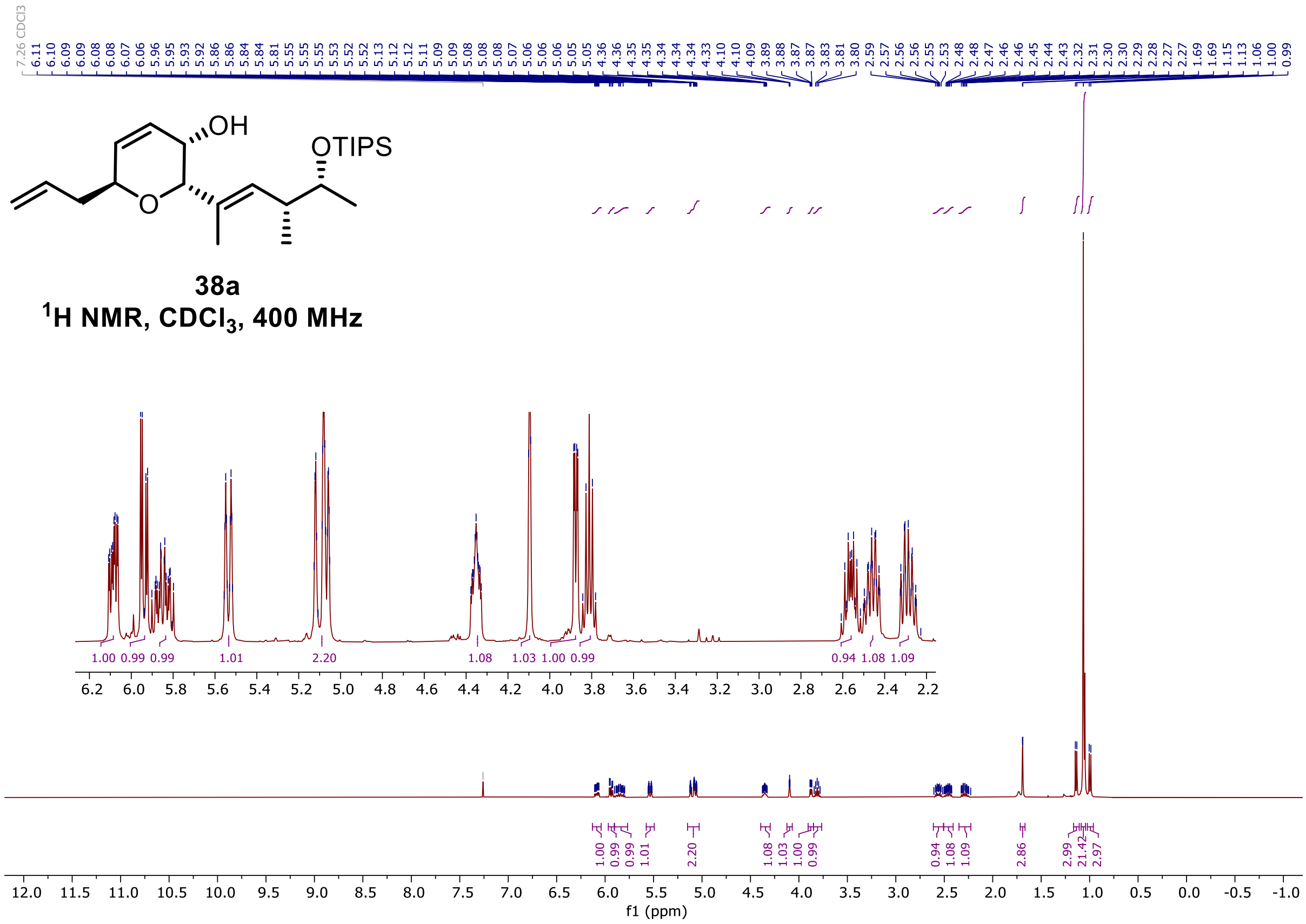

38a

^{1}H NMR, $CDCl_3$, 400 MHz

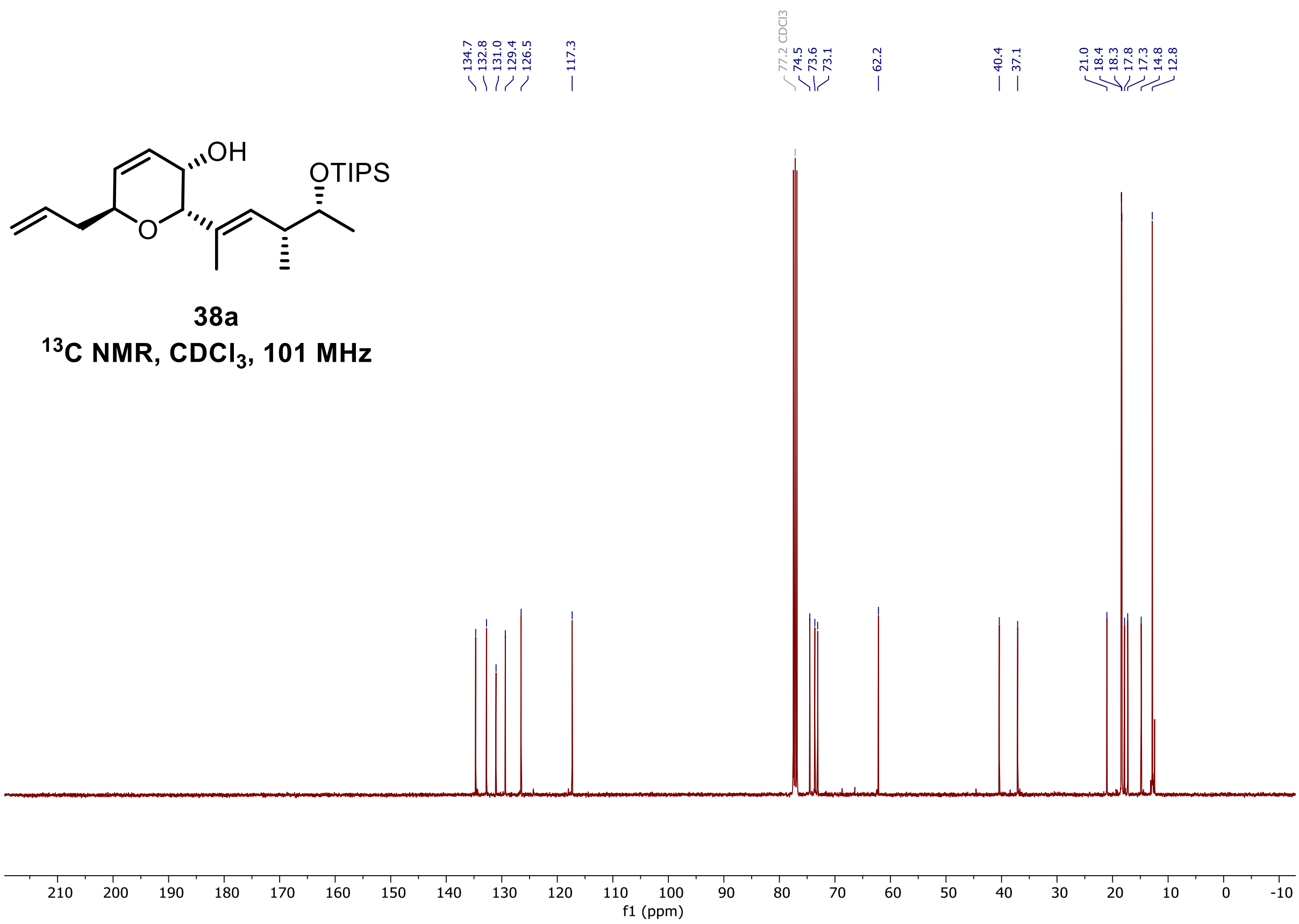

38a

^{13}C NMR, $CDCl_3$, 101 MHz

Benzyl-2-(diphenoxyphosphoryl)acetate (37b)

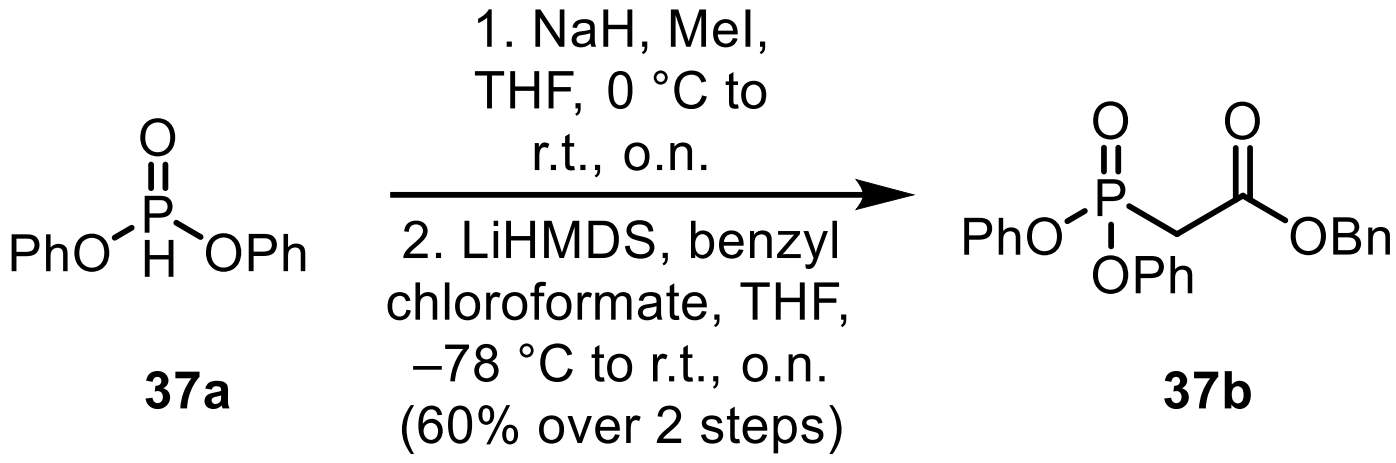

To a 0 °C cooled suspension of NaH (60% in mineral oil, 200 mg, 5.0 mmol, 1.15 eq.) in 10 mL dry THF, was added diphenyl phosphite (**37a**) (0.82 mL, 4.3 mmol, 1.0 eq.) and the mixture was stirred for 30 min. Next, iodomethane (0.52 mL, 8.4 mmol, 1.95 eq.) was added dropwise and the resulting mixture was stirred overnight at r.t. The reaction was quenched with sat. NH_4Cl solution, the layers were separated and the aqueous phase was extracted with Et_2O. The combined organic layer was washed with sat. NaCl solution, dried over $MgSO_4$, and concentrated in vacuo.
The crude material, along with benzyl chloroformate (0.92 mL, 6.6 mmol, 1.5 eq.) was dissolved in 15 mL dry THF and cooled to –78 °C. LiHMDS (1.0M in THF, 8.6 mL, 8.6 mmol, 2.0 eq.) was added slowly, and the mixture was allowed to warm to r.t. and stirred overnight. The reaction was quenched with sat. $NaHCO_3$ solution and the aqueous layer was extracted with Et_2O. The combined organic layer was washed with sat. NaCl solution, dried over $MgSO_4$ and concentrated in vacuo. The residue was purified by column chromatography (pentane/Et_2O, 1:1) to yield benzyl-2-(diphenoxyphosphoryl)acetate (**37b**) (980 mg, 2.6 mmol, 60% over 2 steps) as a colorless oil.

R_f = 0.24 (pentan/Et_2O, 1:1), UV (+), $KMnO_4$ (+).

^{1}H NMR (400 MHz, $CDCl_3$) δ [ppm] = 7.39 – 7.24 (m, 10 H), 7.21 – 7.09 (m, 5H), 5.20 (s, 2H), 3.29 (d, *J* = 21.6 Hz, 2H).

^{13}C NMR (101 MHz, $CDCl_3$) δ [ppm] = 164.8, 150.1, 135.2, 129.9 (d, *J* = 1.1 Hz), 128.7, 128.6 (d, *J* = 1.4 Hz), 125.7 (d, *J* = 1.4 Hz), 120.8, 120.7, 67.8, 34.8.

IR (ATR) ṽ (cm^{-1}) = 1735 (s), 1590 (m), 1488 (s), 1455 (w), 1396 (w), 1282 (s), 1209 (s), 1185 (s), 1161 (s), 1110 (s), 1025 (m), 933 (s), 826 (w), 762 (s), 690 (s), 616 (w), 585 (w), 494 (m).

HRMS (ESI) (*m/z*) calcd. for $[C_{21}H_{19}O_5PH]^+$ = $[M+H]^+$: 383.1048, found: 383.1081.

2-(Diphenoxyphosphoryl)aceticacid (37)

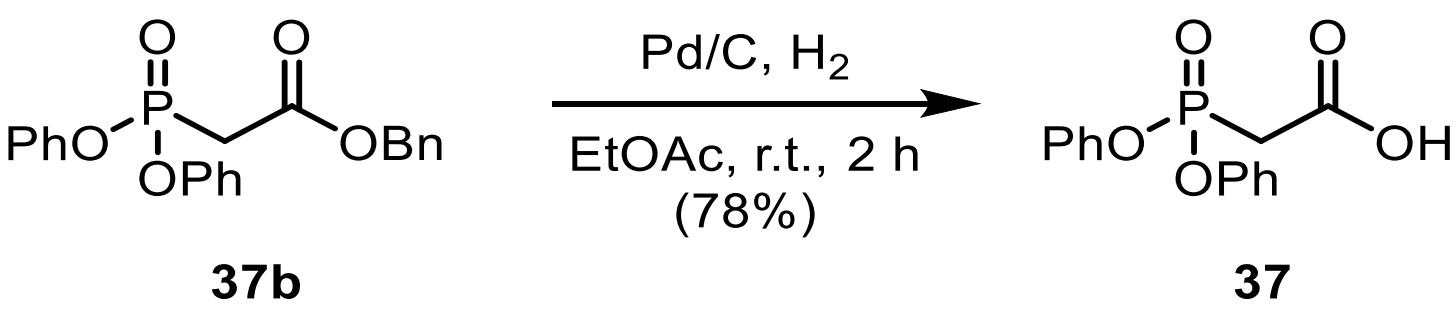

To a solution of benzyl-2-(diphenoxyphosphoryl)acetate (**37b**) (390 mg, 1.02 mmol, 1.0 eq.) in 5 mL EtOAc was added Pd/C (20% wt, 109 mg, 102 µmol, 10 mol%) and purged with hydrogen.

The mixture was stirred for 2 h under hydrogen atmosphere, before it was filtered through a silica pad and the solvent was removed under reduced pressure. After re-crystallization from EtOAc/MeOH (3:1), 2-(diphenoxyphosphoryl)acetic acid (**37**) (235 mg, 804 µmol, 78%) was obtained as colorless crystals.

R$_f$ = 0.50 (Et_2O), UV (+), $KMnO_4$ (+).

^{1}H NMR (400 MHz, methanol-d_4) δ [ppm] = 7.43 – 7.32 (m, 5H), 7.28 – 7.17 (m, 5H), 3.41 (dd, *J* = 21.7, 5.1 Hz, 2H).

^{13}C NMR (101 MHz, methanol-d_4) δ [ppm] = 167.8, 151.3, 131.01 (d, *J* = 1.2 Hz), 126.87 (d, *J* = 1.5 Hz), 121.86 (d, *J* = 4.4 Hz), P*C*H$_2$COOH not detectable.

IR (ATR) ṽ (cm^{-1}) = 1724 (s), 1587 (m), 1485 (s), 1421 (w), 1365 (w), 1304 (m), 1235 (s), 1189 (s), 1177 (s), 1148 (s), 1070 (w), 1023 (w), 1007 (w), 937 (s), 904 (s), 820 (w), 766 (s), 691 (s), 596 (s), 572 (m), 493 (s), 460 (m), 417 (w).

HRMS (ESI) (*m/z*) calcd. for $[C_{14}H_{13}O_5PH]^+$ = $[M+H]^+$: 293.0579, found: 293.0534.

Ester 38

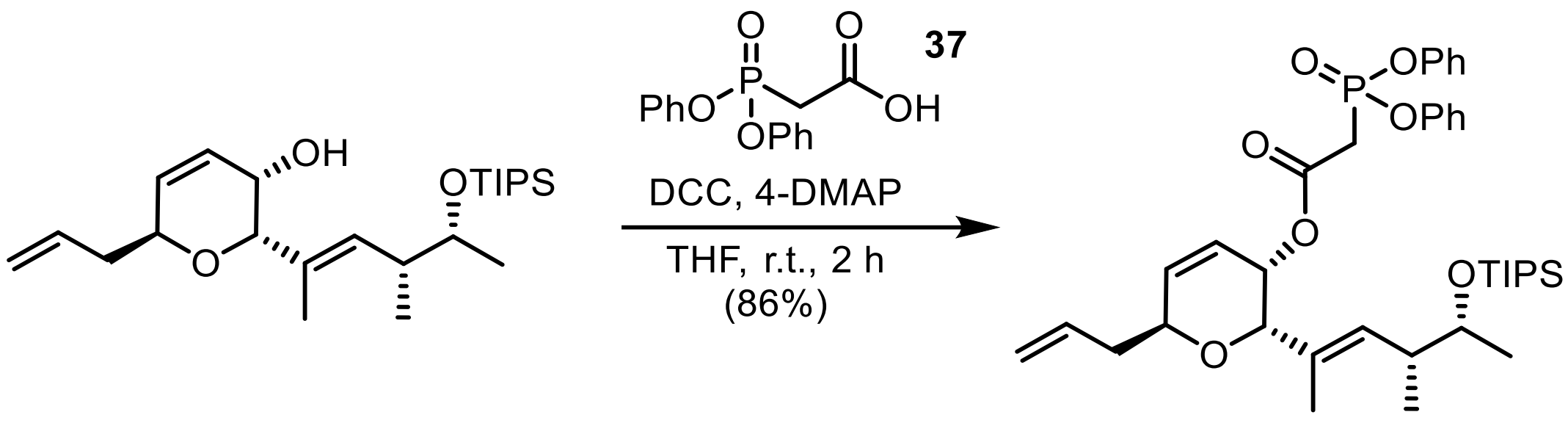

38a 38

Under a nitrogen atmosphere, alcohol **38a** (63 mg, 154 µmol, 1.0 eq.), 2-(diphenoxyphosphoryl)acetic acid (**37**) (67 mg, 230 µg, 1.5 eq.), DCC (47 mg, 228 µmol, 1.5 eq.) and 4-DMAP (2 mg, 16 µmol, 10 mol%) were dissolved in 3 mL dry THF. The solution was stirred for 2 h at r.t. After completion, the reaction was quenched with water and extracted with Et_2O. The combined organic phase was washed with sat. NaCl solution, dried over $MgSO_4$ and the solvent removed under reduced pressure. Th crude material was purified by column chromatography (pentane/Et_2O, 3:2) to afford dihydropyran **38** (90 mg, 132 µmol, 86%) as a colorless, highly viscous oil.

R_f = 0.32 (pentane/Et_2O, 3:2), vanillin: blue, UV (+).

^{1}H NMR (400 MHz, $CDCl_3$) δ [ppm] = 7.36 – 7.27 (m, 4H), 7.25 – 7.14 (m, 6H), 6.11 – 6.00 (m, 2H), 5.84 (dddd, *J* = 16.6, 10.1, 7.7, 6.3 Hz, 1H), 5.43 (dt, *J* = 10.0, 1.5 Hz, 1H), 5.22 (dd, *J* = 4.5, 2.2 Hz, 1H), 5.16 – 5.04 (m, 2H), 4.39 (ddt, *J* = 8.2, 6.5, 1.6 Hz, 1H), 4.24 (t, *J* = 2.0 Hz, 1H), 3.75 – 3.63 (m, 1H), 3.36 – 3.09 (m, 2H), 2.46 (dddt, *J* = 14.4, 8.0, 6.4, 1.6 Hz, 1H), 2.38 (dt, *J* = 10.0, 6.8 Hz, 1H), 2.34 – 2.23 (m, 1H), 1.65 (s, 3H), 1.10 (d, *J* = 7.4 Hz, 3H), 1.05 (s, 21H), 0.96 (d, *J* = 6.7 Hz, 3H).

^{13}C NMR (101 MHz, $CDCl_3$) δ [ppm] = 164.7 (d, *J* = 6.2 Hz), 150.0 (d, *J* = 2.0 Hz), 149.9 (d, *J* = 2.4 Hz), 125.8, 125.7, 125.7, 125.7, 125.6, 122.0, 121.0, 120.9, 120.9, 120.9, 120.9, 120.8, 117.6, 73.3, 73.0, 73.0, 67.0, 41.0, 36.7, 33.8 (d, *J* = 138.3 Hz), 22.1, 18.4, 18.3, 17.8, 17.4, 14.1, 12.9, 12.4, 12.0.

IR (ATR) ṽ (cm^{-1}) = 2941 (m), 2865 (m), 1733 (s), 1591 (m), 1489 (s), 1461 (m), 1386 (w), 1282 (s), 1185 (s), 1161 (s), 1109 (s), 1025 (m), 940 (s), 883 (m), 826 (w), 762 (s), 732 (s), 687 (s), 649 (m), 570 (w), 494 (m).

HRMS (ESI) (*m/z*) calcd. for $[C_{38}H_{55}O_7PSiNH_4]^+$ = $[M+NH_4]^+$: 700.3798, found: 700.3791.

Specific Rotation $[\alpha]_{589}^{20}$= +121.0° (c = 0.53, $CHCl_3$).

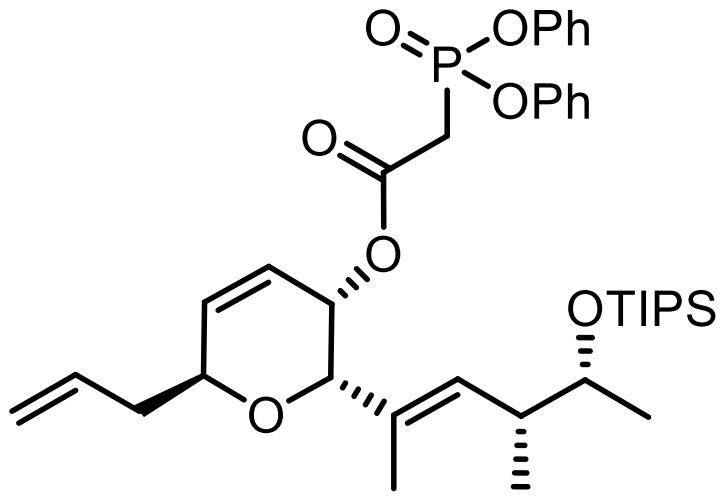

38

^{1}H NMR, CDCl$_3$, 400 MHz

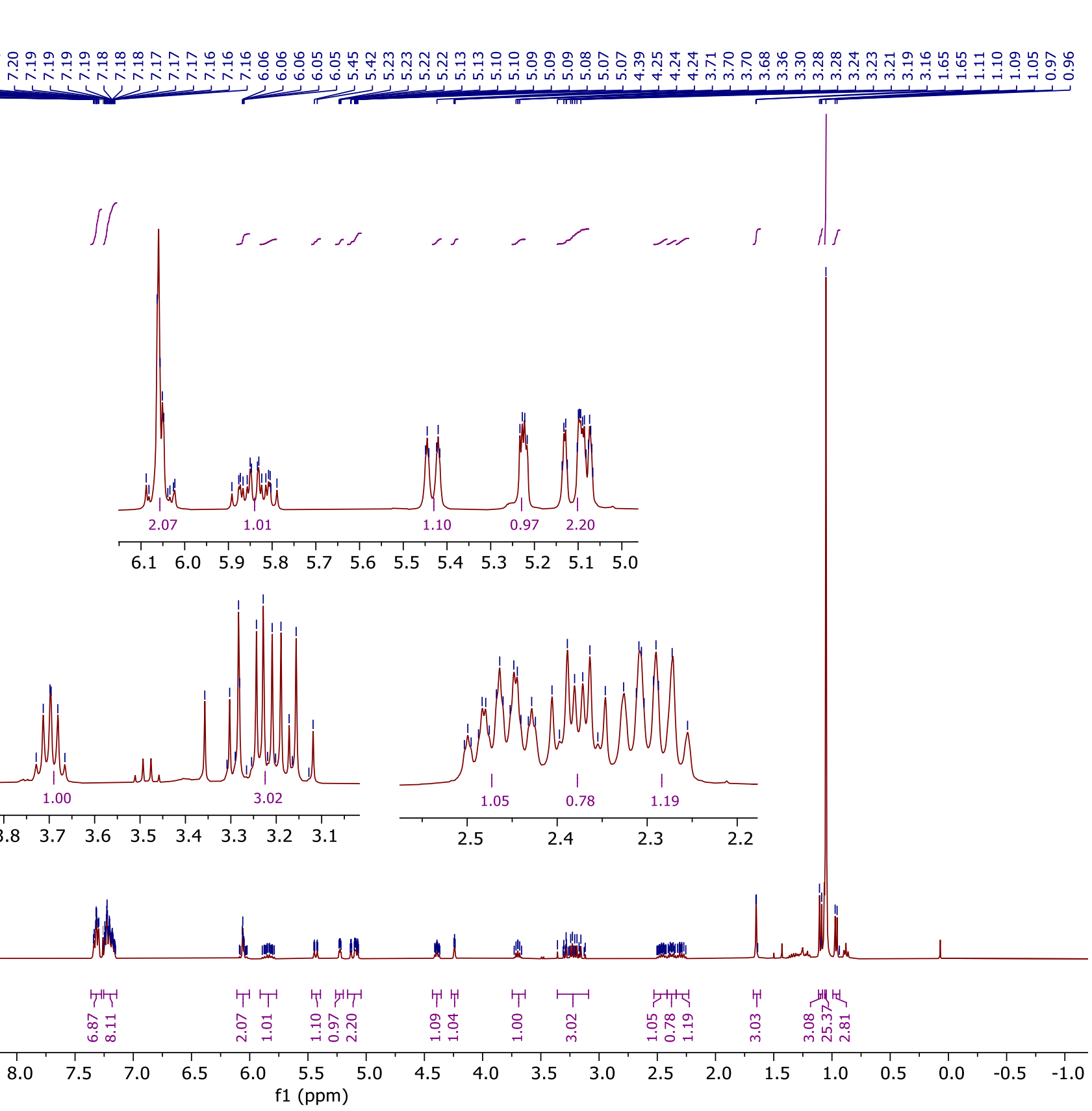

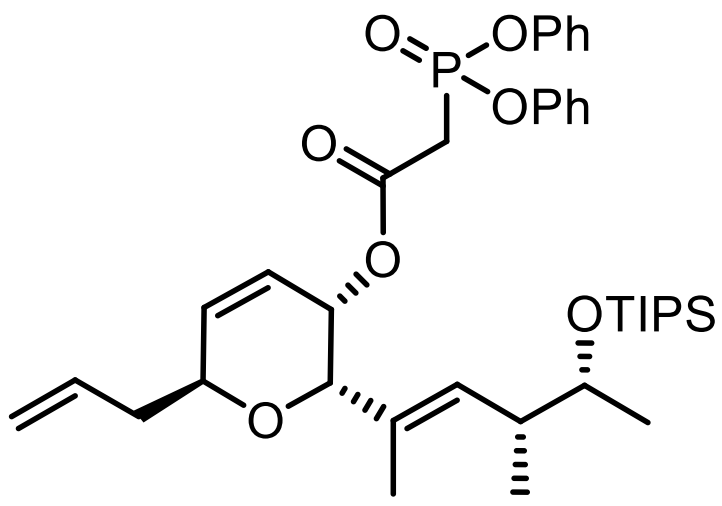

38

^{13}C NMR, CDCl$_3$, 101 MHz

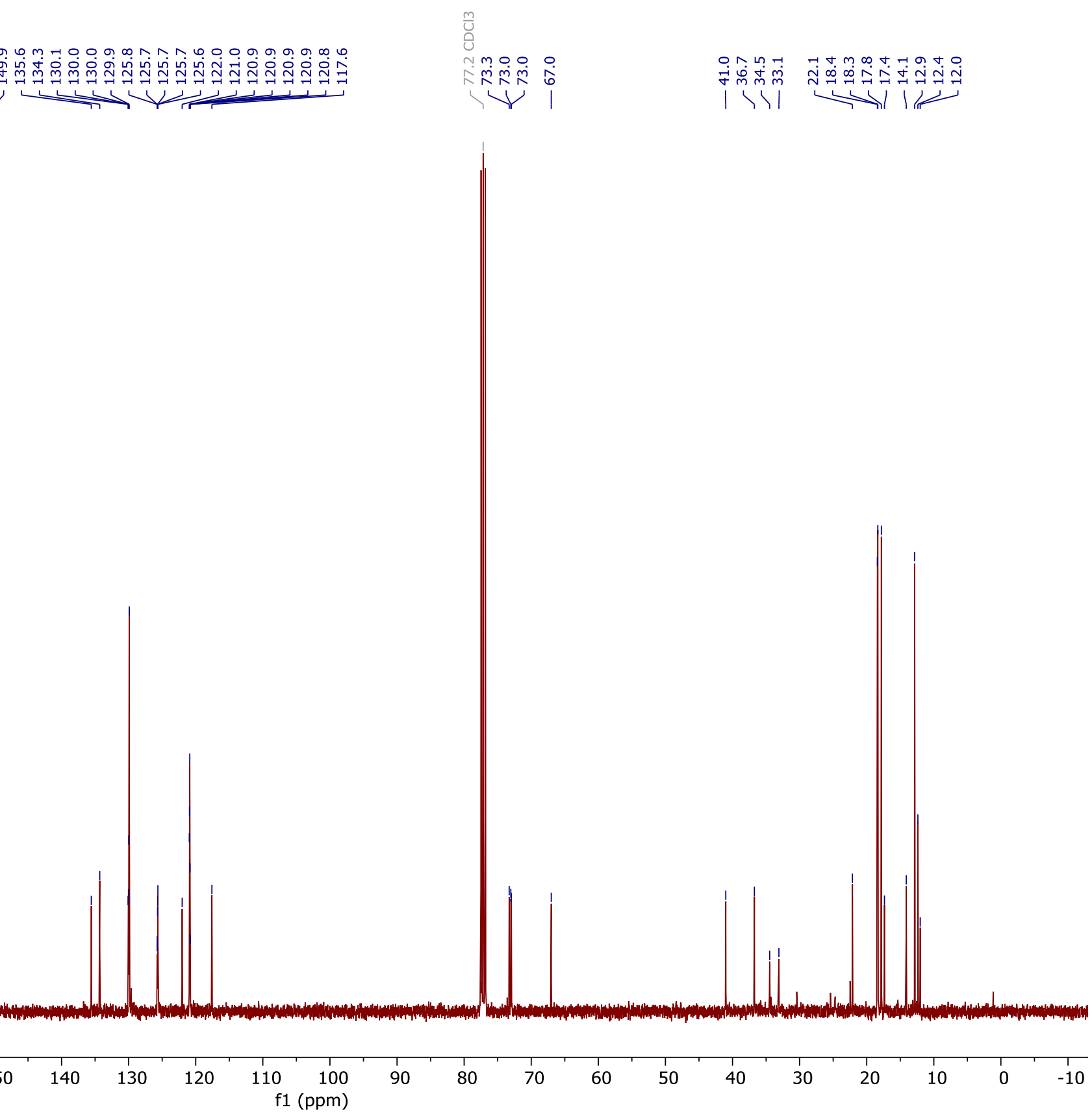

(*Z*)-3-(Tributylstannyl)prop-2-en-1-ole (39b)

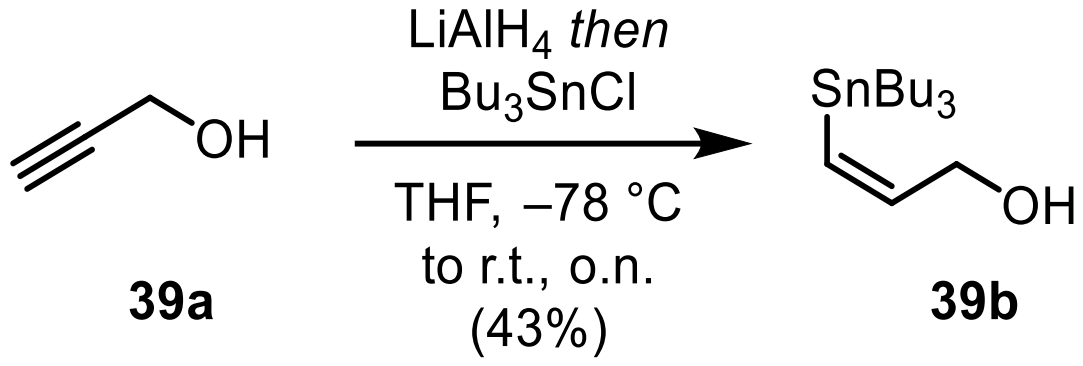

A round-bottom flask was charged with propargyl alcohol (**39a**) (1.51 mL, 25.5 mmol, 3.5 eq.) and 30 mL dry THF. After cooling to 0 °C, $LiAlH_4$ (500 mg, 13.2 mmol, 1.8 eq.) was added portionwise over a period of 30 min and the resulting mixture was stirred overnight at r.t. It was then cooled to –78 °C and subsequently Bu_3SnCl (2.0 mL, 7.4 mmol, 1.0 eq.) was added dropwise. The mixture was warmed to r.t. and stirred overnight. The reaction mixture was quenched with MeOH (10 mL) and diluted with 1M NaOH. After separation of the layers, the aqueous phase was extracted with Et_2O and the combined organic layer was washed with sat. NaCl solution. After drying over $MgSO_4$ and removal of the solvent in vacuo, the crude material was purified by column chromatography (pentane/Et_2O, 12:1) to obtain stannane **39b** (1.1 g, 3.2 mmol, 43%) as a colorless oil.

$\mathbf{R_f}$ = 0.53 (pentane/Et_2O, 3:1), UV (-), $KMnO_4$ (+).

^{1}H NMR (400 MHz, $CDCl_3$) δ [ppm] = 6.69 (dt, *J* = 12.9, 5.8 Hz, 1H), 6.08 (dt, *J* = 12.8, 1.1 Hz, 1H), 4.11 (dt, *J* = 5.8, 1.1 Hz, 2H), 1.58 – 1.43 (m, 6H), 1.37 – 1.22 (m, 6H), 0.95 - 0.84 (m, 15H).

^{13}C NMR (101 MHz, $CDCl_3$) δ [ppm] = 27.2, 29.2, 29.2, 29.2, 66.1, 131.8, 146.2.

IR (ATR) ṽ (cm^{-1}) = 3331 (br), 2956 (s), 2920 (s), 2871 (m), 2852 (m), 1463 (m), 1418 (w), 1377 (m), 1292 (w), 1181 (w), 1073 (w), 1032 (s), 956 (s), 875 (m), 694 (s), 667 (s), 600 (s), 511 (m).

HRMS (ESI) (*m/z*) calcd. for $[C_{15}H_{32}OSnH]^+$ = $[M+H]^+$: 341.1580, found: 341.1587.

(*Z*)-3-(tributylstannyl)acrylaldehyde (39)

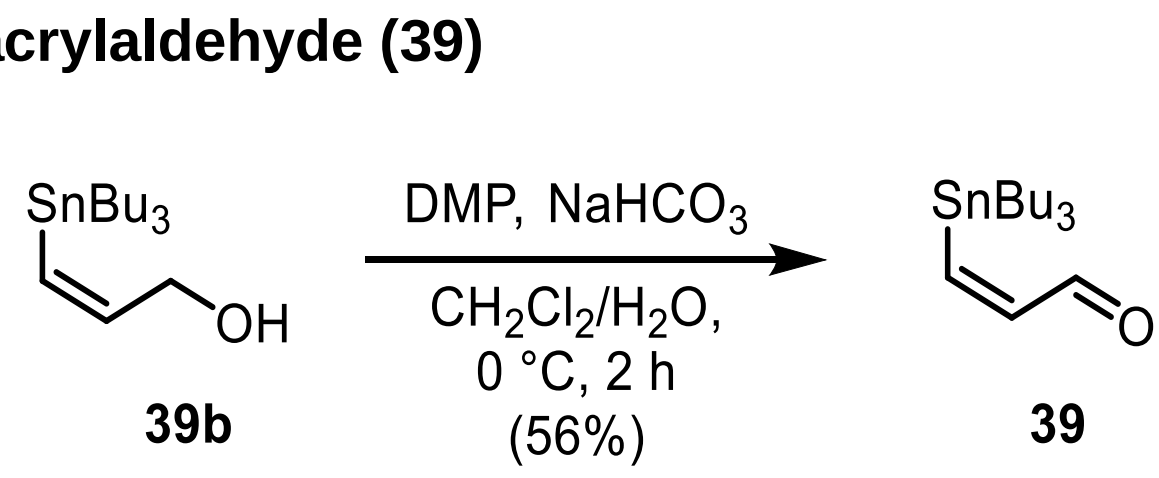

Stannane **39b** (540 mg, 1.56 mmol, 1.0 eq.) was dissolved in 15 mL CH_2Cl_2 (saturated with H_2O) and cooled to 0 °C. Then, a mixture of Dess-Martin periodinane (1.0 g, 2.36 mmol, 1.5 eq.) and $NaHCO_3$ (430 mg, 5.12 mmol, 3.3 eq.) was added portionwise and the resulting mixture was stirred for 2 h at 0 °C. After completion of the reaction, it was diluted with water, extracted with CH_2Cl_2 and the combined organic layer was washed with sat. NaCl solution. The organic layer was dried over $MgSO_4$, concentrated under reduced pressure and the residue

was purified by column chromatography (pentane/Et_2O, 30:1) to provide aldehyde **39** (300 mg, 0.87 mmol, 56%) as a colorless liquid.

R_f = 0.75 (pentane/Et_2O, 25:1), UV (+), $KMnO_4$ (+).

^{1}H NMR (400 MHz, $CDCl_3$) δ [ppm] = 9.50 (dt, *J* = 6.9, 1.8 Hz, 1H), 7.69 (d, *J* = 12.9 Hz, 1H), 6.98 (dd, *J* = 12.9, 6.9 Hz, 1H), 1.59 – 1.42 (m, 6H), 1.36 – 1.23 (m, 6H), 1.06 – 0.97 (m, 6H), 0.92 – 0.82 (m, 9H).

^{13}C NMR (101 MHz, $CDCl_3$) δ [ppm] = 11.3, 13.6, 27.2, 29.0, 145.6, 162.9, 194.7.

IR (ATR) ṽ (cm^{-1}) = 2956 (s), 2921 (s), 2871 (m), 2852 (m), 1687 (s), 1463 (m), 1377 (w), 1173 (m), 909 (m), 875 (w), 792 (w), 733 (s), 698 (s), 667 (m), 598 (m), 529 (m), 500 (m).

HRMS (ESI) (*m/z*) calcd. for $[C_{15}H_{30}OSnH]^+$ = $[M+H]^+$: 339.1423, found: 339.1431.

DHP fragment 7

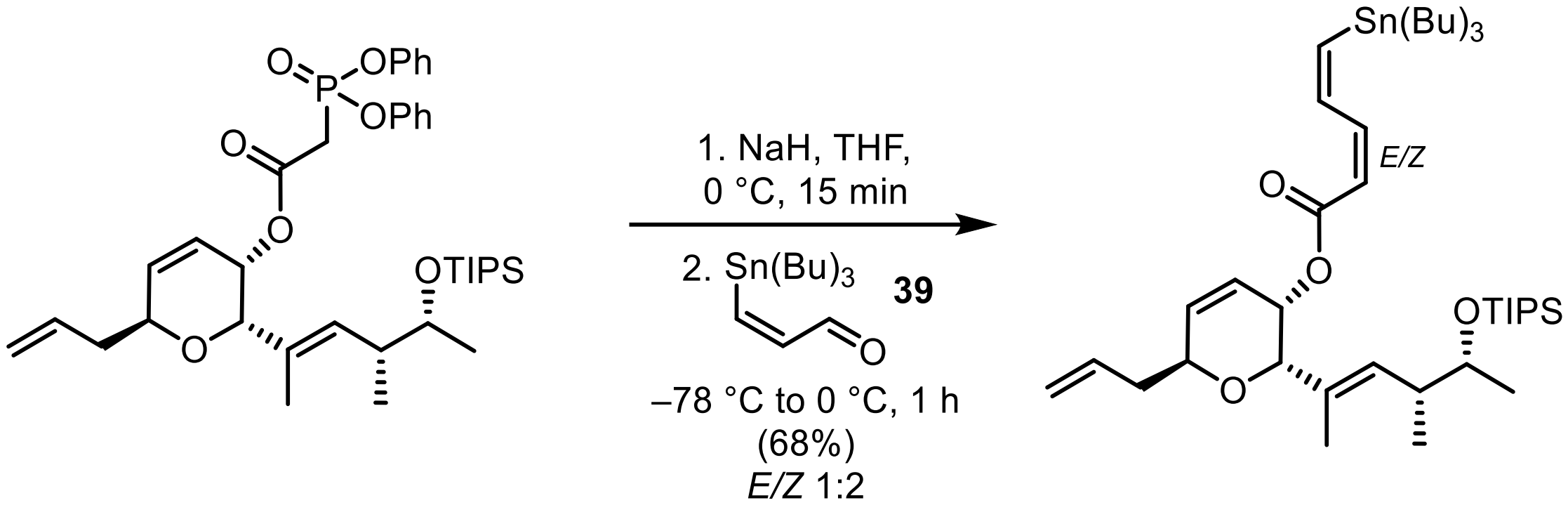

38 **7**

Ester **38** (31.0 mg, 45.4 µmol, 1.0 eq.) was dissolved in 1 mL dry THF and cooled to 0 °C. Subsequently, NaH (60% in mineral oil, 1.8 mg, 45.4 µmol, 1.0 eq.) was added and the mixture was stirred for 15 min. The solution was cooled to –78 °C and stannane **39** (15 mg, 43.5 µmol, 1.0 eq.), dissolved in 0.3 mL dry THF, was added dropwise. The solution was stirred for 30 min at –78 °C and additional 30 min at 0 °C. The reaction was quenched with sat. NH_4Cl solution, the aqueous phase was extracted with Et_2O, the combined organic layer was washed with sat. NaCl solution and dried over $MgSO_4$. The solvent was removed in vacuo and the crude material was purified by column chromatography (pentane/Et_2O, 50:1) to afford dihydropyran **7** (24.0 mg, 30.9 µmol, 68%, *E/Z* 1:2) as a colorless oil.

$\mathbf{R_f}$ = 0.50 (pentane/Et_2O, 20:1), vanillin: blue, UV (+).

^{1}H NMR (400 MHz, $CDCl_3$) δ [ppm] = 8.17 (t, *J* = 1.1 Hz, 1H), 6.83 – 6.60 (m, 1H), 6.50 – 6.34 (m, 1H), 6.05 (d, *J* = 3.0 Hz, 2H), 5.93 – 5.78 (m, 1H), 5.69 (dt, *J* = 11.4, 1.2 Hz, 1H), 5.41 (dt, *J* = 10.2, 1.7 Hz, 1H), 5.21 (dq, *J* = 4.6, 2.5 Hz, 1H), 5.16 – 5.05 (m, 2H), 4.41 (t, *J* = 7.3 Hz, 1H), 4.24 (s, 1H), 3.77 – 3.67 (m, 1H), 2.53 – 2.24 (m, 2H), 1.65 (s, 3H), 1.60 – 1.38 (m, 5H), 1.36 – 1.23 (m, 7H), 1.06 (s, 21H), 1.00 – 0.93 (m, 6H), 0.91 – 0.84 (m, 9H).

^{13}C NMR (101 MHz, $CDCl_3$) δ [ppm] = 165.7, 148.3, 147.1, 142.6, 134.9, 134.5, 130.3, 129.9, 123.1, 118.0, 117.5, 73.4, 73.3, 73.2, 64.9, 41.2, 36.9, 29.2, 27.4, 22.4, 18.4, 18.4, 17.5, 14.2, 13.8, 12.9, 10.7.

HRMS (ESI) (*m/z*) The exact mass could not be detected using various ionization methods (ESI/EI).

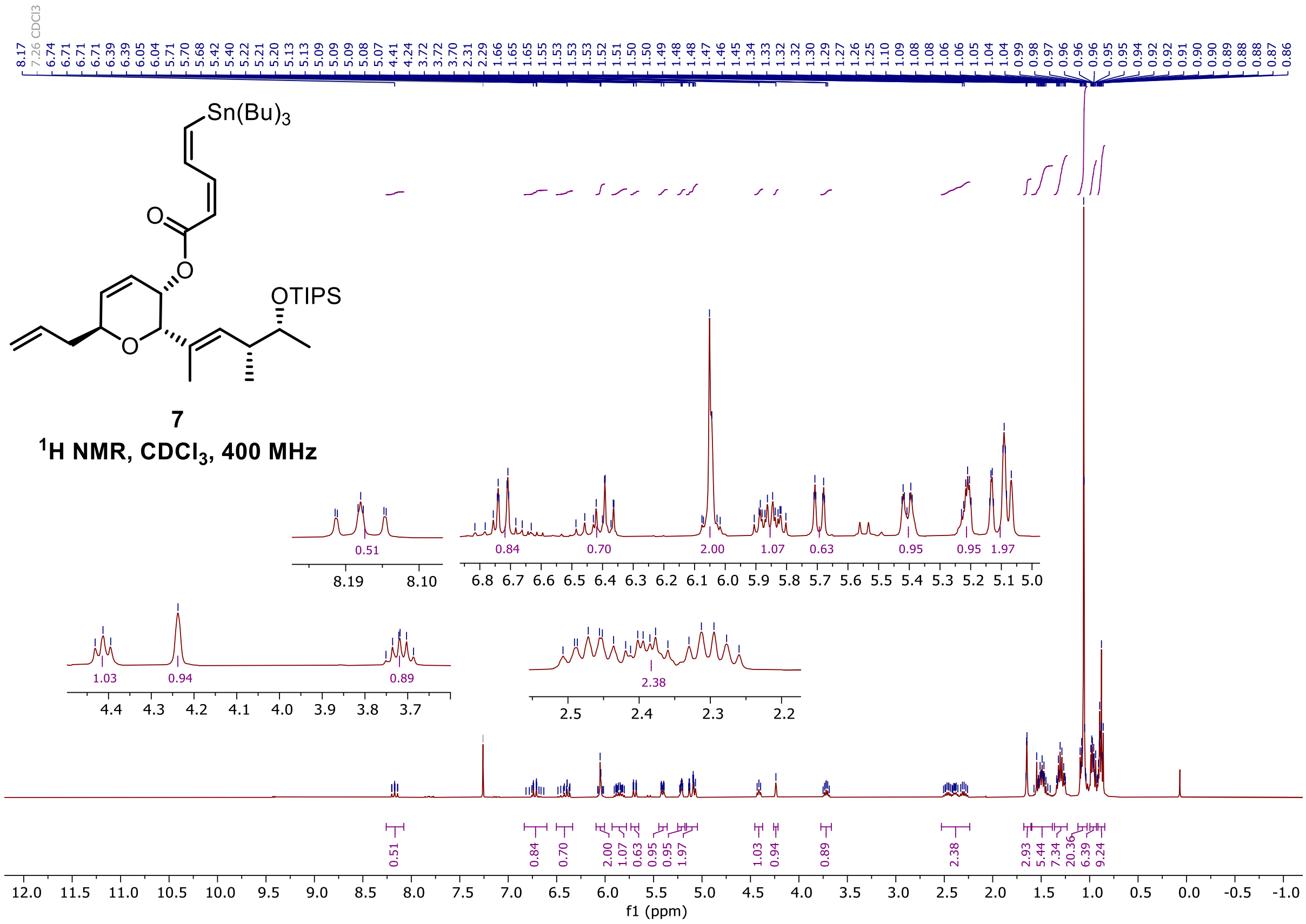

7

^1H NMR, $CDCl_3$, 400 MHz

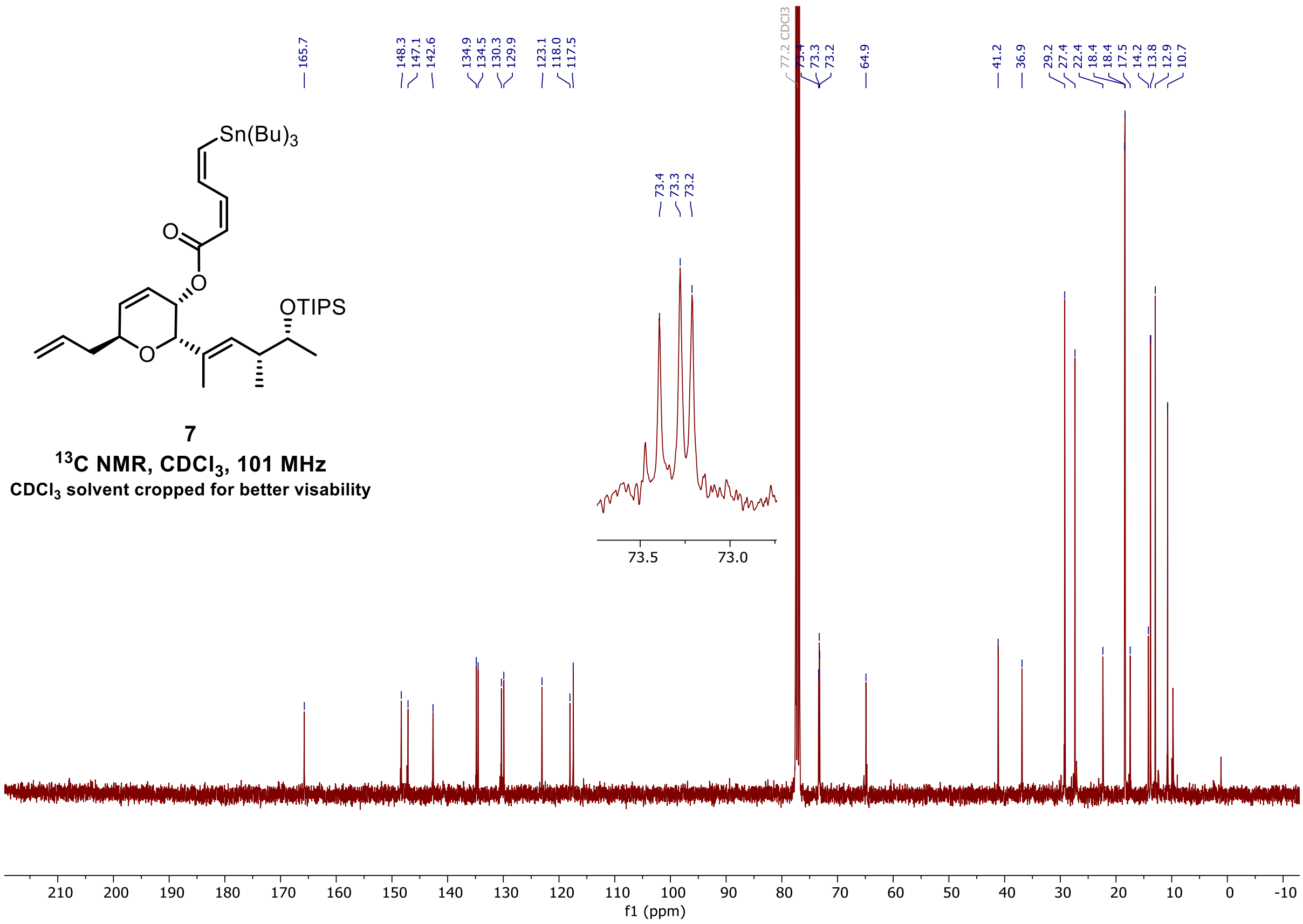

7

^{13}C NMR, $CDCl_3$, 101 MHz

$CDCl_3$ solvent cropped for better visability

(*S*)-4-Benzylthiazolidine-2-thione (11b)

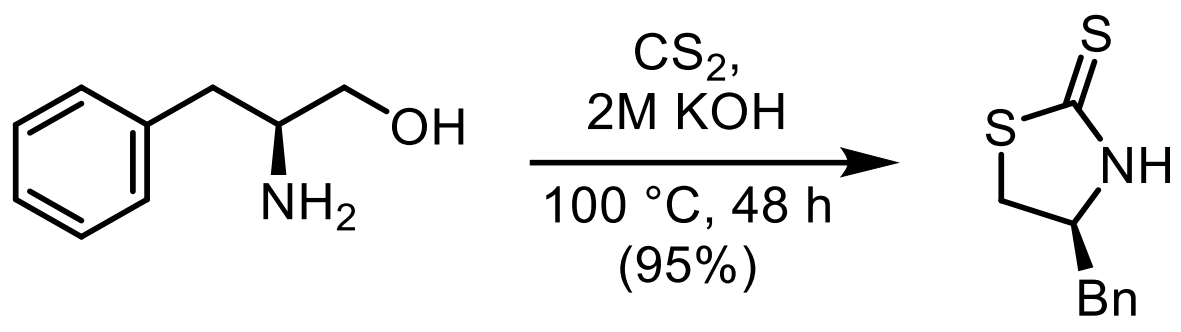

11a **11b**

L-Phenylalaninol (**11a**) (43.3 g, 286.4 mmol, 1.0 eq.) was suspended in 860 mL 2M KOH and stirred at r.t. To this mixture was added CS_2 (86.0 mL, 1.43 mol, 5.0 eq.) and the mixture was refluxed at 100 °C for 48 h using a coil condenser (bp (CS_2) = 46 °C). After cooling to r.t., the mixture was extracted with CH_2Cl_2 (3 x 500 mL), the combined organic layer was dried over Na_2SO_4 and the solvent was removed in vacuo. The crude material was re-crystallized from EtOH (30 mL) to afford auxiliary **11b** (56.9 g, 271.6 mmol, 95%) as a yellow, crystalline solid.

^{1}H NMR (400 MHz, $CDCl_3$) δ [ppm] = 7.53 (s, 1H), 7.39 – 7.27 (m, 3H), 7.24 – 7.16 (m, 2H), 4.46 (tt, *J* = 7.7, 6.6 Hz, 1H), 3.59 (dd, *J* = 11.2, 7.7 Hz, 1H), 3.33 (dd, *J* = 11.2, 6.8 Hz, 1H), 3.08 – 2.93 (m, 2H).

(*S*)-1-(4-Benzyl-2-thioxothiazolidin-3-yl) propan-1-one (11)

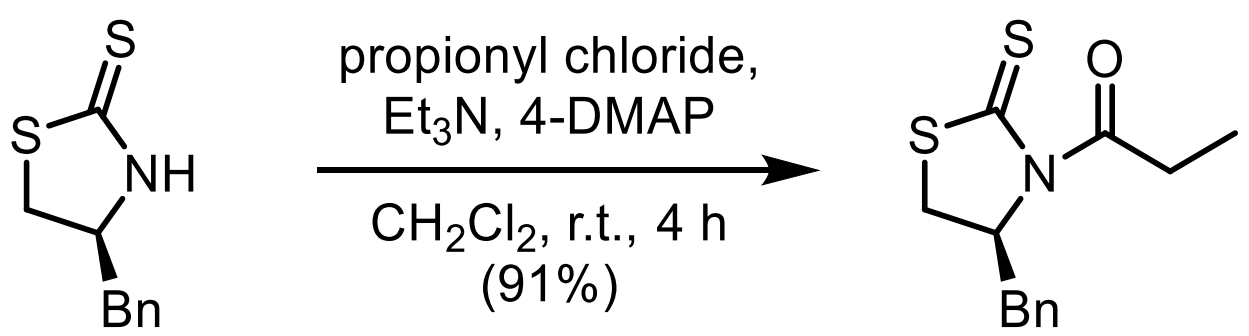

11b **11**

To a solution of auxiliary **11b** (25.66 g, 122.6 mmol, 1.0 eq.) in 500 mL dry CH_2Cl_2 were added Et_3N (20.5 mL, 147.1 mmol, 1.2 eq.) and 4-DMAP (3.0 g, 24.5 mmol, 20 mol%). The mixture was cooled to 0 °C and a solution of propionyl chloride (13.9 mL, 159.4, 1.3 eq.) in 125 mL dry CH_2Cl_2 was added over 20 minutes. The resulting mixture was stirred at r.t. for 4 h, before adding sat. NH_4Cl solution (150 mL) and extracting the aqueous layer with CH_2Cl_2 (3 x 300 mL). The combined organic layer was washed with sat. $NaHCO_3$ solution (150 mL) and sat. NaCl solution (150 mL), dried over Na_2SO_4 and concentrated in vacuo to afford acylated compound **11** (29.57 g, 111.4 mmol, 91%) as a yellow solid, which was used without further purification.

R_f = 0.72 (pentane/Et_2O, 10:1), $KMnO_4$, UV (+)

^{1}H NMR (400 MHz, $CDCl_3$) δ [ppm] = 7.40 – 7.18 (m, 5H), 5.39 (ddd, *J* = 10.8, 7.2, 3.8 Hz, 1H), 3.48 – 3.32 (m, 2H), 3.26 – 2.97 (m, 3H), 2.88 (dd, *J* = 11.6, 0.7 Hz, 1H), 1.19 (t, *J* = 7.2 Hz, 3H).

***anti*-Aldol product 40a**

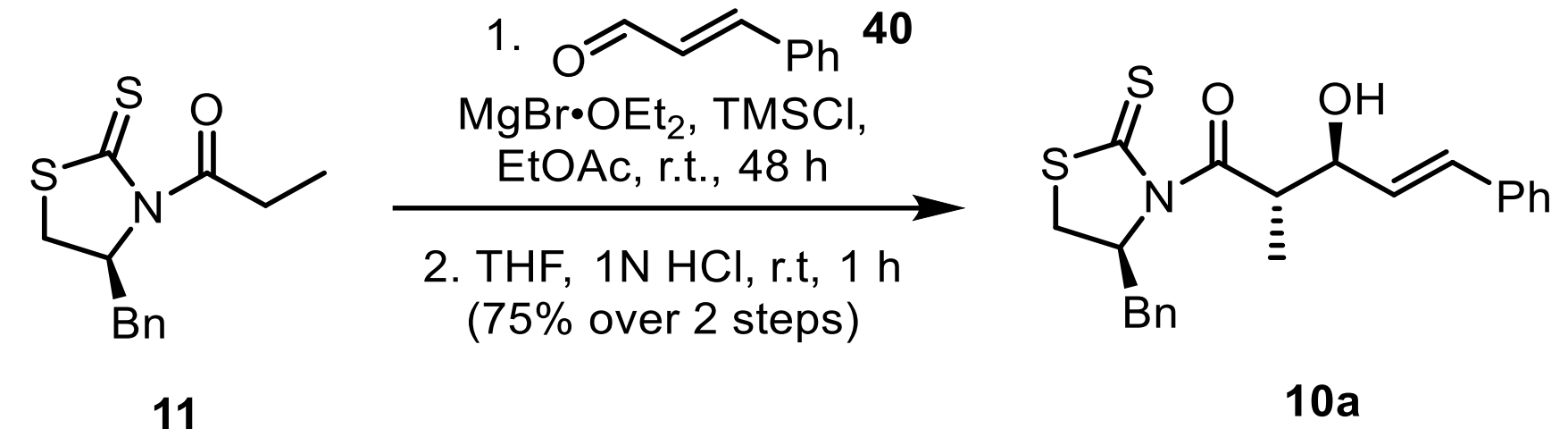

Compound **11** (20.3 g, 76.5 mmol, 1.0 eq.) was dissolved in 155 mL dry EtOAc and treated with *trans*-cinnamaldehyde (**40**) (10.7 mL, 85.0 mmol, 1.1 eq.), magnesium bromide ethyl etherate (2.0 g, 7.7 mmol, 10 mol%), Et_3N (21.4 mL, 153.5 mmol, 2.0 eq.) and TMSCl (14.6 mL,115 mmol, 1.5 eq.). After 48 h stirring at r.t., the reaction mixture was filtered through a silica pad and was washed with 150 mL Et_2O. The filtrate was concentrated under reduced pressure and re-dissolved in 280 mL THF. Next, 1N HCl (80 mL) was added at r.t. and stirring was continued for 1 h, before the mixture was diluted with Et_2O and water. The layers were separated and the aqueous phase was extracted with Et_2O (3 x 100 mL). The combined organic phase was washed with sat. $NaHCO_3$ solution and sat. NaCl solution. After drying over Na_2SO_4 and removal of the solvent in vacuo, the crude material was purified by column chromatography (Et_2O/CH_2Cl_2/pentane, 1:1:3) to afford aldol product **10a** (22.8 g, 57.4 mmol, 75%) as a yellow, highly viscous oil.

Note: Residual *trans*-cinnamaldehyde must be removed prior to the next step to ensure a clean reaction.

R_f = 0.09 (pentane/EtOAc, 5:1), $KMnO_4$, UV (+)

^{1}H NMR (400 MHz, $CDCl_3$) δ [ppm] = 7.42 – 7.17 (m, 10H), 6.64 (dd, *J* = 16.0, 1.2 Hz, 1H), 6.21 (dd, *J* = 15.9, 6.2 Hz, 1H), 5.25 – 5.13 (m, 1H), 4.64 – 4.42 (m, 2H), 3.30 – 3.16 (m, 2H), 3.04 (dd, *J* = 13.2, 10.6 Hz, 1H), 2.82 (dd, *J* = 11.4, 0.7 Hz, 1H), 2.49 (s, 1H), 1.32 (d, *J* = 6.8 Hz, 3H).

^{13}C NMR (101 MHz, $CDCl_3$) δ [ppm] = 201.7, 177.8, 136.6, 136.3, 132.0, 129.8, 129.6, 129.0, 128.8, 128.1, 127.3, 126.6, 76.2, 69.0, 44.9, 36.8, 32.8, 14.8.

HRMS (ESI) (*m/z*) calcd. for $[C_{22}H_{23}NO_2S_2]^+$ = $[M+H]^+$: 398.1248, found: 398.1239.

Acetate 10

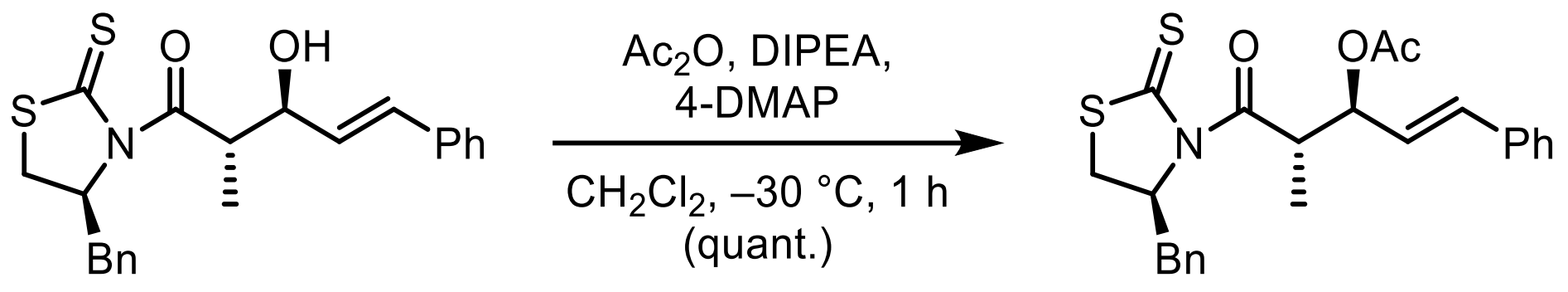

Aldol product **10a** (8.34 g, 21.0 mmol, 1.0 eq.) was dissolved in 85 mL dry CH_2Cl_2 and treated with DIPEA (3.84 mL, 22.0 mmol, 1.05 eq.) and 4-DMAP (51.3 mg, 420 µmol, 20 mol%). After cooling to –30 °C, acetic anhydride (5.6 mL, 59.2 mmol, 2.9 eq.) was added and the reaction was stirred for 1 h at –30 °C. Then, 1N HCl (30 mL) was added and the mixture was stirred for additional 15 min. After separation of the phases, the organic phase was washed with sat. $NaHCO_3$ solution (30 mL) and dried over Na_2SO_4. After removal of the solvent in vacuo, acetate **10** (9.34 g, 21.3 mmol, quant.) was obtained as a yellow-orange residue, which was used without any further purification.

R_f = 0.31 (pentane/EtOAc, 5:1), $KMnO_4$, UV (+)

^{1}H NMR (400 MHz, $CDCl_3$) δ [ppm] = 7.45 – 7.20 (m, 10H), 6.73 (d, *J* = 15.8 Hz, 1H), 6.04 (dd, *J* = 15.9, 8.1 Hz, 1H), 5.65 (ddd, *J* = 9.2, 8.0, 0.9 Hz, 1H), 5.23 (ddd, *J* = 10.6, 6.9, 3.9 Hz, 1H), 4.74 (dq, *J* = 9.1, 6.8 Hz, 1H), 3.36 – 3.21 (m, 2H), 3.06 (dd, *J* = 13.2, 10.6 Hz, 1H), 2.92 (d, *J* = 11.5 Hz, 1H), 2.02 (s, 3H), 1.26 (d, *J* = 6.9 Hz, 3H).

^{13}C NMR (101 MHz, $CDCl_3$) δ [ppm] = 201.3, 175.7, 169.6, 136.5, 136.0, 135.7, 129.6, 129.1, 128.8, 128.5, 127.4, 126.9, 124.7, 69.2, 43.1, 36.8, 32.7, 21.3, 14.5.

IR (ATR) ṽ (cm^{-1}) = 3103 (w), 3083 (w), 3061 (w), 3027 (w), 2962 (w), 2927 (w), 2873 (m), 2852 (w), 1739 (s), 1692 (m), 1602 (w), 1579 (w), 1495 (w), 1452 (w), 1367 (s), 1341 (m), 1319 (w), 1292 (w), 1259 (s), 1226 (s), 1190 (m), 1163 (s), 1136 (s), 1113 (m), 1057 (w), 1016 (s), 966 (s), 915 (w), 884 (w), 860 (w), 850 (w), 832 (w), 806 (w), 744 (s), 693 (s), 666 (w), 651 (w), 613 (w), 601 (w), 536 (w), 532 (w), 511 (w), 487 (w), 465 (w), 432(w).

HRMS (ESI) (*m/z*) calcd. for $[C_{24}H_{25}NO_3S_2]^+$ = $[M+H]^+$: 439.1276, found: 439.1278

Specific Rotation $[\alpha]_{589}^{20}$= = 231.1° (c = 1.5, $CHCl_3$)

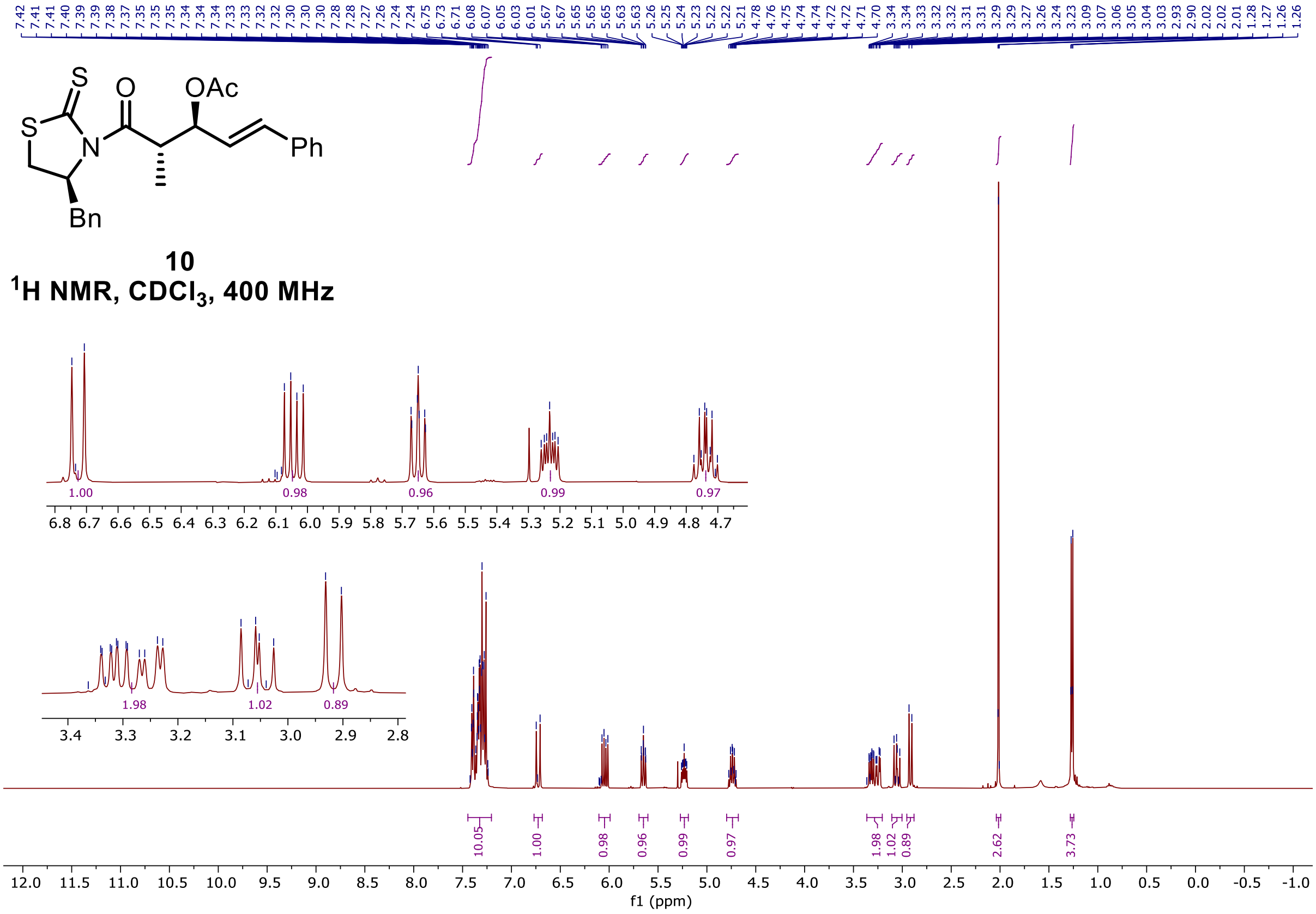

10

^{1}H NMR, $CDCl_3$, 400 MHz

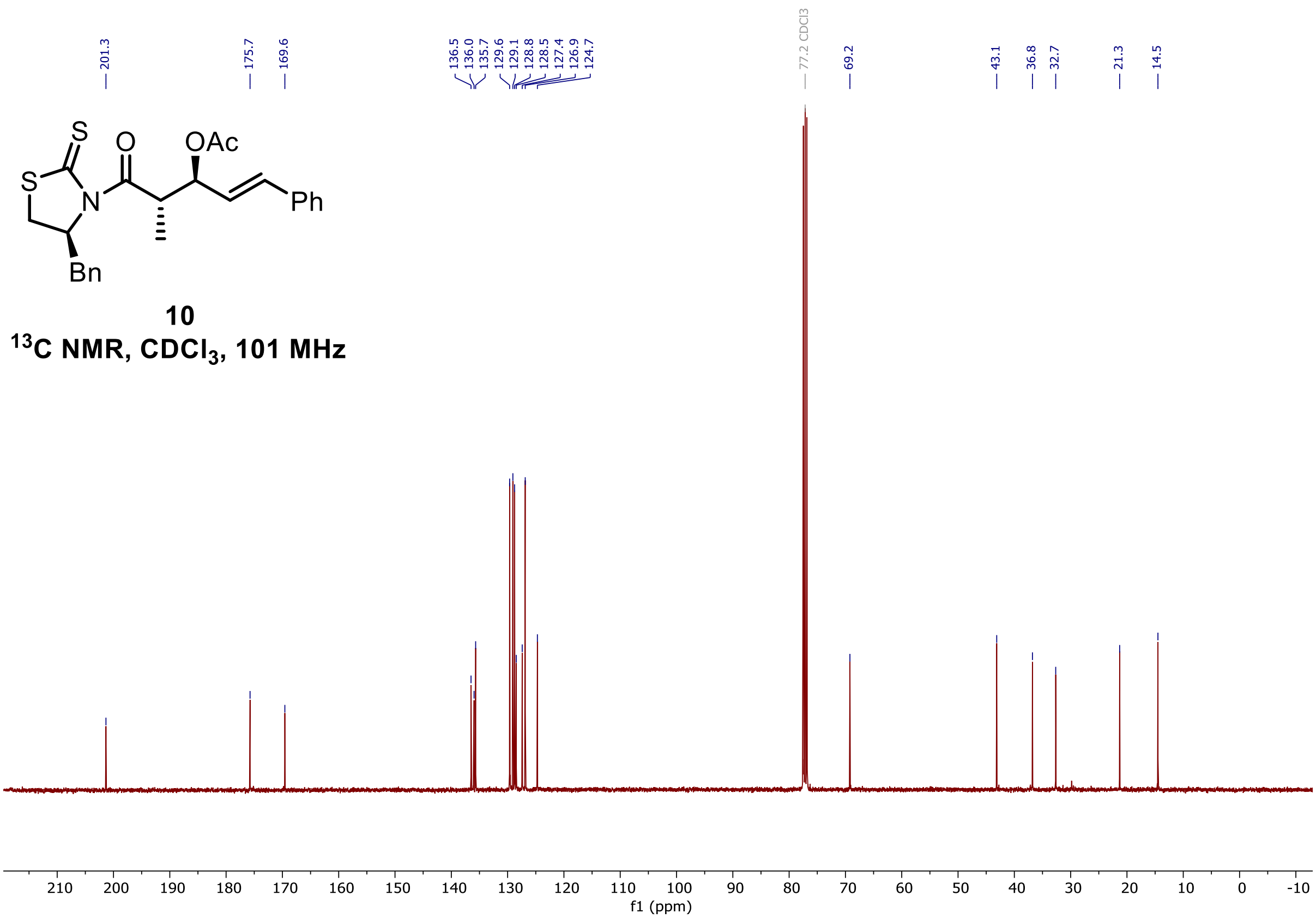

10

^{13}C NMR, $CDCl_3$, 101 MHz

Lactone 41

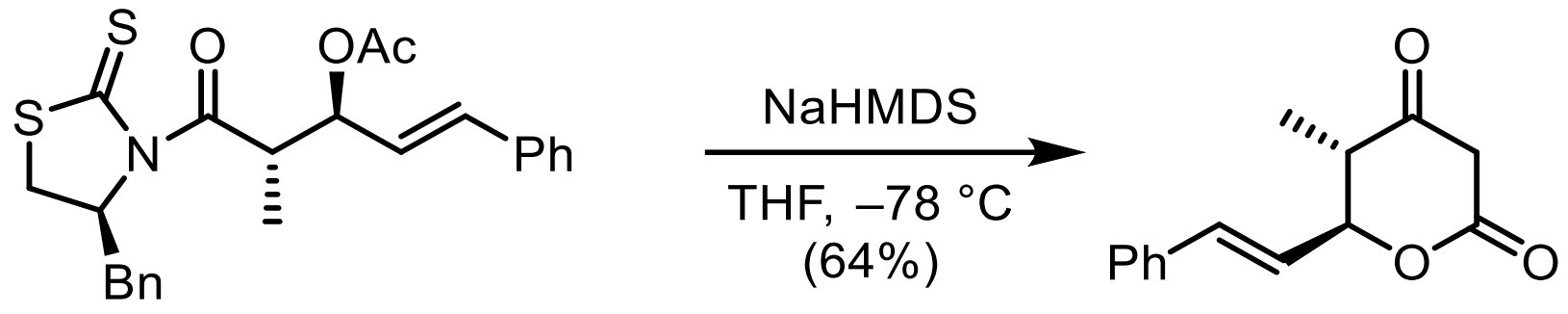

10 **41**

The crude aldol product **10** (46.63 g, 106.1 mmol, 1.0 eq.) was dissolved in 510 mL dry THF and cooled at –78 ° C. Then NaHMDS (2M in THF, 108.2 mL, 216.4 mmol, 2.0 eq.) was added. After the addition, the starting material proved as completely consumed (TLC check). Next, 600 mL 1N HCl/MeOH (v/v, 1:1) were added, the cooling bath was removed and the mixture was further adjusted with 1N HCl to pH 5-6. The mixture was treated with sat. Na_2CO_3 solution until phase separation occurred. The layers were separated and the aqueous layer was adjusted to pH = 1 with conc. HCl, extracted with CH_2Cl_2 (3x) and washed with sat. NaCl solution. The combined organic layer was dried over Na_2SO_4, filtered and concentrated under reduced pressure to yield lactone **41** (15.70 g, 68.2 mmol, 64%) as a yellow solid, which was used without further purification.

For analytical reason, ~15 g crude material was purified by flash chromatography purification (pentane/Et_2O 20%-50%).

R_f = 0.27 (pentane/Et_2O, 2:1), $KMnO_4$, UV (+)

^{1}H NMR (400 MHz, $CDCl_3$) δ [ppm] = 7.46 – 7.27 (m, 5H), 6.77 (d, *J* = 15.8 Hz, 1H), 6.19 (dd, *J* = 15.8, 7.5 Hz, 1H), 4.90 (ddd, *J* = 10.0, 7.6, 1.0 Hz, 1H), 3.69 – 3.46 (m, 2H), 2.65 – 2.50 (m, 1H), 1.21 (d, *J* = 7.1 Hz, 3H).

^{13}C NMR (101 MHz, $CDCl_3$) δ [ppm] = 202.1, 166.9, 136.1, 135.3, 129.0, 128.9, 127.0, 123.4, 81.6, 47.3, 46.1, 11.4.

IR (ATR) ṽ (cm^{-1}) = 3780 (w), 3723 (w), 3698 (w), 3680 (w), 3660 (w), 3640 (w), 2980 (m), 2887 (w), 2378 (w), 2349 (w), 2314 (w), 1726 (w), 1709 (w), 1636 (m), 1560 (m), 1482 (w), 1451 (w), 1381 (w), 1360 (w), 1285 (m), 1262 (s), 1240 (s), 1213 (w), 1162 (w), 1121 (w), 1089 (w), 1074 (w), 1052 (w), 1019 (m), 978 (m), 915 (w), 857 (w), 834 (m), 813 (w), 755 (m), 772 (w), 695 (m), 670 (w), 626 (w), 533 (w), 504 (m), 448 (w), 430 (m).

HRMS (ESI) (*m/z*) calcd. for $[C_{14}H_{15}O_3]^+$ = $[M+H]^+$: 231.1021, found: 231.1025.

Specific Rotation $[\alpha]_{589}^{20}$= 259.0° (c = 1.0, $CHCl_3$)

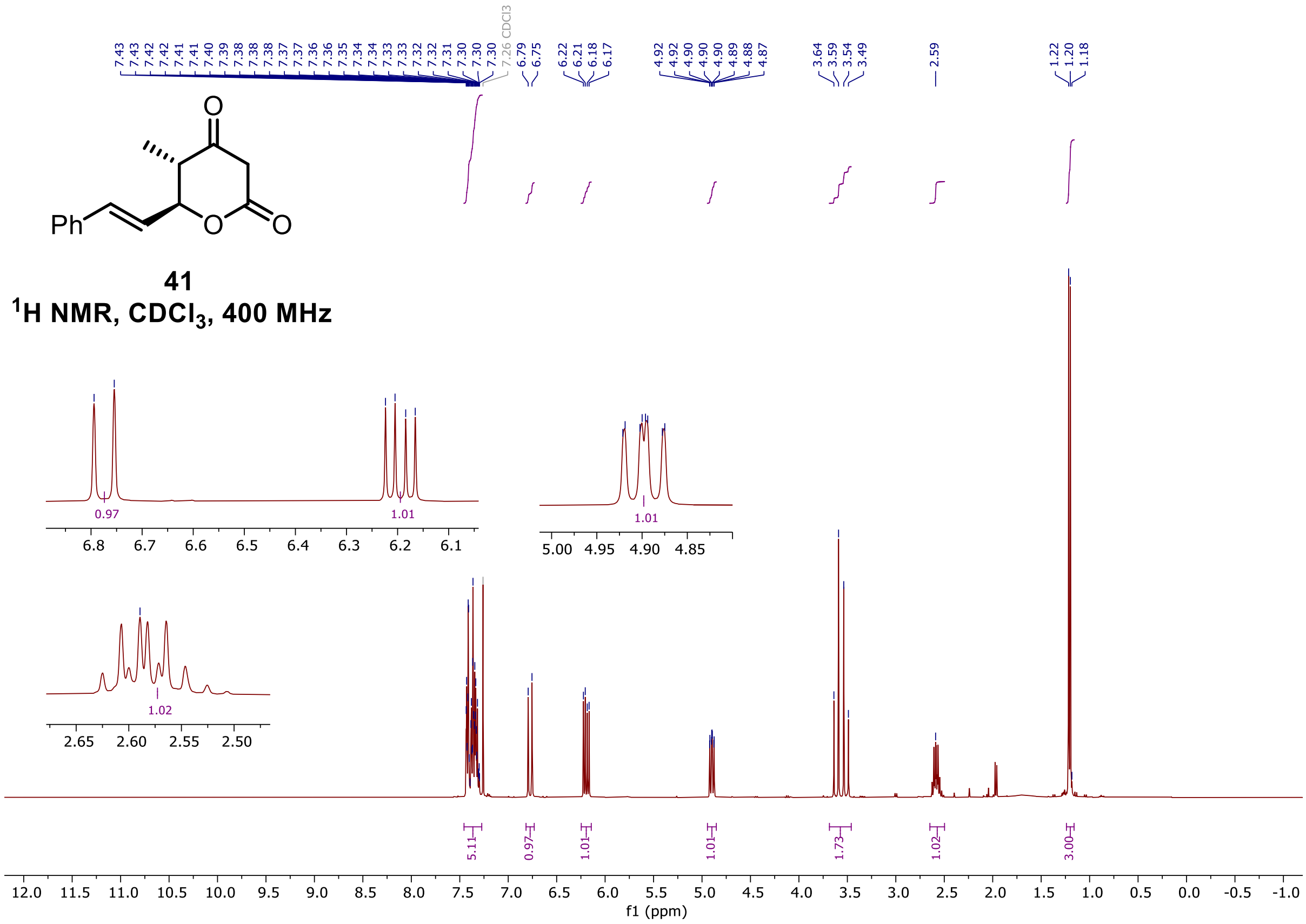

41

^{1}H NMR, CDCl$_3$, 400 MHz

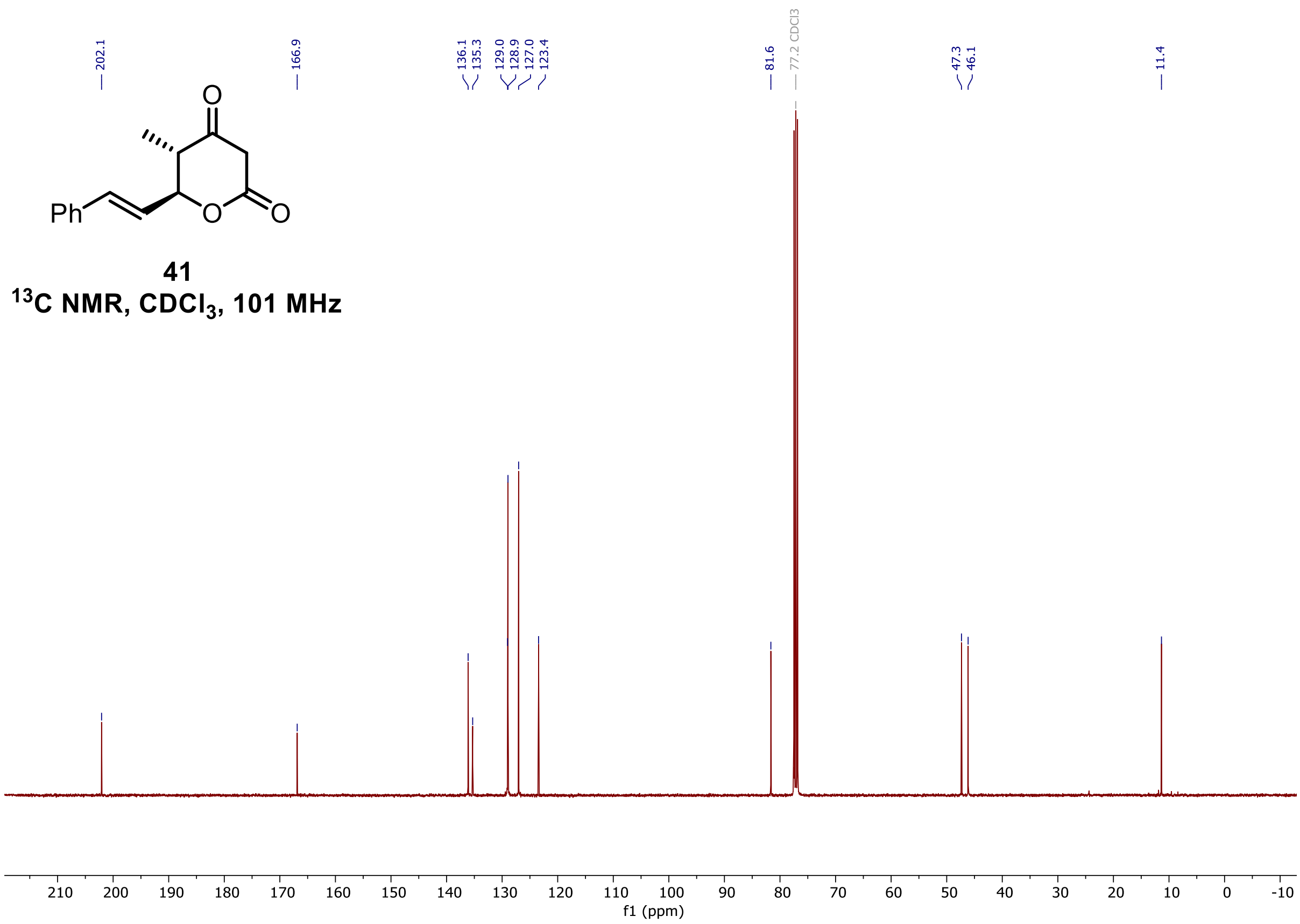

41

^{13}C NMR, CDCl$_3$, 101 MHz

Lactone 42

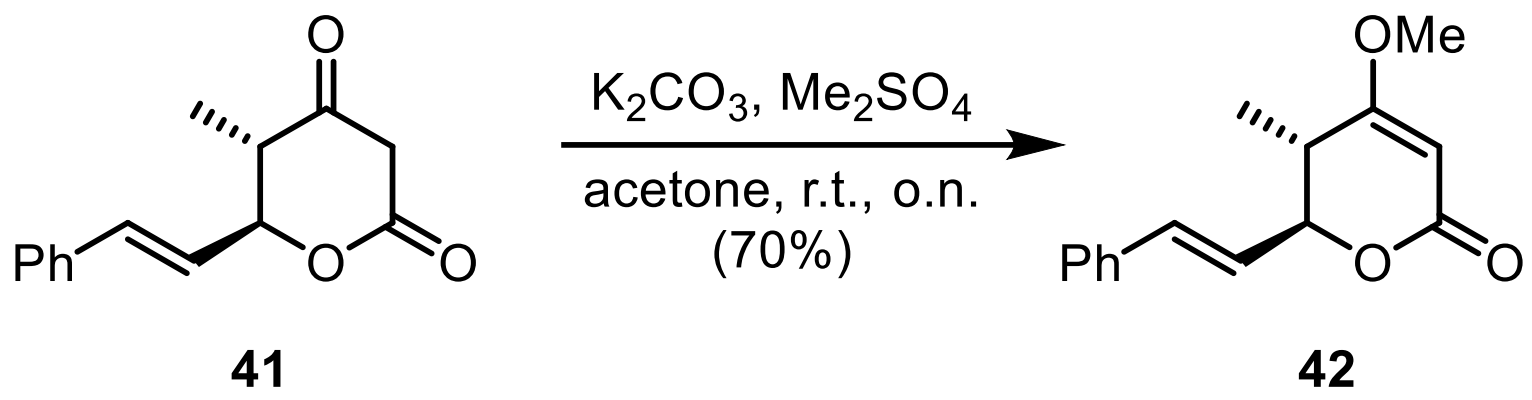

To a solution of lactone **41** (2.75 g, 11.9 mmol, 1.2 eq.) in 120 mL acetone, dry K_2CO_3 (1.82 g, 13.1 mmol, 1.3 eq.) and Me_2SO_4 (0.96 mL, 10.1 mmol, 1.0 eq.) were added at r.t. After stirring at r.t. overnight, the mixture was diluted with Et_2O and water. The layers were separated and the aqueous phase was extracted with Et_2O (2 x 100 mL). The combined organic phase was dried over Na_2SO_4 and the solvent was removed under vacuum. After flash chromatography purification (pentane/EtOAc, 2:1), lactone **42** (1.72 g, 7.0 mmol, 70%) was obtained as a white-yellowish solid.

R_f =0.56 (pentane/EtOAc, 2:1), $KMnO_4$, UV (+)

^{1}H NMR (400 MHz, $CDCl_3$) δ [ppm] = 7.44 – 7.23 (m, 5H), 6.70 (dd, *J* = 16.0, 1.2 Hz, 1H), 6.22 (dd, *J* = 15.9, 7.0 Hz, 1H), 5.15 (d, *J* = 0.8 Hz, 1H), 4.70 (td, *J* = 7.2, 1.2 Hz, 1H), 3.75 (s, 3H), 2.69 – 2.58 (m, 1H), 1.24 (d, *J* = 7.1 Hz, 3H).

^{13}C NMR (101 MHz, $CDCl_3$) δ [ppm] = 175.4, 166.3, 135.9, 134.6, 128.8, 128.5, 126.8, 125.4, 89.9, 82.2, 56.3, 37.2, 14.0.

HRMS (ESI) (*m/z*) calcd. for $[C_{15}H_{17}O_3]^+$ = $[M+H]^+$: 245.1178, found 245.1179.

IR (KBr pressel) ṽ (cm-1) = 3419 (m, br), 3093 (w), 2930 (w), 1703 (s), 1612 (w), 1460 (m), 1378 (m), 1361 (m), 1238 (s), 1122 (m), 1023 (m), 975 (m), 751 (w)

Specific Rotation $[\alpha]^{20}_{589}$= +25.0° (c = 0.025, $CHCl_3$)

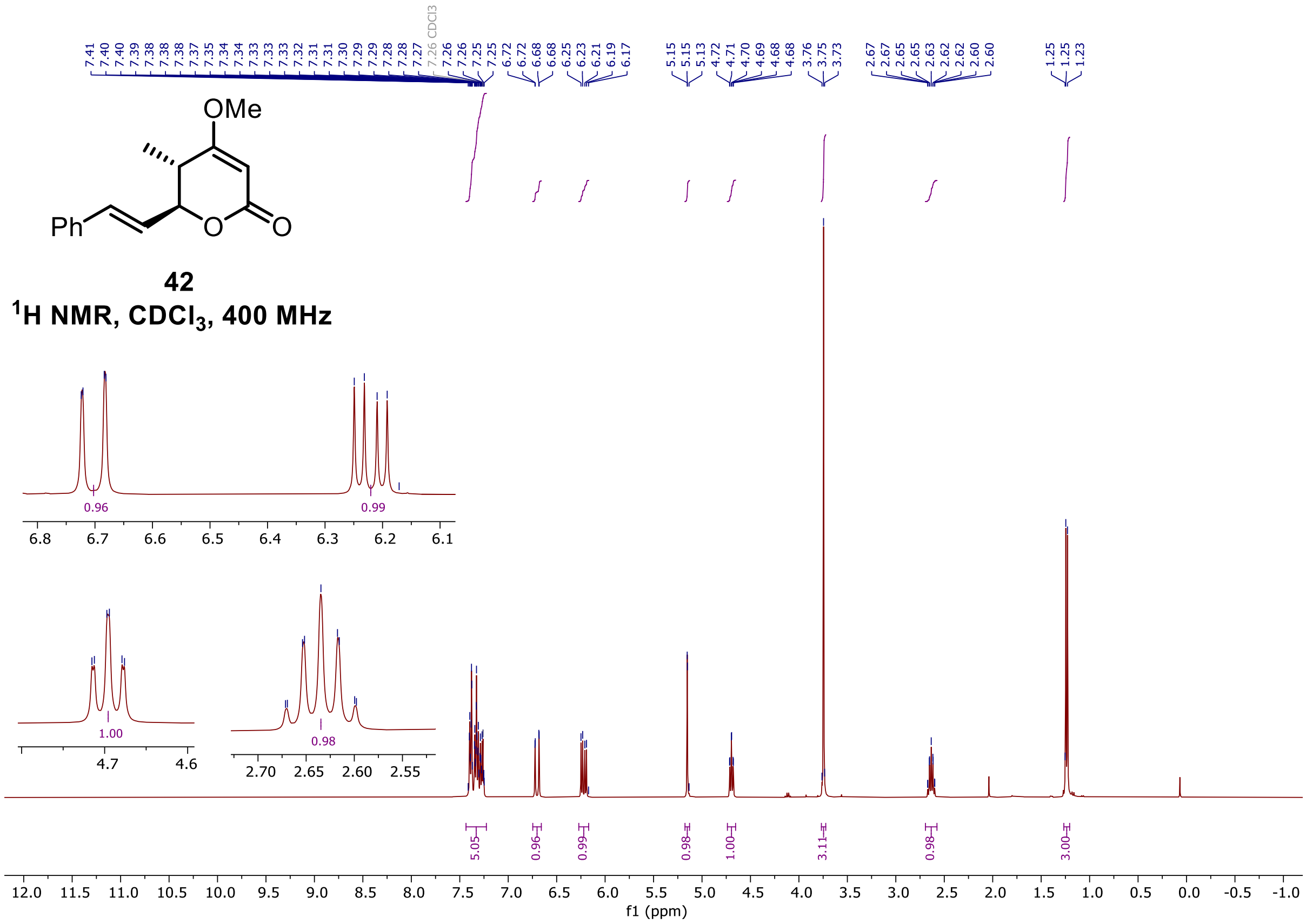

42

^{1}H NMR, $CDCl_3$, 400 MHz

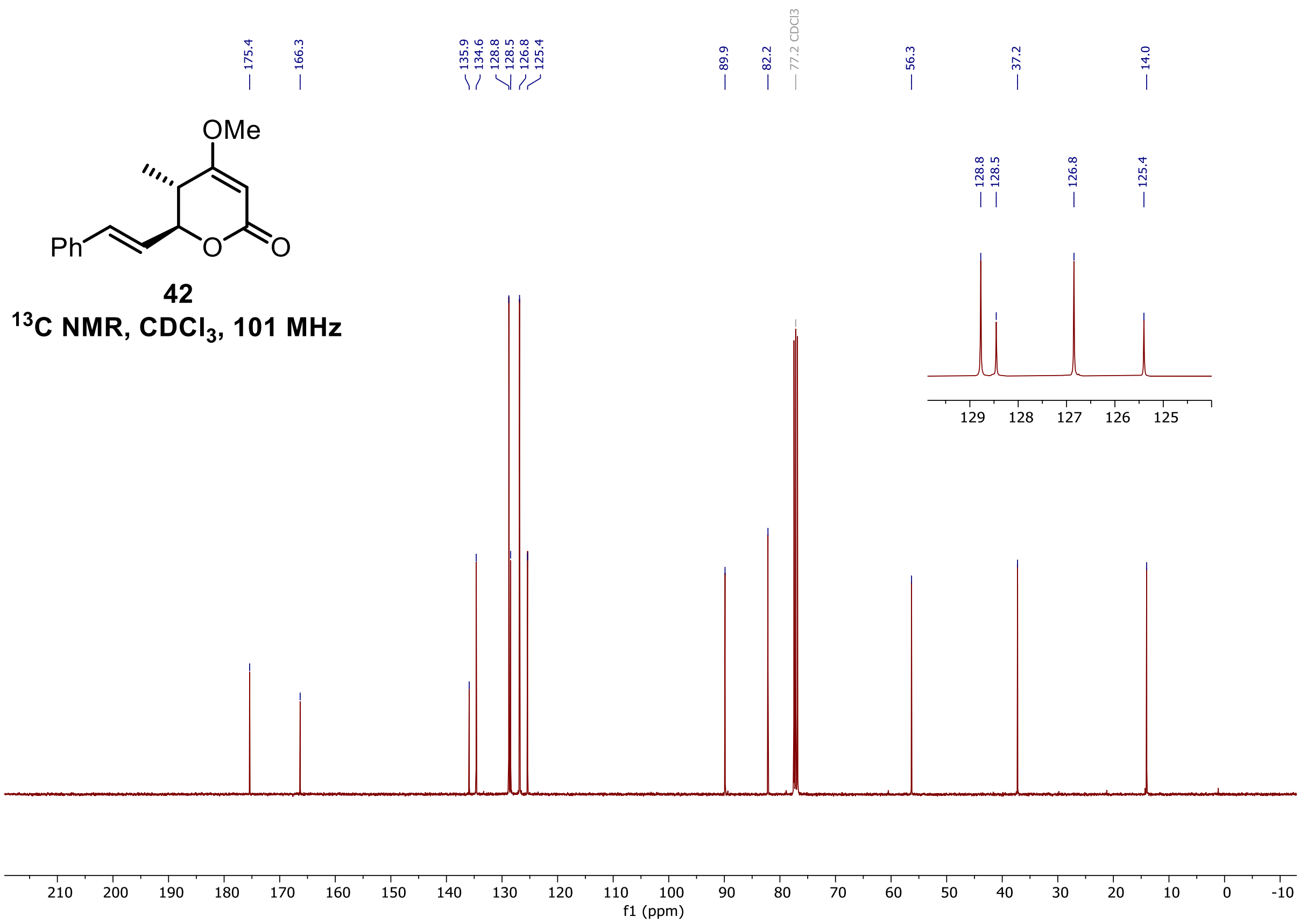

42

^{13}C NMR, $CDCl_3$, 101 MHz

Pyranon 9

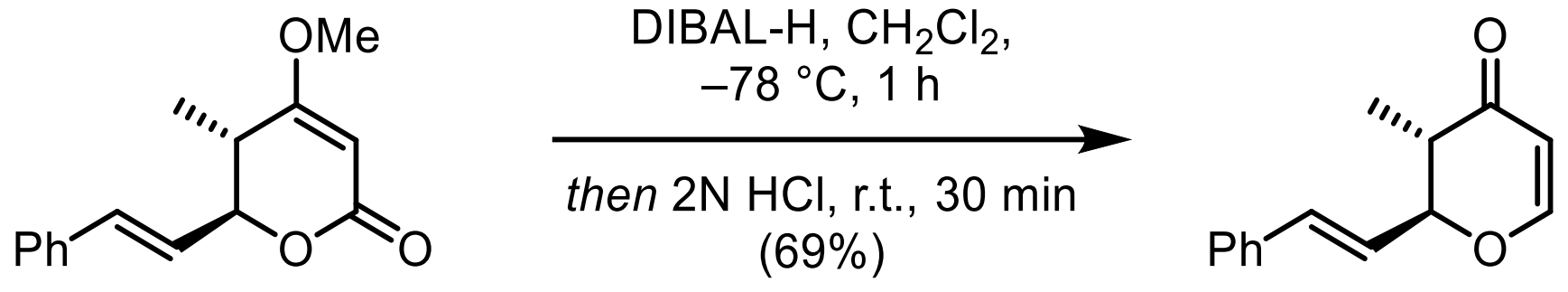

42 **9**

Lactone **42** (11.0 g, 45.0 mmol, 1.0 eq.) was dissolved in 150 mL dry CH_2Cl_2 and cooled to –78 °C. To this solution was added DIBAL-H (1M in *n*-hexane, 50.0 mL, 50.0 mmol, 1.1 eq.) dropwise and the mixture was stirred for 50 min. Since TLC showed remaining starting material, more DIBAL-H (1M in *n*-hexane, 2.0 mL, 2.0 mmol, 0.05 eq.) was added. After additional stirring for 10 min, 2N HCl (50 mL) was added and the cooling bath was removed. The mixture was stirred vigorously for 30 min, the phases were separated and the aqueous phase was extracted CH_2Cl_2. After washing the combined organic phases with water and drying over Na_2SO_4, the solvent was removed under vacuum. After re-crystallization from (Et_2O/pentane 1:2), pyranon **9** (6.5 g) was obtained as a colorless solid. The mother liquor was also concentrated in vacuo and the residue was purified by flash chromatography (Et_2O/pentane, 1:2) to afford additional 0.2 g. The overall yield is 69% (6.7 g, 31.3 mmol, 69%).

$\mathbf{R_f}$ = 0.66 (pentane/EtOAc, 2:1), $KMnO_4$, UV (+)

^{1}H NMR (400 MHz, $CDCl_3$) δ [ppm] = 7.46 – 7.28 (m, 5H), 6.74 (d, *J* = 15.9 Hz, 1H), 6.28 (dd, *J* = 15.9, 7.8 Hz, 1H), 5.45 (d, *J* = 6.0 Hz, 1H), 4.64 (ddt, *J* = 12.3, 7.8, 0.7 Hz, 1H), 2.61 (dq, *J* = 12.2, 7.0 Hz, 1H), 1.14 (d, *J* = 7.1 Hz, 3H).

^{13}C NMR (101 MHz, $CDCl_3$) δ [ppm] = 194.5, 162.3, 135.7, 135.6, 128.8, 128.7, 126.9, 124.9, 106.6, 85.5, 44.3, 10.8.

IR (ATR) ṽ (cm^{-1}) = 3029 (w), 3002 (w), 2973 (w), 2935 (w), 2874 (w), 1652 (s), 1595(s), 1533 (w), 1492 (w), 1453 (w), 1406 (w), 1377 (w), 1296 (w), 1280 (w), 1250 (m), 1202 (w), 1189 (m), 1158 (w), 1121 (w), 1094 (w), 1074 (w), 1050 (m), 1022 (m), 978 (m), 940 (m), 891 (w), 842 (m), 825 (w), 808 (m), 778 (m), 749 (m), 693 (m), 608 (w), 587 (w), 566 (w), 528 (m), 500 (m), 475 (w), 463 (w), 411 (w).

HRMS (ESI) (*m/z*) calcd. for $[C_{14}H_{14}O_2]^+$ = $[M+H]^+$: 215.1072, found: 215.1066.

Specific Rotation $[\alpha]^{20}_{589}$= -160.6° (c = 0.35, $CHCl_3$)

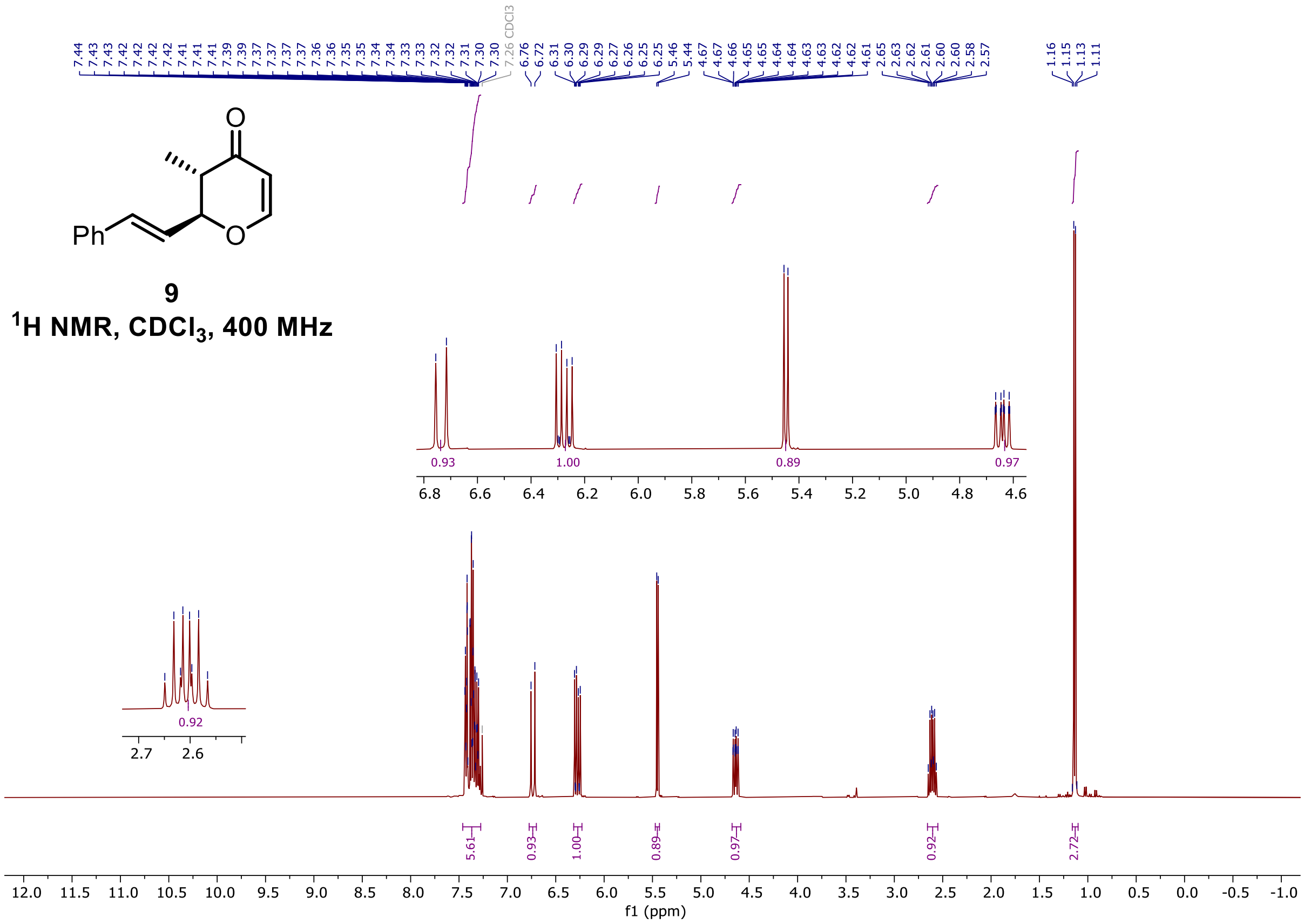

9

^{1}H NMR, $CDCl_3$, 400 MHz

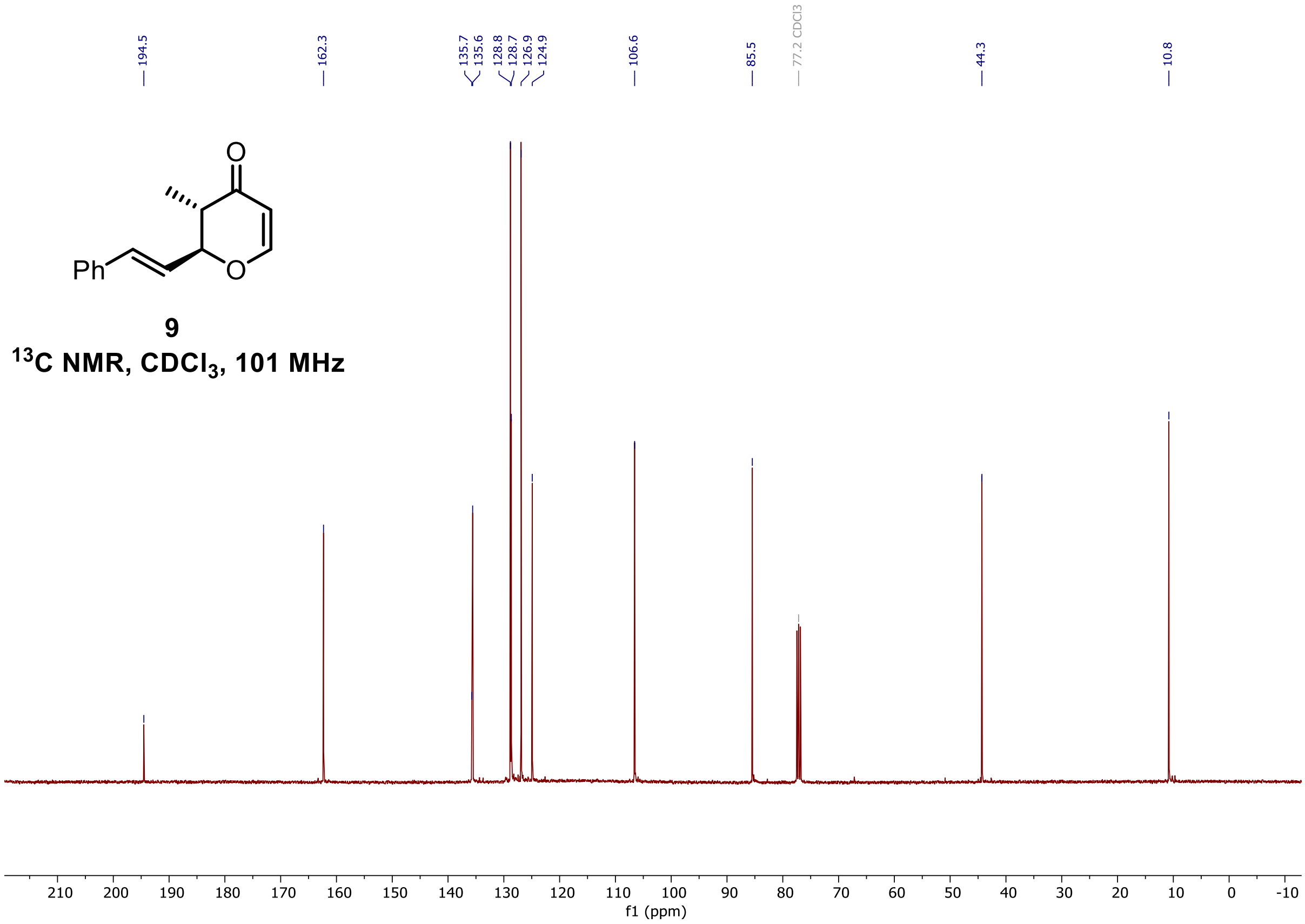

9

^{13}C NMR, $CDCl_3$, 101 MHz

Methyl ester 44a

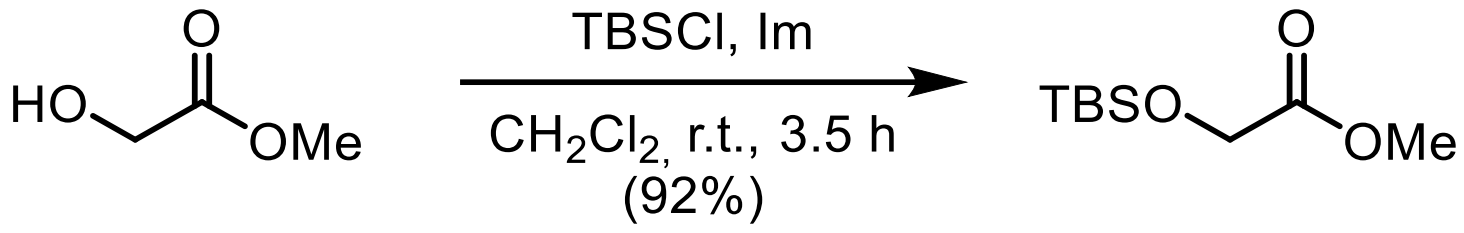

43 **44a**

Methyl glycolate (**43**) (8.2 g, 89.1 mmol, 1.0 eq.) was dissolved in 105 mL dry CH_2Cl_2 at r.t. and imidazole (14.7 g, 213.8 mmol, 2.4 eq.) was added portionwise. Next, TBSCl (16.4 g, 106.9 mmol, 1.2 eq.) was added and the reaction mixture was stirred for 3.5 h at r.t. The mixture was diluted with water (150 mL), the layers were separated and the organic layer was washed with additional water (150 mL) and sat. NaCl solution (150 mL). The organic layer was dried over Na_2SO_4 and the solvent was removed under reduced pressure (max. ~195 mbar, 40 °C water bath due to volatility of the product). The crude material was purified by flash chromatography (pentane/Et_2O, 30:1 → 20:1) to afford methyl ester **44a** (16.8 g, 82.3 mmol, 92%) as a colorless liquid.

$\mathbf{R_f}$ = 0.54 (pentane/Et_2O, 20:1), $KMnO_4$, UV (-)

^{1}H NMR (400 MHz, $CDCl_3$) δ [ppm] = 4.24 (s, 2H), 3.73 (s, 3H), 0.91 (s, 9H), 0.10 (s, 6H).

^{13}C NMR (101 MHz, $CDCl_3$) δ [ppm] = 172.3, 61.8, 51.9, 25.9, 18.6, -5.3.

Ketal 44

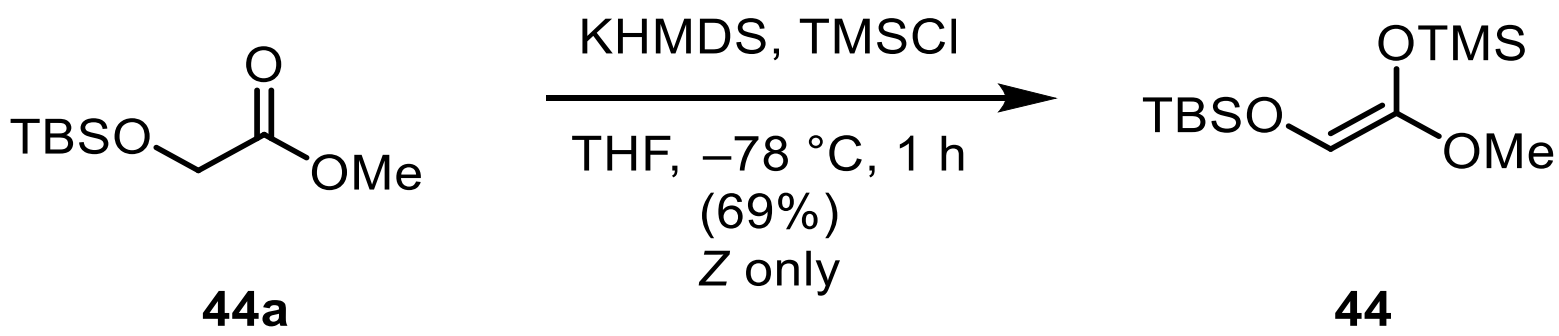

44a **44**

To a solution of KHMDS (6.48 g, 32.5 mmol, 1.1 eq.) in 120 mL dry THF at –78 °C, was added dropwise methyl ester **44a** (6.00 g, 29.4 mmol, 1.0 eq.). After stirring 1 h at –78 °C, TMSCl (4.1 mL, 32.3 mmol, 1.1 eq.) was added, the mixture was stirred for 1 h at –78 °C and additional 20 min at r.t. Next, semi-saturated $NaHCO_3$ solution was added and the mixture was further diluted with pentane. The layers were separated and the organic layer was washed with water and sat. NaCl solution. The organic layer was dried over Na_2SO_4, the solvent was evaporated in vacuo and the residue was purified by distillation under high-vacuum (bp 63 – 65 °C) to afford ketal **44** (5.57 g, 20.1 mmol, 69%, *Z* only) as a colorless liquid.

^{1}H NMR (400 MHz, $CDCl_3$) δ [ppm] = 5.51 (s, 1H), 3.47 (s, 3H), 0.92 (s, 9H), 0.22 (s, 9H), 0.10 (s, 6H).

Pyranon 45

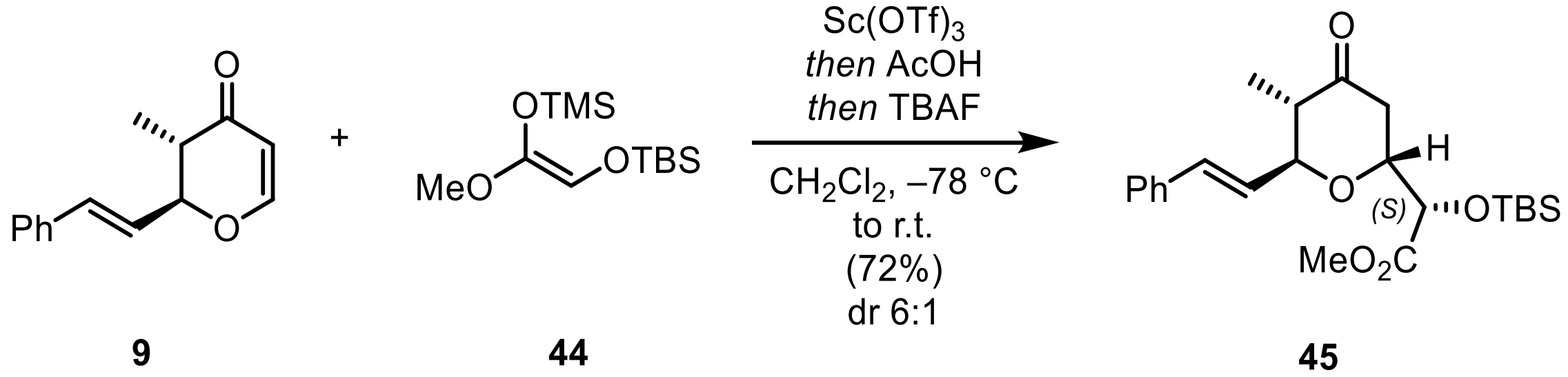

To a –78 °C cooled solution of dihydropyranon **9** (500 mg, 2.33 mmol, 1.0 eq.) and $Sc(OTf)_3$ (30 mg, 61 µmol, 3 mol%) in 10 mL dry CH_2Cl_2, was added a solution of ketal **44** (942 mg, 3.50 mmol, 1.5 eq.) in 20 mL dry CH_2Cl_2 via syringe pump. After full consumption of the starting material, glacial acetic acid (0.33 mL, 5.82 mmol, 2.5 eq.) was added and the reaction was warmed up to 0 °C. Then, TBAF (1M in THF, 3.51 mL, 3.51 mmol, 1.5 eq.) was added and stirring was continued for 10 min, before quenching with sat. $NaHCO_3$ solution and dilution with CH_2Cl_2. After separation of the layers, the aqueous phase was extracted with CH_2Cl_2 and the combined organic layer was dried over $MgSO_4$. The solvent was removed in vacuo and the crude material was purified by flash chromatography (pentane/EtOAc, 5:1) to afford pyranon **45** (701 mg, 1.67 mmol, 72%, dr 6:1) as a colorless oil, which tends to crystallize at r.t.

Note: For this step, the reaction condition, suitable for scales over 15 grams, has been developed. The experimental procedure involves the gradual addition of approximately 6-10 equivalents (monitored by TLC) of crude ketal **44** using a syringe pump at a flow rate up to 6 mL/h. This addition was carried out in a mixture containing well-diluted dihydropyran **9** and 5-10 mol% $Sc(OTf)_3$ at –78 °C.

R_f = 0.30 (pentane/EtOAc, 3:1), vanillin, UV (+)

^{1}H NMR (400 MHz, $CDCl_3$) δ [ppm] = 7.43 – 7.21 (m, 5H), 6.57 (d, *J* = 15.7 Hz, 1H), 6.10 (dd, *J* = 15.9, 7.2 Hz, 1H), 4.74 (ddd, *J* = 8.5, 7.2, 1.1 Hz, 1H), 4.64 (dt, *J* = 7.0, 3.5 Hz, 1H), 4.35 (d, *J* = 3.3 Hz, 1H), 3.76 (s, 3H), 2.63 (ddd, *J* = 15.2, 6.9, 1.1 Hz, 1H), 2.54 (dd, *J* = 15.2, 3.6 Hz, 1H), 2.40 (dtd, *J* = 9.5, 7.3, 6.3 Hz, 1H), 1.09 (d, *J* = 6.8 Hz, 3H), 0.93 (s, 9H), 0.08 (d, *J* = 5.4 Hz, 6H).

^{13}C NMR (101 MHz, $CDCl_3$) δ [ppm] = 206.8, 171.5, 136.5, 133.7, 128.7, 128.1, 128.1, 126.8, 80.0, 77.1, 74.9, 52.3, 48.8, 41.2, 25.9, 18.4, 11.0, -5.2, -5.3.

IR (KBr pressel) ṽ (cm^{-1}) = 3439 (s, br), 3028 (w), 2953 (s), 2884 (s), 2040 (w, br), 1761 (s), 1719 (s), 1631 (m), 1496 (w), 1463 (m), 1365 (m), 1282 (s), 1258 (s), 1174 (s), 1123 (s), 1097 (s), 968 (s), 930 (m), 839 (s), 783 (s), 693 (m), 673 (w), 601 (w), 530 (m), 460 (w)

HRMS (ESI) (*m/z*) calcd. for $[C_{23}H_{34}O_5Si]^+$ = $[M+H]^+$: 419.2254, found: 419.2253.

Specific Rotation $[\alpha]_{589}^{20}$ = –42.0° (c = 0.2, $CHCl_3$)

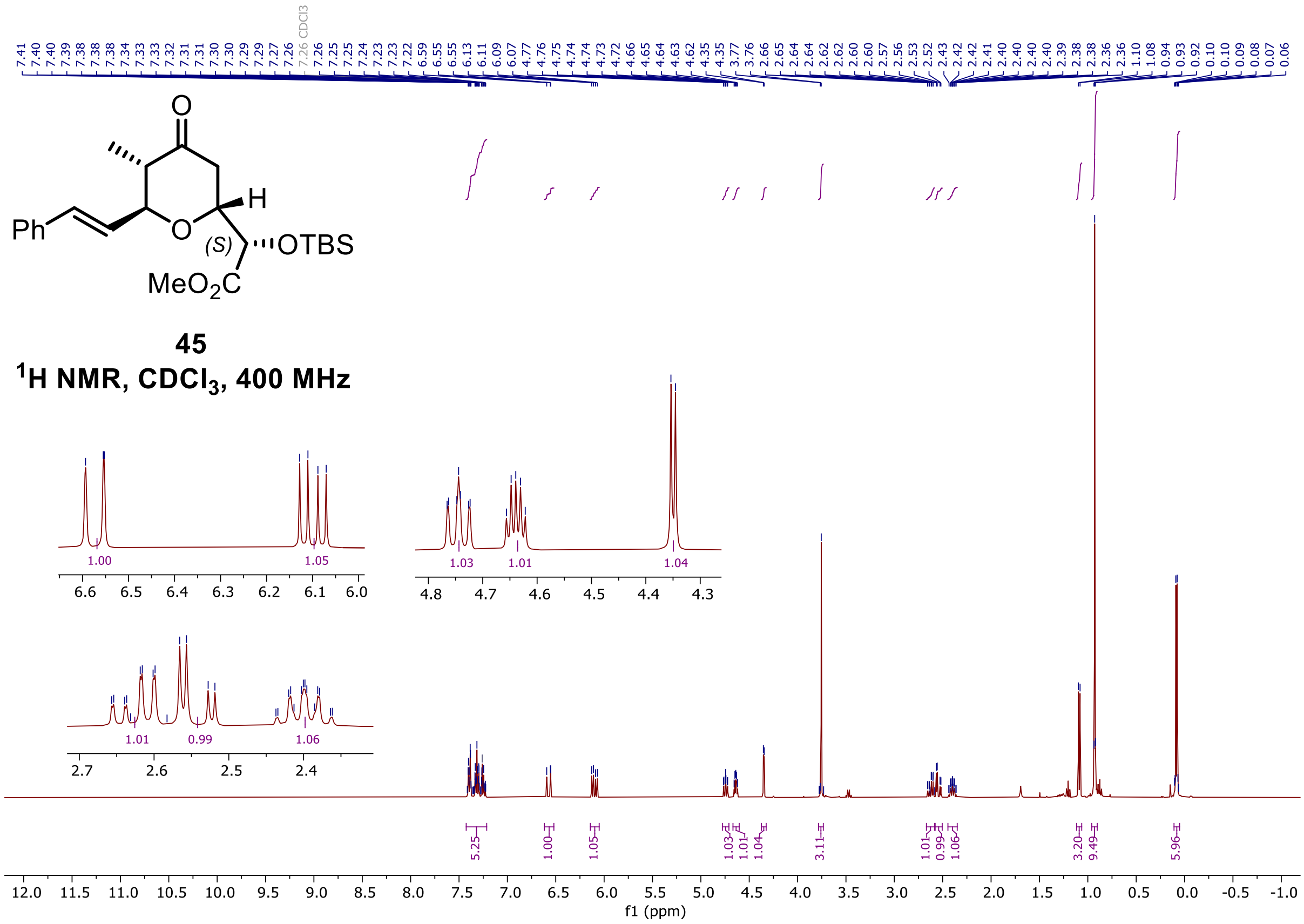

45

^{1}H NMR, $CDCl_3$, 400 MHz

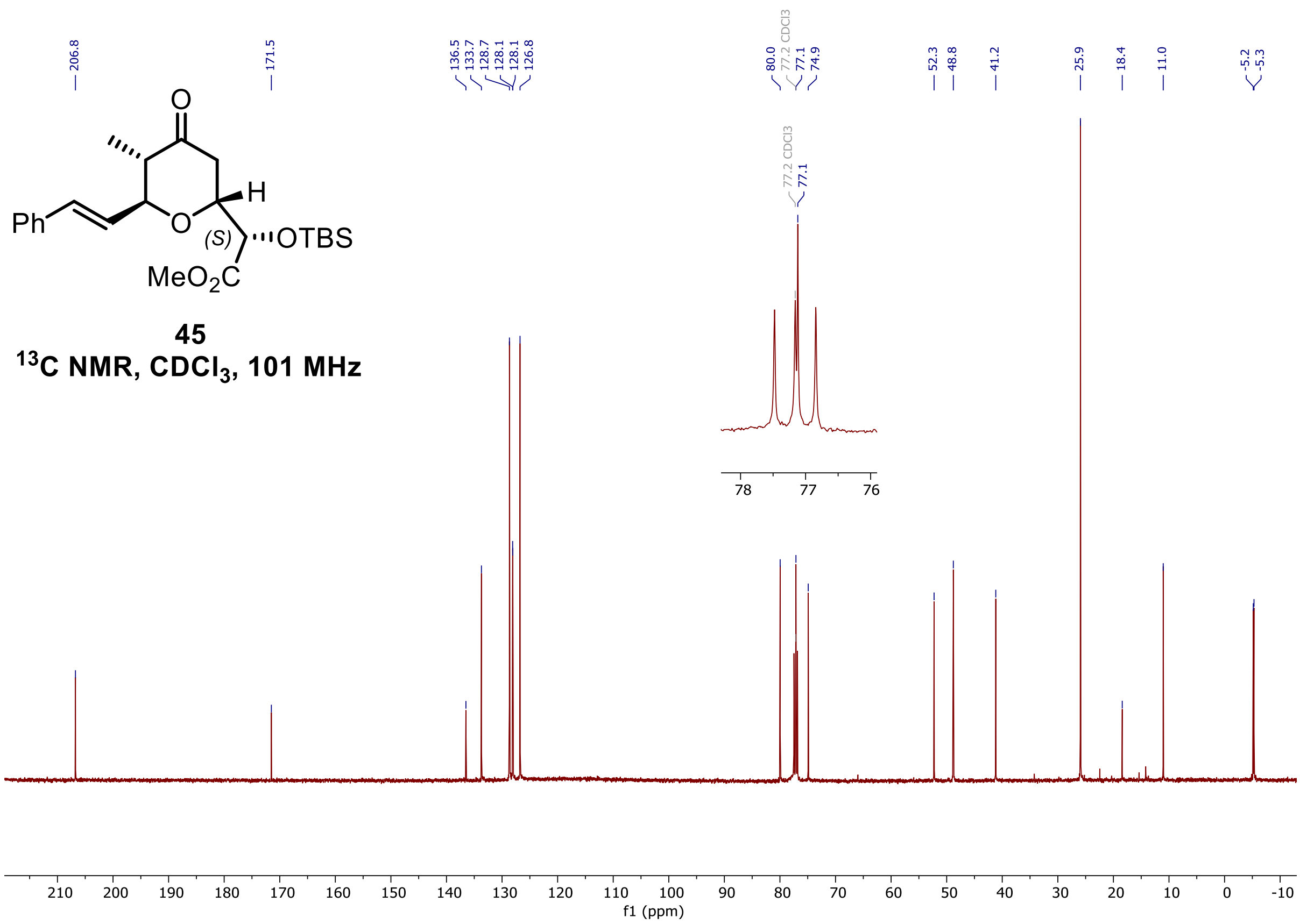

45

^{13}C NMR, $CDCl_3$, 101 MHz

Diol 46

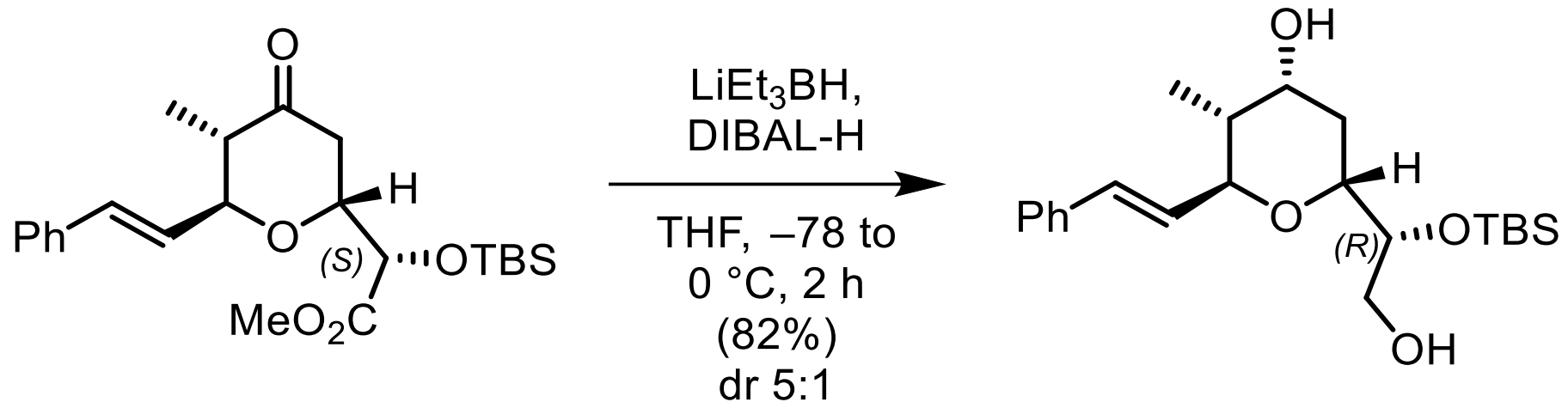

45 **46**

Ester **45** (1.66 g, 3.97 mmol, 1.0 eq.) was dissolved in 20 mL dry THF and cooled to –78 °C, before $LiEt_3BH$ (1M in THF, 4.17 mL, 4.17 mmol, 1.05 eq.) was slowly added. After 45 min, DIBAL-H (1M in *n*-hexane, 11.91 mL, 11.91 mmol, 3.0 eq.) was added and the mixture was stirred for additional 30 min. The temperature was raised to 0 °C and stirring was continued for another 45 min. The reaction was quenched with sat. NH_4Cl solution, followed by a 10% (w/w) potassium/sodium tartrate solution. The mixture was stirred vigorously for 1 h at r.t., the phases were separated and the aqueous layer was extracted with Et_2O. After drying the combined organic layer over Na_2SO_4 and removing the solvent in vacuo, the crude product was purified by flash chromatography (Et_2O/pentane 4:1) to afford diol **46** (1.28 g, 3.26 mmol, 82%, dr 5:1) as colorless oil.

$\mathbf{R_f}$ = 0.45 (pentane/Et_2O, 1:4), vanillin, UV (+)

^{1}H NMR (400 MHz, $CDCl_3$) δ [ppm] = 7.43 – 7.19 (m, 5H), 6.63 – 6.55 (m, 1H), 6.23 – 6.13 (m, 1H), 4.42 (td, *J* = 6.7, 1.4 Hz, 1H), 4.01 (dt, *J* = 6.4, 4.0 Hz, 1H), 3.92 – 3.63 (m, 4H), 2.54 (br s, 2H), 2.11 – 1.65 (m, 3H), 1.03 (d, *J* = 7.1 Hz, 3H), 0.90 (s, 9H), 0.09 (s, 3H), 0.08 (s, 3H).

^{13}C NMR (101 MHz, $CDCl_3$) δ [ppm] = 136.8, 132.5, 129.1, 128.7, 127.8, 126.6, 76.1, 74.1, 71.3, 66.7, 66.0, 64.2, 39.1, 32.9, 26.0, 26.0, 18.4, 15.4, 13.1, -5.2, -5.3.

IR (KBr pressel) ṽ (cm^{-1}) = 3854 (w), 3382 (s), 3029 (m), 2928 (s), 2368 (w), 1945 (w), 1634 (w), 1471 (s), 1372 (s), 1252 (s), 1123 (s), 1074 (s), 968 (s), 925 (m), 832 (s), 782 (s), 707 (m), 569 (w), 536 (w), 459 (w)

HRMS (ESI) (*m/z*) calcd. for $[C_{22}H_{36}O_4Si]^+$ = $[M+H]^+$: 393.2461, found: 393.2458.

Specific Rotation $[\alpha]_{589}^{20}$= –19.0° (c = 0.035, $CHCl_3$)

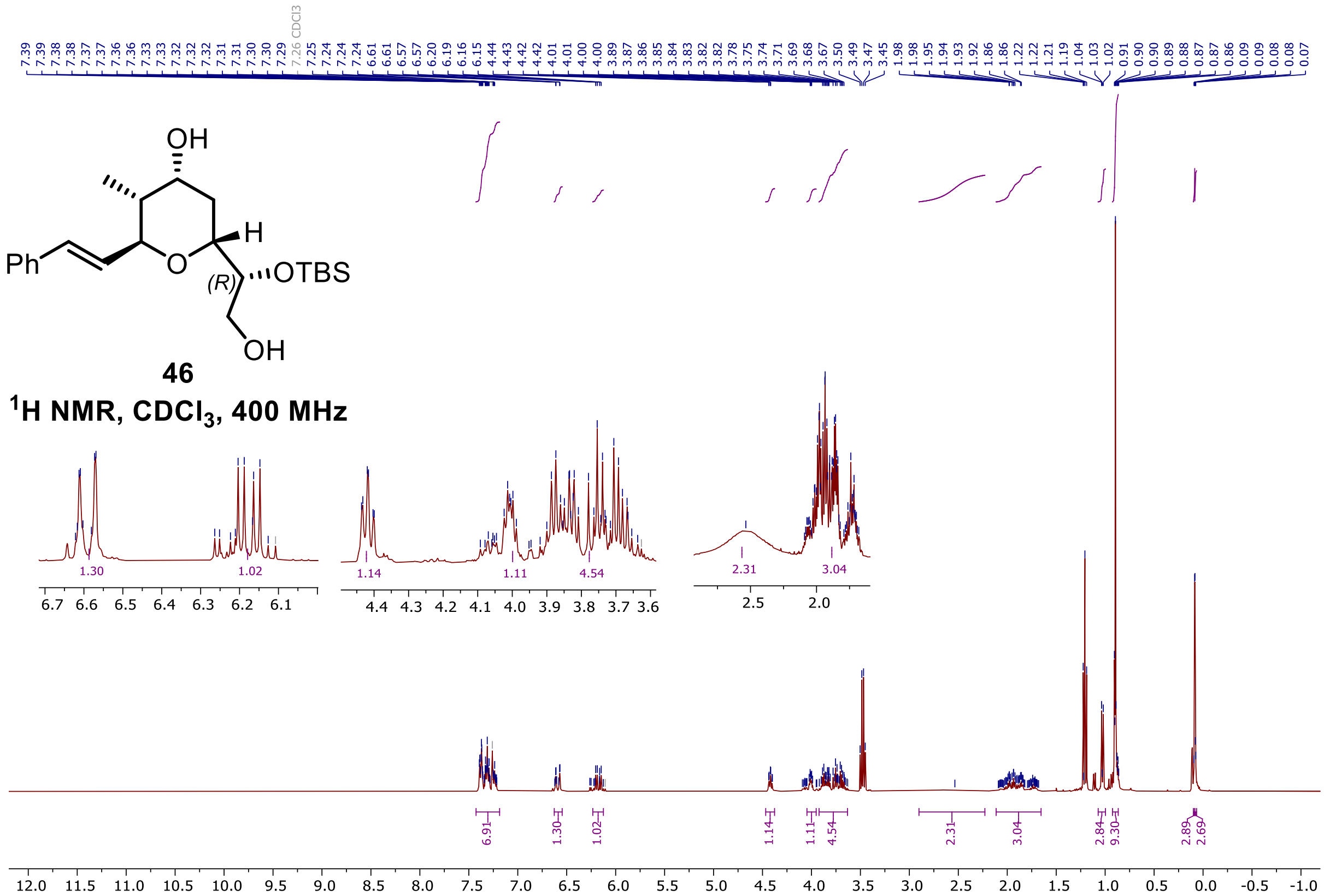

46

^{1}H NMR, CDCl$_3$, 400 MHz

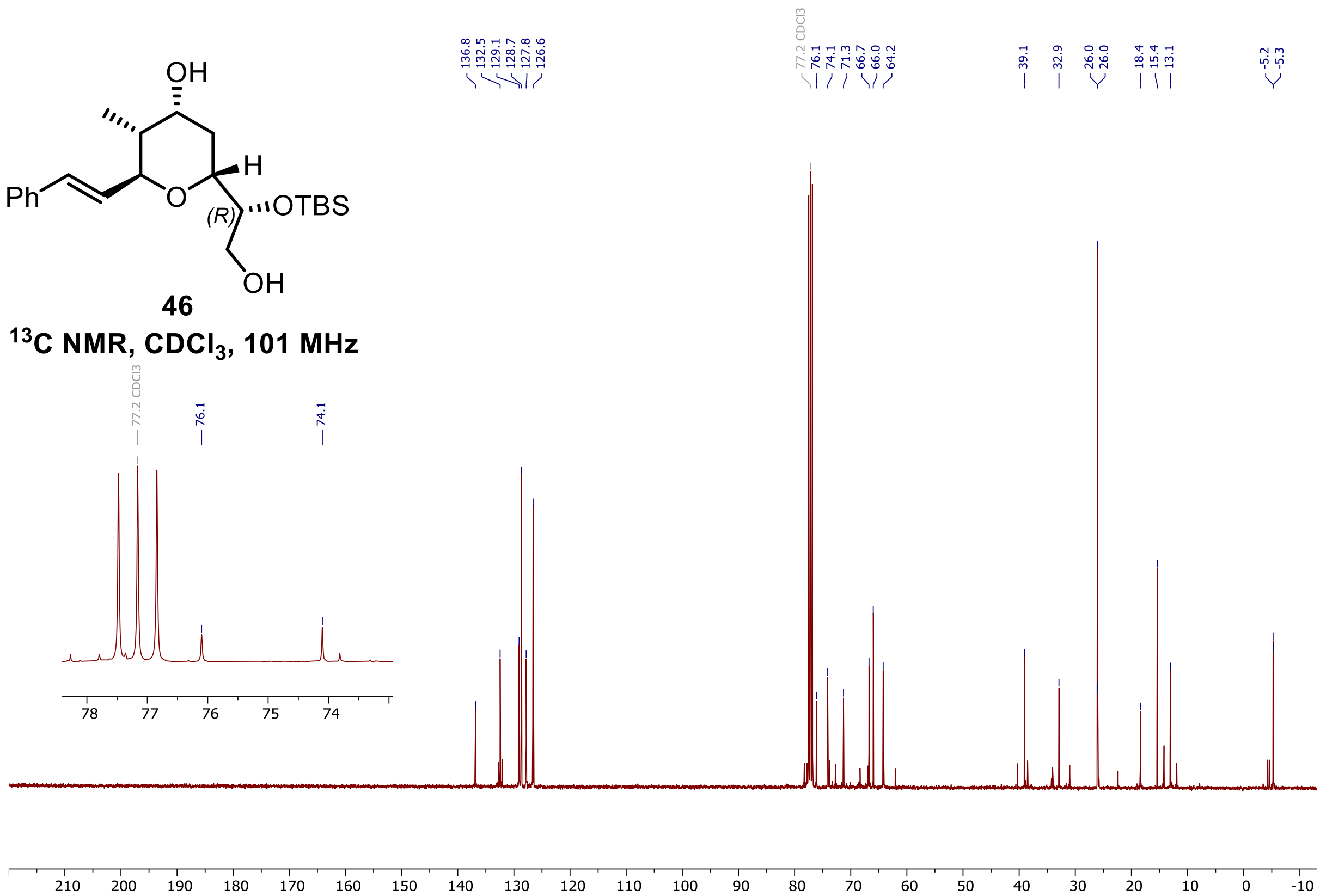

46

^{13}C NMR, CDCl$_3$, 101 MHz

Diol 47

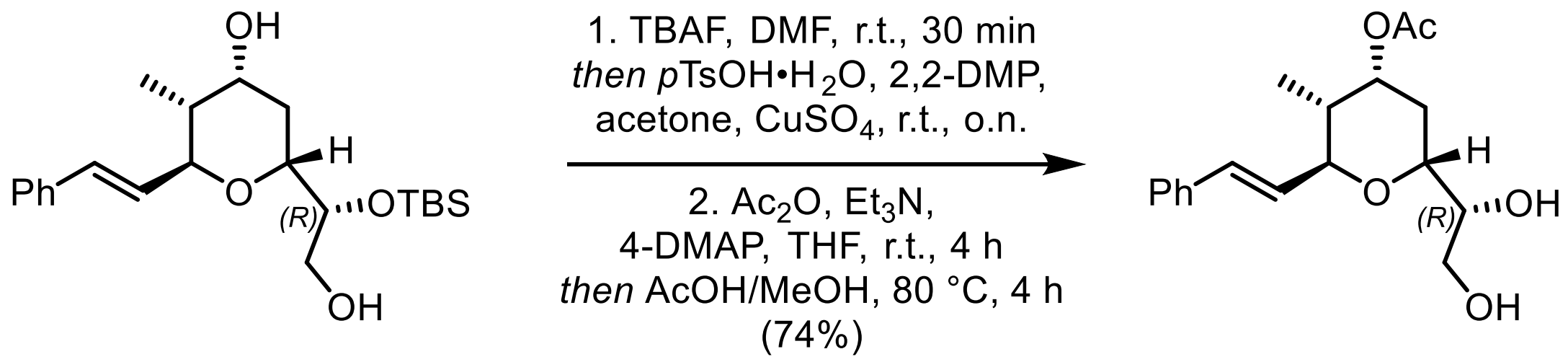

Diol **46** (460 mg, 1.17 mmol, 1.0 eq.) was dissolved in 7 mL dry DMF and TBAF (1M in THF, 1.75 mL, 1.75 mmol, 1.5 eq.) was added at r.t. After stirring for 30 min, *p*TsOH•H_2O (676 mg, 3.55 mmol, 3.0 eq.), 3.5 mL acetone, 2,2-DMP (2.93 mL, 23.90 mmol, 20.4 eq.), and anhydrous $CuSO_4$ (1.51 g, 9.46 mmol, 8.0 eq.) were added and the resulting mixture was stirred overnight. The reaction solution was slowly added to a sat. $NaHCO_3$ solution and diluted with Et_2O. After filtering off the excess of $CuSO_4$, the phases were separated and the organic phase was washed with water and sat. NaCl solution, and dried over $MgSO_4$. After removing the solvent in vacuo, the residue was dissolved in 1.5 mL dry THF, before addition of acetic anhydride (220 µL, 2.33 mmol, 2.0 eq.), Et_3N (285 µL, 2.04 mmol, 1.75 eq.), 4-DMAP (10 mg, 0.08 mmol, 7 mol%) and the mixture was stirred for 4 h at r.t. After adding 0.2 mL MeOH, stirring was continued for additional 30 min and acetic acid (70%, 15 mL) was added. The mixture was heated to 80 °C for 4 h, cooled to r.t., concentrated in vacuo and the remaining water and acetic acid were removed by azeotropically drying with toluene. Purification of the crude material by flash chromatography (pentane/EtOAc, 1:2) yielded diol **47** (276 mg, 0.86 mmol, 74%) as a colorless oil.

$\mathbf{R_f}$ = 0.31 (Et_2O), vanillin, UV (+)

^{1}H NMR (400 MHz, $CDCl_3$) δ [ppm] = 7.43 – 7.22 (m, 5H), 6.65 (dd, *J* = 16.2, 1.7 Hz, 1H), 6.25 (dd, *J* = 16.2, 5.3 Hz, 1H), 5.20 (dt, *J* = 9.9, 4.5 Hz, 1H), 4.43 (ddd, *J* = 5.3, 3.5, 1.8 Hz, 1H), 3.91 (ddd, *J* = 9.4, 5.5, 3.6 Hz, 1H), 3.76 – 3.70 (m, 2H), 3.65 (dd, *J* = 12.3, 5.9 Hz, 1H), 2.72 (s, 3H), 2.25 (qq, *J* = 6.6, 3.4 Hz, 1H), 2.07 (s, 3H), 1.91 (dt, *J* = 13.1, 9.8 Hz, 1H), 1.10 (d, *J* = 7.0 Hz, 3H).

^{13}C NMR (101 MHz, $CDCl_3$) δ [ppm] = 170.6, 136.4, 133.2, 128.8, 128.7, 128.1, 127.7, 126.7, 77.9, 73.1, 71.6, 69.4, 63.9, 35.5, 28.4, 21.3, 12.5.

IR (KBr pressel) ṽ (cm^{-1}) = 3443 (s), 3082 (w), 2939 (s), 2882 (s), 1732 (s), 1662 (m), 1600 (w), 1577 (w), 1545 (m), 1496 (m), 1448 (s), 1372 (s), 1239 (s), 1105 (s), 1034 (s), 988 (s), 878 (m), 761 (m), 695 (s), 565 (m), 521 (w), 485 (m)

HRMS (ESI) (*m/z*) calcd. for $[C_{18}H_{24}O_5]^+$ = $[M+H]^+$: 321.1702, found: 321.1710.

Specific Rotation $[\alpha]_{589}^{20}$= –51.1° (c = 0.65, $CHCl_3$)

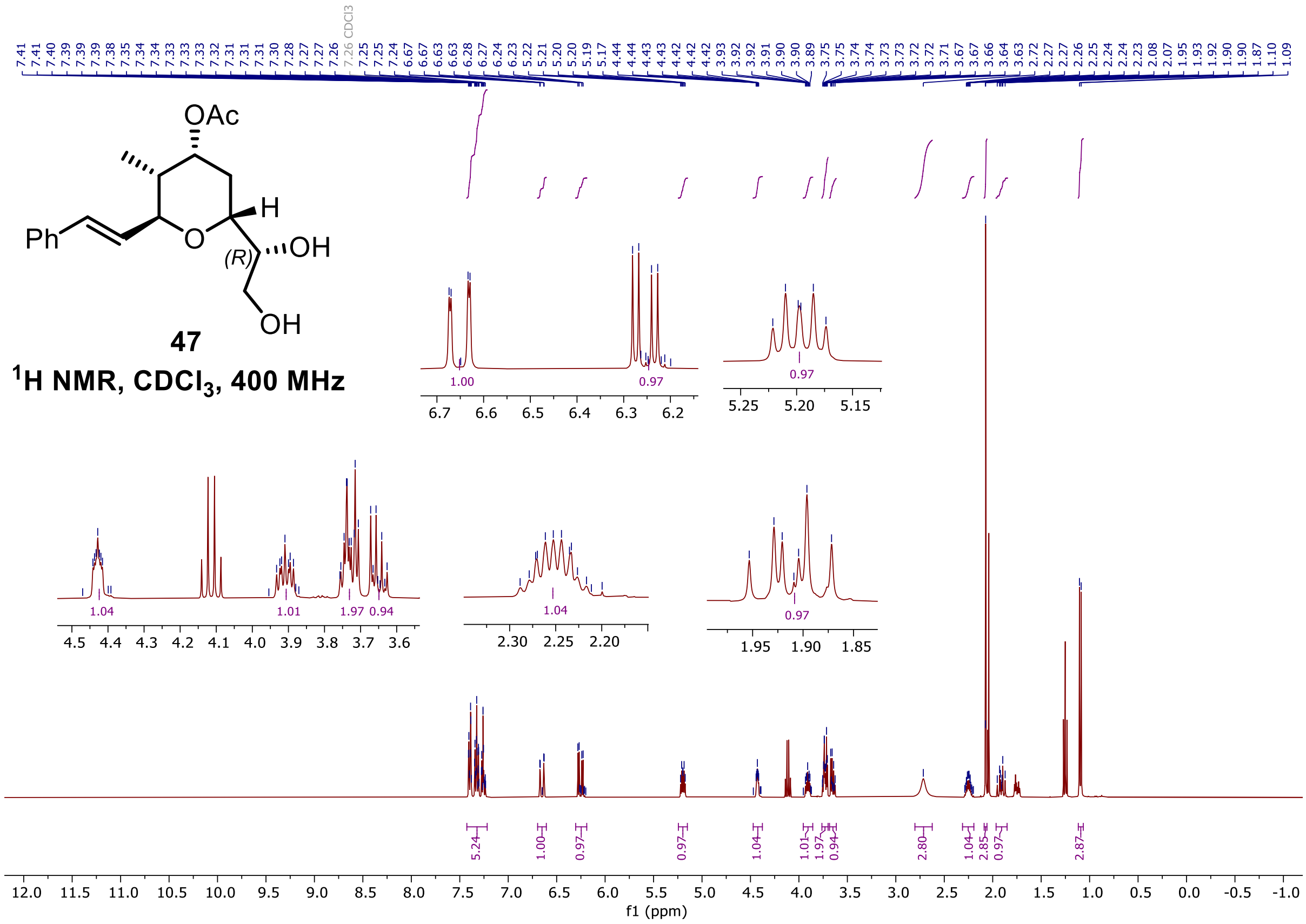
OAc
H
Ph
O
(R)
OH
OH
47
^{1}H NMR, CDCl$_3$, 400 MHz
f1 (ppm)

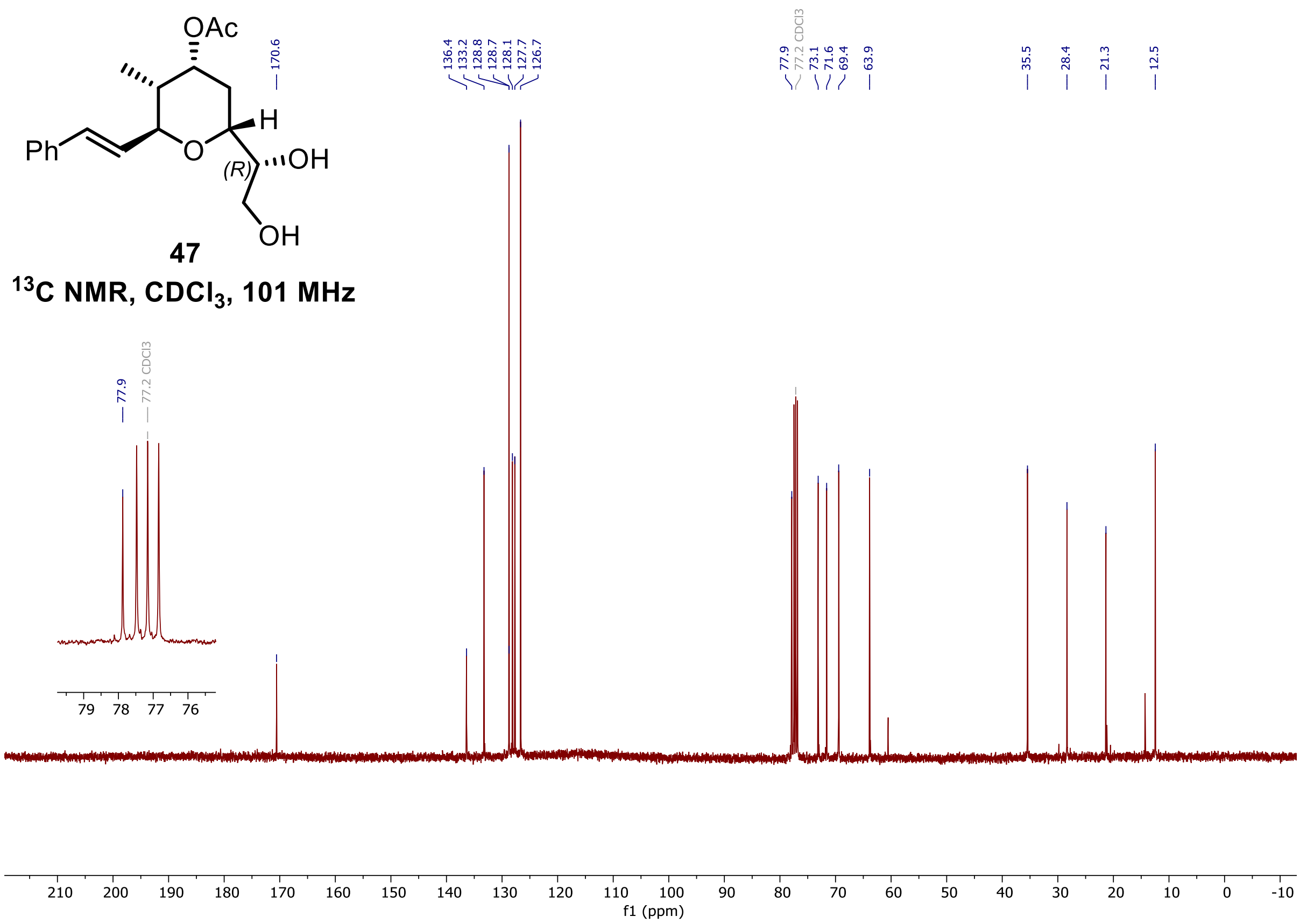
OAc
H
Ph
O
(R)
OH
OH
47
^{13}C NMR, CDCl$_3$, 101 MHz
f1 (ppm)

TIPS ether 8a

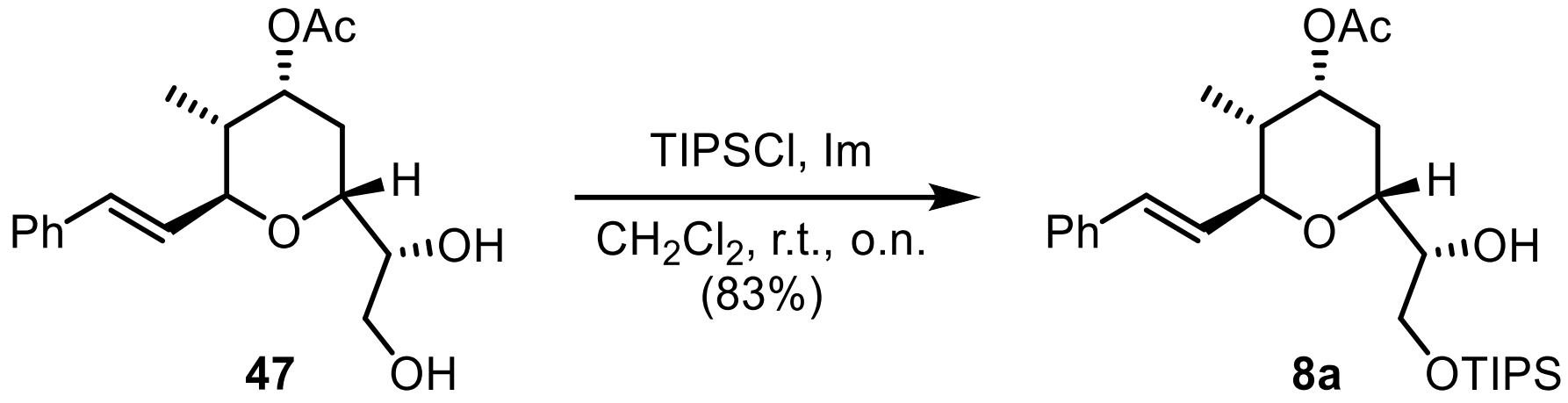

Diol **47** (235 mg, 733 µmol, 1.0 eq.) and imidazole (101 mg, 1.48 mmol, 2.0 eq.) were dissolved in 3.5 mL dry CH_2Cl_2. TIPSCl (200 µL, 942 mmol, 1.3 eq.) was added and the mixture was stirred overnight at r.t. Next, sat. NH_4Cl solution was added, the layers were separated and the aqueous phase was extracted with CH_2Cl_2. After drying the combined organic phases over Na_2SO_4, the solvent was removed in vacuo and the residue was purified by flash chromatography (pentane/Et_2O, 6:1), to afford TIPS ether **8a** (291 mg, 610 µmol, 83%) as a colorless oil.

R_f = 0.77 (pentane/Et_2O, 2:1), vanillin, UV (+)

^{1}H NMR (400 MHz, $CDCl_3$) δ [ppm] = 7.42 – 7.20 (m, 5H), 6.66 (dd, *J* = 16.3, 1.8 Hz, 1H), 6.28 (dd, *J* = 16.3, 5.1 Hz, 1H), 5.22 (dt, *J* = 10.4, 4.6 Hz, 1H), 4.45 (ddd, *J* = 5.0, 2.9, 1.8 Hz, 1H), 3.97 (ddd, *J* = 10.2, 4.8, 3.4 Hz, 1H), 3.81 – 3.65 (m, 3H), 2.61 (s, 1H), 2.35 – 2.23 (m, 1H), 2.08 (s, 3H), 2.02 – 1.89 (m, 1H), 1.74 (dt, *J* = 12.8, 3.9 Hz, 1H), 1.12 (d, *J* = 7.0 Hz, 3H), 1.12 – 0.95 (m, 21H).

^{13}C NMR (101 MHz, $CDCl_3$) δ [ppm] = 170.6, 136.6, 132.9, 128.7, 128.2, 127.9, 126.7, 78.1, 73.6, 70.1, 69.9, 64.1, 35.4, 28.4, 21.4, 18.1, 12.5, 12.0.

IR (film) ṽ (cm^{-1}) = 3475 (m), 2942 (s), 2866 (s), 2725 (w), 1740 (s), 1496 (m), 1463 (s), 1449 (s), 1383 (s), 1367 (s), 1333 (m), 1241 (s), 1109 (s), 1061 (s), 1034 (s), 1015 (s), 992 (s), 971 (s), 917 (m), 883 (s), 803 (m), 758 (m), 714 (m), 690 (s), 660 (s), 561 (w)

HRMS (ESI) (*m/z*) calcd. for $[C_{27}H_{45}O_5Si]^+$ = $[M+H]^+$: 477.3036, found: 477.3031.

Specific Rotation $[\alpha]_{589}^{20}$= –46°(c = 0.95, $CHCl_3$)

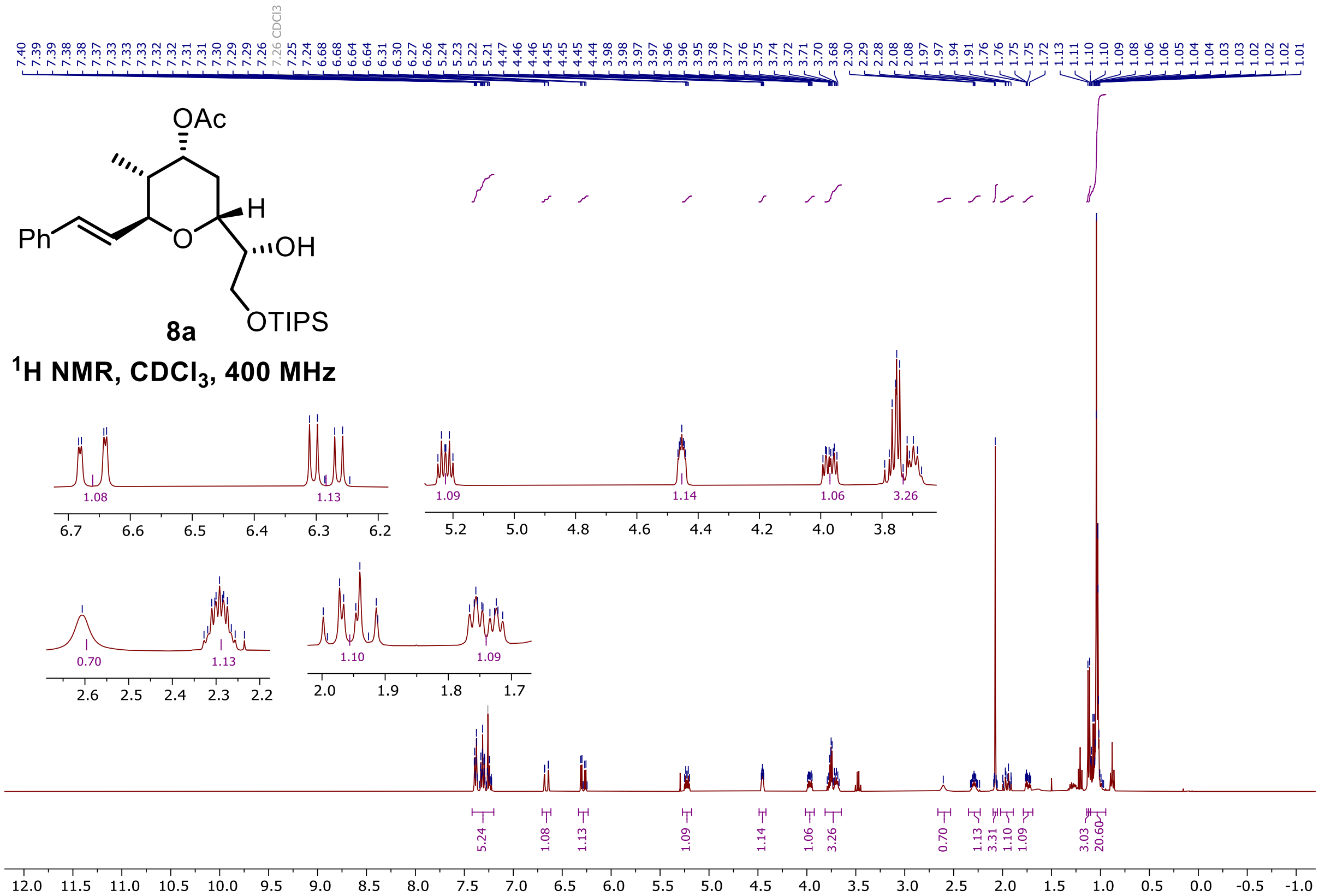
OAc
H
Ph
O
OH
OTIPS
8a
1H NMR, CDCl3, 400 MHz

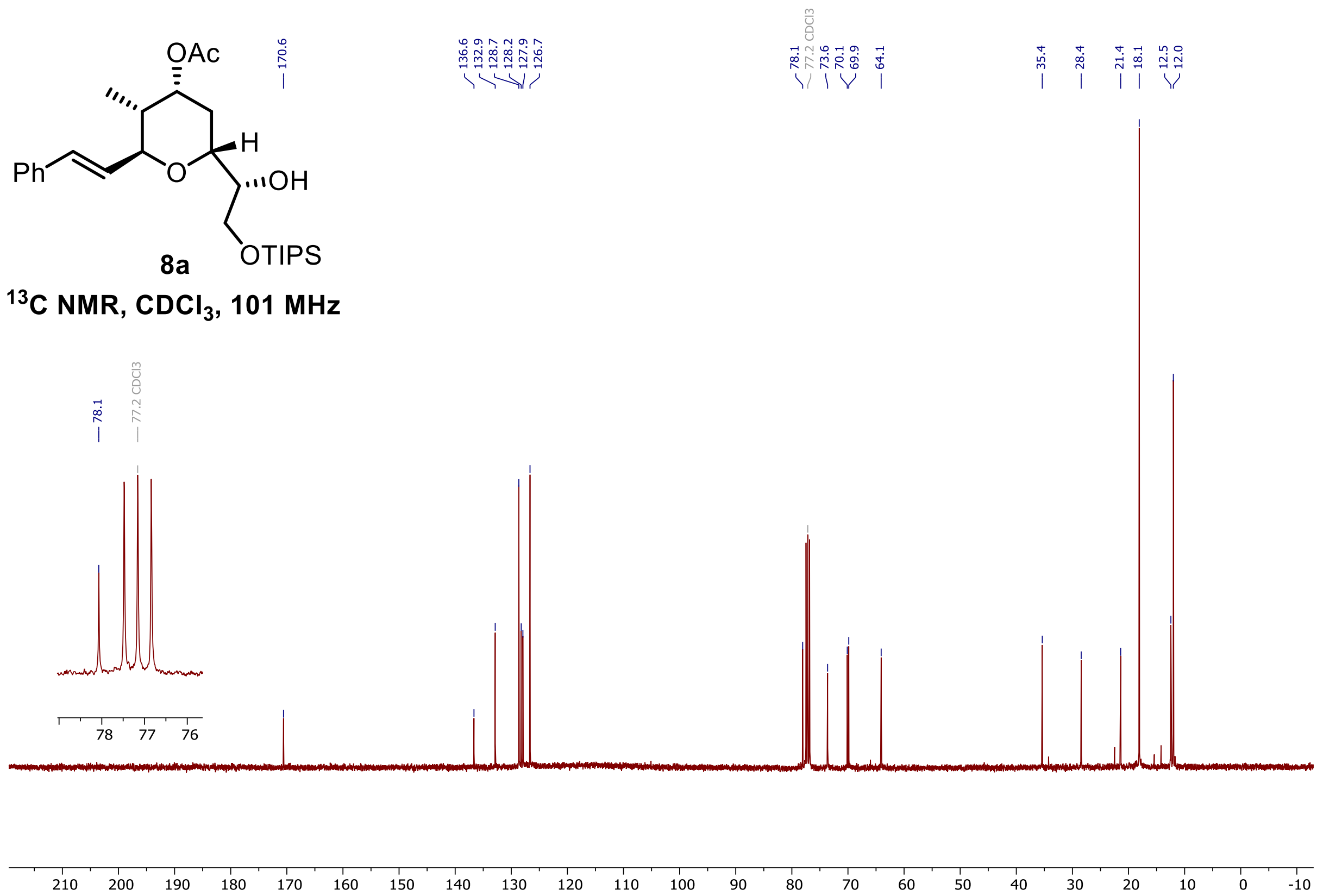
OAc
H
Ph
O
OH
OTIPS
8a
13C NMR, CDCl3, 101 MHz

Triflate 8

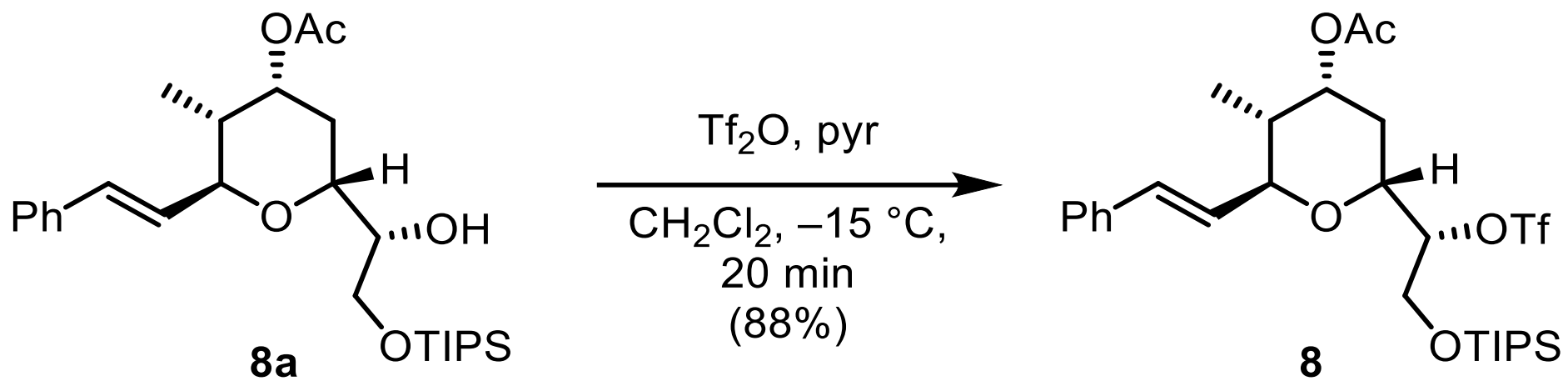

A solution of TIPS ether **8a** (256 mg, 537 µmol, 1.0 eq.) and pyridine (130 µL, 1.61 mmol, 3.0 eq.) in 5 mL dry CH_2Cl_2 was cooled to –15 °C. Next, Tf_2O (130 µL, 773 mmol, 1.4 eq.) was added and the mixture was stirred for 20 min at –15 °C. After completion of the reaction, it was quenched with 1N HCl, washed with sat. $NaHCO_3$ solution and sat. NaCl solution. The organic phase was dried over Na_2SO_4, the solvent was removed under reduced pressure and the crude material was azeotropically dried with toluene. Notably, a long drying period should be omitted due to the decomposition tendency of the product. Eventually, 298 mg of crude triflate **8** were obtained, which after calculation was corrected to 286 mg (470 µmol, 88%).

Note: The triflate is unstable and undergoes rapid decomposition. Therefore, it should be used immediately after preparation. Due to this instability, acquisition of a ^{13}C NMR spectrum was not feasible.

^{1}H NMR (400 MHz, $CDCl_3$) δ [ppm] = 7.46 – 7.28 (m, 5H), 6.73 (dd, *J* = 16.2, 1.7 Hz, 1H), 6.21 (dd, *J* = 16.3, 5.3 Hz, 1H), 5.25 (td, *J* = 6.5, 4.0 Hz, 1H), 5.08 (dt, *J* = 6.5, 4.2 Hz, 1H), 4.48 (td, *J* = 5.0, 1.7 Hz, 1H), 4.33 (q, *J* = 6.4 Hz, 1H), 4.13 (dd, *J* = 11.7, 3.8 Hz, 1H), 3.99 – 3.92 (m, 1H), 2.23 (tt, *J* = 7.1, 3.4 Hz, 1H), 2.13 (s, 3H), 1.97 – 1.89 (m, 2H), 1.14 – 1.07 (m, 21H).

^{19}F NMR (377 MHz, $CDCl_3$) δ [ppm] = -75.0.

IR (film) ṽ (cm^{-1}) = 3440 (m), 3084 (m), 2945 (s), 2893 (s), 2868 (s), 1742 (s), 1600 (w), 1578 (w), 1496 (m), 1463 (s), 1411 (s), 1241 (s), 1145 (s), 1072 (s), 1030 (s), 970 (s), 922 (s), 883 (s), 775 (s), 760 (s), 691 (s), 666 (s), 639 (s), 514 (m), 463 (m)

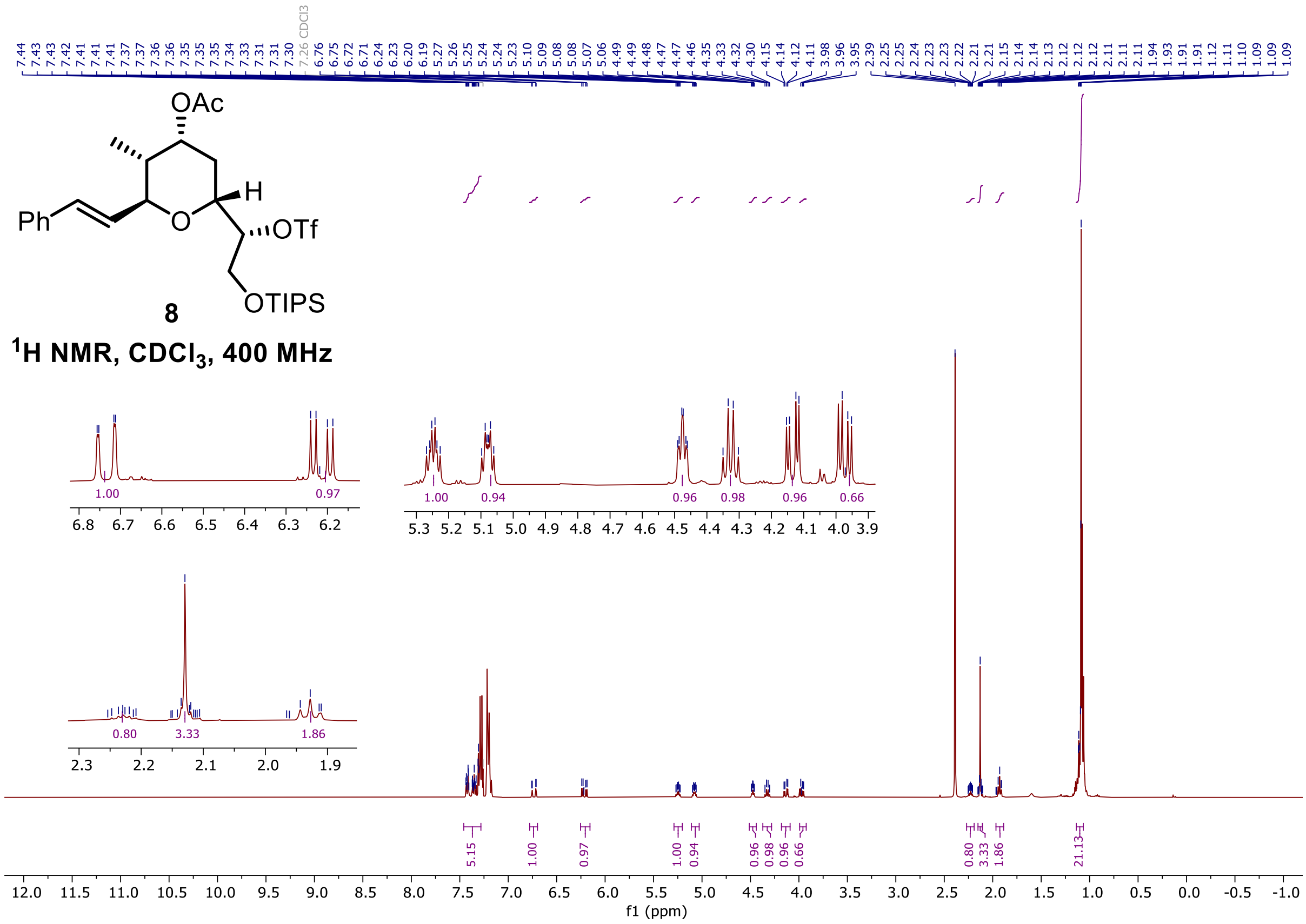
7.44 7.43 7.43 7.42 7.41 7.41 7.37 7.37 7.36 7.36 7.35 7.35 7.35 7.34 7.33 7.31 7.31 7.30 7.26 CDCl3 6.76 6.75 6.72 6.71 6.24 6.23 6.20 6.19 5.27 5.26 5.25 5.24 5.24 5.23 5.10 5.09 5.08 5.08 5.07 5.06 4.49 4.48 4.47 4.47 4.46 4.35 4.33 4.32 4.30 4.15 4.14 4.12 4.11 3.98 3.96 3.95 2.39 2.25 2.25 2.24 2.23 2.23 2.22 2.21 2.21 2.15 2.14 2.14 2.13 2.12 2.12 2.11 2.11 1.94 1.93 1.91 1.91 1.12 1.11 1.10 1.09 1.09 1.09
OAc
H
Ph
O
OTf
OTIPS
8
1H NMR, CDCl3, 400 MHz
1.00
0.97
0.94
0.96
0.98
0.96
0.66
0.80
3.33
1.86
5.15
21.13
f1 (ppm)

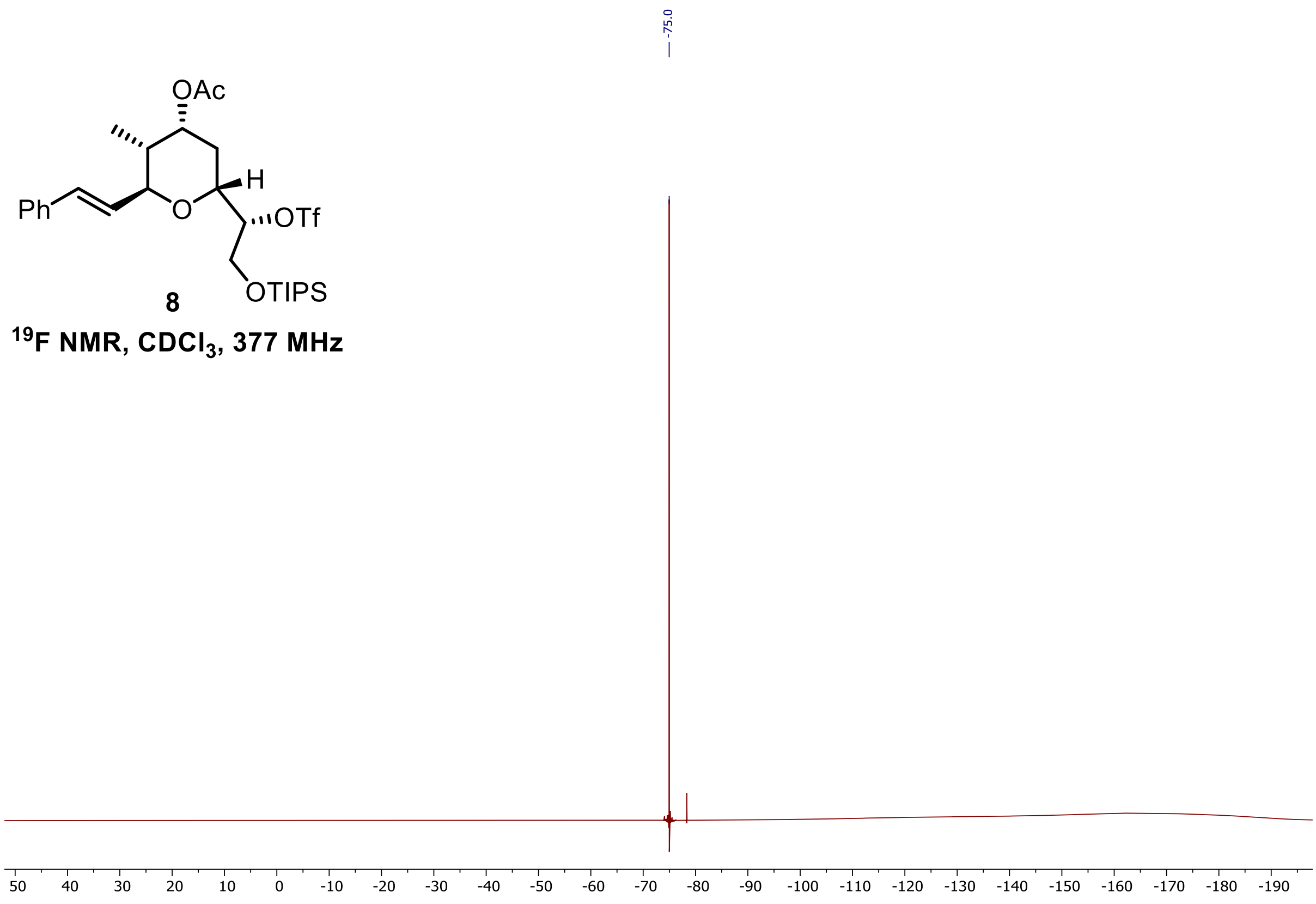
-75.0
OAc
H
Ph
O
OTf
OTIPS
8
19F NMR, CDCl3, 377 MHz

Dioxabicyclo[3.2.1]octane 49

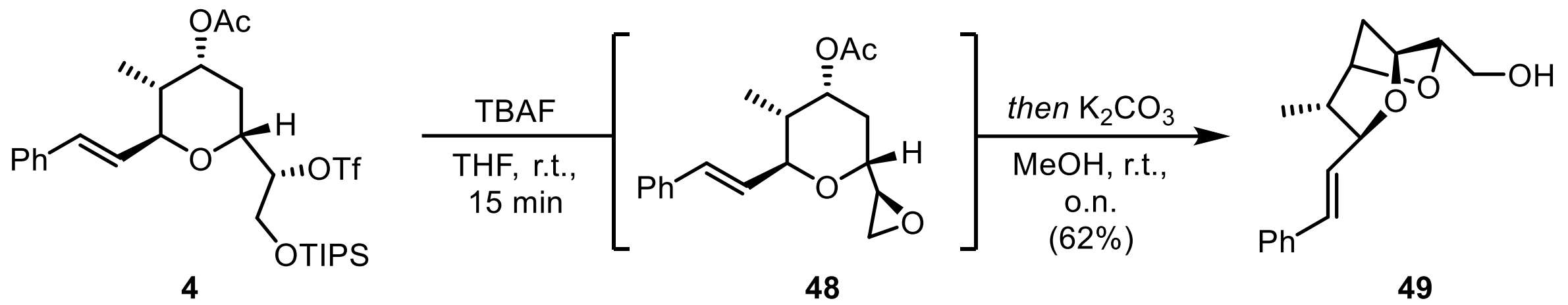

Triflate **8** (3.29 g, 5.40 mmol, 1.0 eq.) was dissolved in 40 mL dry THF and TBAF (1M in THF, 6.5 mL, 6.5 mmol, 1.2 eq.) was added at r.t. After stirring for 15 min at r.t., 40 mL MeOH were added, followed by dry K_2CO_3 (7.50 g, 54.26 mmol, 10.0 eq.). The mixture was stirred overnight, was subsequently diluted with water, the layers were separated and the aqueous phase was extracted with EtOAc. The combined organic layer was dried over Na_2SO_4, the solvent was removed in vacuo and the residue was purified by flash chromatography (pentane/EtOAc, 1:1) to afford bicyclooctane **49** (866 mg, 3.33 mmol, 62%) as a white solid.

R_f =0.22 (pentane/EtOAc, 1:1), vanillin, UV (+)

^{1}H NMR (400 MHz, $CDCl_3$) δ [ppm] = 7.41 – 7.20 (m, 5H), 6.58 (d, *J* = 15.8 Hz, 1H), 6.12 (dd, *J* = 15.8, 7.6 Hz, 1H), 4.45 (dt, *J* = 3.2, 1.7 Hz, 1H), 4.32 (d, *J* = 6.6 Hz, 1H), 4.15 – 4.02 (m, 3H), 4.00 – 3.91 (m, 1H), 2.14 – 2.03 (m, 1H), 1.95 (dd, *J* = 11.7, 1.6 Hz, 1H), 1.64 (br s, 1H), 1.53 (dtd, *J* = 9.1, 7.2, 6.2 Hz, 1H), 0.94 (d, *J* = 6.7 Hz, 3H).

^{13}C NMR (101 MHz, $CDCl_3$) δ [ppm] = 136.7, 132.7, 129.2, 128.7, 127.9, 126.7, 83.1, 79.9, 79.2, 74.6, 61.1, 41.8, 39.0, 15.4.

IR (KBr pressel) ṽ (cm^{-1}) = 3247 (m), 2976 (m), 2934 (m), 2888 (m), 1740 (w), 1496 (w), 1449 (m), 1371 (w), 1360 (w), 1239 (m), 1143 (s), 1078 (m), 1061 (s), 1037 (s), 962 (s), 929 (w), 746 (m), 693 (m), 544 (w)

HRMS (ESI) (*m/z*) calcd. for $[C_{16}H_{20}O_3]^+$ = $[M+H]^+$: 261.1491, found: 261.1488.

Specific Rotation $[\alpha]_{589}^{20}$= –55.0°(c = 1.0, CH_2Cl_2)

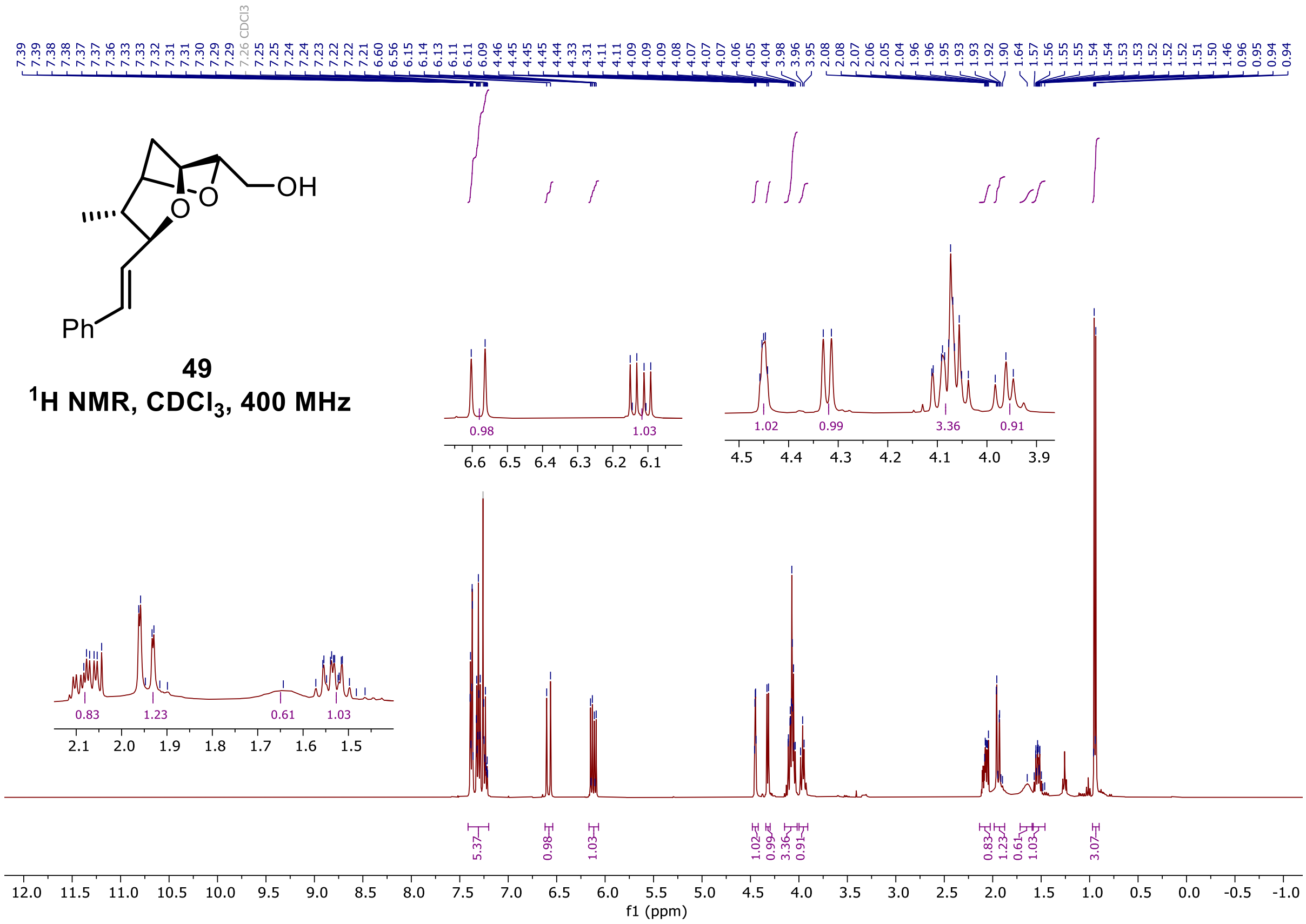
OH
O
O
Ph
49
^{1}H NMR, $CDCl_3$, 400 MHz
f1 (ppm)

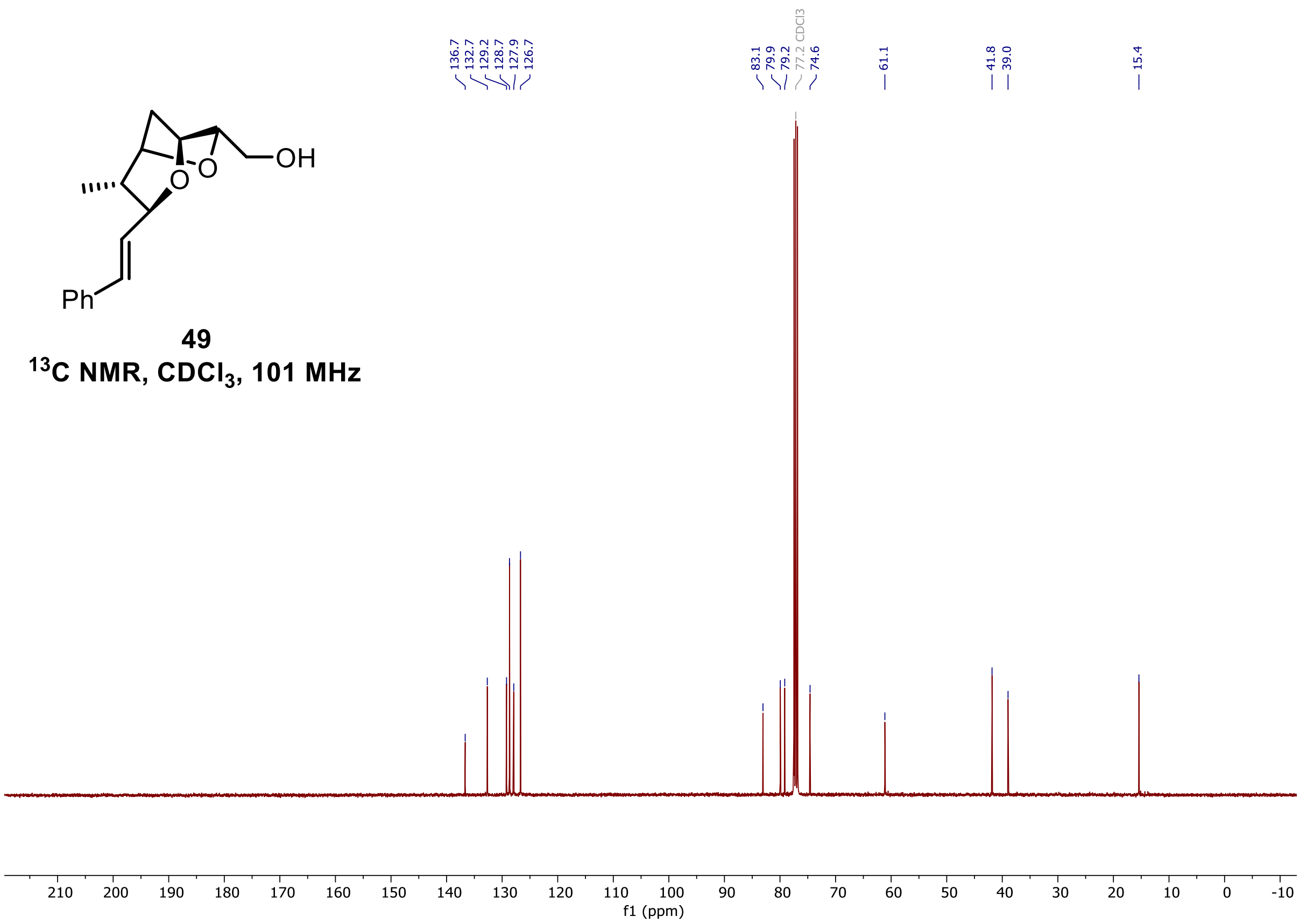
136.7
132.7
129.2
128.7
127.9
126.7
83.1
79.9
79.2
77.2 CDCl3
74.6
61.1
41.8
39.0
15.4
OH
O
O
Ph
49
^{13}C NMR, $CDCl_3$, 101 MHz
f1 (ppm)

MEM ether 50

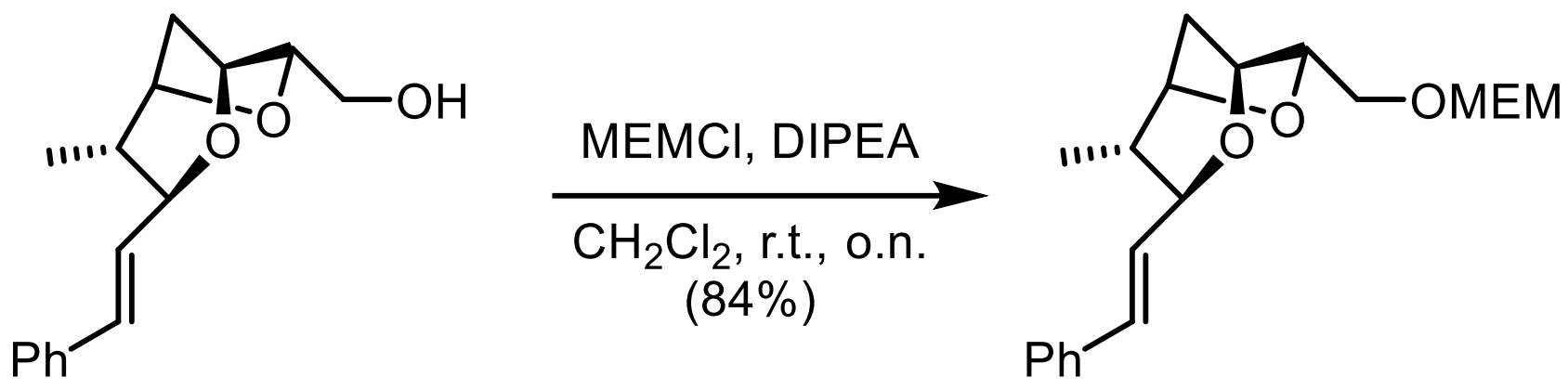

49 **50**

Bicyclooctane **49** (202 mg, 0.78 mmol, 1.0 eq.) was dissolved in 10 mL dry CH_2Cl_2 at r.t. and subsequently DIPEA (270 µL, 1.55 mmol, 2.0 eq.) and MEMCl (180 µL, 1.58 mmol, 2.0 eq.) were added dropwise. After stirring at r.t. overnight, the mixture was washed with 1N HCl and sat. $NaHCO_3$ solution. The organic phase was dried over Na_2SO_4, and the solvent was evaporated in vacuo. After flash chromatography (pentane/Et_2O, 1:2) MEM ether **50** (228 mg, 0.65 mmol, 84%) was obtained as a colorless oil.

$\mathbf{R_f}$ = 0.67 (Et_2O), vanillin, UV (+)

^{1}H NMR (400 MHz, $CDCl_3$) δ [ppm] = 7.41 – 7.18 (m, 5H), 6.58 (d, *J* = 15.8 Hz, 1H), 6.11 (dd, *J* = 15.8, 7.5 Hz, 1H), 4.81 (s, 2H), 4.42 (q, *J* = 2.2 Hz, 1H), 4.30 (d, *J* = 6.6 Hz, 1H), 4.13 – 4.02 (m, 2H), 4.00 – 3.90 (m, 2H), 3.77 – 3.68 (m, 3H), 3.60 – 3.53 (m, 3H), 3.40 – 3.37 (m, 4H), 2.06 (ddd, *J* = 11.7, 6.6, 2.7 Hz, 1H), 1.92 (dd, *J* = 11.8, 1.6 Hz, 1H), 1.57 – 1.46 (m, 1H), 1.20 (t, *J* = 7.0 Hz, 1H), 0.93 (d, *J* = 6.7 Hz, 3H).

^{13}C NMR (101 MHz, $CDCl_3$) δ [ppm] = 136.7, 132.6, 129.2, 128.7, 128.6, 127.8, 126.7, 95.7, 81.5, 79.8, 79.1, 74.9, 71.9, 66.8, 65.6, 59.2, 41.8, 38.8, 15.3.

IR (ATR) ṽ (cm^{-1}) = 3058 (w), 3026 (w), 2961 (m), 2931 (m), 2876 (m), 2817 (w), 1599 (w), 1578 (w), 1493 (w), 1450 (w), 1415 (w), 1398 (w), 1366 (w), 1345 (w), 1313 (w), 1299 (w), 1284 (w), 1243 (w), 1221 (w), 1199 (w), 1174 (w), 1110 (s), 1075 (s), 1043 (s), 1007 (s), 965 (s), 920 (m), 907 (m), 876 (m), 846 (m), 790 (w), 744 (m), 694 (m), 650 (w), 615 (w), 607 (w), 581 (w), 540 (w), 515 (w), 427 (w).

HRMS (ESI) (*m/z*) calcd. for [$C_{20}H_{28}O_5$] = [M]: 348.1937, found 348.1931.

Specific Rotation $[\alpha]_{589}^{20}$= –36.8° (c = 0.74, $CHCl_3$)

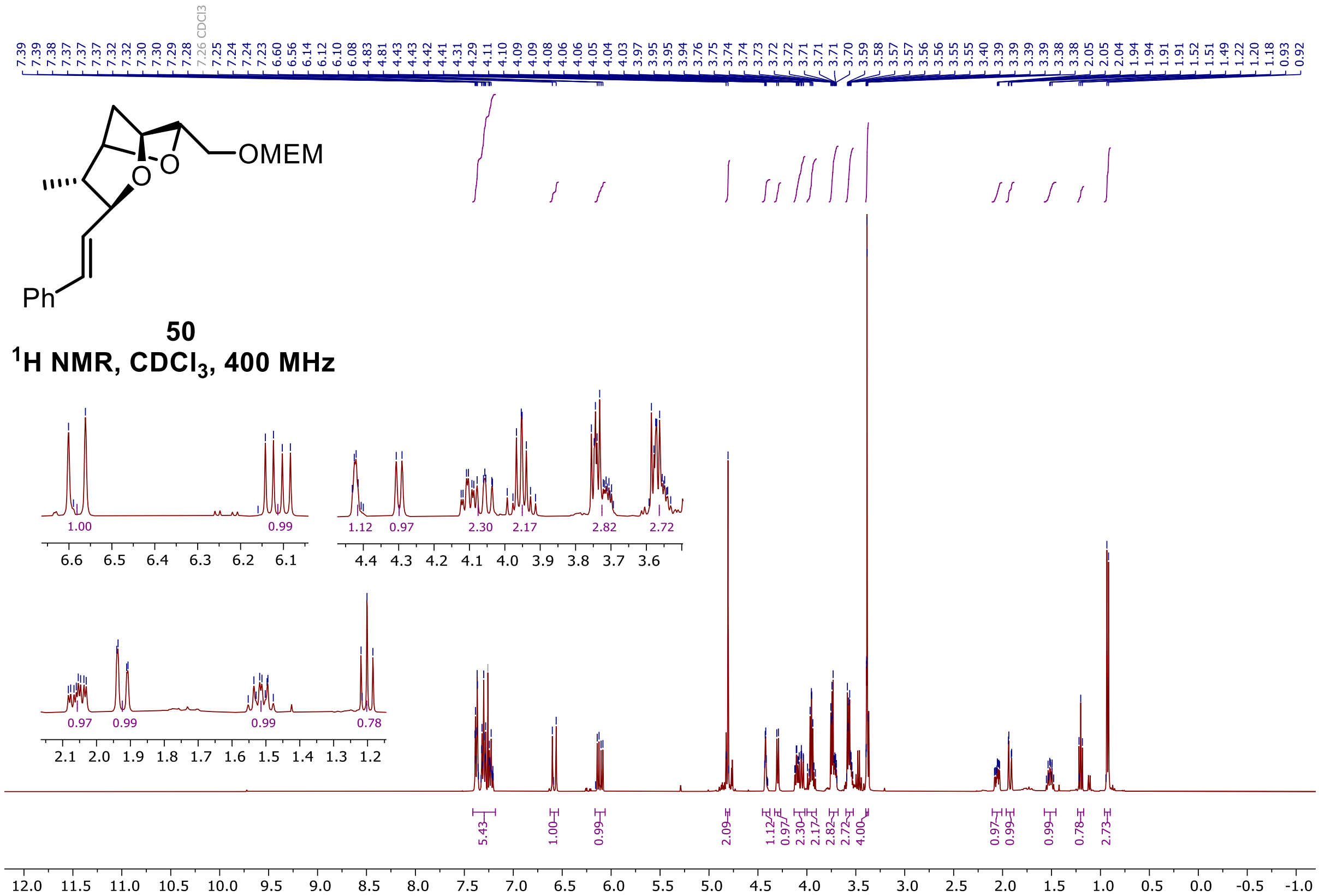

50

^{1}H NMR, CDCl$_3$, 400 MHz

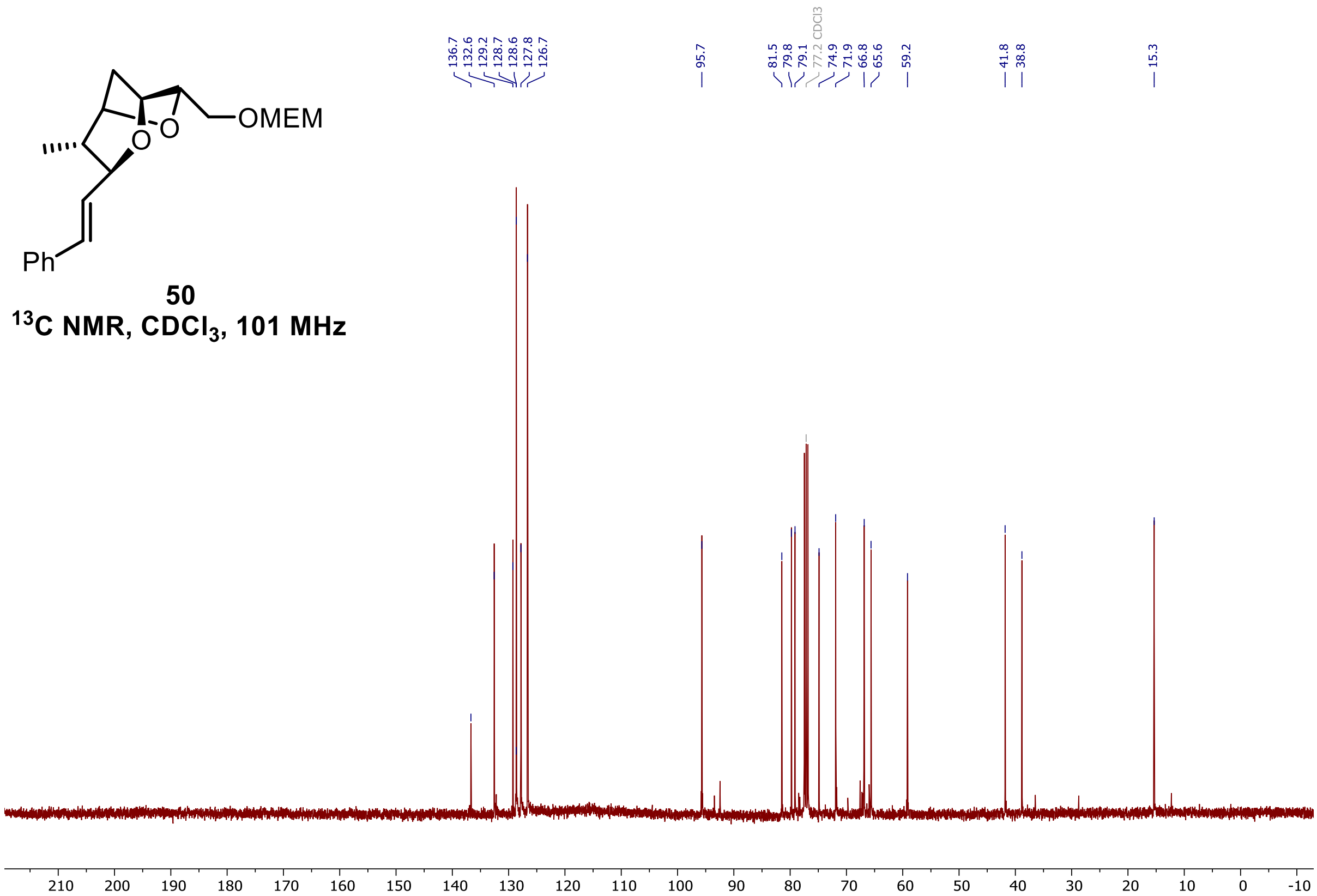

50

^{13}C NMR, CDCl$_3$, 101 MHz

BCO fragment 5

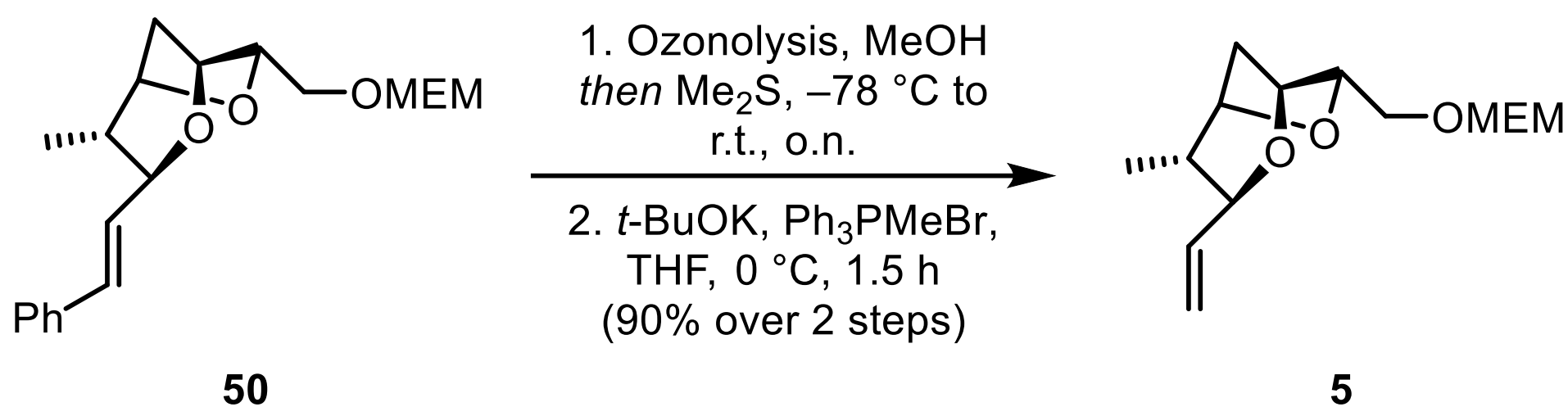

A solution of MEM ether **50** (1.12 g, 3.21 mmol, 1.0 eq.) in 32.5 mL MeOH was cooled to –78 °C and ozone was bubbled through the solution until it turned blue. Afterwards, oxygen was bubbled through for 15 min, followed by addition of Me_2S (4.78 mL, 64.6 mmol, 20.0 eq.). The reaction mixture was allowed to warm to r.t. and stirred overnight. After removal of all volatiles in vacuo, the intermediate aldehyde was used without further purification.
To a 0 °C cooled solution of *t*-BuOK (1.46 g, 13.0 mmol, 4.0 eq.) in 32 mL dry THF, was added Ph_3PMeBr (5.80 g, 16.2 mmol, 5.0 eq.) and the reaction was stirred for 30 min. Next, a solution of the crude aldehyde, dissolved in 8 mL dry THF, was added slowly. The mixture was stirred for 1 h at 0 °C, quenched with sat. NH_4Cl solution (10 mL) and diluted with CH_2Cl_2 (20 mL) and water (20 mL). The phases were separated and the aqueous layer was extracted with CH_2Cl_2 (2 x 20 mL). The combined organic layer was dried over Na_2SO_4, solvent was removed in vacuo and the crude material was purified via flash chromatography (30% EtOAc in hexane) to yield BCO fragment **5** (790 mg, 2.90 mmol, 90% over 2steps) as a colorless oil.

$\mathbf{R_f}$ = 0.23 (Et_2O), vanillin, UV (-)

^{1}H NMR (400 MHz, $CDCl_3$) δ [ppm] = 5.74 (ddd, *J* = 17.4, 10.2, 7.3 Hz, 1H), 5.22 (ddd, *J* = 17.1, 1.7, 1.0 Hz, 1H), 5.14 (ddd, *J* = 10.2, 1.7, 0.8 Hz, 1H), 4.78 (s, 2H), 4.41 – 4.35 (m, 1H), 4.26 (d, *J* = 6.6 Hz, 1H), 4.07 (td, *J* = 6.1, 2.2 Hz, 1H), 3.92 – 3.82 (m, 3H), 3.76 – 3.67 (m, 2H), 3.58 – 3.54 (m, 2H), 3.39 (s, 3H), 2.02 (ddd, *J* = 11.6, 6.6, 2.7 Hz, 1H), 1.86 (dd, *J* = 11.7, 1.6 Hz, 1H), 1.46 – 1.34 (m, 1H), 0.88 (d, *J* = 6.8 Hz, 3H).

^{13}C NMR (101 MHz, $CDCl_3$) δ [ppm] = 138.0, 117.3, 95.7, 81.4, 80.1, 79.1, 74.7, 71.9, 66.8, 65.6, 59.2, 41.3, 38.8, 15.2.

IR (ATR) ṽ (cm^{-1}) = 3078 (w), 3018 (w), 2932 (m), 2876 (m), 2818 (w), 1646 (w), 1454 (w), 1423 (w), 1365 (w), 1345 (w), 1285 (w), 1243 (w), 1221 (w), 1175 (w), 1112 (s), 1076 (s), 1044 (s), 918 (s), 951 (m), 918 (m), 900 (m), 874 (m), 848 (m), 827 (w), 801 (w), 775 (w), 638 (w), 598 (w), 545 (w), 519 (w), 436 (w), 418 (w).

HRMS (ESI) (*m/z*) calcd. for $[C_{14}H_{24}O_5]^+$ = $[M+H]^+$: 272.1624, found: 272.1622.

Specific Rotation $[\alpha]_{589}^{20}$= -67.5° (c = 0.45, $CHCl_3$)

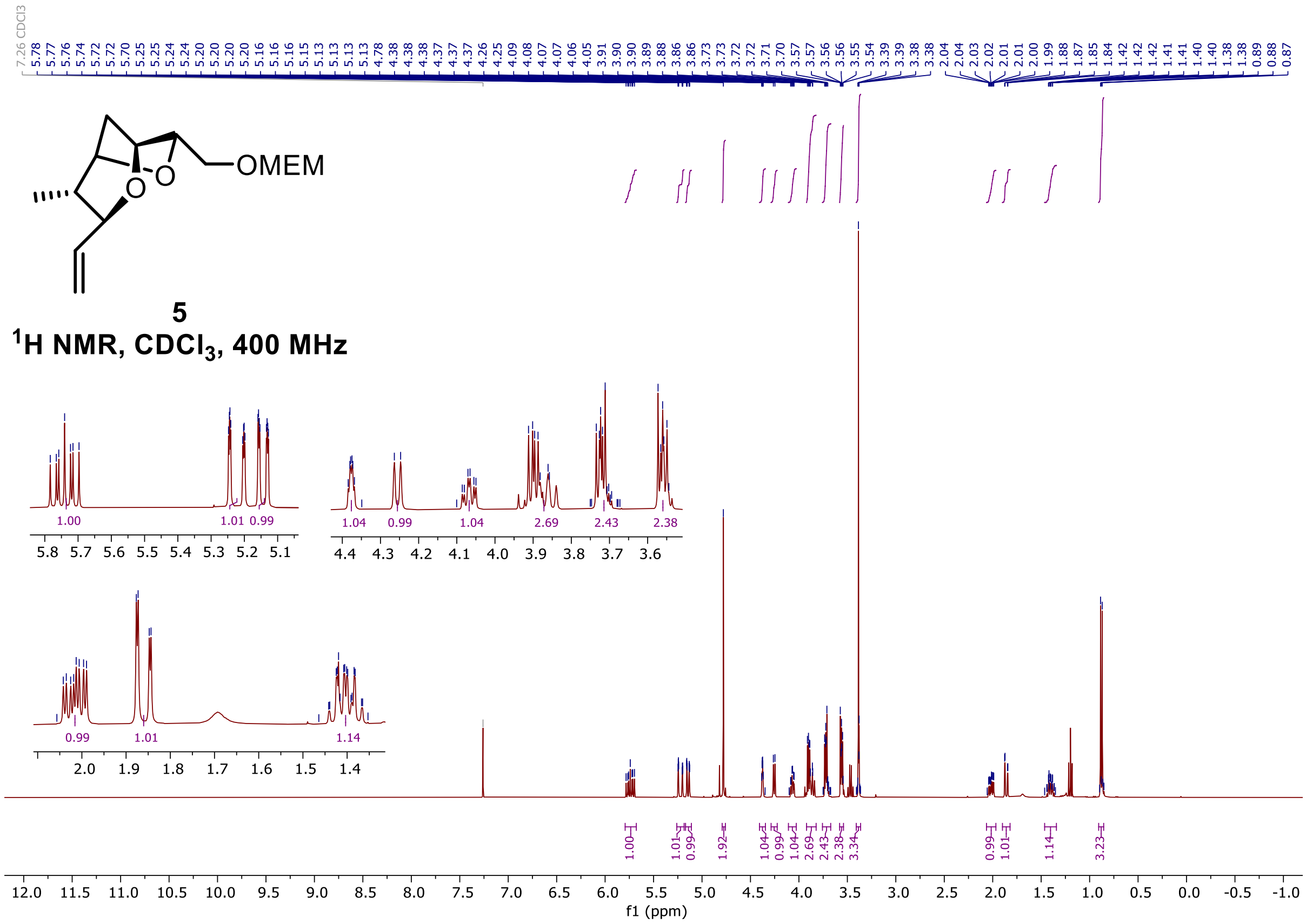

5

^{1}H NMR, $CDCl_3$, 400 MHz

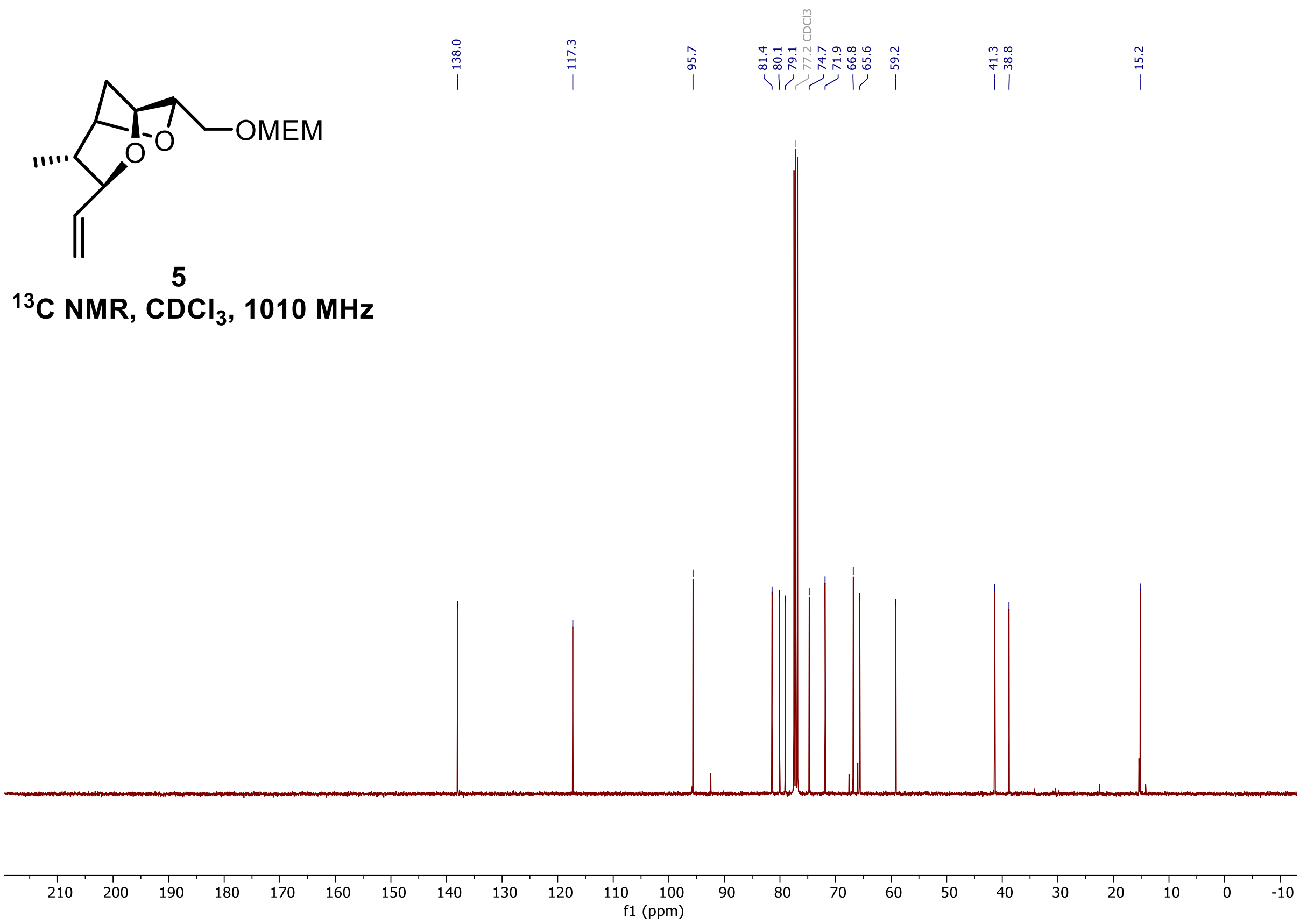

5

^{13}C NMR, $CDCl_3$, 1010 MHz

BCO-THP fragment 51

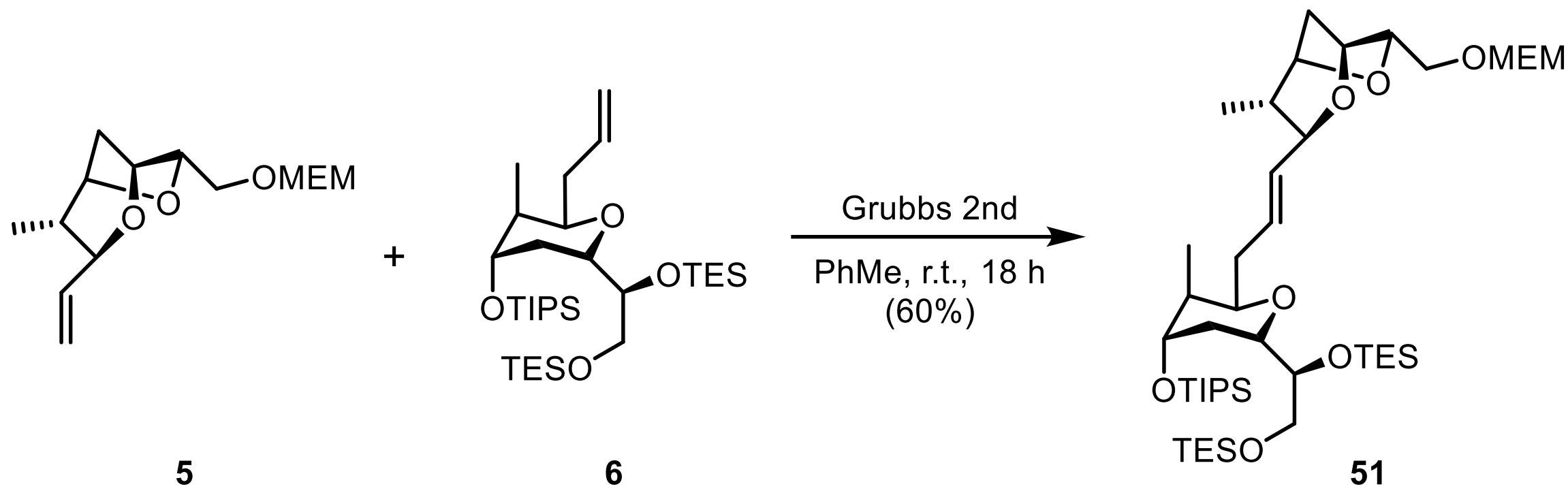

5 6 51

To a solution of bicyclooctane **5** (1.64 g, 6.02 mmol, 1.7 eq.) in 15 mL degassed, dry toluene was added a solution of tetrahydropyran **6** (2.13 g, 3.54 mmol, 1.0 eq.) in 15 mL dry toluene dropwise over 6 h via syringe pump at r.t. Simultaneously, a solution of Grubbs 2nd generation catalyst (452 mg, 0.53 mmol, 15 mol%) in 4 mL degassed, dry toluene was added via syringe pump over 6 h. Upon completion of the addition, the reaction was stirred for 12 h at r.t., the flask was opened to air and stirred for an additional 1 h. The reaction mixture was concentrated under reduced pressure and the crude product was purified by flash chromatography (10-20% EtOAc in hexane) to yield BCO-THP fragment **51** (1.81 g, 2.14 mmol, 60%) as a colorless oil.

$\mathbf{R_f}$ = 0.65 (hexane/Et_2O, 50:1), vanillin, UV (-)

^{1}H NMR (600 MHz, $CDCl_3$) δ [ppm] = 5.61 (ddd, *J* = 14.5, 9.0, 5.0 Hz, 1H), 5.42 (dd, *J* = 15.6, 7.4 Hz, 1H), 4.78 (s, 2H), 4.35 (q, *J* = 2.1 Hz, 1H), 4.25 (d, *J* = 6.6 Hz, 1H), 4.09 – 4.03 (m, 1H), 3.98 – 3.86 (m, 5H), 3.84 – 3.77 (m, 2H), 3.74 – 3.71 (m, 2H), 3.71 – 3.67 (m, 1H), 3.62 – 3.53 (m, 3H), 3.47 (dd, *J* = 10.2, 6.0 Hz, 1H), 3.39 (s, 3H), 2.29 (dddd, *J* = 12.0, 7.0, 5.1, 1.7 Hz, 1H), 2.08 – 1.97 (m, 3H), 1.85 (dd, *J* = 11.7, 1.6 Hz, 1H), 1.69 (ddd, *J* = 14.0, 11.7, 2.6 Hz, 1H), 1.55 (qd, *J* = 6.4, 4.2 Hz, 1H), 1.52 – 1.45 (m, 1H), 1.42 – 1.34 (m, 1H), 1.10 – 0.99 (m, 18H), 0.94 (td, *J* = 8.0, 1.4 Hz, 21H), 0.83 (dd, *J* = 7.0, 2.4 Hz, 6H), 0.66 – 0.53 (m, 12H).

^{13}C NMR (151 MHz, $CDCl_3$) δ [ppm] = 131.6, 131.1, 95.7, 81.5, 79.9, 79.1, 76.1, 74.8, 73.6, 73.0, 71.9, 71.3, 66.9, 65.8, 64.6, 59.2, 41.7, 38.9, 38.4, 35.8, 29.0, 18.3, 15.3, 12.4, 10.7, 7.1, 6.9, 5.2, 4.5.

IR (ATR) ṽ (cm^{-1}) = 2952 (m), 2940 (m), 2915 (m), 2873 (m), 1968 (w), 1460 (m), 1414 (w), 1379 (w), 1343 (w), 1315 (w), 1297 (w), 1285 (w), 1239 (w), 1199 (w), 1143 (m), 1115 (s), 1077 (s), 1049 (s), 1005 (s), 969 (s), 920 (w), 903 (w), 881 (w), 837 (w), 803 (w), 768 (w), 738 (s), 726 (s), 678 (s), 607 (w), 568 (w), 539 (w), 523 (w), 501 (w),468 (w), 444 (w), 428 (w).

HRMS (ESI) (*m/z*) calcd. for $[C_{44}H_{88}O_9Si_3Na]^+$ = $[M+Na]^+$: 867.5634; found: 867.5633.

Specific Rotation $[\alpha]_{589}^{20}$= –3.6° (c = 0.61, $CHCl_3$).

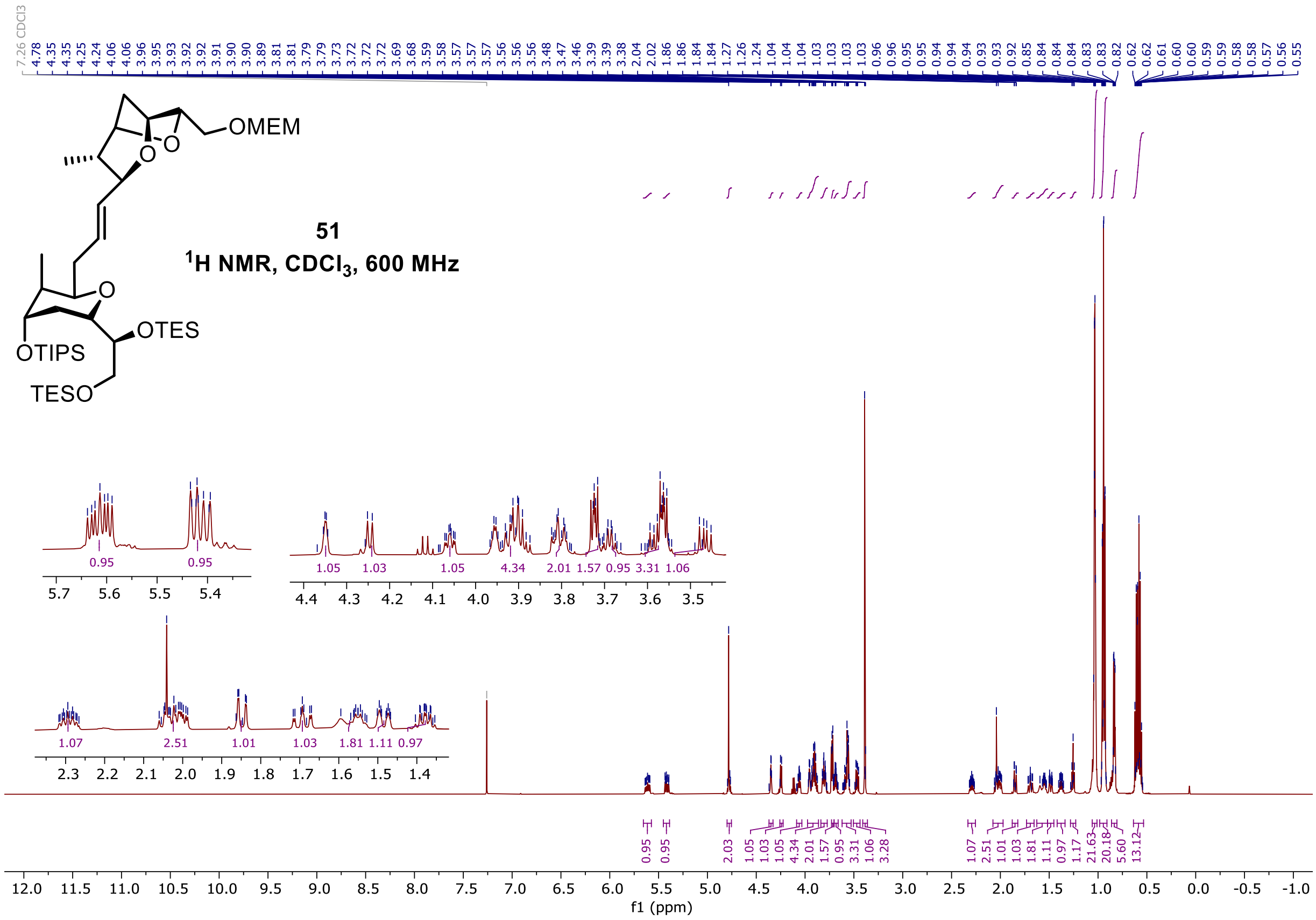

51

^{1}H NMR, CDCl$_3$, 600 MHz

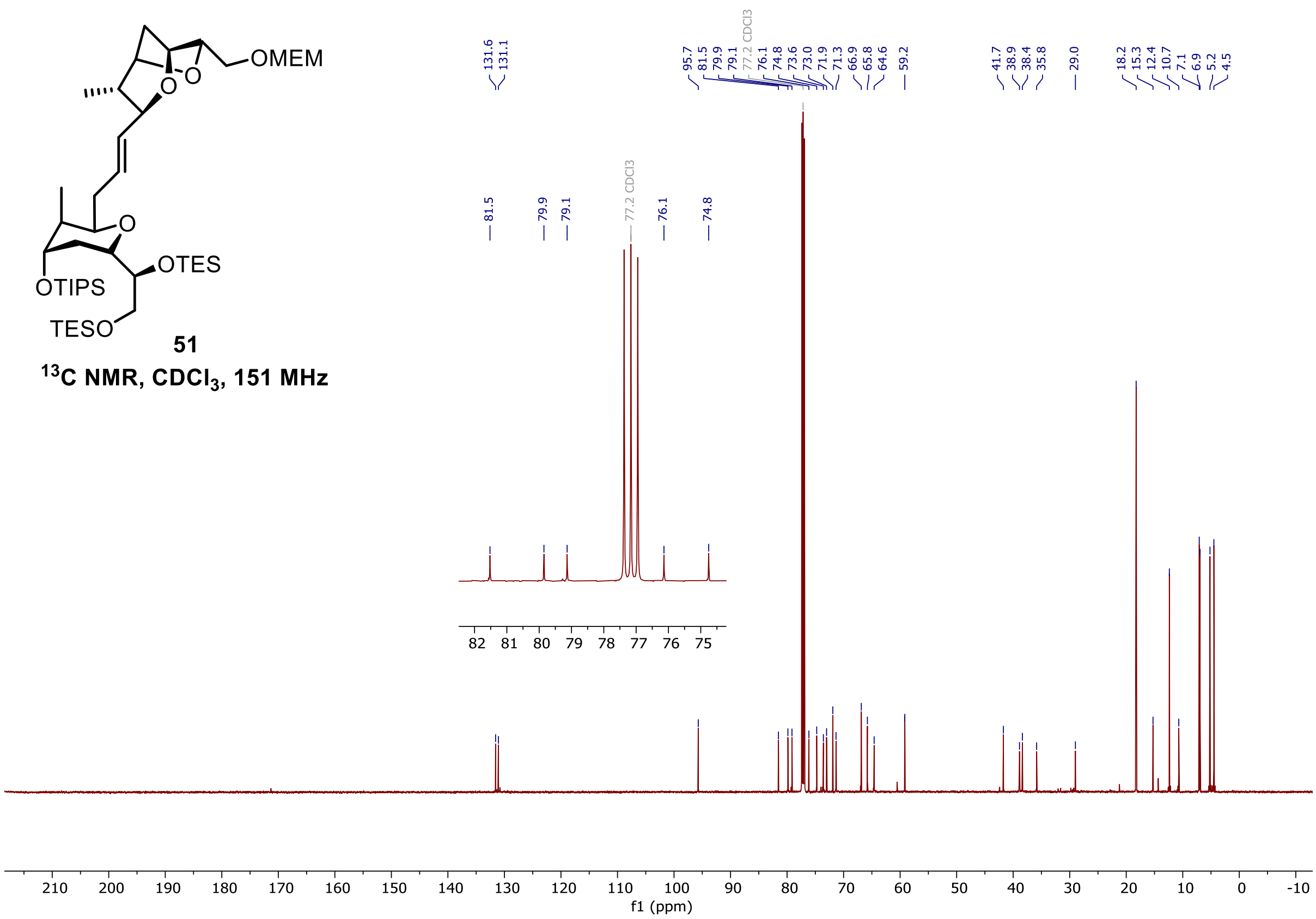

51

^{13}C NMR, CDCl$_3$, 151 MHz

Aldehyde 52

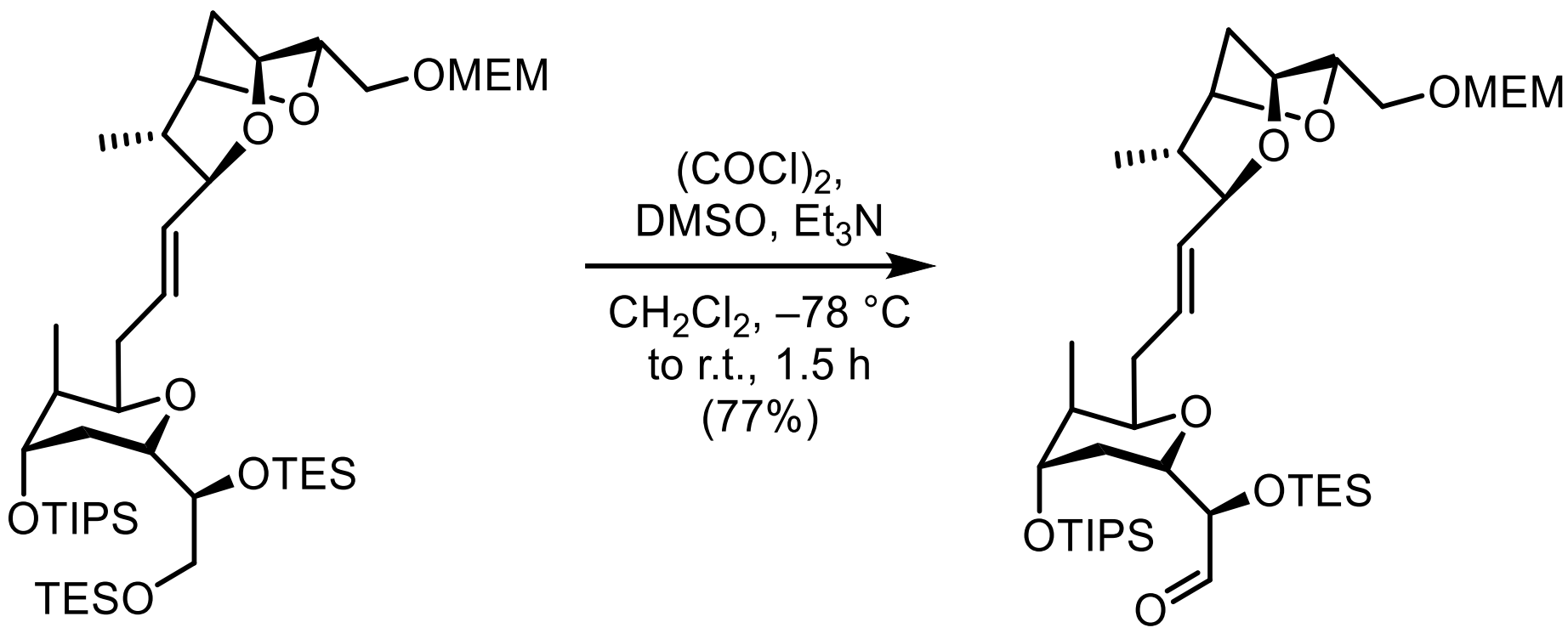

51 **52**

In a 50 mL three-neck flask, oxalyl chloride (0.47 mL, 5.5 mmol, 5.0 eq.) was dissolved in 4 mL dry CH_2Cl_2 and the solution was cooled to –78 °C. DMSO (0.71 mL, 10.0 mmol, 9.4 eq.), dissolved in 4 mL dry CH_2Cl_2, was slowly added, and the mixture was stirred for 15 min at –78 °C. Next, olefin **51** (900 mg, 1.06 mmol, 1.0 eq.), dissolved in 4.5 mL dry CH_2Cl_2 was slowly added to the reaction mixture. The mixture was stirred for 15 min at –78 °C, followed by 1 h at –45 °C. Upon completion of the reaction, the mixture was cooled again to –78 °C and Et_3N (3.0 mL, 21.5 mmol, 20 eq.) was slowly added. The mixture was slowly warmed to r.t. and carefully quenched with sat. $NaHCO_3$ solution. The phases were separated and the aqueous phase was extracted with CH_2Cl_2. The combined organic layer was washed with sat. NaCl solution, dried over $MgSO_4$, and concentrated under reduced pressure. The crude material was purified by flash chromatography (pentane/Et_2O, 2:3) to afford aldehyde **52** (600 mg, 0.82 mmol, 77%) as a colorless oil.

$\mathbf{R_f}$ = 0.67 (Et_2O), vanillin: blue, UV (-).

^{1}H NMR (400 MHz, $CDCl_3$) δ [ppm] = 9.62 (d, *J* = 1.6 Hz, 1H), 5.59 (ddd, *J* = 14.1, 8.5, 5.2 Hz, 1H), 5.42 (dd, *J* = 15.3, 7.7 Hz, 1H), 4.78 (s, 2H), 4.35 (q, *J* = 2.1 Hz, 1H), 4.25 (d, *J* = 6.4 Hz, 1H), 4.07 (ddd, *J* = 11.9, 5.5, 2.1 Hz, 2H), 4.00 – 3.86 (m, 5H), 3.81 (t, *J* = 7.6 Hz, 1H), 3.75 – 3.67 (m, 2H), 3.60 – 3.52 (m, 2H), 3.39 (s, 3H), 2.29 (dt, *J* = 11.7, 5.5 Hz, 1H), 2.09 – 1.96 (m, 2H), 1.89 – 1.73 (m, 2H), 1.68 – 1.52 (m, 2H), 1.46 – 1.32 (m, 2H), 1.03 (s, 21H), 0.95 (t, *J* = 7.9 Hz, 9H), 0.84 (dd, *J* = 7.0, 1.4 Hz, 6H), 0.62 (q, *J* = 7.9 Hz, 6H).

^{13}C NMR (101 MHz, $CDCl_3$) δ [ppm] = 203.1, 131.9, 130.4, 95.7, 81.5, 80.2, 79.7, 79.1, 74.8, 74.1, 73.9, 71.9, 70.9, 66.9, 65.8, 59.2, 41.7, 38.9, 38.3, 35.6, 30.1, 18.2, 15.3, 12.3, 10.7, 6.8, 4.9.

IR (ATR) ṽ (cm^{-1}) = 2942 (m), 2872 (m), 1737 (m), 1461 (m), 1242 (w), 1101 (m), 1051 (s), 1008 (m), 971 (m), 881 (m), 825 (w), 739 (m), 680 (m), 607 (w).

HRMS (ESI) (*m/z*) calcd. for $[C_{38}H_{72}O_9Si_2NH_4]^+$ = $[M+NH_4]^+$: 746.5059, found: 746.5057.

Specific Rotation $[\alpha]_{589}^{20}$= –6.7° (c = 1.75, $CHCl_3$).

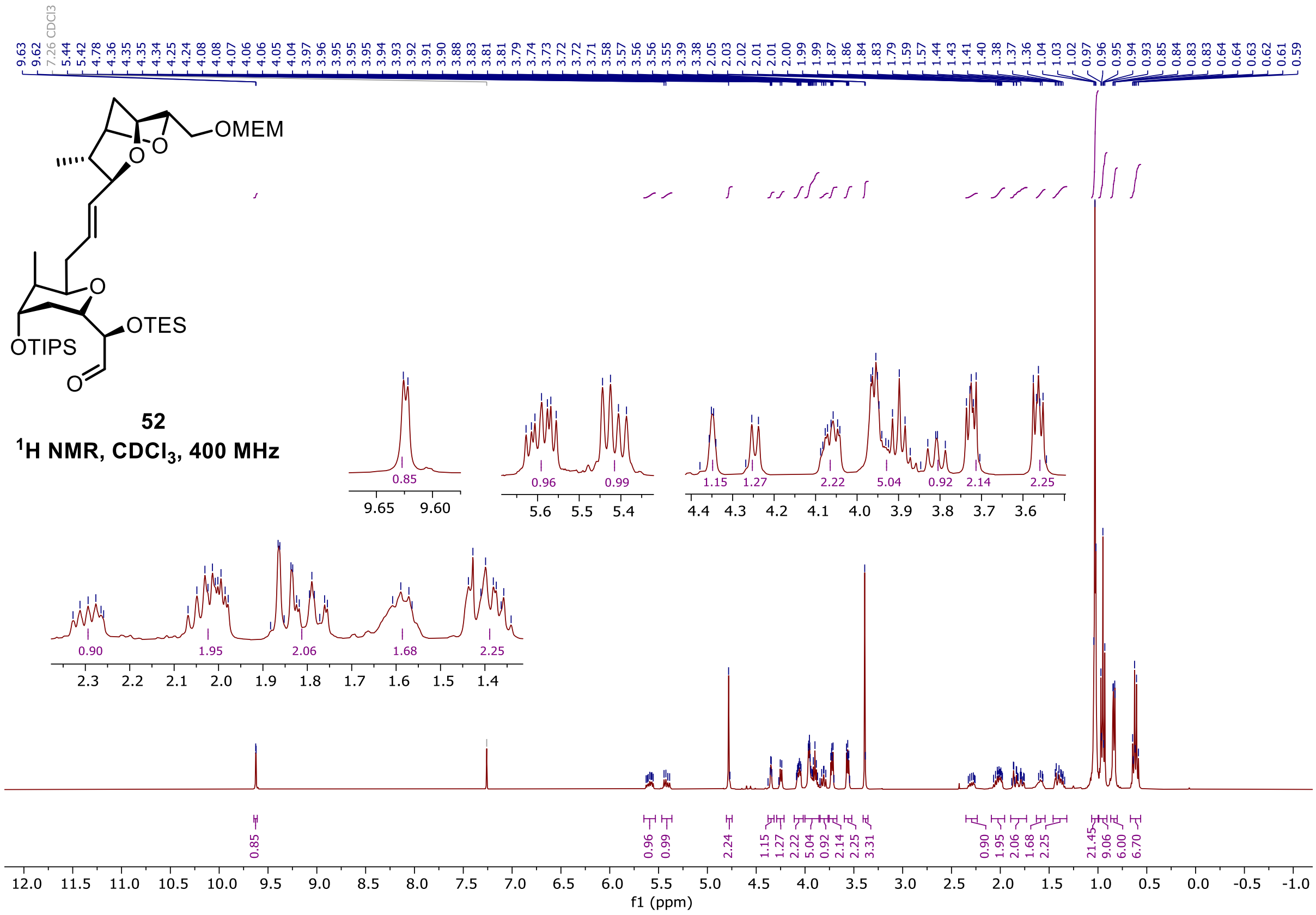

52
^{1}H NMR, $CDCl_3$, 400 MHz

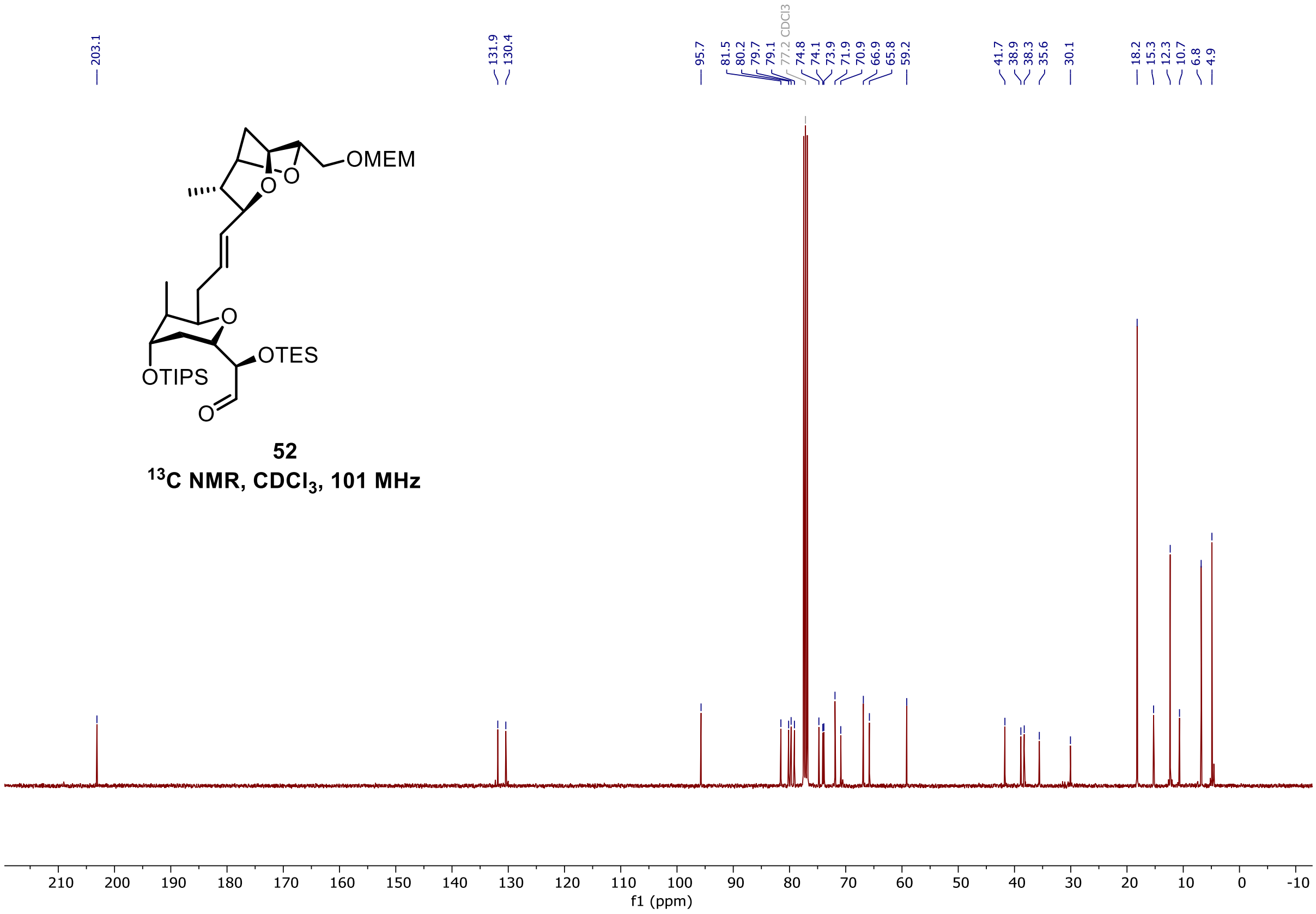

52
^{13}C NMR, $CDCl_3$, 101 MHz

(*E*)-1-iodohexa-1,5-diene (53b)

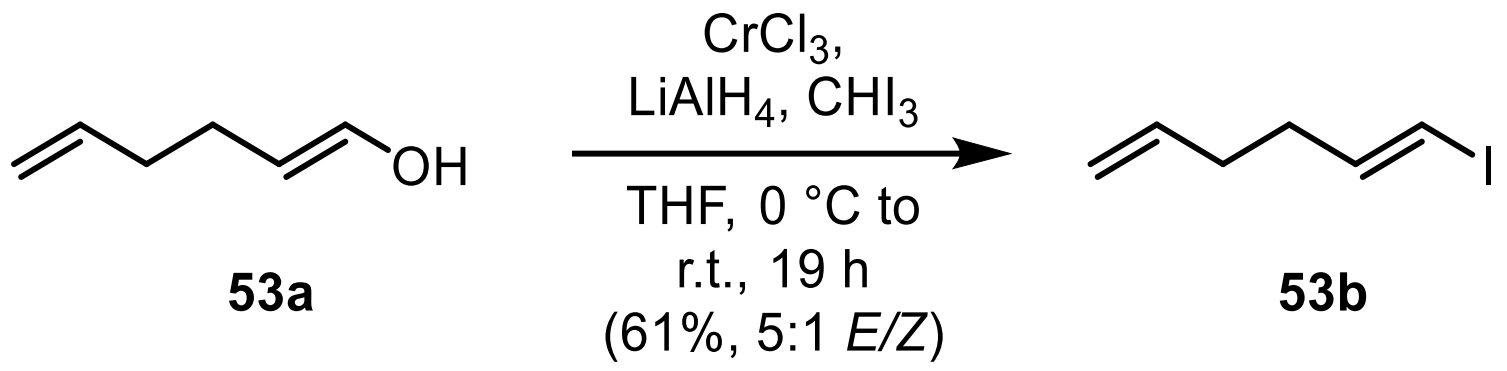

A round-bottom flask was charged with $CrCl_3$ (25.0 g, 157.9 mmol, 7.1 eq.), suspended in 200 mL dry THF and cooled to 0 °C. $LiAlH_4$ (3.0 g, 79.0 mmol, 3.5 eq.) was carefully added portionwise over a period of 30 min. The resulting black suspension was stirred for 1 h at 0 °C, before addition of a solution of 5-hexen-1-ol (**53a**) (2.2 mL, 22.3 mmol, 1.0 eq.) and iodoform (21.0 g, 53.3 mmol, 2.4 eq.) in 100 mL dry 1,4-dioxane. The mixture was stirred for 4 h at 0 °C and 14 h at r.t. before dilution with pentane (200 mL). The precipitate was filtered off through a short silica plug and wash with pentane. The collected organic phase was subsequently washed with sat. $Na_2S_2O_3$ solution, water and sat. NaCl solution, dried over Na_2SO_4 and concentrated in vacuo carefully (volatile product). The crude material was purified by flash chromatography (pentane) to yield iodide **53b** (2.85 g, 13.7 mmol, 61%, *E/Z* 5:1) as a red liquid.

Note: Due to the volatility of the product, the solvents were distilled off while using a Vigreux column.

R_f = 0.80 (pentane), $KMnO_4$, UV (+).

^{1}H NMR (400 MHz, $CDCl_3$) δ [ppm] = 6.58 – 6.45 (m, 1H), 6.02 (d, *J* = 1.3 Hz, 1H), 5.89 – 5.71 (m, 1H), 5.08 – 4.96 (m, 2H), 2.18 – 2.06 (m, 4H).

^{13}C NMR (101 MHz, $CDCl_3$) δ [ppm] = 145.8, 137.3, 115.6, 115.5, 75.1, 35.5, 32.6.

IR (ATR) ṽ (cm^{-1}) = 2980 (w), 2923 (w), 1640 (w), 1605 (w), 1438 (w), 1237 (w), 1216 (w), 1178 (w), 991 (m), 938 (m), 912 (s), 692 (w), 663 (w), 628 (w), 419 (w).

HRMS (ESI) (*m/z*) calcd. for $[C_6H_9IH]^+$ = $[M+H]^+$:208.9827, found: 208.9837.

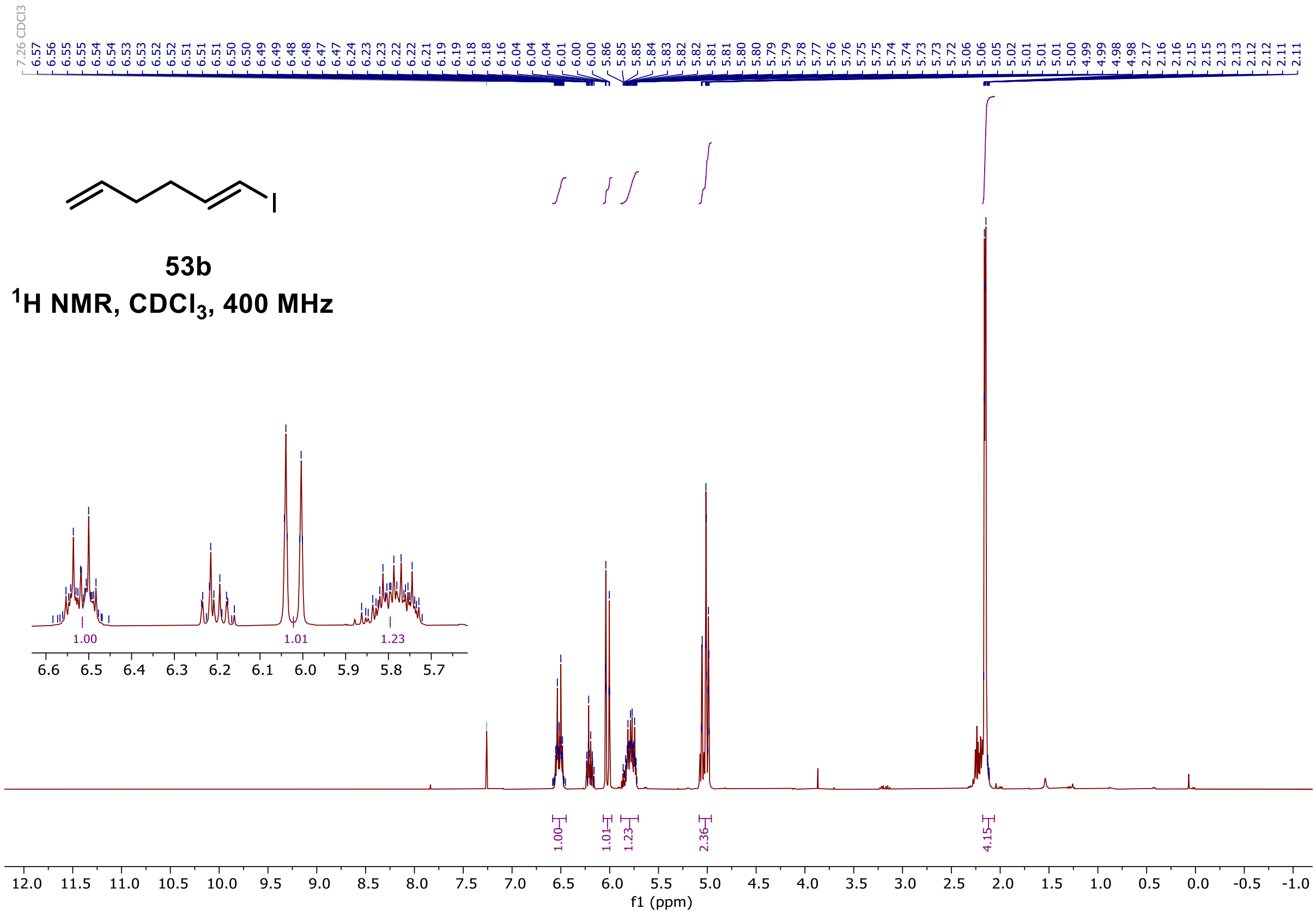
53b
1H NMR, CDCl3, 400 MHz
1.00
1.01
1.23
2.36
4.15
f1 (ppm)

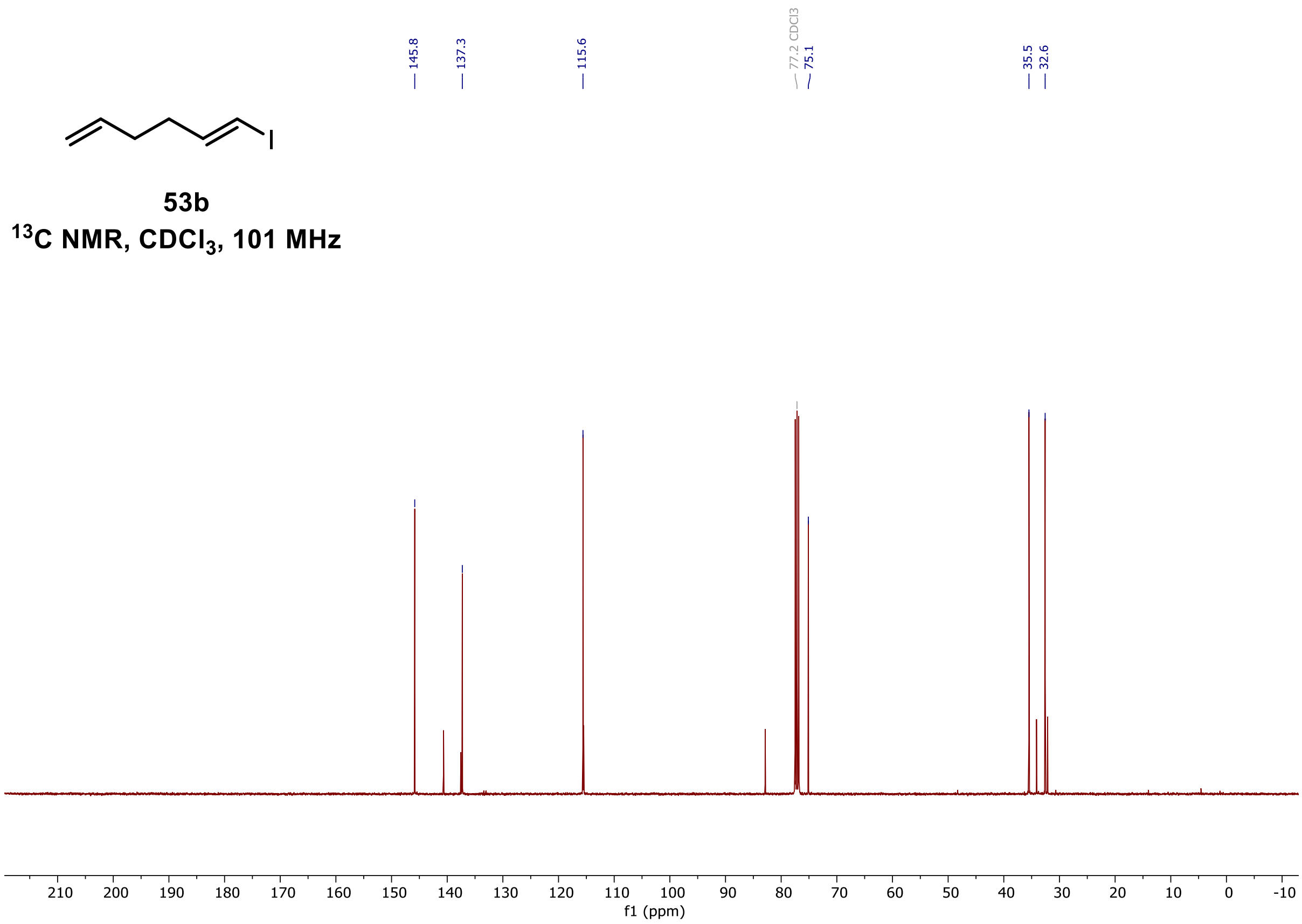
145.8
137.3
115.6
77.2 CDCl3
75.1
35.5
32.6
53b
13C NMR, CDCl3, 101 MHz
f1 (ppm)

Ketal 55

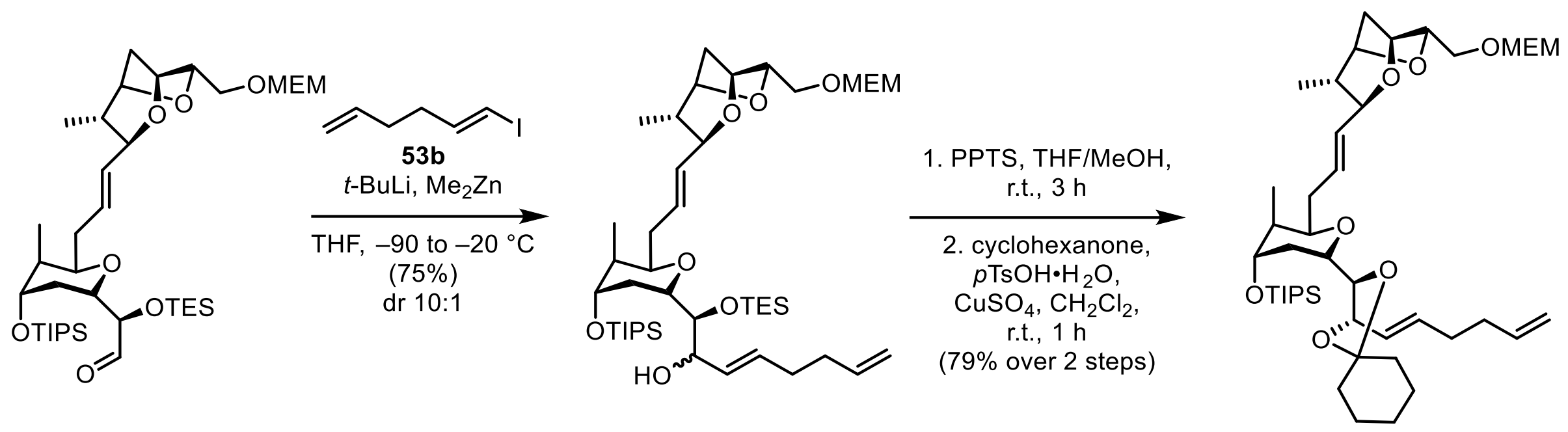

52 **54** **55**

A three-neck flask was charged with vinyl iodide **53b** (740 mg, 3.56 mmol, 3.0 eq.), dissolved in 20 mL dry THF and cooled to –78 °C. To this solution was slowly added *t*-BuLi (1.6M in pentane, 4.4 mL, 7.04 mmol, 6.0 eq.) and the mixture was stirred for 1 h at –78 °C. A dimethyl zinc solution (1M in *n*-heptane, 4.7 mL, 4.70 mmol, 4.0 eq.) was added and the reaction was stirred for additional 30 min at –78 °C before cooling it to –90 °C. Next, a solution of aldehyde **52** (860 mg, 1.18 mmol, 1.0 eq.), dissolved in 6 mL dry THF, was slowly added and the mixture was stirred for 1 h at –90 °C, then warmed up to –20 °C. At this temperature, the reaction was quenched carefully with sat. $NaHCO_3$ solution and warmed up to r.t. After further dilution with sat. $NaHCO_3$ solution (50 mL), the mixture was extracted with Et_2O and the combined organic layer was washed with sat. NaCl solution and dried over $MgSO_4$. After removal of the solvent, the crude material was purified by flash chromatography (pentane/Et_2O, 1:1) to obtain the crude triene **54** (717 mg, 0.88 mmol, 75%, dr 10:1, R_f = 0.67 (Et_2O), vanillin: black, UV (-)) as a highly viscous oil. The undesired isomer was found to be only separated after the next steps, therefore the mixture was used for the following reaction.
In a round-bottom flask, the diastereomeric mixture of compound **54** (717 mg, 0.88 mmol, 1.0 eq.) was dissolved in 50 mL dry MeOH/THF (4:1, v/v). PPTS (200 mg, 0.80 mmol, 0.9 eq.) was added and the resulting mixture was stirred for 3 h at r.t. After completion, the mixture was quenched with water, the layers were separated and the aqueous layer was extracted with CH_2Cl_2. The combined organic layer was washed with sat. NaCl solution and dried over $MgSO_4$. The solvent was subsequently removed under reduced pressure and the crude diol **55a** (800 mg) was used for the next step without further purification.
The crude diol **55a** (800 mg) was dissolved in 40 mL dry CH_2Cl_2, before addition of cyclohexanone (500 µL, 4.83 mmol, 5.5 eq.), dry $CuSO_4$ (1.0 g, 6.27 mmol, 7.1 eq.) and *p*TsOH•H_2O (200 mg, 1.05 mmol, 1.2 eq.). The reaction was stirred 1 h at r.t. before quenching with water, separating the layers and extracting the aqueous layer with CH_2Cl_2. The combined organic layer was washed with sat. NaCl solution, dried over $MgSO_4$ and the solvent was removed in vacuo. Flash chromatography of the crude material (pentane/Et_2O, 2:3) afforded ketal **55** (540 mg, 0.70 mmol, 79% over 2 steps) as a colorless, highly viscous oil.

$\mathbf{R_f}$ = 0.21 (pentane/Et_2O, 1:1), vanillin: blue, UV (-).

^{1}H NMR (600 MHz, $CDCl_3$) δ [ppm] = 5.88 – 5.73 (m, 2H), 5.59 – 5.49 (m, 2H), 5.46 – 5.38 (m, 1H), 5.05 – 4.92 (m, 2H), 4.77 (d, *J* = 5.1 Hz, 2H), 4.61 (ddd, *J* = 7.0, 5.8, 1.0 Hz, 1H), 4.37 – 4.30 (m, 1H), 4.27 – 4.21 (m, 1H), 4.05 (ddd, *J* = 6.9, 5.3, 2.2 Hz, 1H), 3.98 – 3.92 (m, 1H), 3.92 – 3.78 (m, 5H), 3.73 – 3.69 (m, 2H), 3.57 – 3.53 (m, 2H), 3.38 (s, 4H), 2.29 – 2.22 (m, 1H), 2.20 – 2.06 (m, 5H), 1.99 (ddd, *J* = 11.6, 6.6, 2.7 Hz, 1H), 1.87 – 1.80 (m, 1H), 1.75 – 1.68

(m, 1H), 1.65 – 1.50 (m, 5H), 1.40 – 1.30 (m, 4H), 1.11 – 0.98 (m, 21H), 0.83 (dd, *J* = 7.0, 5.5 Hz, 6H).

^{13}**C NMR** (151 MHz, $CDCl_3$) δ [ppm] = 138.3, 133.4, 132.2, 129.9, 126.3, 114.9, 108.8, 95.7, 81.5, 79.6, 79.5, 79.1, 78.6, 74.7, 73.1, 71.9, 71.3, 71.0, 66.9, 65.7, 59.1, 41.7, 38.8, 37.9, 37.8, 35.9, 35.2, 33.5, 32.0, 31.6, 25.3, 24.2, 23.9, 18.2, 15.3, 12.4, 10.6.

IR (ATR) ṽ (cm^{-1}) = 2933 (s), 2865 (s), 1461 (m), 1366 (m), 1284 (w), 1096 (s), 1046 (s), 970 (s), 907 (m), 881 (m), 679 (m), 657 (m), 541 (w), 501 (w).

HRMS (ESI) (*m/z*) calcd. for $[C_{44}H_{76}O_9SiNH_4]^+$ = $[M+NH_4]^+$: 794.5602, found: 794.5594.

Specific Rotation $[\alpha]_{589}^{20}$= –0.1° (c = 2.0, $CHCl_3$).

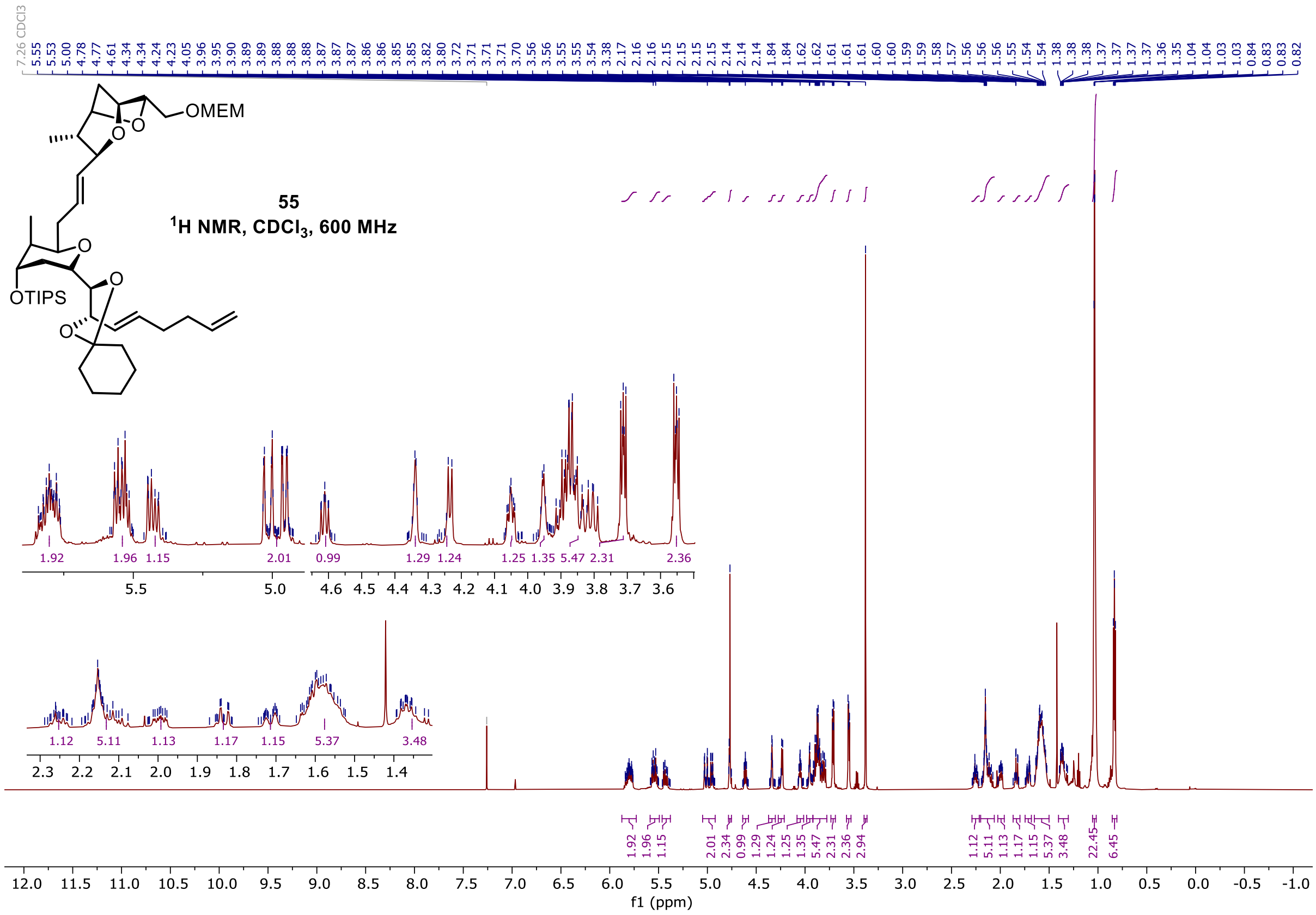

55

^{1}H NMR, $CDCl_3$, 600 MHz

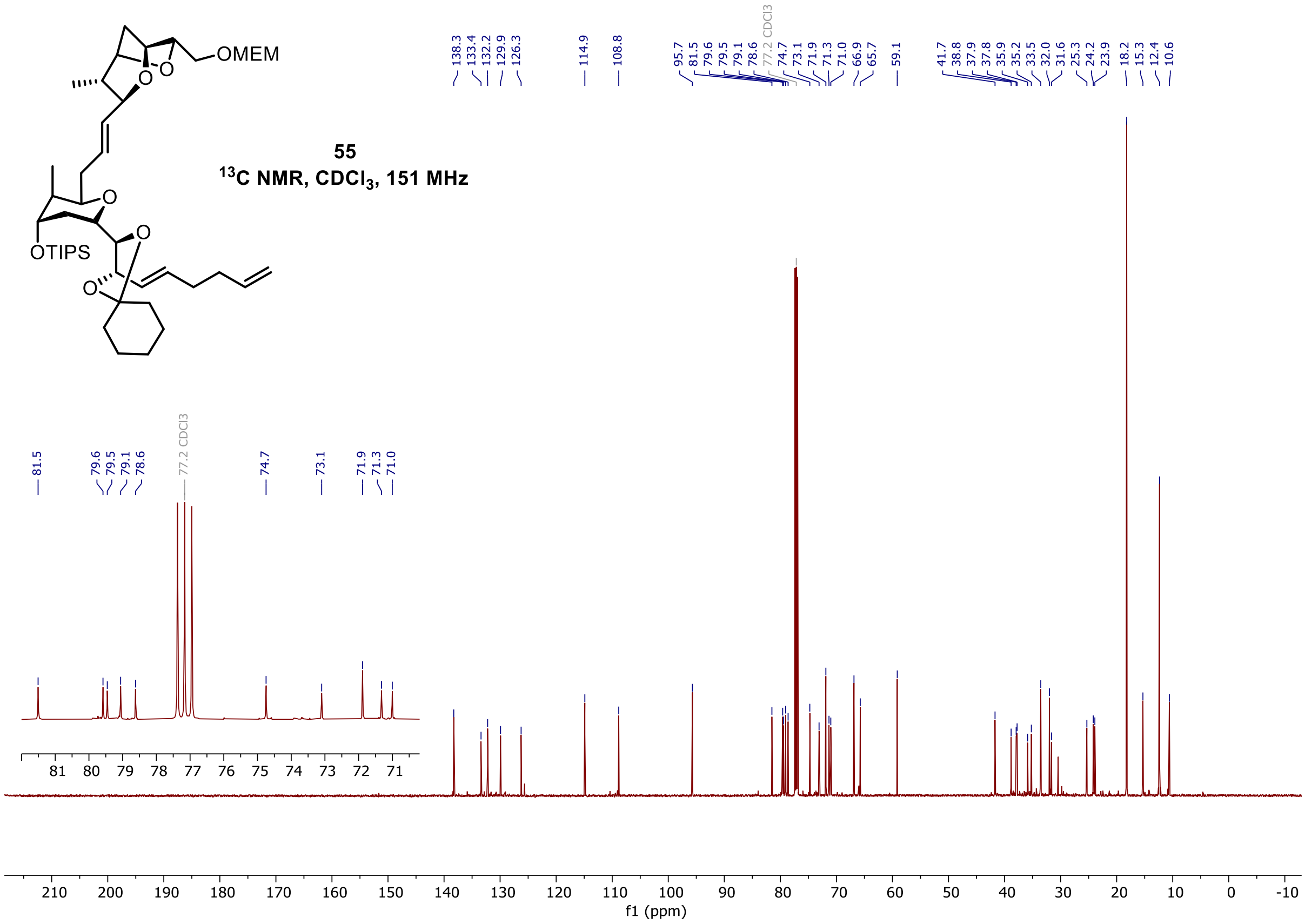

55

^{13}C NMR, $CDCl_3$, 151 MHz

Alcohol 56

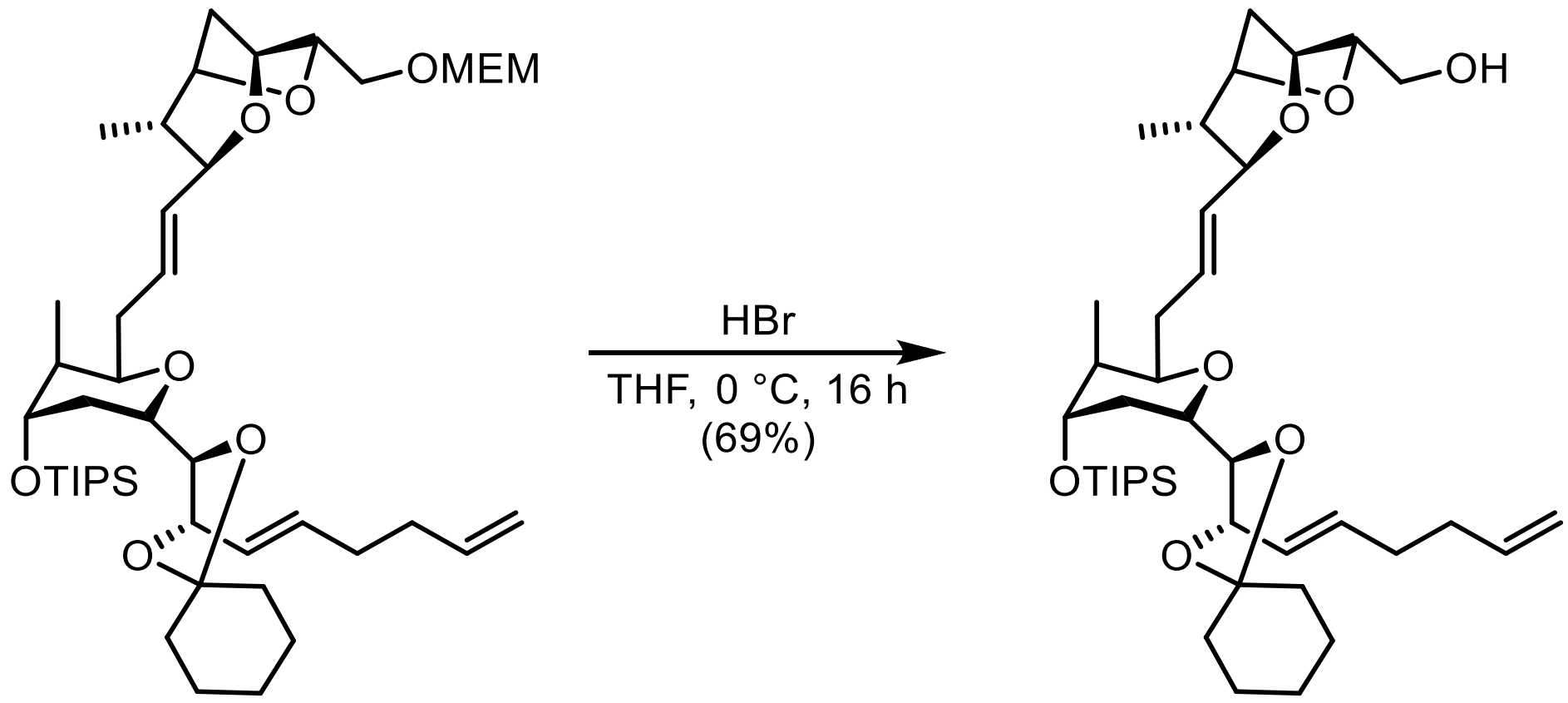

55 **56**

In a round-bottom flask, ketal **55** (130 mg, 0.167 mmol, 1.0 eq.) was dissolved in 5 mL dry THF and cooled to 0 °C. Next, HBr (33% in AcOH, 0.2 mL, 7.3 eq.) was added slowly and the resulting mixture was stirred for 16 h at 0 °C. After completion, the reaction was neutralized with sat. $NaHCO_3$ solution, the aqueous phase was extracted with Et_2O and the combined organic layer was washed with sat. NaCl solution. After drying over $MgSO_4$, the solvent was removed in vacuo and the crude material was purified by flash chromatography (pentane/Et_2O, 1:2) to yield alcohol **56** (80 mg, 0.116 mmol, 69%) as a colorless oil.

$\mathbf{R_f}$ = 0.30 (Et_2O), vanillin: violet, UV (-).

^{1}H NMR (600 MHz, $CDCl_3$) δ [ppm] = 5.85 – 5.73 (m, 2H), 5.57 – 5.48 (m, 2H), 5.46 – 5.37 (m, 1H), 5.04 – 4.98 (m, 1H), 4.98 – 4.90 (m, 1H), 4.59 (ddd, *J* = 7.0, 5.8, 1.0 Hz, 1H), 4.36 – 4.32 (m, 1H), 4.23 (d, *J* = 6.6 Hz, 1H), 4.02 – 3.90 (m, 3H), 3.90 – 3.78 (m, 4H), 2.33 – 2.20 (m, 3H), 2.18 – 2.10 (m, 3H), 2.10 – 2.04 (m, 1H),1.99 (ddd, *J* = 11.6, 6.7, 2.7 Hz, 1H), 1.87 – 1.80 (m, 1H), 1.70 (ddt, *J* = 13.7, 3.4, 1.7 Hz, 1H), 1.64 – 1.48 (m, 4H), 1.40 – 1.31 (m, 1H), 1.32 – 1.23 (m, 1H), 1.09 – 0.96 (m, 21H), 0.83 (d, *J* = 1.8 Hz, 2H), 0.81 (d, *J* = 1.4 Hz, 3H).

^{13}C NMR (151 MHz, $CDCl_3$) δ [ppm] = 138.2, 133.3, 132.1, 130.2, 126.3, 114.8, 108.8, 83.1, 79.7, 79.4, 79.0, 78.5, 74.4, 73.1, 71.3, 71.0, 65.9, 60.9, 41.6, 38.9, 38.0, 37.7, 35.8, 35.2, 33.5, 32.0, 31.6, 25.3, 24.1, 23.8, 18.2, 15.3, 12.3, 10.6.

IR (ATR) ṽ (cm^{-1}) = 3442 (br), 2935 (s), 2865 (s), 1740 (m), 1461 (m), 1367 (m), 1281 (w), 1239 (m), 1143 (m), 1095 (s), 1046 (s), 970 (s), 907 (m), 881 (s), 819 (m), 712 (w), 679 (m), 544 (w), 499 (w).

HRMS (ESI) (*m/z*) calcd. for $[C_{40}H_{68}O_7SiNH_4]^+$ = $[M+NH_4]^+$: 706.5078, found: 706.5085.

Specific Rotation $[\alpha]_{589}^{20}$= +0.3° (c = 0.375, $CHCl_3$).

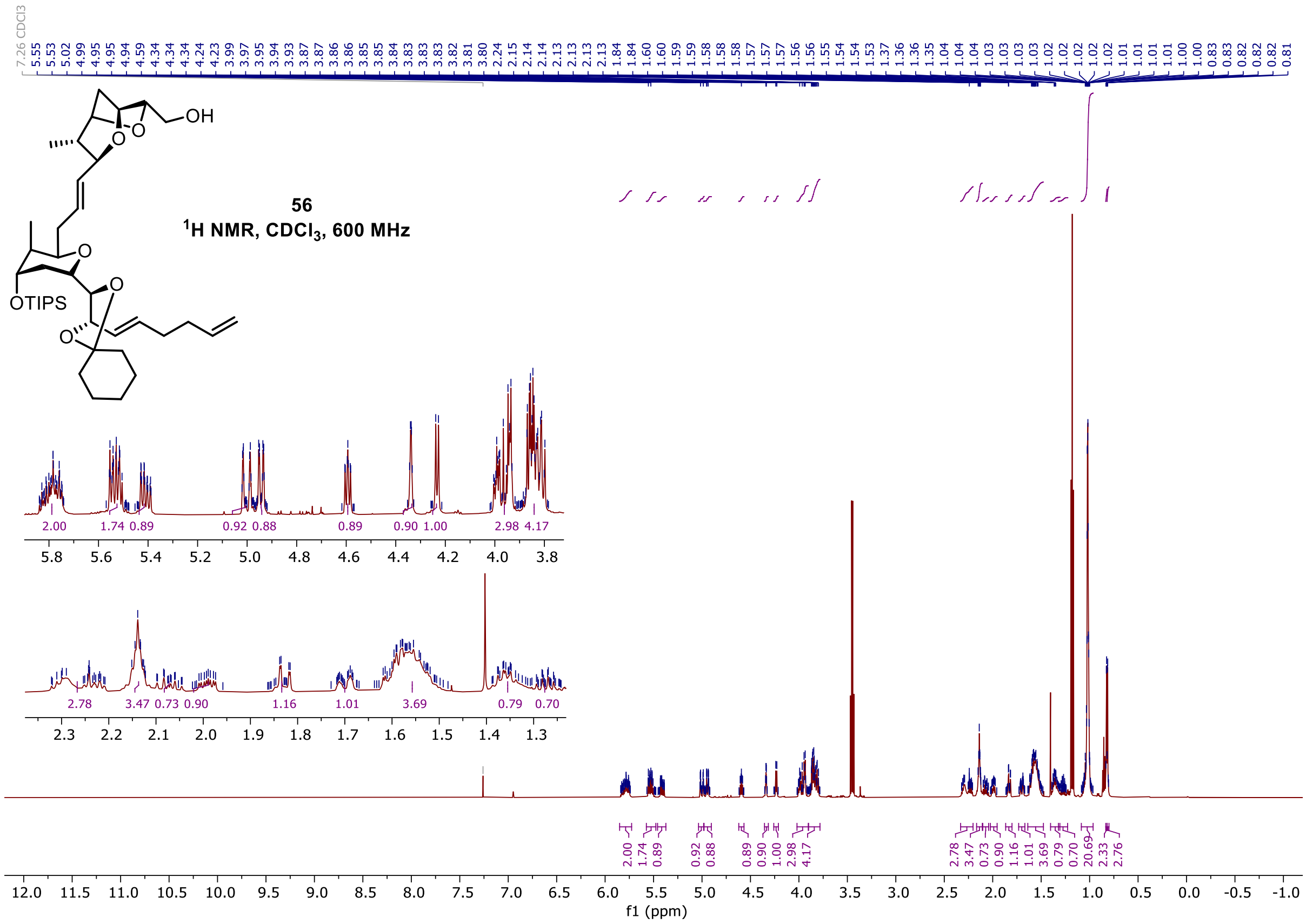
56
1H NMR, CDCl3, 600 MHz
f1 (ppm)

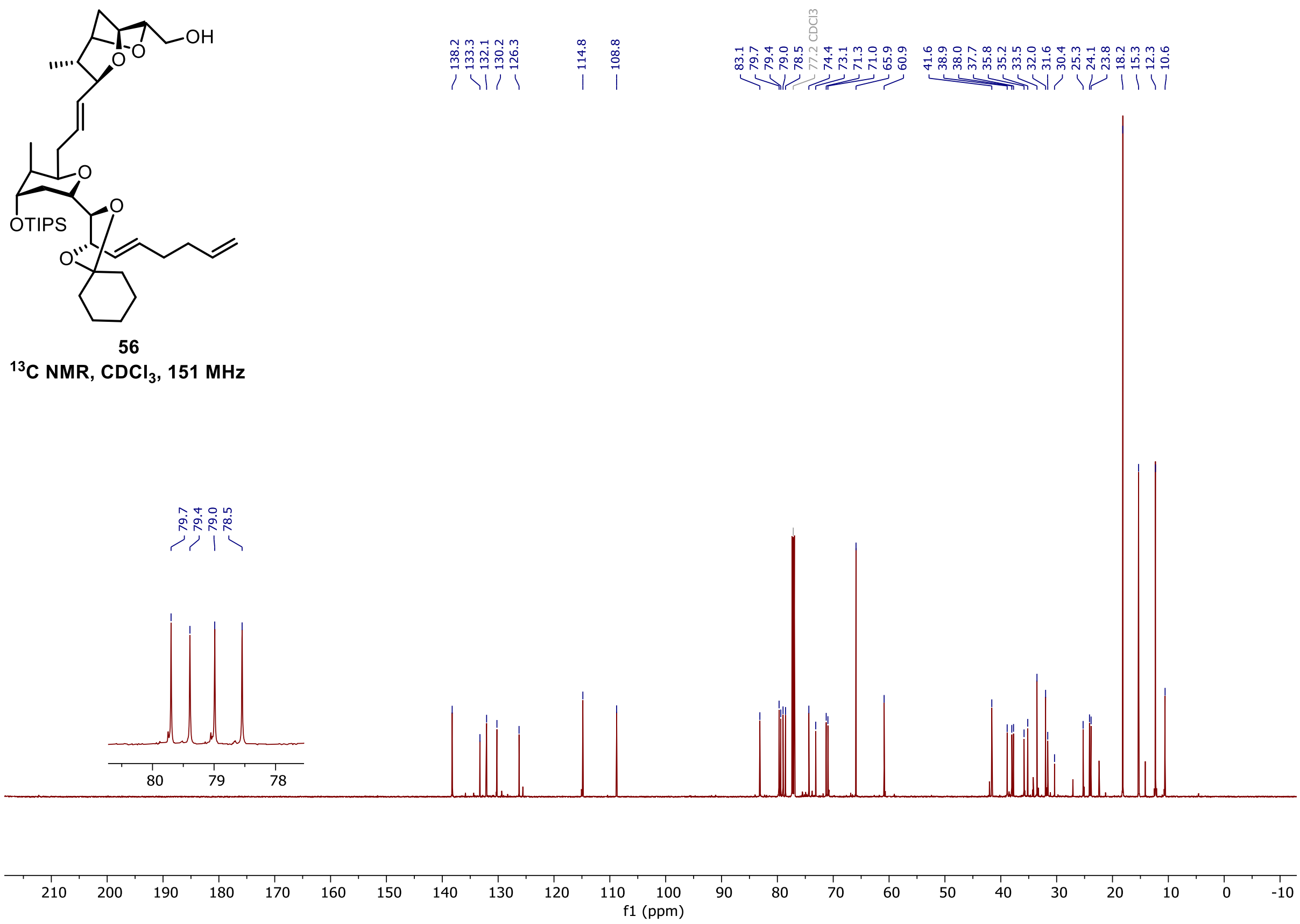
56
13C NMR, CDCl3, 151 MHz
f1 (ppm)

Alkyne 57

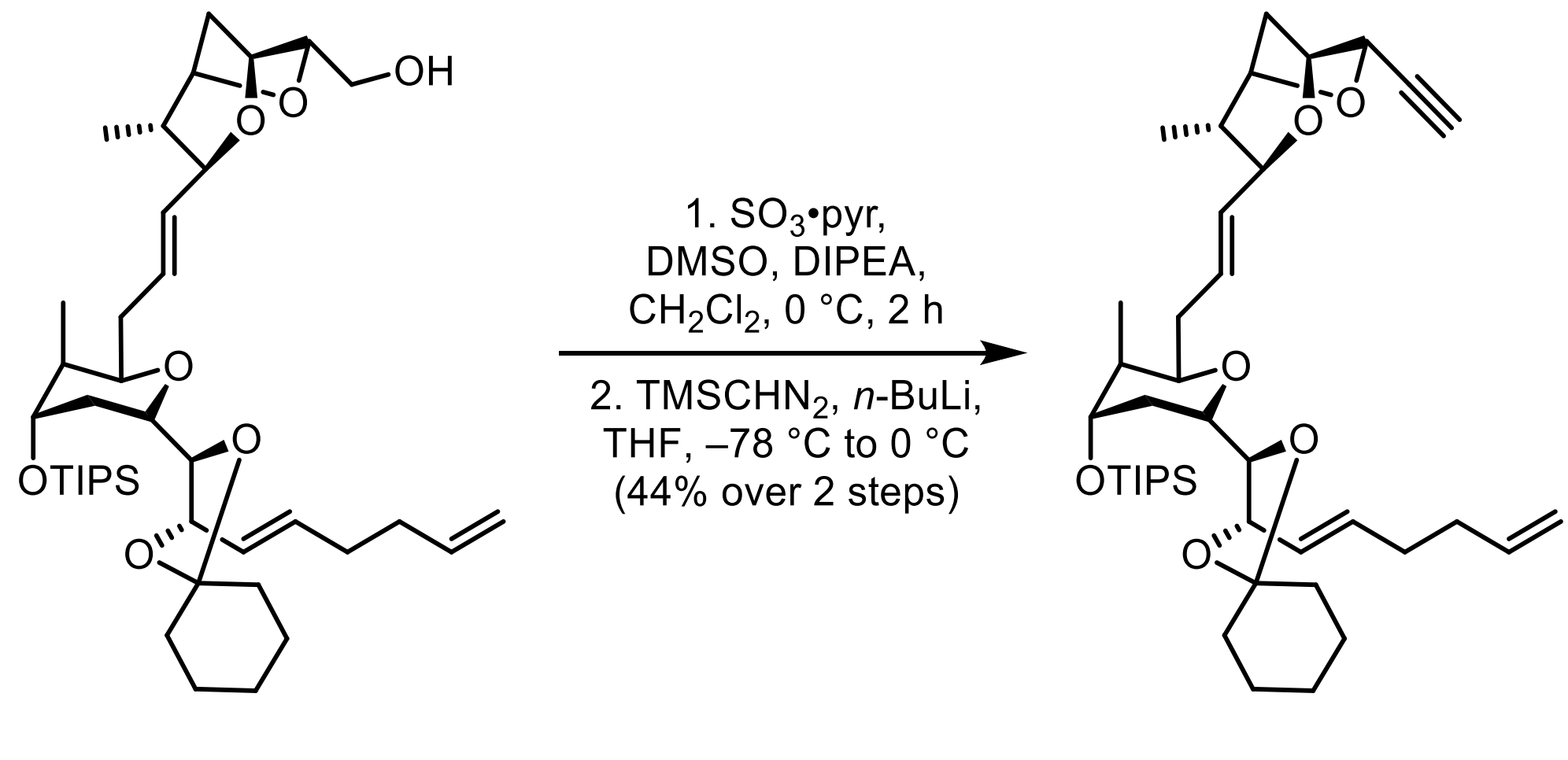

56 **57**

Alcohol **56** (30 mg, 44 µmol, 1.0 eq.) was dissolved in 1 mL dry CH_2Cl_2 and cooled to 0 °C. SO_3•pyridine (26 mg, 160 µmol, 3.8 eq.), together with DMSO (12 µL, 169 µmol, 3.9 eq.) and DIPEA (42 µL, 241 µmol, 5.5 eq.) were added and the resulting mixture was stirred for 2 h at 0 °C. After completion of the reaction, the mixture was diluted with CH_2Cl_2, the combined organic layer was washed subsequently with water, 5% $KHSO_4$ solution and sat. NaCl solution. The organic layer was dried over Na_2SO_4, the solvent was removed under reduced pressure to afford the crude aldehyde as a yellowish liquid, which was used without further purification. In a separate flask, $TMSCHN_2$ (22 µL, 132 µmol, 3.0 eq.) was dissolved in 1 mL dry THF and cooled to –78 °C. *n*-BuLi (2.5M in *n*-hexane, 34 µL, 85 mmol, 1.1 eq.) was added dropwise and the mixture was stirred for 30 min at –78 °C. Then, the crude aldehyde, dissolved in 0.5 mL dry THF, was added, the mixture was stirred for 30 min at –78 °C and then slowly warmed to 0 °C. The mixture was carefully quenched with sat. NH_4Cl solution, the aqueous layer was extracted with Et_2O and the combined organic layer was washed with sat. NaCl solution. After drying over Na_2SO_4 and removal of the solvent, the crude residue was purified by flash chromatography (pentane/Et_2O, 2:1) to afford alkyne **57** (13 mg, 19 µmol, 44% over 2 steps) as a colorless oil.

R_f = 0.58 (pentane/Et_2O, 1:1), vanillin: grey, UV (-).

^{1}H NMR (600 MHz, $CDCl_3$) δ [ppm] = 5.88 – 5.75 (m, 2H), 5.66 – 5.59 (m, 1H), 5.55 (ddt, *J* = 15.2, 7.6, 1.3 Hz, 1H), 5.47 (ddq, *J* = 15.3, 7.8, 0.9 Hz, 1H), 5.02 (dq, *J* = 17.2, 1.6 Hz, 1H), 4.95 (ddd, *J* = 10.3, 2.0, 1.1 Hz, 1H), 4.64 – 4.57 (m, 2H), 4.41 (q, *J* = 2.3 Hz, 1H), 4.35 (dd, *J* = 9.6, 7.7 Hz, 1H), 4.25 (d, *J* = 5.5 Hz, 1H), 3.96 (q, *J* = 2.9 Hz, 1H), 3.91 – 3.81 (m, 3H), 2.59 (d, *J* = 2.4 Hz, 1H), 2.31 – 2.23 (m, 1H), 2.21 – 2.07 (m, 5H), 1.97 – 1.88 (m, 2H), 1.75 – 1.68 (m, 1H), 1.66 – 1.50 (m, 10H), 1.42 – 1.31 (m, 3H), 1.06 – 1.01 (m, 21H), 0.86 (d, *J* = 3.9 Hz, 3H), 0.84 (d, *J* = 4.3 Hz, 3H).

^{13}C NMR (151 MHz, $CDCl_3$) δ [ppm] = 138.3, 133.4, 131.4, 130.7, 126.3, 114.8, 108.9, 80.3, 79.5, 78.8, 78.7, 76.5, 76.1, 73.2, 71.3, 71.2, 71.0, 41.1, 38.6, 38.0, 37.8, 35.9, 35.2, 33.6, 32.0, 31.7, 25.3, 24.2, 23.9, 18.2, 15.1, 12.4, 10.6.

IR (ATR) ṽ (cm^{-1}) = 2933 (s), 2865 (s), 1461 (m), 1366 (m), 1282 (w), 1250 (w), 1143 (m), 1095 (s), 1067 (s), 1051 (s), 970 (s), 908 (s), 880 (s), 818 (m), 732 (m), 678 (s), 659 (s), 500 (w).

HRMS (ESI) (*m/z*) calcd. for $[C_{41}H_{66}O_6SiH]^+$ = $[M+H]^+$: 683.4707, found: 683.4718.

Specific Rotation $[\alpha]_{589}^{20}$ = –9.0° (c = 0.7, $CHCl_3$).

57

^{1}H NMR, CDCl$_3$, 600 MHz

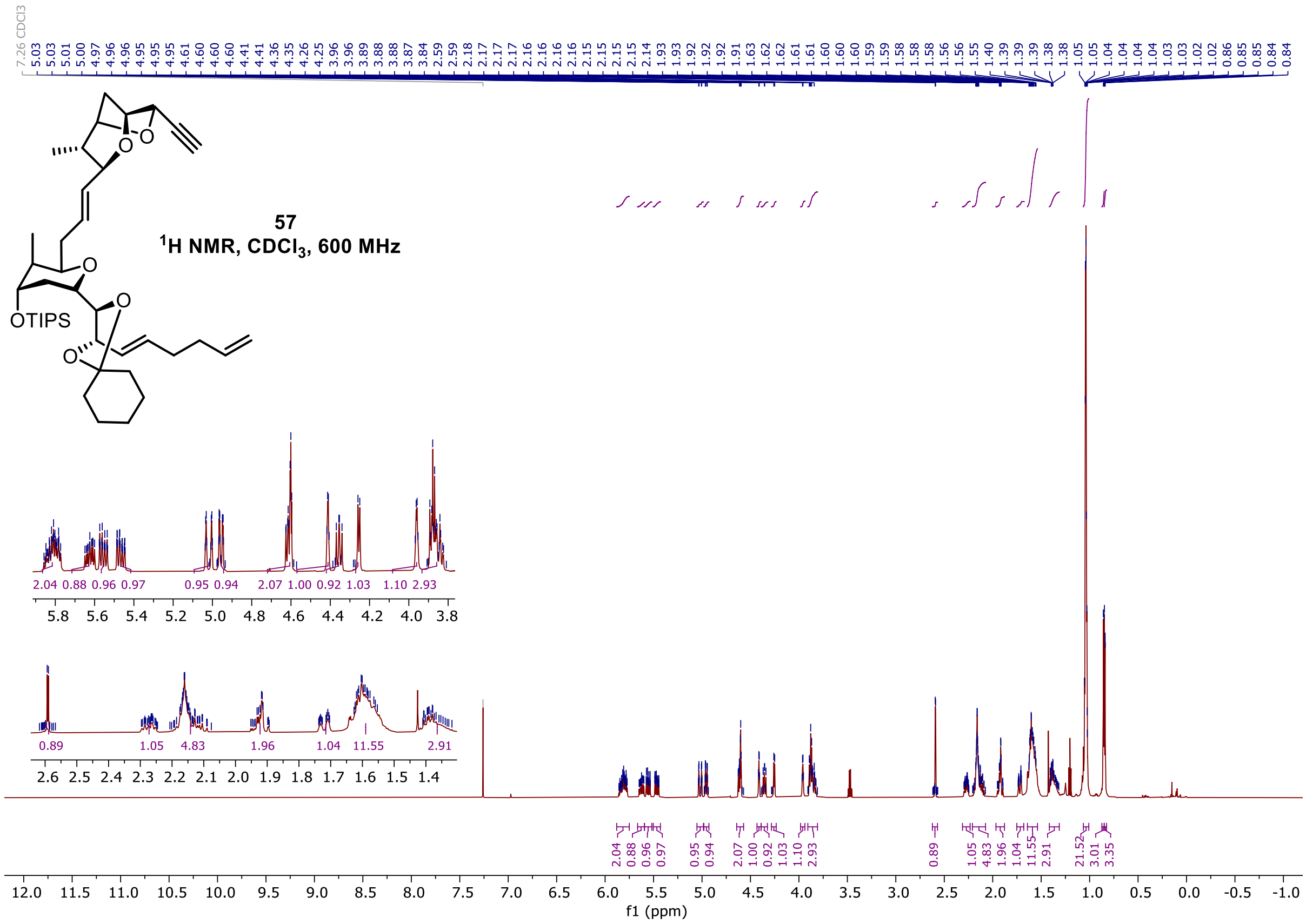

57

^{13}C NMR, CDCl$_3$, 151 MHz

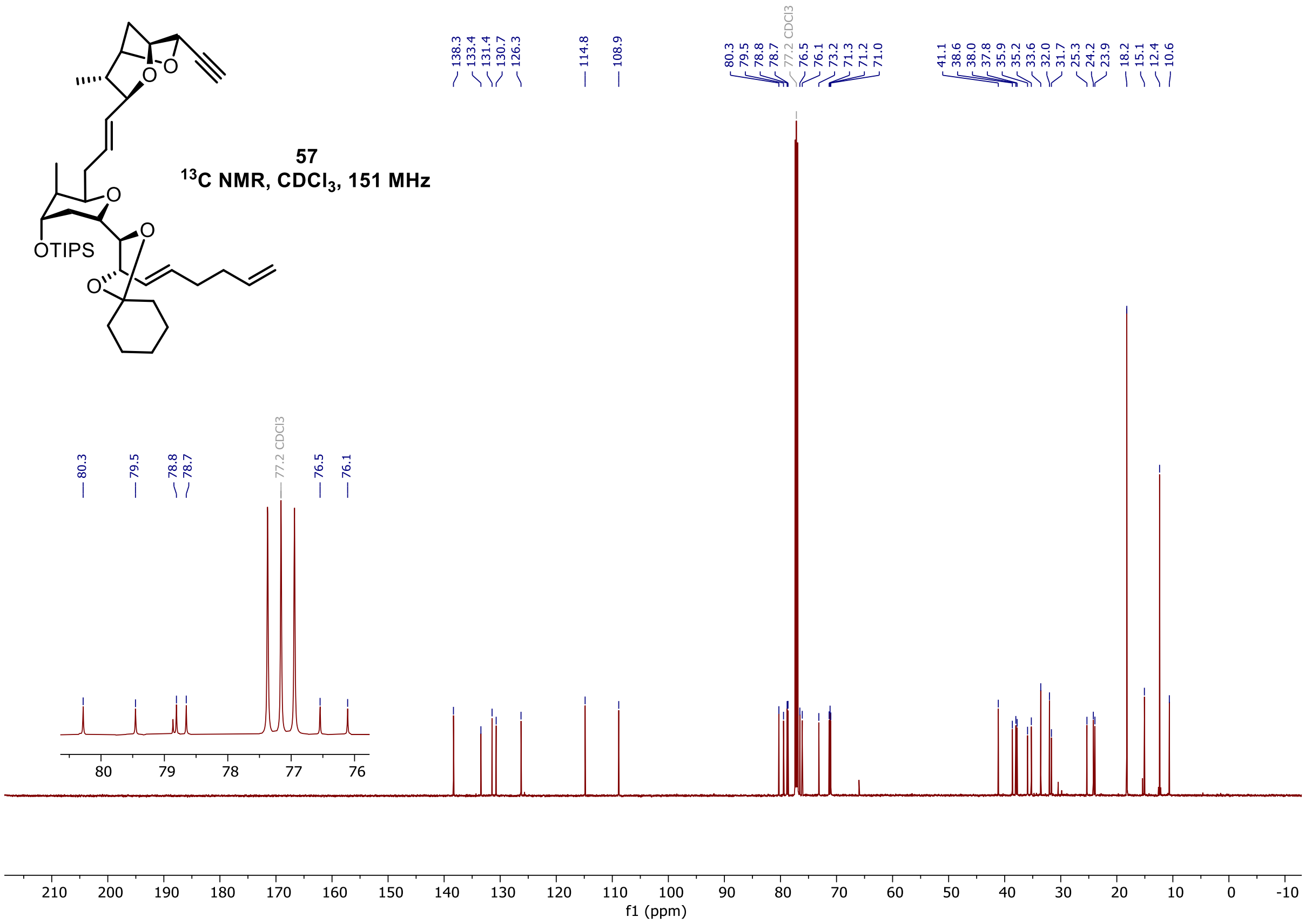

Vinyl iodide 58

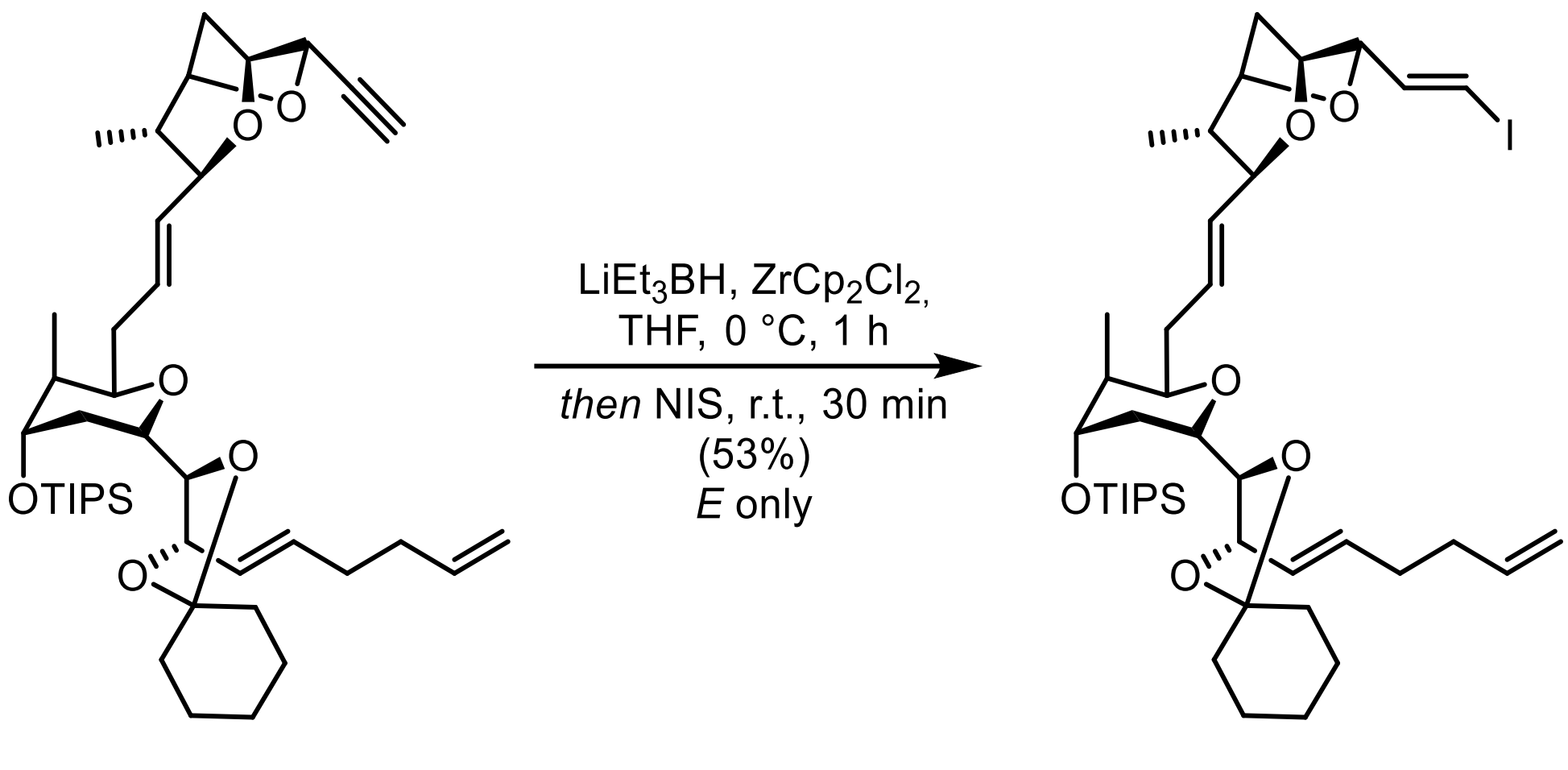

57 58

Alkyne **57** (40 mg, 58 µmol, 1.0 eq.) was dissolved in 1 mL dry THF, and cooled to 0 °C before adding a lithium triethylborohydride solution (1.0M in THF, 58 µL, 58 µmol, 1.0 eq.). In a separate flask, covered in aluminum foil, $ZrCp_2Cl_2$ (20 mg, 68 µmol, 1.2 eq.) was suspended in 0.5 mL dry THF and subsequently treated with lithium triethylborohydride solution (1.0M in THF, 116 µL, 116 µmol, 2.0 eq.). This mixture was stirred for 1 h in the dark, before the solution, containing the alkyne, was added to the generated Schwartz' reagent and stirred for 1 h at 0 °C. Then, NIS (33 mg, 147 µmol, 2.5 eq.) was added and the mixture was stirred for 30 min at r.t. The reaction was quenched with sat. $NaHCO_3$ solution and the aqueous layer was extracted with Et_2O. The combined organic layer was washed with sat. $Na_2S_2O_3$ solution and sat. NaCl solution, followed by drying over $MgSO_4$. After removal of the solvent in vacuo, the crude material was purified by flash chromatography (pentane/Et_2O, 3:1) to afford vinyl iodide **58** (25 mg, 31 µmol, 53% over two steps, *E* only) as a colorless oil.

$\mathbf{R_f}$ = 0.58 (pentane/Et_2O, 1:1), vanillin: blue, UV (-).

^{1}H NMR (600 MHz, $CDCl_3$) δ [ppm] = 6.86 – 6.80 (m, 1H), 6.57 (dd, *J* = 14.6, 1.8 Hz, 1H), 5.82 – 5.73 (m, 1H), 5.55 (dddd, *J* = 14.1, 5.0, 3.9, 2.4 Hz, 2H), 5.45 – 5.38 (m, 1H), 4.62 (ddd, *J* = 8.5, 5.0, 2.0 Hz, 1H), 4.36 (td, *J* = 2.6, 1.5 Hz, 1H), 4.31 (dt, *J* = 4.6, 2.1 Hz, 1H), 4.27 (d, *J* = 6.5 Hz, 1H), 3.96 (q, *J* = 2.9 Hz, 1H), 3.92 – 3.80 (m, 4H), 3.19 (t, *J* = 7.0 Hz, 2H), 2.30 – 2.22 (m, 1H), 2.20 – 2.07 (m, 3H), 1.98 (ddd, *J* = 11.6, 6.5, 2.8 Hz, 1H), 1.90 – 1.82 (m, 3H), 1.76 – 1.69 (m, 1H), 1.66 – 1.53 (m, 8H), 1.44 – 1.31 (m, 3H), 1.12 – 0.99 (m, 21H), 0.85 (d, *J* = 2.6 Hz, 3H), 0.83 (d, *J* = 2.2 Hz, 3H).

^{13}C NMR (151 MHz, $CDCl_3$) δ [ppm] = 141.1, 138.3,133.3, 131.8, 130.6, 126.5, 114.9, 108.9, 83.6, 79.6, 79.6, 79.4, 78.9, 78.6, 75.8, 73.1, 71.3, 71.0, 41.4, 38.8, 37.9, 37.8, 35.9, 35.3, 33.3, 31.7, 31.6, 30.3, 25.3, 24.2, 23.9, 18.3, 15.2, 12.4, 10.6, 7.0.

IR (ATR) ṽ (cm^{-1}) = 2961 (s), 2934 (s), 2864 (s), 2329 (w), 1460 (m), 1366 (m), 1279 (w), 1165 (m), 1142 (m), 1094 (s), 1066 (s), 967 (s), 939 (s), 881 (m), 710 (w), 678 (m), 658 (m), 410 (w).

HRMS (ESI) (*m/z*) calcd. for $[C_{41}H_{67}IO_6SiNH_4]^+$ = $[M+ NH_4]^+$: 828.4095, found: 828.4038.

Specific Rotation $[\alpha]_{589}^{20}$= –9.2° (c = 1.5, $CHCl_3$).

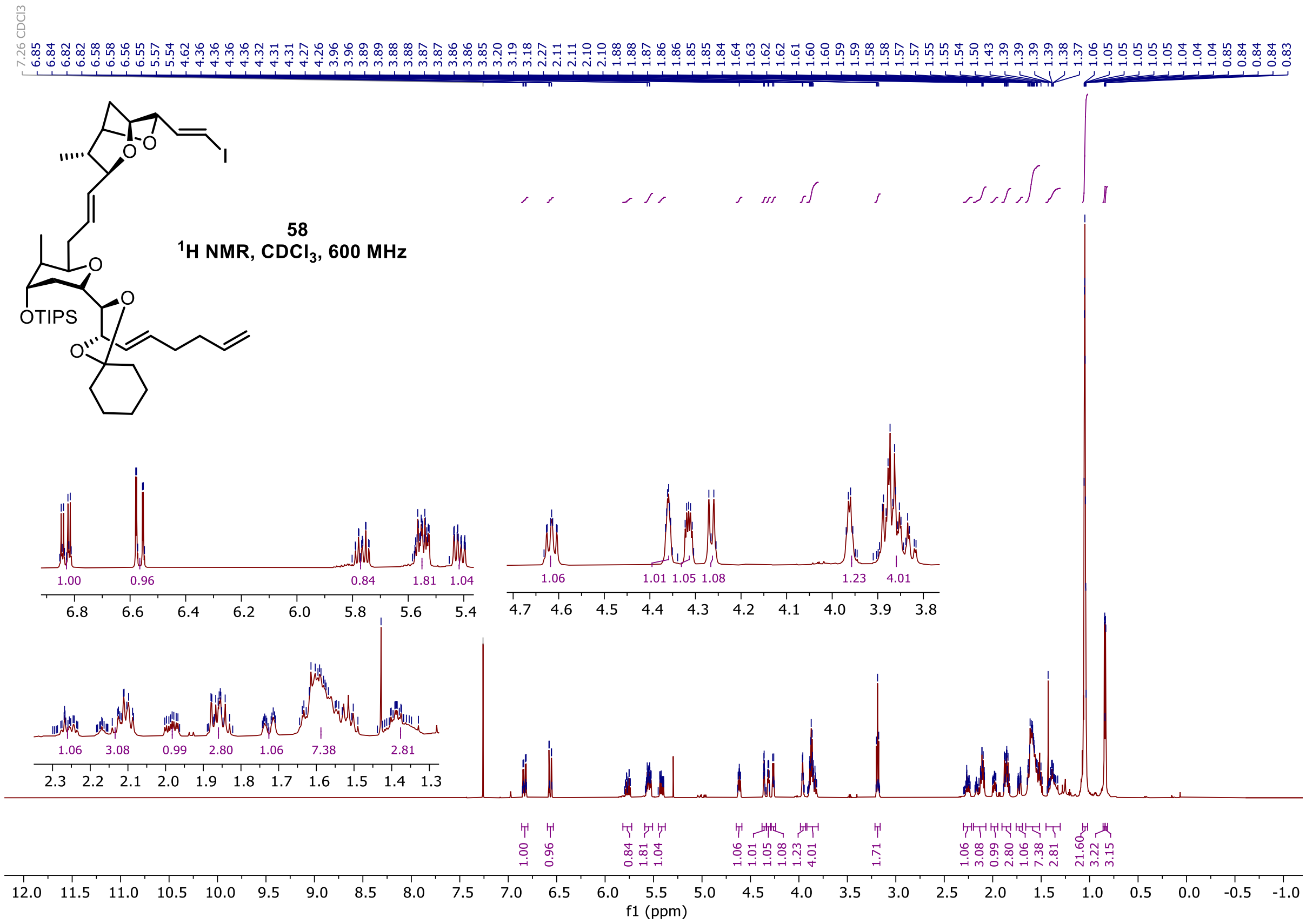
58
1H NMR, CDCl3, 600 MHz
OTIPS
f1 (ppm)

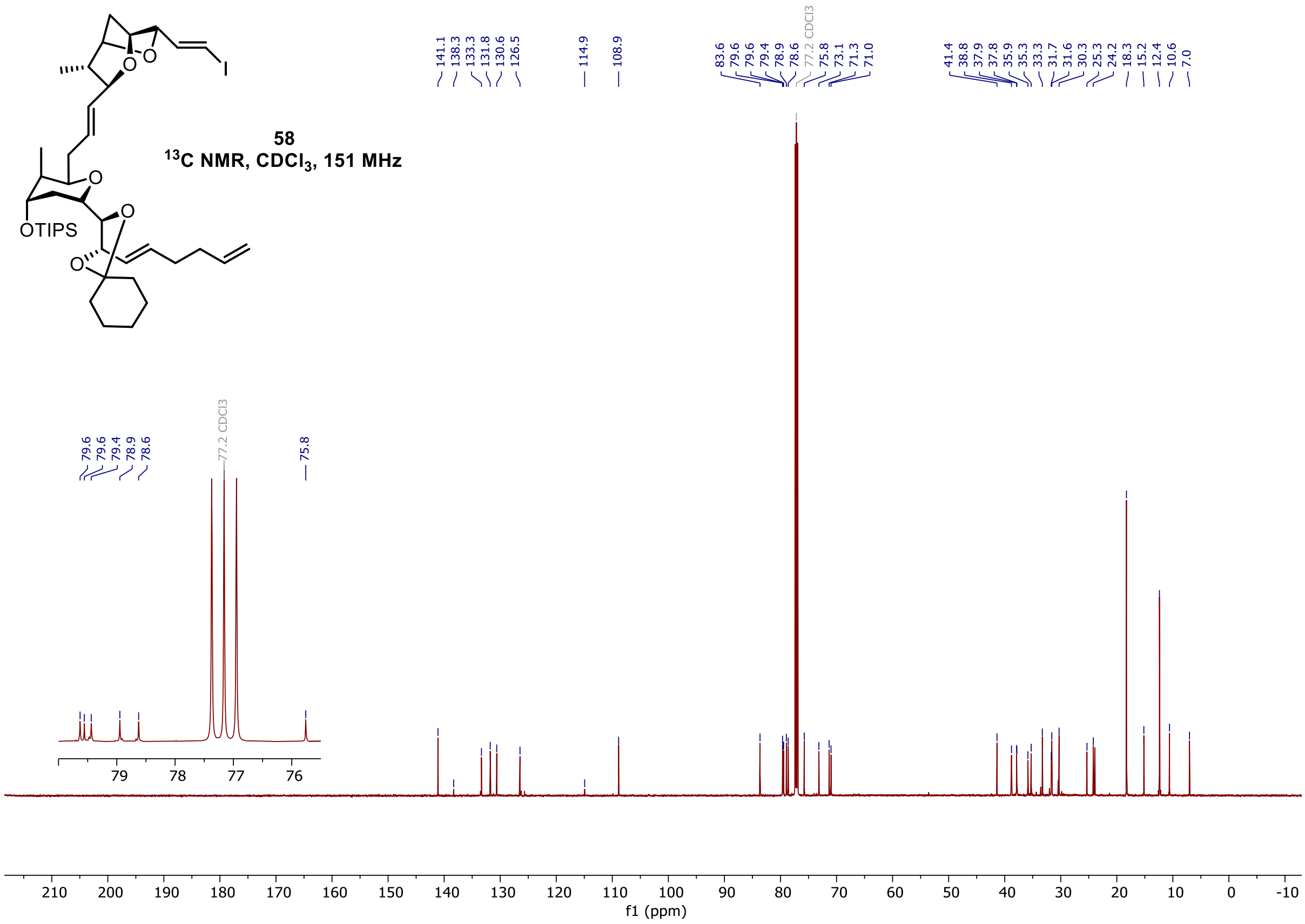
58
13C NMR, CDCl3, 151 MHz
OTIPS
f1 (ppm)

Triene 4

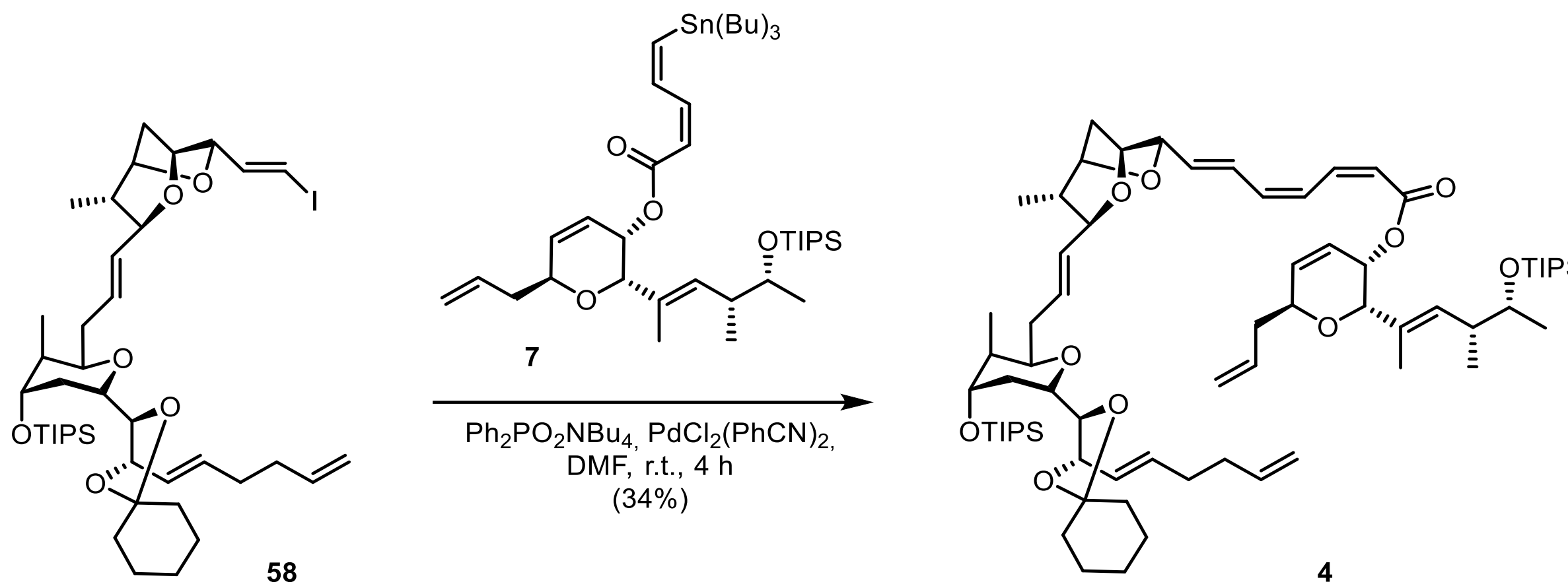

Tin ester **7** (17.5 mg, 22.5 µmol, 1.0 eq.), vinyl iodide **58** (20.0 mg, 24.7 µmol, 1.1 eq.), and $Ph_2PO_2NBu_4$ (69 mg, 148.0 µmol, 6.6 eq.) were dissolved in degassed dry DMF (1 mL). $PdCl_2(PhCN)_2$ (1.0 mg, 0.28 µmol, 12 mol%) was added, and the mixture was stirred for 4 h under exclusion of light in a glovebox under an argon atmosphere. The mixture was then diluted with Et_2O/hexane (1:1, 2 mL) and filtered through a short pad of silica into a sat. NaCl solution. The silica pad was washed with additional Et_2O/hexane (1:1), and the layers were separated. The aqueous phase was extracted with Et_2O/hexane (1:1), and the combined organic layers were washed with sat. NaCl solution, dried over Na_2SO_4, and concentrated under reduced pressure. The crude product was purified by column chromatography (15% EtOAc/hexane) to afford compound **4** (9.0 mg, 7.7 µmol, 34%).

R_f = 0.30 (hexane/EtOAc, 9:1), vanillin: dark blue, UV (-).

HRMS (ESI) (*m/z*) calcd. for $[C_{70}H_{114}O_{10}Si_2NH_4]^+$ = $[M+ NH_4]^+$: 1193.785, found: 1193.796.

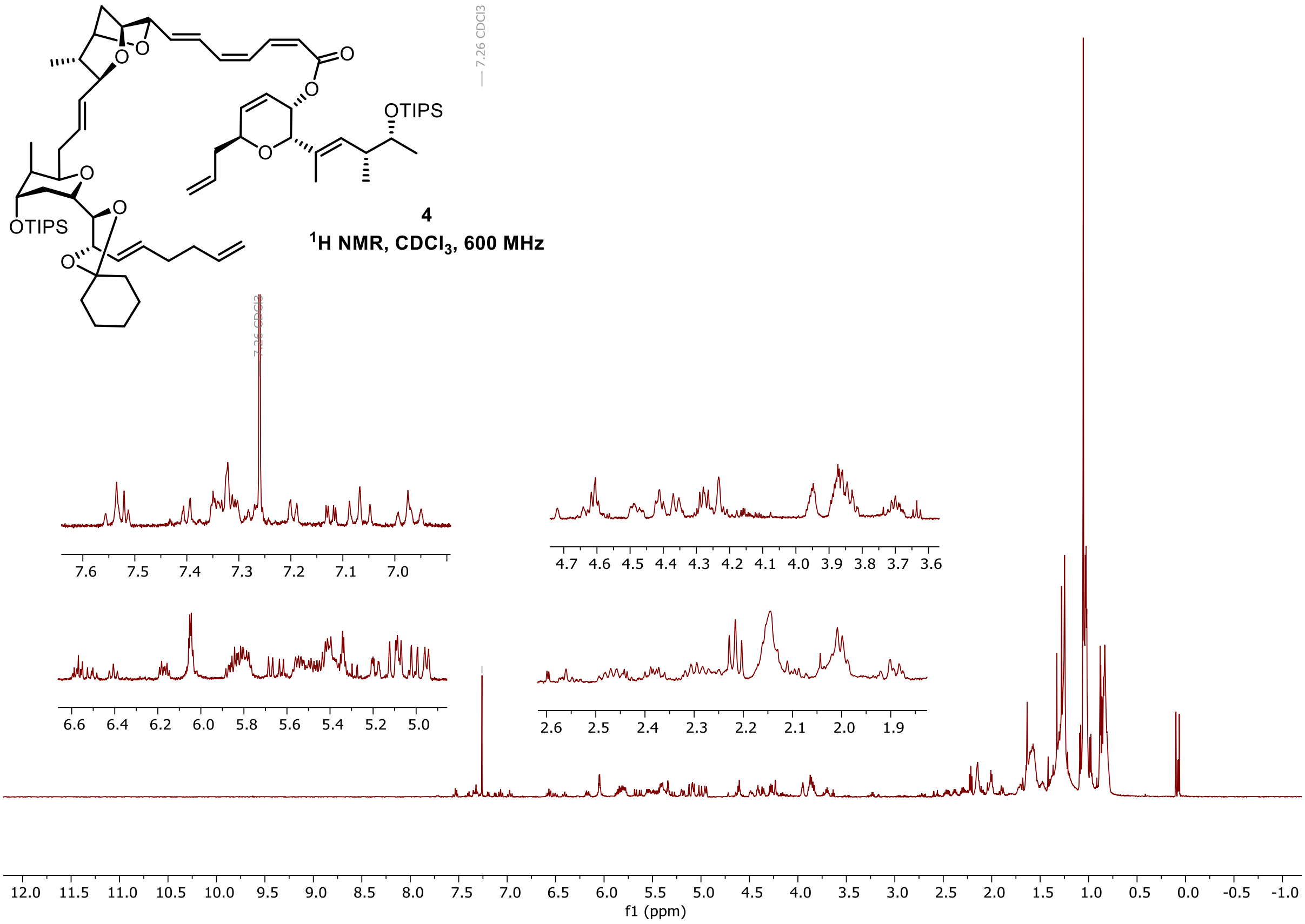

Protected synthetic (+)-neosorangicin A (59)

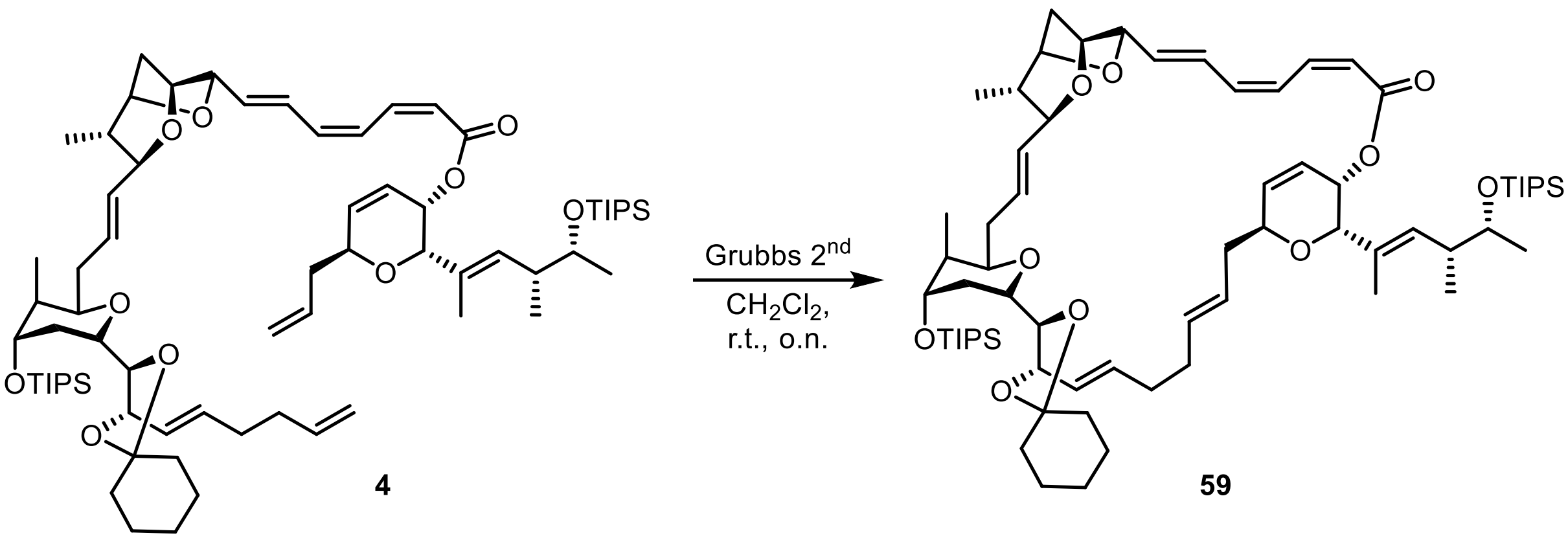

4

59

Compound **4** (7 mg, 6 µmol, 1.0 eq.) was dissolved in 10 mL dry, degassed CH_2Cl_2. Grubbs 2nd generation catalyst (1 mg, 1.2 µmol, 20 mol%) was dissolved in 3 mL dry, degassed CH_2Cl_2 and added dropwise to the reaction solution over 8 h. The reaction mixture was then stirred at r.t. overnight. After completion, the reaction mixture was concentrated under a stream of air, and the crude product was purified by flash chromatography (pentane/EtOAc, 1:1). Insufficient material was isolated for full analytical characterization; however, the exact mass of compound **59** was determined by LC–MS.

R_f =0.28 (*n*-hexane/EtOAc), vanillin: light blue, UV (-).

HRMS (ESI) (*m/z*) calcd. for $[C_{68}H_{110}O_{10}Si_2NH_4]^+$ = $[M+NH_4]^+$: 1160.7981, found: 1160.7958.

(+)-Neosorangicin A (1) (via relay approach from natural (+)-neosorangicin A)

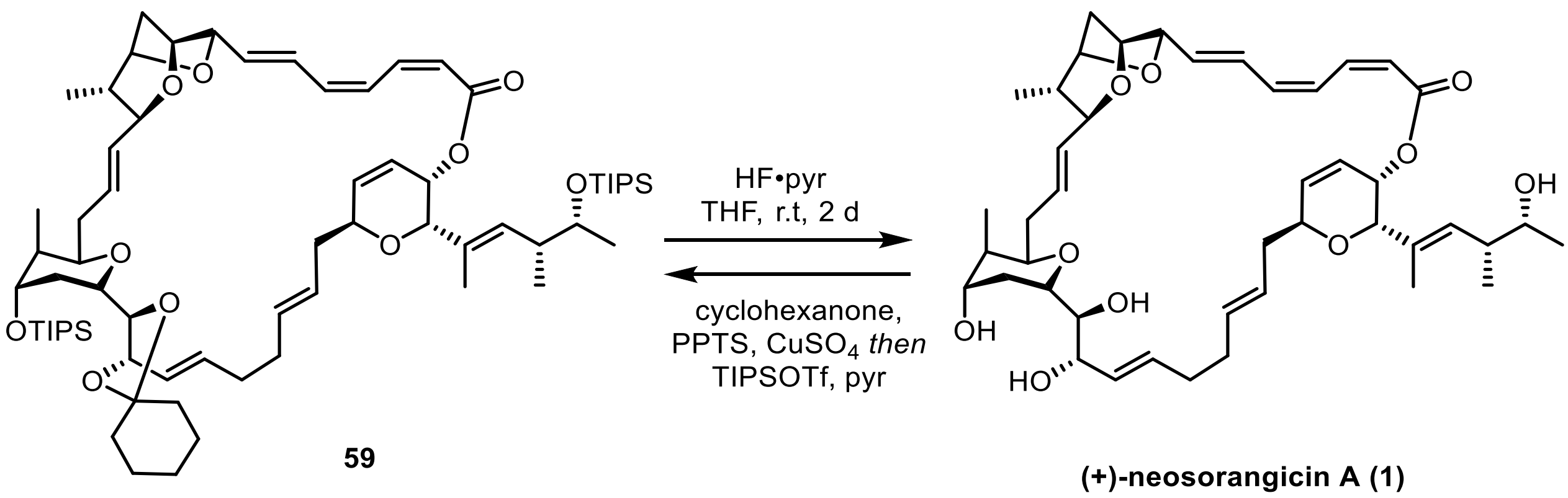

59 **(+)-neosorangicin A (1)**

Compound **59** (0.5 mg), prepared from a sample of natural (+)-neosorangicin A (**1**) by treatment with an excess of cyclohexanone and PPTS, was dissolved in dry THF (0.2 mL), and HF•pyridine (50 µL) was added. The reaction mixture was stirred at room temperature for 2 days. The reaction was co-spotted with natural (+)-neosorangicin A (**1**), and upon completion, the exact mass was determined by LC–MS.

R_f = 0.15 (EtOAc), vanillin: black, UV (+).

HRMS (ESI) (*m/z*) calcd. for $[C_{44}H_{62}O_{10}H]^+$ = $[M+H]^+$: 751.4421, found: 751.4421.

^{1}H NMR of protected natural (+)-neosorangicin A

600 MHz, CDCl$_3$, -1.2 – 12.2 ppm

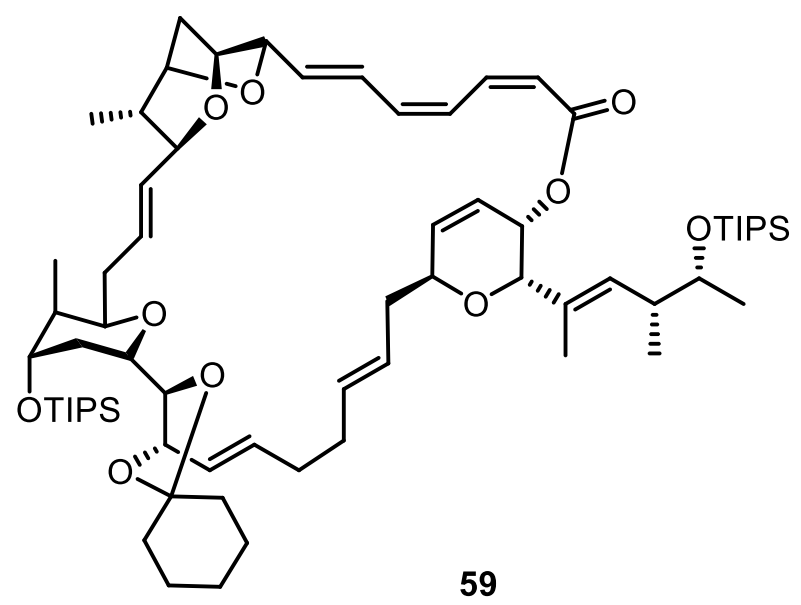

59

^{1}H NMR, CDCl$_3$, 600 MHz

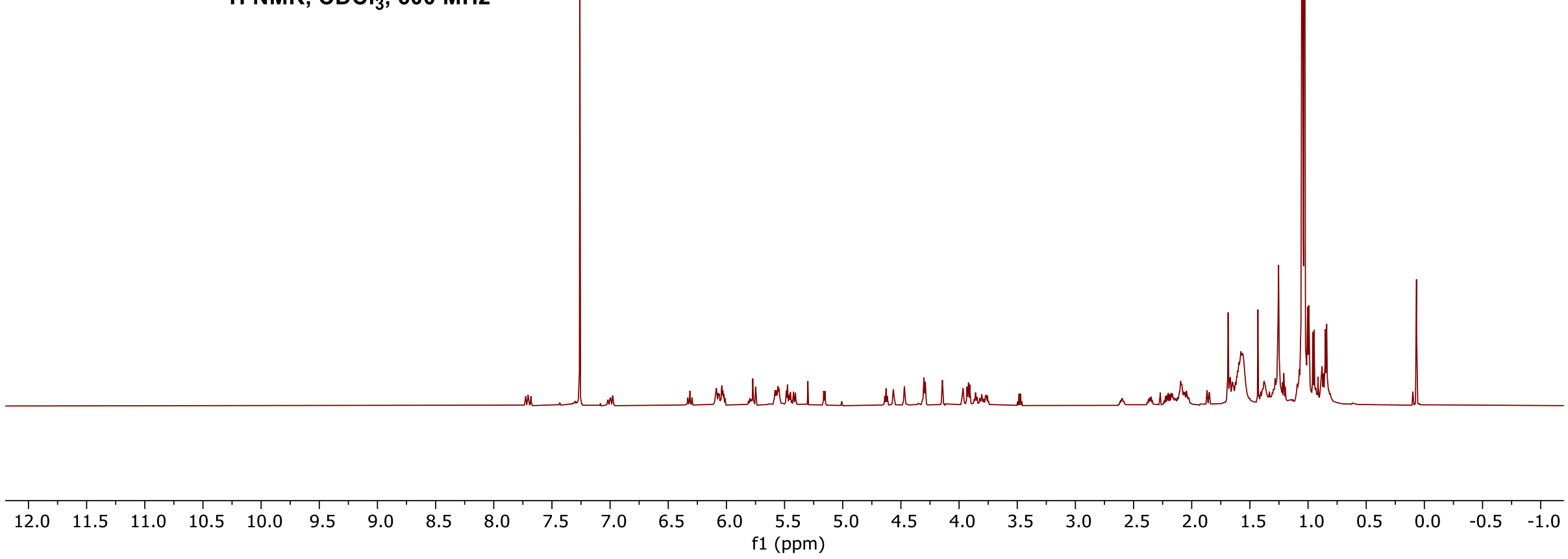

^{1}H NMR of protected natural (+)-neosorangicin A

600 MHz, CDCl$_3$, different regions

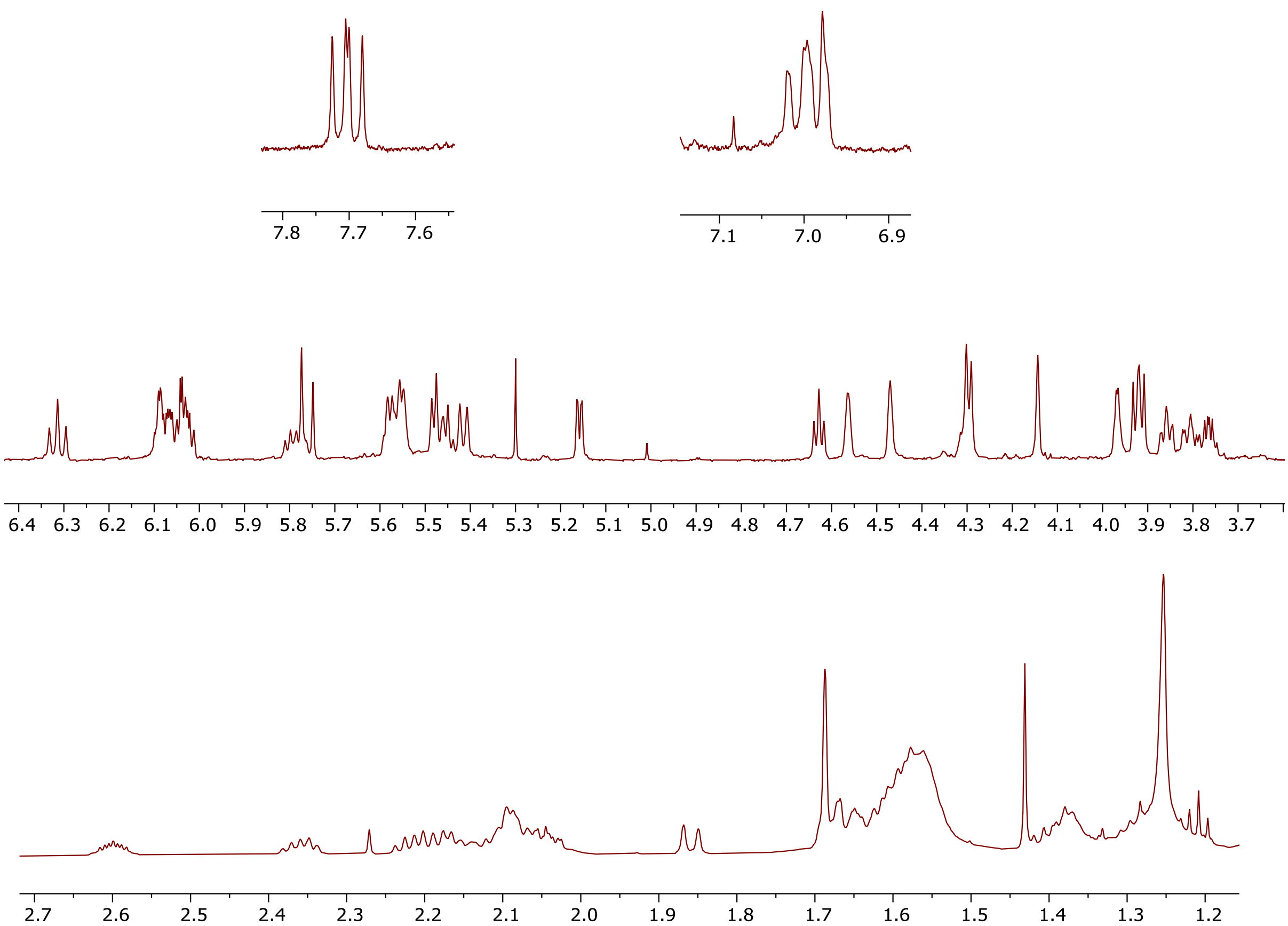

COSY NMR of protected natural (+)-neosorangicin A

600 MHz, CDCl$_3$, 0.0 – 9.5 ppm

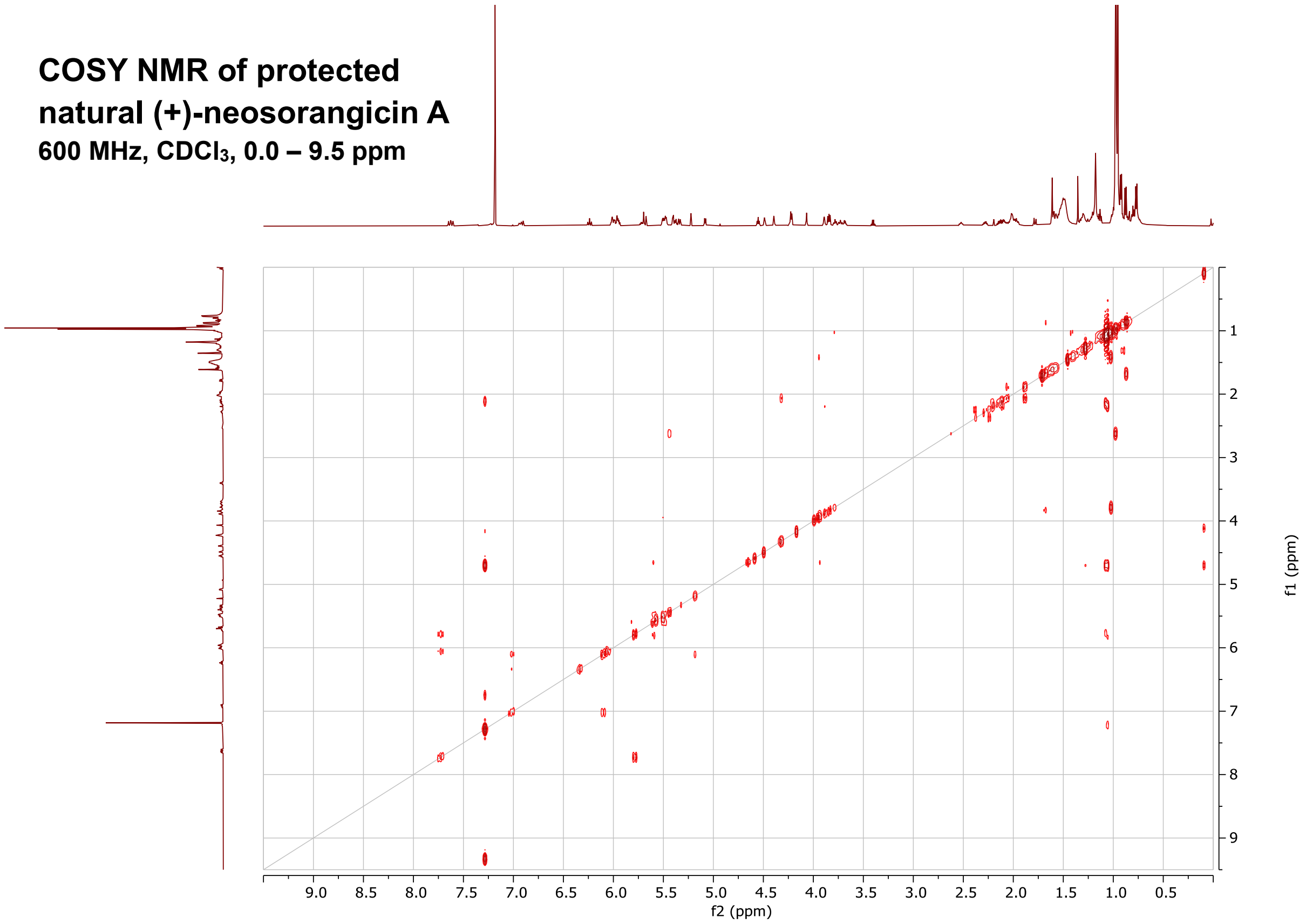

HSQC NMR of protected natural (+)-neosorangicin A

600 MHz, CDCl$_3$, -0.5 – 7.8 ppm

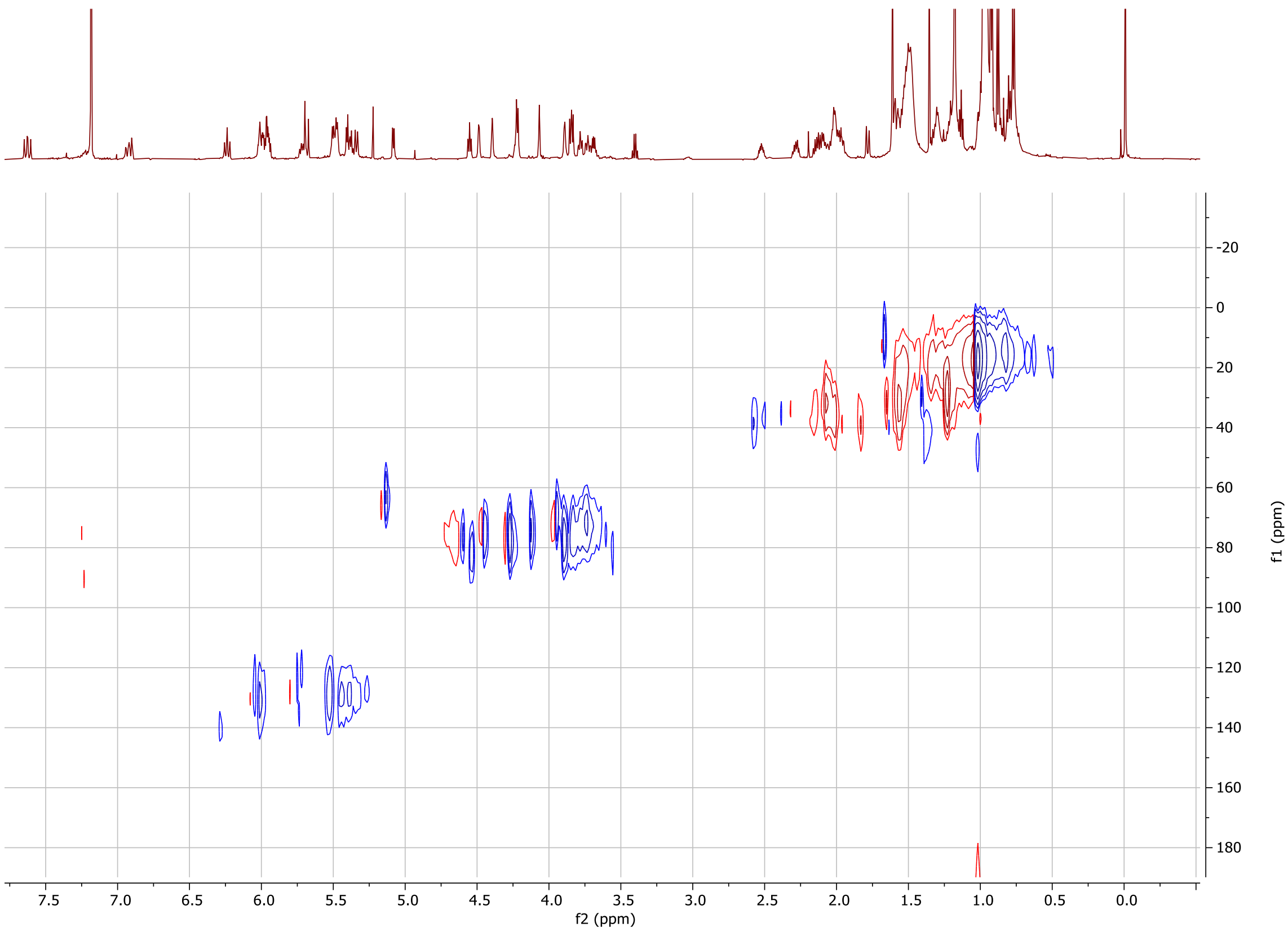

Protected natural (+)-neosorangicin A (59)

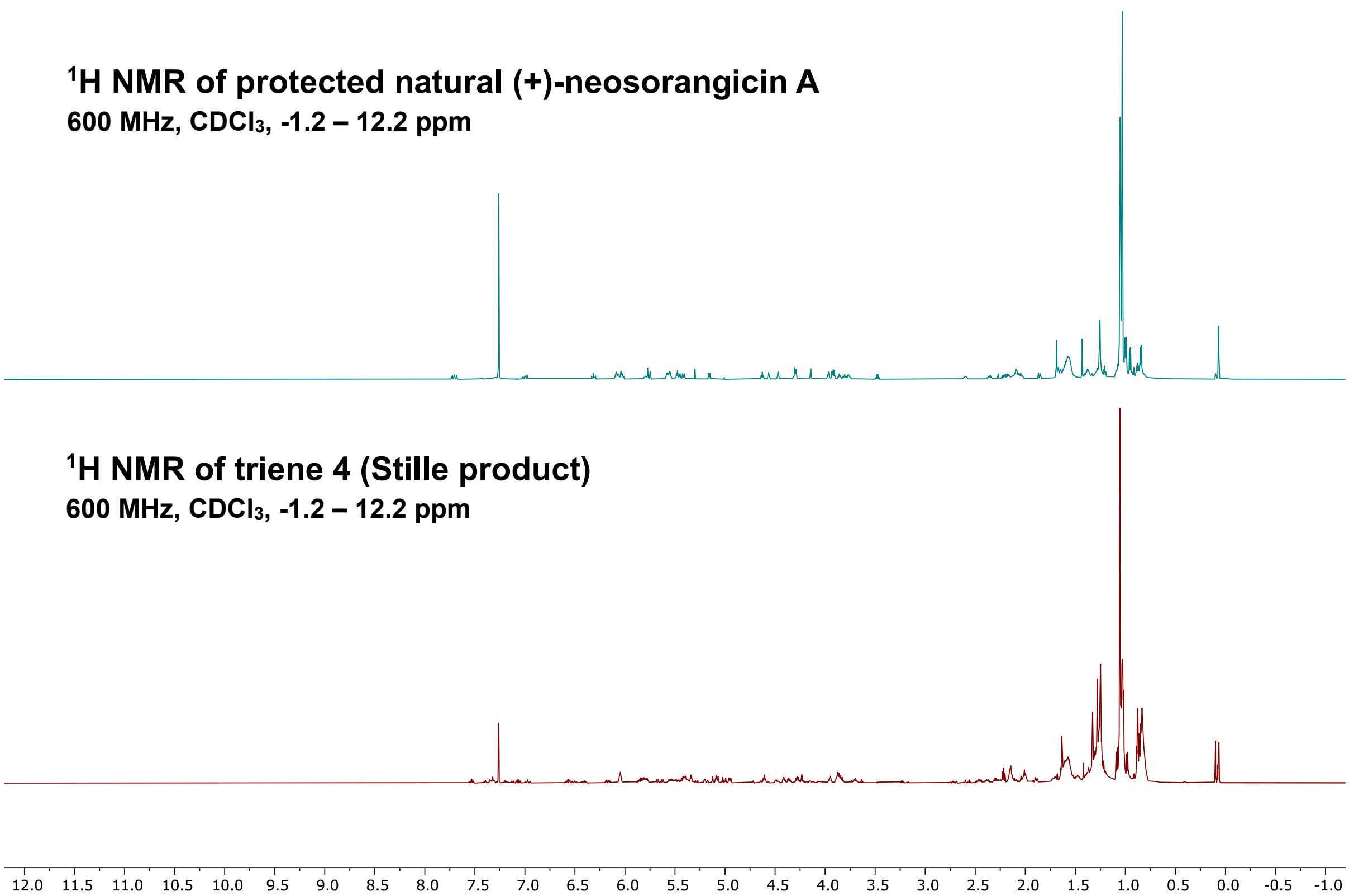
1H NMR of protected natural (+)-neosorangicin A
600 MHz, CDCl3, -1.2 – 12.2 ppm
1H NMR of triene 4 (Stille product)
600 MHz, CDCl3, -1.2 – 12.2 ppm
12.0 11.5 11.0 10.5 10.0 9.5 9.0 8.5 8.0 7.5 7.0 6.5 6.0 5.5 5.0 4.5 4.0 3.5 3.0 2.5 2.0 1.5 1.0 0.5 0.0 -0.5 -1.0

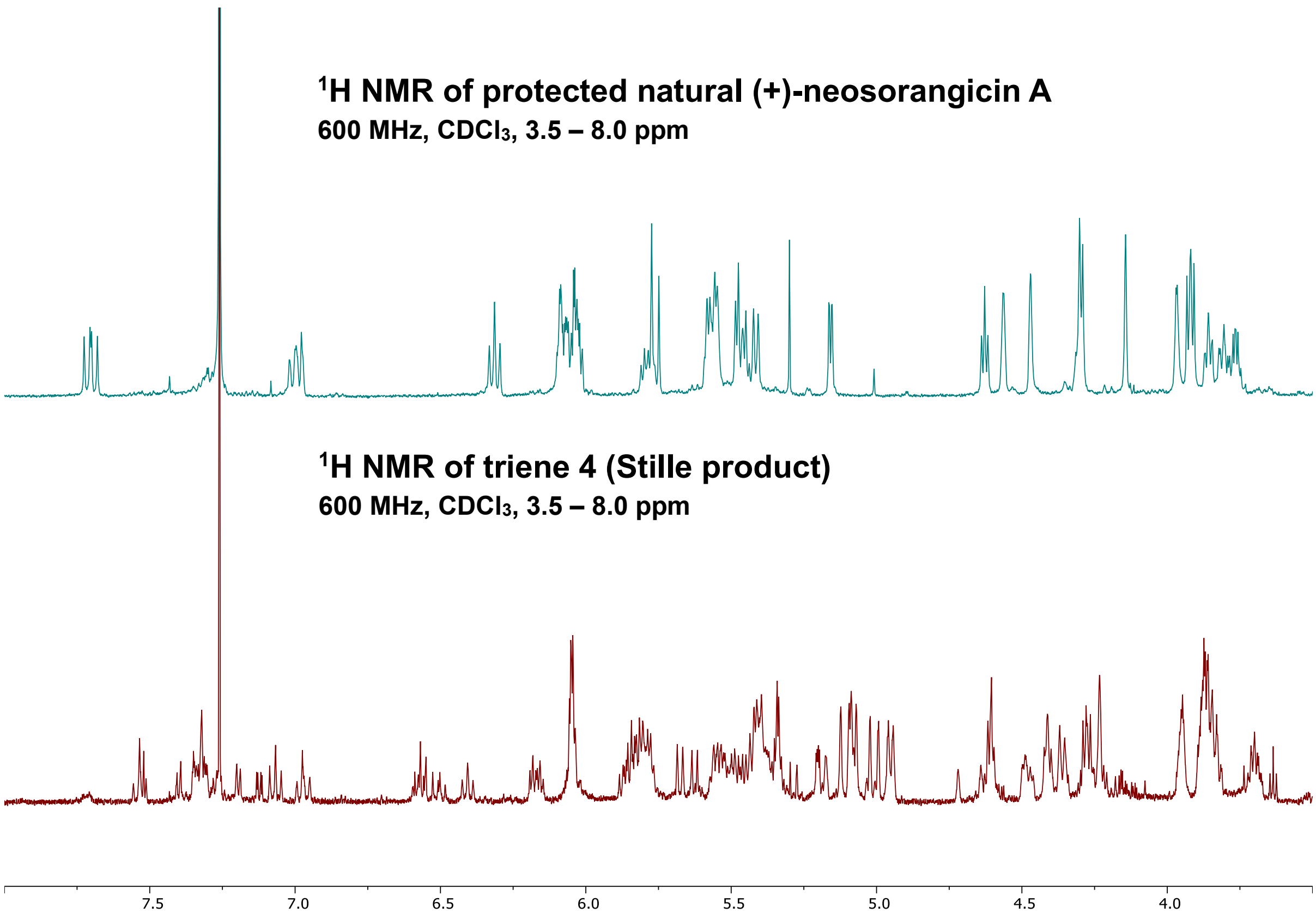
1H NMR of protected natural (+)-neosorangicin A
600 MHz, CDCl3, 3.5 – 8.0 ppm
1H NMR of triene 4 (Stille product)
600 MHz, CDCl3, 3.5 – 8.0 ppm
7.5 7.0 6.5 6.0 5.5 5.0 4.5 4.0

^{1}H NMR of naturally isolated (+)-neosorangicin A

600 MHz, acetone-d6, -1.0 – 8.0 ppm

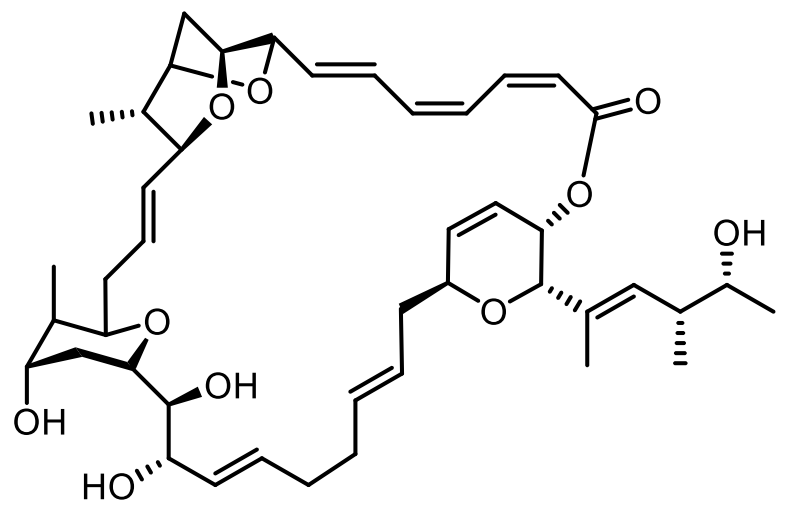

1

^{1}H NMR, acetone-d6, 600 MHz

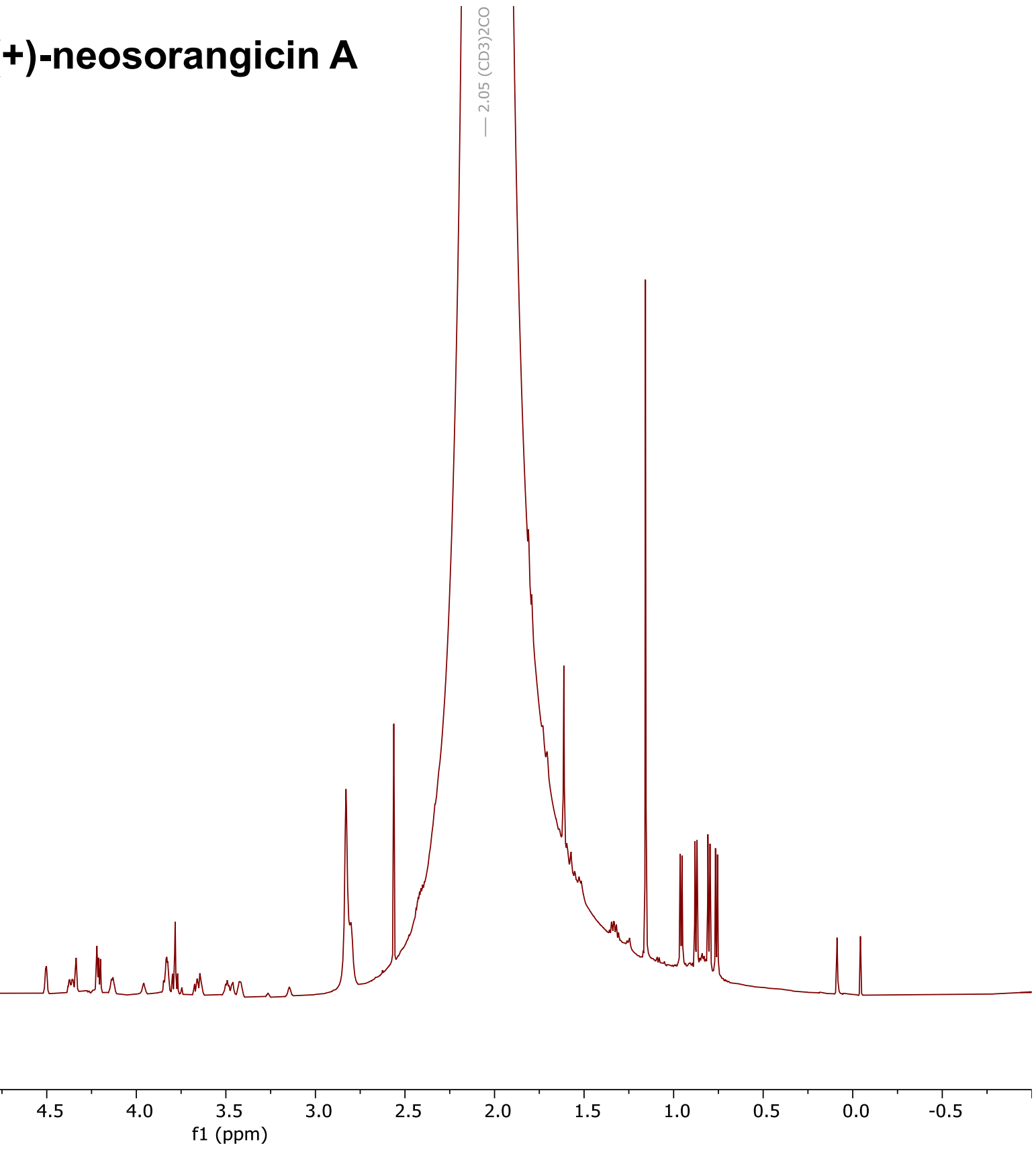

Supplement: Supplementary file 1 [file CHEM-32-e71360-s001.pdf]
